# Ruthenium(II) Polypyridyl Complexes Containing COUBPY Ligands as Potent Photosensitizers for the Efficient Phototherapy of Hypoxic Tumors

**DOI:** 10.1021/jacs.4c15036

**Published:** 2025-02-15

**Authors:** Diego Abad-Montero, Albert Gandioso, Eduardo Izquierdo-García, Sergi Chumillas, Anna Rovira, Manel Bosch, Mireia Jordà-Redondo, Davor Castaño, Joaquín Bonelli, Valentin V. Novikov, Alba Deyà, José Luis Hernández, Jorge Galino, Marta E. Alberto, Antonio Francés-Monerris, Santi Nonell, Gilles Gasser, Vicente Marchán

**Affiliations:** † Departament de Química Inorgànica i Orgànica, Secció de Química Orgànica, 152690 Universitat de Barcelona (UB), and Institut de Biomedicina de la Universitat de Barcelona (IBUB), Martí i Franquès 1-11, E-08028 Barcelona, Spain; ‡ 52845 Chimie ParisTech, PSL University, CNRS, Institute of Chemistry for Life and Health Sciences, Laboratory for Inorganic Chemical Biology, F-75005 Paris, France; § Unitat de Microscòpia Òptica Avançada, Centres Científics i Tecnològics, 16724 Universitat de Barcelona, Av. Diagonal 643, E-08028 Barcelona, Spain; ∥ Institut Químic de Sarrià, Universitat Ramon Llull, Vía Augusta 390, E-08017 Barcelona, Spain; ⊥ Departament de Química Inorgànica i Orgànica, Secció de Química Inorgànica, Universitat de Barcelona (UB), and Institute of Nanoscience and Nanotechnology of the University of Barcelona (IN2UB), Martí i Franquès 1-11, E-08028 Barcelona, Spain; # Health and Biomedicine Department, Leitat Technological Center, Carrer de la Innovació 2, E-08225 Terrassa, Spain; ∇ Dipartimento di Chimica e Tecnologie Chimiche, 428781 Università della Calabria, Arcavacata di Rende I-87036, Italy; ○ Institut de Ciència Molecular, 16781 Universitat de València, P.O. Box 22085, València 46071, Spain

## Abstract

Hypoxia, a hallmark of many solid tumors, is linked to increased cancer aggressiveness, metastasis, and resistance to conventional therapies, leading to poor patient outcomes. This challenges the efficiency of photodynamic therapy (PDT), which relies on the generation of cytotoxic reactive oxygen species (ROS) through the irradiation of a photosensitizer (PS), a process partially dependent on oxygen levels. In this work, we introduce a novel family of potent PSs based on ruthenium­(II) polypyridyl complexes with 2,2′-bipyridyl ligands derived from COUPY coumarins, termed COUBPYs. Ru-COUBPY complexes exhibit outstanding *in vitro* cytotoxicity against CT-26 cancer cells when irradiated with light within the phototherapeutic window, achieving nanomolar potency in both normoxic and hypoxic conditions while remaining nontoxic in the dark, leading to impressive phototoxic indices (>30,000). Their ability to generate both Type I and Type II ROS underpins their exceptional PDT efficiency. The lead compound of this study, **SCV49**, shows a favorable *in vivo* pharmacokinetic profile, excellent toxicological tolerability, and potent tumor growth inhibition in mice bearing subcutaneous CT-26 tumors at doses as low as 3 mg/kg upon irradiation with deep-red light (660 nm). These results allow us to propose **SCV49** as a strong candidate for further preclinical development, particularly for treating large hypoxic solid tumors.

## Graphical abstract

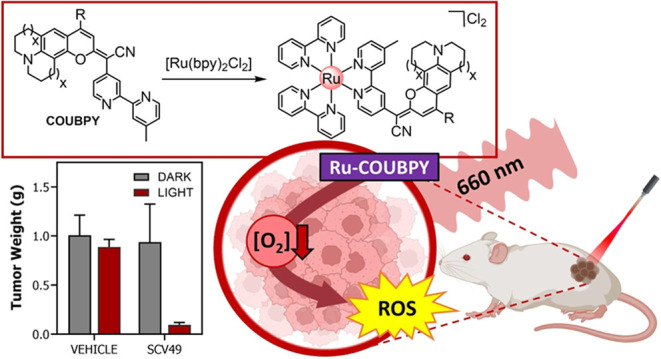

## Introduction

Hypoxia, or low oxygen concentration, is a feature commonly found in aggressive solid tumors, such as glioblastoma, colorectal, pancreatic, and breast cancers.^1^ While the oxygen level in normal tissues is typically above 40 mmHg, hypoxic areas within tumors have oxygen levels below 10 mmHg (equivalent to 1–2% O_2_ or even below) due to rapid tumor cell proliferation and abnormal blood vessel formation.^2^ This low-oxygen environment promotes tumor angiogenesis, metastasis, and resistance to conventional treatments like chemotherapy, radiotherapy, and immunotherapy, leading to poorer patient outcomes and a higher risk of cancer recurrence.^3,4^

Photodynamic therapy (PDT) is a clinically approved method for eradicating tumors and/or tumor vasculature that uses light-responsive drugs known as photosensitizers (PSs).^5–7^ This technique involves administering locally or systemically a nontoxic dose of a PS, followed by light activation directly at the tumor site, producing a series of highly cytotoxic reactive oxygen species (ROS) that cause cell damage and ultimately lead to tumor cell death. Besides these direct effects, PDT-stimulated immune response also induces local acute inflammation, whereas the phototriggered vascular damage can lead to tumor infarction.^8,9^ PDT is also effective at treating other conditions, such as actinic keratosis, age-related macular degeneration, and some fungal and microbial infections. PSs can operate through two main mechanisms: Type I and Type II. On the one hand, the Type II mechanism involves sensitizing singlet oxygen (^1^O_2_) through an energy-transfer process from the excited triplet state of the PS to molecular oxygen in the ground state. On the other hand, the Type I PDT mechanism is based on electron transfer reactions that generate a variety of ROS, such as superoxide (^•^O_2_^–^) and hydroxyl (^•^OH) radicals. While the effectiveness of Type II PDT relies heavily on surrounding oxygen levels, the Type I PDT mechanism can remain effective even in low-oxygen environments, presenting a promising approach for addressing the hypoxia problem in cancer therapy.^10,11^

Compared to conventional cancer treatments, PDT offers several advantages, such as noninvasiveness and spatial and temporal selectivity, which are associated with much milder and localized side effects. However, it still faces significant challenges that limit its broad clinical application. To date, most marketed photosensitizers based on the well-known tetrapyrrolic scaffold, including porphyrins, chlorins, and phthalocyanines, share three main limitations: (i) dark toxicity, which causes undesired side effects and limits the dose patients can receive; (ii) reduced effectiveness in hypoxic tumors due to their reliance on the Type II PDT mechanism; and (iii) activation by short-wavelength light, limiting tissue penetration and access to larger tumors. Furthermore, this kind of PSs often suffer from poor water solubility and prolonged skin photosensitivity and requires complex synthetic processes that produce mixtures of compounds. As the incidence of cancer continues to rise worldwide, the global PDT market is rapidly expanding and requires alternative PSs beyond traditional tetrapyrrolic scaffolds. Ideally, these new PSs should be activatable by long-wavelength light (deep-red to near-infrared, NIR) and work through both Type I and Type II mechanisms to effectively treat large hypoxic solid tumors.^12–20^ Additionally, to enhance therapeutic efficacy and minimize toxicity, an ideal PS should preferentially accumulate in key subcellular organelles, such as mitochondria, that are essential for several crucial cellular processes.^21–23^

Metal-based PSs hold great promise for anticancer PDT due to their unique properties, as illustrated by the entrance of the Ru­(II) polypyridyl complex TLD-1433 in clinical trials.^24^ These transition metal complexes feature multiple electronic excited states that enable efficient ROS-generating photoreactions, and their modular three-dimensional architecture allows for easy modification of their chemical structures to optimize photophysical, photochemical, and photobiological properties through the careful selection of appropriate ligand–metal combinations.^25–28^ However, despite recent advances, particularly with cyclometalated Ir­(III) complexes as well as Ru­(II) and Os­(II) polypyridyl complexes^29–40^ most metal-based PSs still share some of the drawbacks of traditional tetrapyrrolic-based PSs, including high dark toxicity, reduced efficacy under hypoxia, and activation with relatively short-wavelength light. Photoactivated chemotherapy (PACT) using Ru­(II) complexes also offers great potential for the treatment of hypoxic tumors due to its oxygen-independent mechanism, which is based on the release of a bioactive cargo molecule from a caged compound upon light irradiation.^41–44^ Additionally, several strategies have been developed to enhance PDT efficiency in hypoxic tumors by increasing oxygen availability within the tumor microenvironment.^45–47^

Organic fluorophores, particularly those operating in the optical window of biological tissues (600–900 nm), are essential tools for bioimaging applications and phototherapies. We recently developed a new family of coumarin-based deep-red/NIR fluorophores, known as COUPYs, based on the incorporation of a cyano­(1-alkyl-4-pyridin-1-ium)­methylene group at position 2 of the coumarin backbone (Figure 1A).^48^ The photophysical properties of COUPY dyes can be easily tuned with minimal structural modifications,^49,50^ making them suitable for fluorescently labeling biomolecules.^51,52^ Additionally, COUPY fluorophores show significant potential as PDT agents, whether in their free form,^53^ nanoencapsulated,^54^ or when conjugated to transition metal complexes.^55–58^

**Figure 1:**
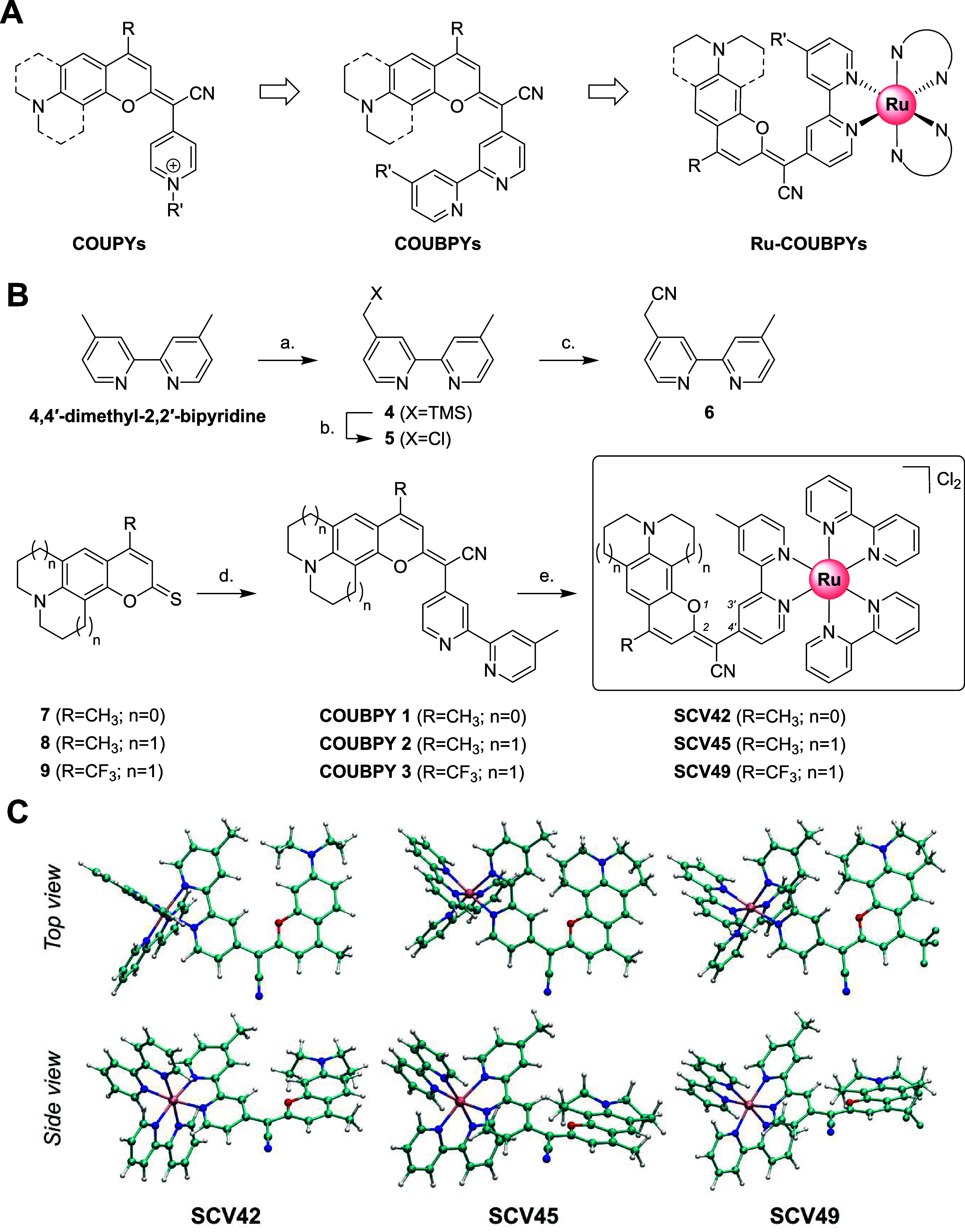
Rational design, synthesis, and characterization of Ru-COUBPY complexes. (A) Design of the COUBPY ligands and of the corresponding Ru­(II) polypyridyl complexes. (B) Synthetic route for the preparation of COUBPY ligands **1–3** and Ru-COUBPY complexes **SCV42**, **SCV45**, and **SCV49**. Reagents and conditions: (a) (1) LDA, THF, – 78 °C, 1 h, (2) TMSCl, – 78 °C, 10 s, (3) EtOH, – 78 °C to rt, 1 h, 76%; (b) (CCl_3_)_2_, CsF, ACN, 60 °C, 3.5 h, 57%; (c) KCN, 18-crown-6, ACN, rt to 50 °C, overnight, 64%; (d) (1) NaH, 6, ACN, rt, 3 h, (2) AgNO_3_, rt, 2 h, 20–75%; (e) [Ru­(bpy)_2_Cl_2_], EtOH-H_2_O (3:1), 80 °C, overnight, 62–93%. (C) Ground-state geometries of Ru-COUBPY complexes in ACN optimized by the PBE0/6-31+G­(d,p)/SDD method in ACN.

Building on these precedents, in this work, we describe the first development of a new family of PSs based on Ru­(II) polypyridyl complexes incorporating unprecedented 2,2′-bipyridyl ligands derived from COUPY coumarins, termed COUBPYs, in the metal coordination sphere (Figure 1A). These PSs exhibit exceptional *in vitro* cytotoxicity against cancer cells upon irradiation with light within the phototherapeutic window, under both normoxic and hypoxic conditions, while remaining nontoxic in the dark. The strong phototoxic activity of Ru-COUBPY PSs under hypoxia can be attributed to their ability to simultaneously photogenerate Type I and Type II ROS, providing a distinct advantage over current marketed PSs that primarily rely on the latter mechanism. Moreover, the results from the *in vivo* safety and efficacy studies in mice underscore the potential of Ru-COUBPY PSs, particularly the lead compound **SCV49** (Figure 1B), as promising candidates for further preclinical development in the PDT treatment of challenging hypoxic tumors.

## Results and Discussion

### Design, Synthesis, and Chemical Characterization of Ru-COUBPY PSs

Ru-COUBPY complexes were successfully obtained following the synthetic strategies depicted in Figure 1B. First, the required COUBPY ligands **1**–**3** incorporating 2,2′-bipyridine (bpy) at position 2 of the coumarin skeleton were synthesized through a condensation reaction between suitable thiocoumarin derivatives and a 2,2′-bipyridyl acetonitrile precursor (**6**), which was prepared from the commercially available 4,4′-dimethyl-2,2′-bipyridine. Based on previous structure–photophysical property relationships within the COUPY scaffold,^50,57^ the *N,N*-dialkylamino benzene group in COUBPY **1** was replaced with a julolidine moiety (**2** and **3**) to achieve a redshift in the absorption and emission maxima. Similarly, the incorporation of a strong electron-withdrawing CF_3_ group at position 4 of the coumarin backbone in COUBPY **3** was anticipated to cause a further redshift and enhance photostability.^48,49^ Three Ru-COUBPY complexes, **SCV42**, **SCV45** and **SCV49**, were assembled by reaction between COUBPY ligands **1**, **2** and **3**, respectively, and a Ru­(II) dichlorido complex precursor, [Ru­(bpy)_2_Cl_2_], in a EtOH/H_2_O 3:1 (v/v) mixture at 80 °C overnight. The complexes were easily isolated by silica column chromatography with good yields (62–93%) and fully characterized by 1D ^1^H and ^13^C NMR, 2D ^1^H,^1^H NOESY NMR and HRMS. The purity of the products was assessed by reversed-phase HPLC-MS analysis, revealing a single peak in all cases (Figure S1). Interestingly, as previously found in COUPY fluorophores, the ^1^H NMR spectra of Ru-COUBPY complexes showed two sets of proton signals, the proportion of which remained nearly the same as in the case of the free COUBPY ligand (≈90–95:10–5). The same duplicity was found in the ^13^C and ^19^F (only for **3** and **SCV49**) NMR spectra. The presence of exchange cross-peaks in the NOESY spectra (e.g., see Figure S23 for **SCV42**) confirmed the existence of rotamers in solution around the exocyclic double bond connecting the C2 of the coumarin moiety and the C4 of the bipyridine, which accounts for the strong electronic delocalization along the π-system of the COUBPY ligand. In all cases, the presence of characteristic NOE cross-peaks confirmed that the *E* rotamer was the major species in solution (Figures S20–S25). In coherence with this finding, the molecular models of Ru-COUBPY complexes shown in Figure 1C have been built in the predominant *E* disposition.

### Photophysical Characterization: Experimental and Computational Studies

The photophysical properties of Ru-COUBPY complexes were experimentally measured in acetonitrile (ACN) at room temperature. As shown in Table 1 and Figure 2A, the absorption spectra of the Ru-COUBPY complexes differ significantly from that of the reference Ru­(II) polypyridyl complex [Ru­(bpy)_3_]­Cl_2_, due to the replacement of one bpy ligand with COUBPY ligands. The strong absorption band around 450 nm in [Ru­(bpy)_3_]­Cl_2_, assigned to the metal-to-ligand charge transfer (MLCT) transition, is slightly red-shifted in the Ru-COUBPY complexes. Furthermore, the spectra of the Ru-COUBPYs exhibit additional bands beyond 500 nm, that are not present in the [Ru­(bpy)_3_]^2+^ molecule, and in which computations reveal a contribution from COUPBY ligands (*vide infra*). In the cases of **SCV42** and **SCV45**, two sharp almost fused bands appear in the 500–600 nm region. Remarkably, **SCV49** exhibits a broader band centered at 570 nm with some weak absorption extending beyond 700 nm.

**Figure 2:**
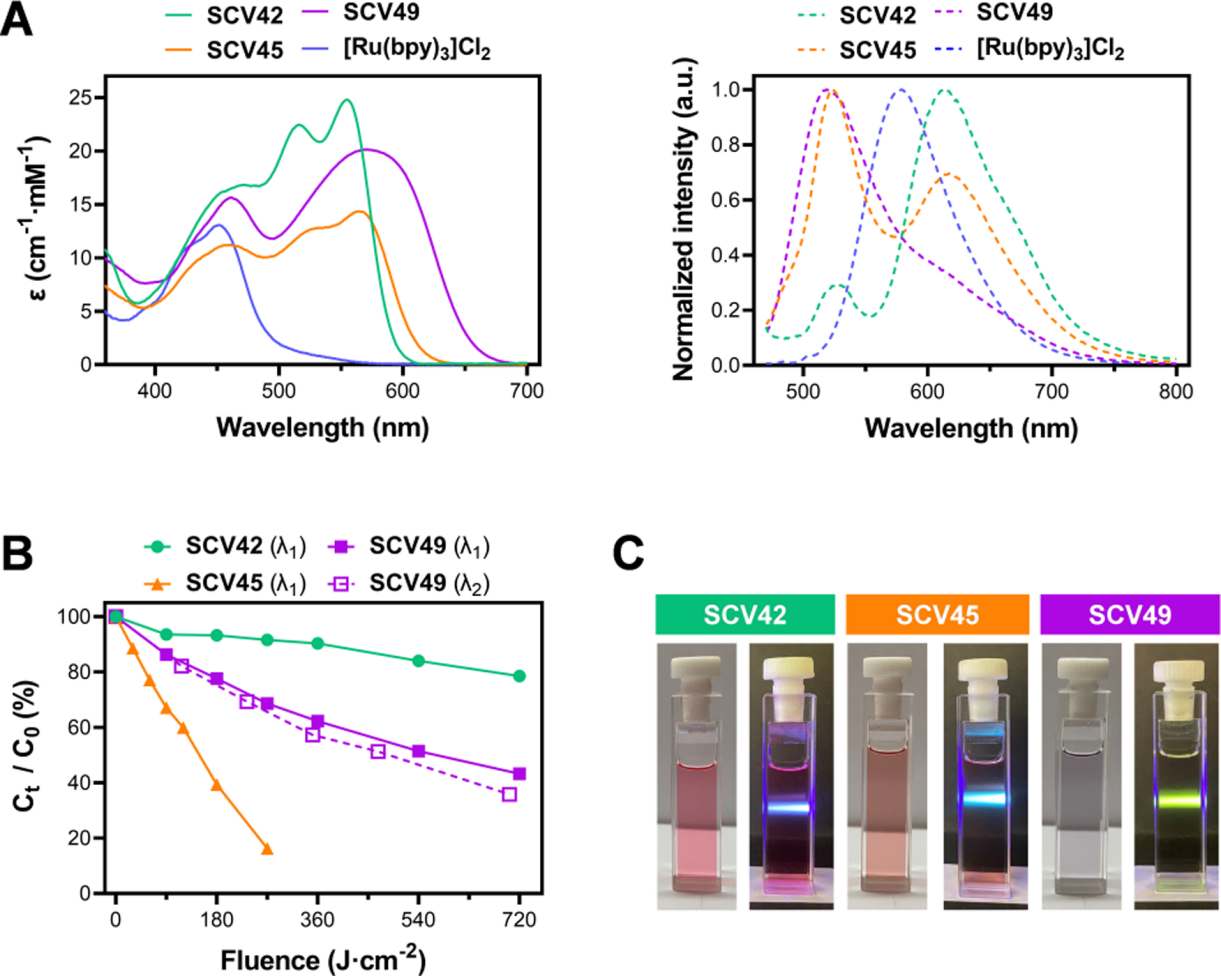
Photophysical characterization of Ru-COUBPY complexes. (A) Absorption (left panel) and emission (λ_exc_ = 460 nm) (right panel) spectra of the Ru-COUBPY complexes in ACN. (B) Photostability of the complexes in supplemented cell culture medium at 37 °C after irradiation with green (λ_1_ = 505 ± 35 nm, 100 mW cm^–2^) or red (λ_2_ = 620 ± 15 nm; 130 mW cm^–2^) light. *C*_0_ and *C_t_* represent the concentration of the compound at the beginning of the experiment (*t* = 0) and at various time points throughout the experiment, respectively. (C) Photographic images of Ru-COUBPY complex solutions (50 μM) in DCM under daylight (left panel) and in the dark (right panel) upon irradiation with a blue laser (405 nm).

**Table 1:** Photophysical Properties and Singlet Oxygen Quantum Yields of Ru-COUBPY Complexes in ACN at Room Temperature^a^

	spectroscopic properties					singlet oxygen quantum yield Φ_Δ_	
	λ_abs_/nm (ε/mM^–1^ cm^–1^ × 10^3^)	λ_em_/nm (460 nm)	λ_em_/nm (520 nm)	λ_em_/nm (600 nm)	τ air/ns	direct (532 nm)	indirect (505 nm)
**SCV42**	289 (53), 472 (17), 520 (22), 555 (25)	527, 612	599, 667	-	3.7, 116	0.33	0.48
**SCV45**	289 (43), 460 (11), 515 (12), 564 (14)	523, 617	616	632	3.8, 126	0.19	0.32
**SCV49**	289 (55), 461 (16), 571 (20)	519	664	667	5.5, 148	0.12	0.21

a Absorption (λ_abs_) maxima wavelengths, molar absorption coefficients at λ_abs_ (ε), emission (λ_em_) maxima wavelengths at the indicated λ_exc_, emission lifetimes (τ), and singlet oxygen quantum yield (Φ_Δ_) by direct and indirect method upon excitation at the indicated wavelengths.

The emission properties of the Ru-COUBPY complexes were investigated by using excitation at three different wavelengths (460, 520, and 600 nm) (Figures 2A and S26). When excited within the COUBPY absorption band (λ_exc_ = 520 or 600 nm), all three complexes exhibit emission signals in the far-red to NIR region. As expected, **SCV49** shows a significantly red-shifted emission maximum (λ_em_ = 667 nm) upon excitation at 600 nm, compared to **SCV42** and **SCV45**. However, it is worth noting that the spectra of the Ru-COUBPY complexes are not the simple sum of those of [Ru­(bpy)_3_]^2+^ and the appended coumarins, indicating some degree of mixing of their excited states. Indeed, the wavelength dependence of the emission spectra reflects different deactivation pathways depending on the nature of the originally excited chromophore. Time and spectrally resolved luminescence spectroscopy nevertheless confirmed the presence of coumarin and [Ru­(bpy)_3_]^2+^ features. Specifically, two luminescence decays could be observed for the three Ru-COUBPY complexes with short (3.7–5.5 ns) and long (116–148 ns) components upon excitation at 405 nm in air-saturated acetonitrile solutions (Figure S27), which can be loosely assigned to the fluorescence of the appended coumarin moiety and the phosphorescence of the ruthenium complex core, respectively.

To gain more insight into the spectroscopic properties of Ru-COUBPY complexes, their ground-state and excited singlet and triplet state properties in ACN were studied using density functional theory (DFT) and time-dependent (TD)-DFT calculations. As shown in Figure 1C, the Ru metal center adopts an octahedral disposition, whereas the coumarin fragment is quasi coplanar to the bpy ligand to which it is attached to a different extent depending on the complex. The values of the O1–C2–C4′-C3′ dihedral angle (see atom numbering in italics in Figure 1B) are 13.8, 30.8, and 31.2° for **SCV42**, **SCV45**, and **SCV49**, respectively, and quantify the relative torsion between the bpy and the coumarin moiety. The higher values for **SCV45** and **SCV49** are coherent with the larger steric hindrance induced by the julolidine ring in the latter two compounds.

The absorption properties in the visible range are rationalized in Tables S1–S3 and Figures S28–S31. The lowest energy bands experimentally centered at 555, 564, and 571 nm for **SCV42**, **SCV45**, and **SCV49**, respectively (Table 1), have been computed at slightly shorter wavelengths (Tables S1–S3) and have been fully characterized as MLCT bands in the first two cases and with a mixed MLCT/IL_cou_ character for **SCV49** as a result of the impact of the CF_3_ substituent in the π → π* absorption in the **COUBPY** moiety. This is clearly revealed by the natural transition orbitals (NTOs)^59,60^ and from inspection of the quantitative wave function analysis^61^ displayed in Figures S28–S31. Indeed, Figure S29 corroborates that the Ru­(II)-coordinated ligands local components (blue color) dominate the lowest-energy S_1_ states in all cases except **SCV49**, in which the increasing contribution of the **COUBPY** intraligand (IL) charge transfer component (red color) is connected with the red-shift of the absorption band observed going from **SCV42** to **SCV49**. The bands experimentally found at 520 and 515 nm for **SCV42** and **SCV45**, missing in **SCV49**, are well reproduced by the singlet–singlet transition to S_3_ computed at 507 and 513 nm, which mixes MLCT and IL_cou_ character. A non-negligible **COUBPY** → Ru­(II) complex charge transfer component is also detected in both transitions, although it is larger for **SCV45** with respect to **SCV42** (Figure S29). Several transitions around the most intense one computed at 469 nm (S_6_) contribute to the broad shoulder experimentally recorded at ∼459 nm for **SCV42** and, analogously, the same band at 460 nm for **SCV45** can be attributed to the S_6_ state computed at 479 nm. A similar absorption feature in this region characterizes the spectrum of **SCV49** in which two transitions of almost equal intensities, computed at 441 and 448 nm, are responsible for the band registered experimentally at 461 nm. In all cases, quantitative wave function analysis and inspection of the NTOs (Figures S28–S31) reveal a dominant MLCT/IL_cou_ nature for the band in this region.

### Dark and Light Stability of Ru-COUBPY Complexes in Biological Media

The stability of the Ru-COUBPY complexes was investigated in a complete cell culture medium (DMEM supplemented with 10% FBS), both in the dark and under visible light irradiation. According to HPLC-MS analysis, all compounds remained completely stable after 24 h of incubation in the dark at 37 °C (Figures S32–S35). Furthermore, both **SCV42** and **SCV49** exhibited remarkable photostability after 1 h of irradiation with green light (505 ± 35 nm, 100 mW·cm^–2^, 360 J cm^–2^), with **SCV42** showing greater resistance to photodegradation than **SCV49** (Figures 2B and S36–S39). Surprisingly, **SCV45** was fully photobleached after the same irradiation time. This suggests that the incorporation of the CF_3_ group at position 4 of the coumarin backbone in **SCV49** enhances the photostability, whereas the substitution of the 7-dialkylamino group with a julolidine moiety has a detrimental effect. Furthermore, **SCV49** experienced less than 35% photobleaching after 1 h of irradiation with red light (620 ± 15 nm; 130 mW cm^–2^, 468 J cm^–2^). Noteworthy, all three Ru-COUBPY complexes were found completely photostable (<3% photodegradation by HPLC-MS analysis) under the typical fluences used in *in vitro* photocytotoxicity experiments (e.g., 9 J cm^–2^ with 540 and 645 nm light; *vide infra*).

### Photochemical Characterization: Experimental and Computational Studies

The ability of Ru-COUBPY complexes to photogenerate various types of ROS was evaluated by using a combination of spectroscopic methods. First, singlet oxygen sensor green (SOSG) was used to confirm that the complexes can sensitize singlet oxygen (^1^O_2_) upon visible light irradiation (Figure 3A). As expected, the increase in the SOSG fluorescence signal observed during light irradiation of the Ru-COUBPY complexes was suppressed in the presence of the selective scavenger sodium azide (Figure S41). Then, singlet oxygen quantum yields (Φ_Δ_) were determined either by direct observation of the ^1^O_2_ phosphorescence (λ_exc_ = 355 or 532 nm) (Figure S42), or by using an indirect method with 1,3-diphenylisobenzofuran (DPBF) as a ^1^O_2_ scavenger, and [Ru­(bpy)_3_]­Cl_2_ or methylene blue (MB) as standards (Figures S43–S44). As shown in Tables 1 and S4, both methods confirmed that **SCV42** is more efficient at sensitizing ^1^O_2_ than **SCV45** or **SCV49**, which reproduced the results with SOSG. The fact that the singlet oxygen quantum yields determined by the indirect method using DPBF are slightly higher than those determined by measuring ^1^O_2_ phosphorescence can be attributed to its ability to react with ROS other than ^1^O_2_ such as superoxide.^62^ The use of two fluorogenic probes, dihydrorhodamine 123 (DHR123) and hydroxyphenyl fluorescein (HPF), confirmed that Ru-COUBPY complexes can also generate superoxide anion radicals (^•^O_2_^–^) and hydroxyl radicals (^•^OH), respectively, upon green light irradiation. This was further validated by the suppression of the probe fluorescence signal in the presence of specific scavengers (i.e., tiron for ^•^O_2_^–^ and terephthalic acid for ^•^OH) (Figures 3B,C and S45–S48). Once again, **SCV42** demonstrated greater efficiency in generating both ^•^O_2_^–^ and ^•^OH radicals compared to those of its julolidine-containing counterparts. To our delight, **SCV49** photogenerates ^1^O_2_, ^•^O_2_^–^ and ^•^OH radicals even under red light irradiation (Figures S41, S46, and S48).

**Figure 3:**
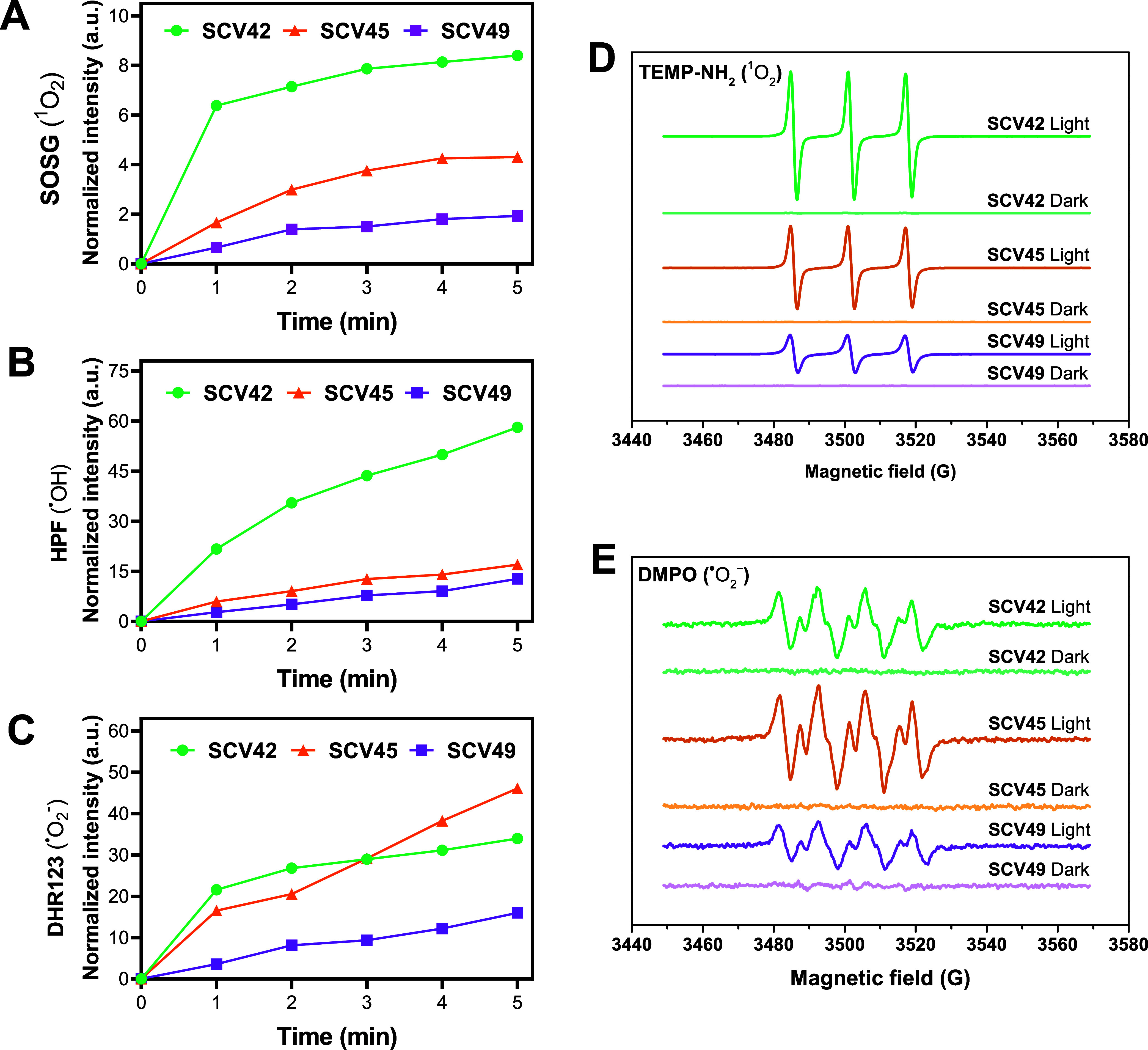
Photogeneration of ROS by Ru-COUBPY complexes studied using specific fluorogenic probes (A–C) and EPR spectroscopy (D–E). Left panels: Increase in fluorescence emission of probes SOSG (5 μM) (A), HPF (5 μM) (B), and DHR123 (10 μM) (C) occurred upon irradiation of Ru-COUBPY complexes (10 μM) in PBS (2% DMSO). Right panels: EPR spectra of Ru-COUBPY complexes trapped by 4-amino-TEMP (D) or DMPO (E) in MeOH, measured in the dark and after green light irradiation.

Further evidence for the light-induced generation of ROS by Ru-COUBPY complexes was provided by electron paramagnetic resonance (EPR). In these experiments, 4-amino-2,2,6,6-tetramethylpiperidine (4-amino-TEMP) and 5,5-dimethyl-1-pyrroline-*N*-oxide (DMPO) were used as spin traps to detect the production of ^1^O_2_ and ^•^O_2_^–^, respectively, upon green light irradiation. The ability of Ru-COUBPYs to photogenerate ^1^O_2_ was confirmed by the observation of the characteristic EPR triplet signal (peak integral ratio 1:1:1, Figure 3D), corresponding to the TEMPO spin adduct. Similarly, the appearance of the diagnostic signal for the DMPO-^•^O_2_^–^ (peak integral ratio 1:1:1:1) adduct confirmed the photogeneration of ^•^O_2_^–^ (Figure 3E). No paramagnetic signal was detected in the dark, demonstrating that the production of ROS is a strictly light-induced process. As shown in Figure 3, there is a good correlation between the intensity of the EPR signals and the relative ability of Ru-COUBPY complexes to photogenerate ^1^O_2_ and ^•^O_2_^–^. Again, the photogeneration of ^1^O_2_ and ^•^O_2_^–^ by **SCV49** upon red light irradiation was also confirmed by EPR (Figure S49).

In order to gain more insight into the ability of Ru-COUBPY complexes to photogenerate Type I ROS, theoretical methods were used to study PDT electron transfer mechanisms. Thermodynamics of the characteristic electron transfer reactions of Type I PDT are compiled in Table 2. Electron transfer in the dark (1) is highly endothermic, in agreement with the absence of ROS formation in the dark (Figures 3E and S49). Direct electron transfer to O_2_ (2) in the triplet state is however favorable for **SCV45** (Δ*E* = −0.17 eV) and **SCV42** (Δ*E* = −0.03 eV). In striking contrast, reaction 2 is significantly endothermic for **SCV49** by Δ*E* = 0.24 eV, due to the weaker electron donor capacities of **SCV49**. Thus, it is reasonable to conclude that **SCV49** is the least efficient system in ^•^O_2_^–^ photoproduction through reaction 2.

**Table 2:** Thermodynamics [Δ*E* = *E*(products) – *E*(reactants)] of the Type I PDT Reactions Computed with the Vertical Electron Affinities (VEAs) and Vertical Ionization Potential (VIP) Values Shown in Tables S5–S7 ^a^

	system		
reaction	42	45	49
1 S1CV2++O32→S2CV3++(O2−•)2	2.00	1.83	1.97
2 S3CV2++O32→S2CV3++(O2−•)2	–0.03	–0.17	0.24
3 S1CV2++S1CV2+→S2CV3++S2CV+	0.50	0.42	0.55
4 S3CV2++S3CV2+→S2CV3++S2CV+	–1.54	–1.58	–1.18
5 S2CV++O32→S1CV2++(O2−•)2	–0.53	–0.59	–0.31
C3OU+[1Ru(bpy)2(dmbpy)]2+→C2OU++[Ru(bpy)2(dmbpy)]2+ 6	0.06	–0.04	0.44
[Ru(bpy)2(dmbpy)]2++O32→[1Ru(bpy)2(dmbpy)]2++(O2−•)2 7	–0.27^b^		
8 [Ru(bpy)2(dmbpy)]2++C2OU+→[Ru(bpy)2(dmbpy)]12++C1OU	–1.81	–1.64	–1.85
9 S3CV2++(O2−•)2→S2CV++O32	–1.50	–1.41	–1.42

a Molecular models of ^2^ **[Ru­(bpy)**_**2**_ **(dmbpy)]** ^**+**^ and coumarin fragments **COU42** ^**+**^, **COU45** ^**+**^, and **COU49** ^**+**^ are shown in Figure S50. Values in eV. The number in the superscript at the left indicates spin, while the superscript at the right indicates the molecular charge. Thus, ^1^SCV^2+^ stands for the Ru-COUBPY complex in the singlet ground state (S_0_) with a total charge of +2, ^2^SCV^3+^ refers to the first doublet state (D_1_) of the oxidized complex with a total charge of +3, and so on.

b This reaction is not specific to any **COU** structure since only the Ru center, the (bpy)_2_ and dmbpy ligands, and molecular oxygen are involved.

The autoionization in reaction 3, in which one PS molecule is in its excited state and the other one is in the ground state, is not thermodynamically favored for any Ru-COUBPY complex (Table 2). Nevertheless, for the autoionization reaction 4, in which the two PS molecules are in the triplet excited state, the net electron transfer is largely exothermic by Δ*E* < −1 eV for the three systems (Table 2). Considering that the electron transfer from the reduced PS to the polarized O_2_ (5) is clearly favorable for all species, reactions 4 and 5 operate in the three compounds and therefore explain the observed Type I PDT photoreactions throughout the series. Nonetheless, **SCV42** and **SCV45** have the extra channel (2), which could account for the larger ^•^O_2_^–^ production observed for these two species with respect to **SCV49** (Figure 3B).

The possibility of intramolecular charge transfer from the **COUBPY** moiety to the Ru^2+^ center complex to generate **COUBPY** ^**+**^ and Ru^+^ is studied in the triplet state, which dominates slow excited-state processes.^63^ Figure S51 reveals that the first triplet state (T_1_) of **SCV42** and **SCV45** is a mixture mostly of a local **COUBPY** excitation (∼0.4, red box) and a **COUBPY**→ Ru­(II) complex component (>0.2, orange box), whereas for **SCV49** the COUPY local excitation is significantly larger (>0.6) and the charge transfer weight is clearly smaller (<0.2). The NTOs for the T_1_ state shown in Figure S52 also confirm this partial **COUBPY** → Ru­(II) complex nature, while the energy differences summarized in Table 2 for process (6), which represents the mentioned electron transfers, are close to zero for **SCV42** and **SCV45**, whereas the formation of a Ru­(I) center is clearly unfavorable for **SCV49**. The thermodynamic analysis thus reveals that this superoxide photoproduction mechanism could be operative for **SCV42** and **SCV45**, even more considering that the electron transfer from the reduced ^2^[Ru­(bpy)_2_(dmbpy)]^+^ fragment to ^3^O_2_ is thermodynamically favorable by Δ*E* = −0.27 eV [process (7), see Table 2]. Nevertheless, this reaction competes with recombination (8), highly favored thermodynamically. **SCV49** is clearly not capable of undergoing intramolecular electron transfer (6), supporting the smaller ^•^O_2_^–^ production observed for this system (Figure 3B). All in all, although possible, the production of superoxide via Ru­(I) through processes (6) and (7) should be considered as a secondary pathway for **SCV42** and **SCV45** and irrelevant for **SCV49**.

Table 2 also shows that ^•^O_2_^–^ can act itself as a reducing agent reacting with the three Ru-COUBPY complexes in the triplet excited states, as illustrated by photoreaction (9). This O_2_ “partial recycling” ^64,65^ may be one rationale for the high phototoxicity induced by these compounds at low oxygen concentrations.

### Cellular Uptake and Subcellular Localization

The cellular uptake of Ru-COUBPY complexes was initially investigated by confocal microscopy in living HeLa cells, taking advantage of the luminescence of the metal complexes for visualization. In all cases, a clear fluorescence signal was detected inside the cells after only 30 min of incubation at a concentration of 10 μM (Figures 4 and S53–S55). While **SCV42** and **SCV45** displayed a distinctive filamentous staining pattern, suggesting preferential accumulation in the mitochondria, **SCV49** exhibited a more diffuse staining pattern with additional localization in intracellular vesicles. A clear indication of the high phototoxicity of Ru-COUBPY complexes was the rapid appearance of membrane blebbing and mitochondrial disintegration, observed after less than 2 min of exposure to the laser light of the confocal microscope.

**Figure 4:**
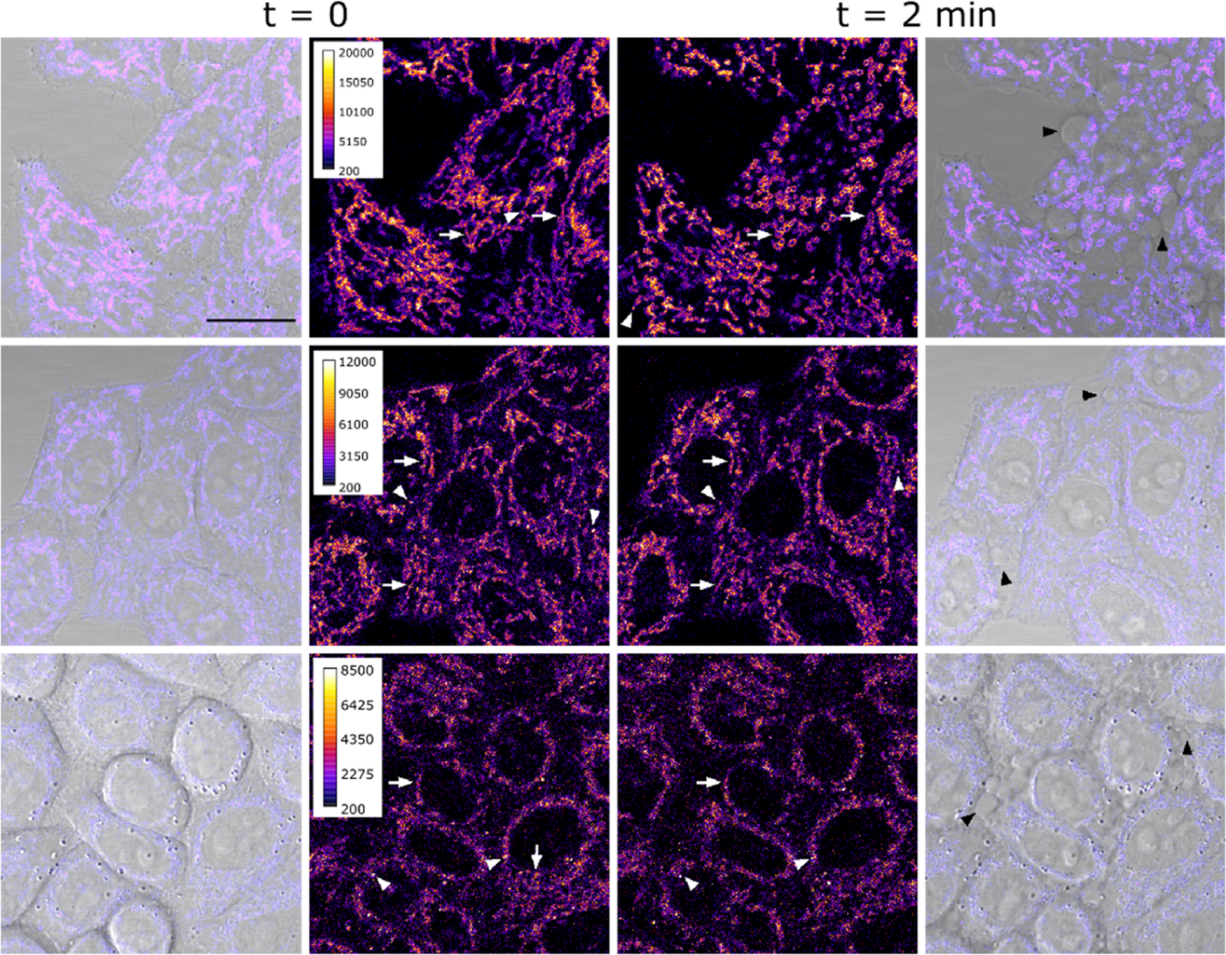
Cellular uptake studies of Ru-COUBPY complexes in living HeLa cells by confocal microscopy. Single confocal planes of HeLa cells incubated with the compounds **SCV42** (top panel), **SCV45** (center panel), and **SCV49** (bottom panel) for 30 min (10 μM) at 37 °C, imaged at *t* = 0 and after 2 min of first observation. Excitation was performed with a 514 nm laser line. White arrows point out mitochondria and white arrowheads point out vesicle staining. Black arrowheads on the right column indicate cell blebbings. Scale bar: 20 μm. LUT for fluorescence images: Fire. Intensity calibration bars are shown in the left central column. Left and right columns: merge of compound and brightfield images.

In order to confirm the subcellular localization of the compounds, a series of colocalization experiments were conducted using the mitochondria, lysosomes, and lipid droplet-specific fluorescent markers Mitoview 650, Lysoview 633, and Lipidspot 610, respectively. As shown in Figure 5, the fluorescence signals of **SCV42** and Mitoview 650 showed a strong overlap, supported by high values in Pearson’s correlation coefficient (PCC = 0.80) and Manders’ colocalization coefficients (M1 = 0.59 corresponding to the colocalization of the compound over the Mitoview 650 channel; M2 = 0.78 corresponding to the colocalization of the Mitoview 650 over the compound channel), indicating predominant accumulation in the mitochondria. A similar behavior was observed for the julolidine-containing Ru-COUBPY complex **SCV45** (Figure S56). In contrast, the correlation between the signals of Mitoview 650 and **SCV49** was weaker, indicating that this Ru-COUBPY complex exhibits slightly reduced specificity for mitochondria (Table S8). This reduced specificity was particularly evident under 405 nm excitation, where **SCV49** displayed a more prominent vesicular distribution pattern. Colocalization studies with Lysoview 633 and LipidSpot 610 (Table S8 and Figure S57) confirmed that this vesicular distribution was predominantly associated with lipid droplets.

**Figure 5:**
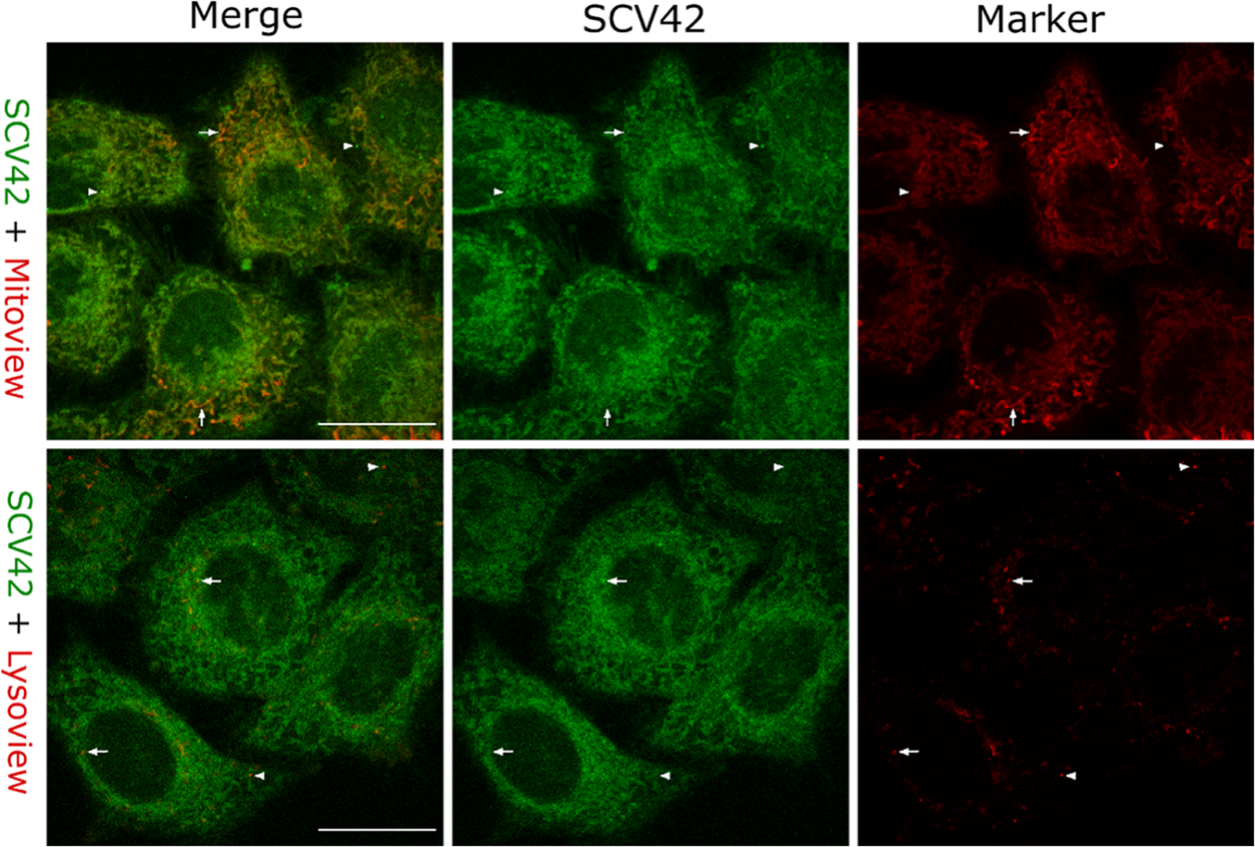
Colocalization studies of **SCV42** in living HeLa cells by confocal microscopy. Single confocal planes of HeLa cells incubated with the **SCV42** compound (10 μM, green) and Mitoview 650 (0.1 μM, red), or Lysoview 633 (1×, red). Left panel: Merge of the two staining. Center panel: **SCV42** signal. Right panel: Mitoview (top) or Lysoview (bottom) signal. White arrows and arrowheads indicate positive and negative colocalization, respectively. Scale bar: 20 μm.

To evaluate the internalization of Ru-COUBPY complexes in the cellular model intended for upcoming *in vitro* photobiological studies, intracellular ruthenium levels were measured by inductively coupled plasma mass spectrometry (ICP-MS) following the incubation of murine colon cancer cells (CT-26) with **SCV42** and **SCV49** (4 h, 5 μM). As shown in Figure S58, the intracellular Ru content for **SCV42** was slightly higher than that of **SCV49**, likely due to subtle differences in lipophilicity between the two compounds, as reflected by the experimental octanol–water distribution coefficients. Indeed, according to the logP_O/W_ values (Table S9 and Figure S59), all Ru-COUBPY complexes were predominantly found in the aqueous phase, with **SCV45** and **SCV49** being less lipophilic than **SCV42**, despite the presence of the julolidine moiety. However, all three Ru-COUBPY complexes were more lipophilic than the reference compound [Ru­(bpy)_3_]­Cl_2_, lacking the coumarin moiety, which indicates that the incorporation of the COUBPY ligand results in an increase of lipophilicity.

### Evaluation of (Photo)­cytotoxicity in 2D Monolayer Cancer Cells

After confirming that Ru-COUBPY complexes can photogenerate both Type I and Type II ROS and readily internalize in living cells, preferentially accumulating in the mitochondria, we next evaluated their cytotoxicity against CT-26 cells under both dark and light conditions. To that end, cells were incubated for 4 h with increasing concentrations of the compounds, and after refreshing the medium, they were either illuminated or kept in the dark. Following a 48-h period after treatment, cell viability was assessed using the resazurin assay, and IC_50_ values were determined from the dose–response curves (Figures 6A and S60). In all experiments, Protoporphyrin IX (PpIX), the active metabolite of clinically used PS 5-ALA, was used as a reference. The phototoxic index (PI), defined as the ratio of dark to light IC_50_ values, was used to quantify the phototherapeutic efficiency of the tested compounds. Given the broad absorption band of Ru-COUBPY complexes in the visible spectrum (Figure 2A), a chromatic screening was conducted to evaluate their phototoxicity across seven wavelengths. Using a well-by-well monochromatic LED device, cells were irradiated with green (540 nm, 40 min), yellow (595 nm, 1 h), red (620 nm, 1 h), deep-red (645 nm, 1 h), far-red (670 nm, 1 h), or NIR (740 nm, 1 h) light, with fluences ranging from 3.4 J/cm^2^ (595 nm) to 13.5 J/cm^2^ (670 nm).

**Figure 6:**
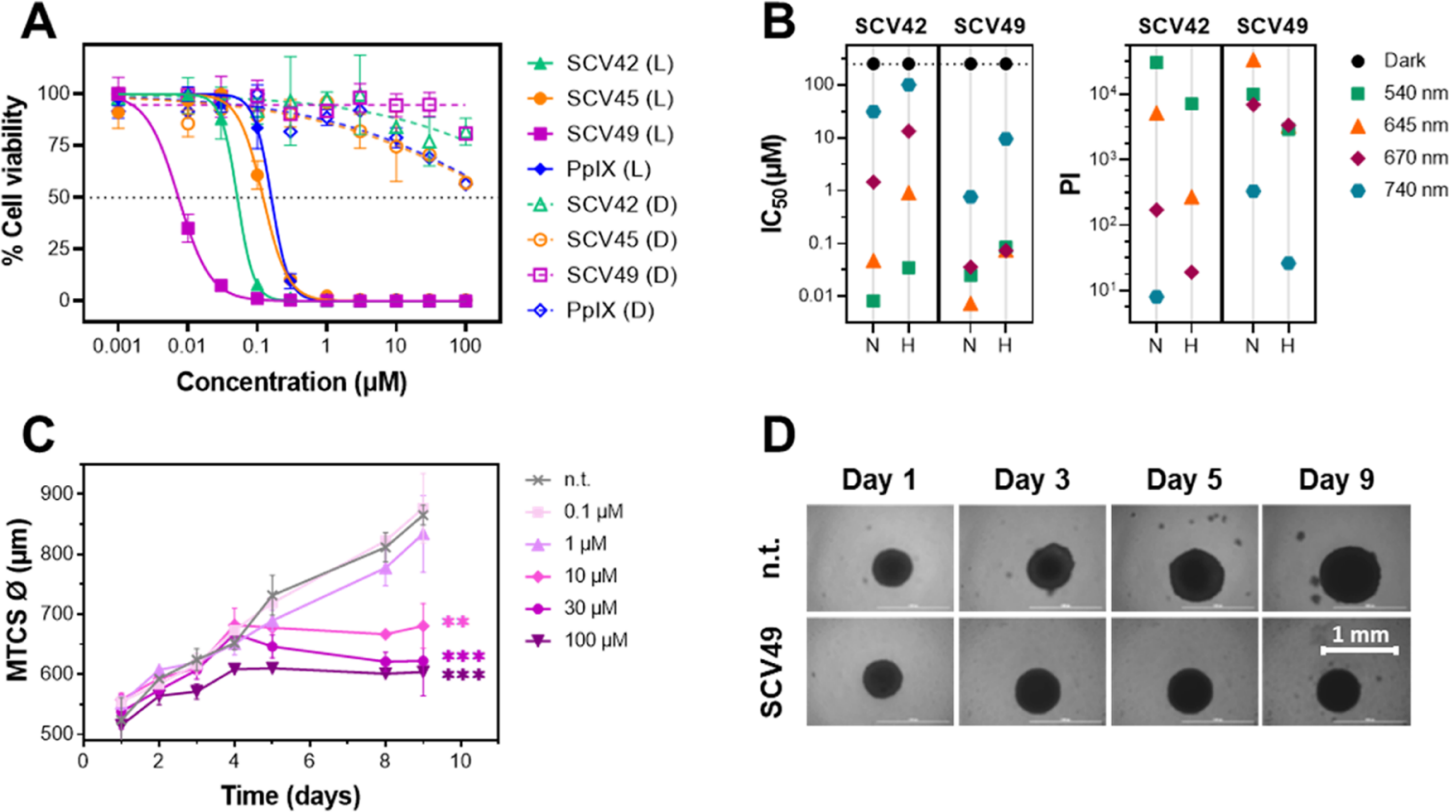
*In vitro* photobiological characterization of Ru-COUBPY complexes in CT-26 2D monolayer cell cultures and 3D multicellular tumor spheroid (MCTS) models. (A) Dose–response curves for **SCV42** (green), **SCV45** (orange), **SCV49** (purple), and PpIX (blue) in CT-26 cells, after 4 h of incubation, upon deep-red light (645 nm, 9.0 J cm^–2^) irradiation (filled symbols) or in the dark (unfilled symbols) under normoxic conditions. (B) Activity plots illustrating the chromatic (photo)­cytotoxicity screening of compounds **SCV42** and **SCV49** in CT-26 cells under green (540 nm, 9.0 J cm^–2^), deep-red (645 nm, 9.0 J cm^–2^), far-red (670 nm, 13.5 J cm^–2^), and NIR (740 nm, 12.6 J cm^–2^) light irradiation, as well as in the dark, under normoxic (21% O_2_) and hypoxic (2% O_2_) conditions. The plots highlight IC_50_ values (left panel) and phototherapeutic indexes (PIs) (right panel). Detailed IC_50_ values with standard deviations and corresponding PI values are provided in Table 4. (C) Evolution of the CT-26 MCTS diameter over a 9-day period. On day 3, MCTSs were treated with varying concentrations of **SCV49** (0.1 to 100 μM) or drug-free cell culture medium (n.t.) for 36 h in the dark, followed by 1 h of deep-red light (645 nm, 9.0 J cm^–2^) irradiation. Data are presented as mean ± SD from three replicates. Statistical significance on day 9 was determined using one-way ANOVA followed by Bonferroni’s multiple comparison test (Asterisks: **p < 0.02, ***p < 0.002). (D) Brightfield micrographs of CT-26 MCTS treated with SCV49 (100 μM) or drug-free cell culture medium (nt) for 36 h, followed by 1 h of deep-red light (645 nm, 9.0 J cm^–2^) irradiation. Scale bar: 1 mm.

As shown in Table 3, Ru-COUBPY complexes displayed no toxicity against CT-26 cells in the dark (IC_50_ > 250 μM), a key feature for an ideal PDT agent, but became highly toxic after irradiation with visible light. **SCV42** exhibited potent nanomolar activity across wavelengths from 540 to 645 nm, with the highest toxicity observed under green light (IC_50_[540 nm] = 8.2 nM), and slightly lower but still excellent results under red and deep-red light (IC_50_[620 nm] = 42 nM; IC_50_[645 nm] = 48 nM). Strikingly, the julolidine-containing complex **SCV45** showed significantly reduced phototoxicity compared to its 7-dialkylamino counterpart **SCV42**, even at wavelengths with higher molar absorptivity (e.g., **SCV45**: IC_50_[595 nm] = 76 nM; **SCV42**: IC_50_[595 nm] = 13 nM), likely due to lower photostability and reduced efficiency in ROS photogeneration. To our delight, complex **SCV49** demonstrated exceptional toxicity under deep-red and far-red light, with IC_50_ values in the very low nanomolar range (e.g., IC_50_[645 nm] = 7.4 nM; IC_50_[670 nm] = 36 nM). Furthermore, **SCV49** retained considerable phototoxic activity even under highly penetrating NIR light (IC_50_[740 nm] = 0.76 μM). This highlights that the bathochromic shift and enhanced photostability resulting from the incorporation of the CF_3_ group at position 4 of the coumarin backbone played a crucial role in boosting the overall PDT activity of **SCV49**. Impressively, all the Ru-COUBPY complexes outperformed the reference PS PpIX at wavelengths below 645 nm, and **SCV49** surpassed PpIX even under far-red and NIR irradiation (e.g., PpIX: IC_50_[670 nm] = 770 nM; **SCV49**: IC_50_[670 nm] = 36 nM). The absence of dark toxicity in these PSs, combined with their exceptional toxicity under light irradiation, resulted in remarkable PI values across the entire visible spectrum. At peak performance, both **SCV42** and **SCV49** exhibited PI values exceeding 30,000 (e.g., **SCV42**: PI­[540 nm] > 30,487; **SCV49**: PI­[645 nm] > 33,783), positioning Ru-COUBPYs among the most phototherapeutically efficient Ru­(II) polypyridyl PSs reported to date.

**Table 3:** (Photo)­cytotoxicity of Ru-COUBPY Complexes and of PpIX towards CT-26 Cancer Cells Expressed as IC_50_ Values (μM) under Normoxia (21% O_2_)­^a^

	**PpIX**		**SCV42**		**SCV45**		**SCV49**	
	IC_50_ (μM)	PI^b^	IC_50_ (μM)	PI^b^	IC_50_ (μM)	PI^b^	IC_50_ (μM)	PI^b^
dark	>100	-	>250	-	>250	-	>250	-
540 nm	0.320 ± 0.09	>312	0.0082 ± 0.0006	>30,487	0.033 ± 0.004	>7575	0.025 ± 0.002	>10,000
595 nm	0.400 ± 0.01	>250	0.013 ± 0.003	>19,230	0.076 ± 0.007	>3289	0.025 ± 0.004	>10,000
620 nm	0.660 ± 0.210	>151	0.042 ± 0.006	>5952	0.118 ± 0.027	>2118	0.017 ± 0.003	>14,705
645 nm	0.170 ± 0.210	>588	0.048 ± 0.003	>5208	0.117 ± 0.002	>2136	0.0074 ± 0.0006	>33,783
670 nm	0.770 ± 0.200	>129	1.460 ± 0.450	>171	1.11 ± 0.32	>225	0.036 ± 0.003	>6944
740 nm	2.100 ± 0.200	>50	31.3 ± 6.1	>8	32.8 ± 4.6	>7.6	0.76 ± 0.06	>329

a Experimental conditions: Cells were incubated for 4 h at 37 °C, followed by either 1 h in the dark or irradiation under the specified light conditions. Cell viability was determined after 44 h using the resazurin assay. Irradiation parameters: 540 nm (3.75 mW cm^–2^, 9.0 J cm^–2^), 595 nm (0.94 mW cm^–2^, 3.4 J cm^–2^), 620 nm (1.88 mW cm^–2^, 6.7 J cm^–2^), 645 nm (2.50 mW cm^–2^, 9.0 J cm^–2^), 670 nm (3.75 mW cm^–2^, 13.5 J cm^–2^), and 740 nm (3.50 mW cm^–2^, 12.6 J cm^–2^).

b Phototherapeutic index (PI) = IC_50_(dark)/IC_50_(light).

As discussed earlier, hypoxia is a major factor contributing to the failure of most conventional anticancer therapies. Given the exceptional phototherapeutic profiles of **SCV42** and **SCV49** under visible light irradiation in normoxic conditions (21% O_2_), we next investigated the cytotoxicity of these compounds against CT-26 cells under challenging hypoxic conditions (2% O_2_), both in the dark and upon irradiation at four representative wavelengths (540, 645, 670, and 740 nm). The impact of oxygen concentration on phototherapeutic efficiency was measured by using the hypoxia index (HI), defined as the ratio of IC_50_ values obtained under hypoxic (2% O_2_) and normoxic (21% O_2_) conditions after light irradiation. Under hypoxia, Ru-COUBPY complexes **SCV42** and **SCV49** remained nontoxic in the dark (IC_50_ > 250 μM) while retaining nanomolar cytotoxicity under visible light irradiation (Table 4 and Figures 6B, S61–S62), although their phototoxic activity was slightly diminished compared to normoxic conditions. Once more, **SCV42** performed best under green light (IC_50_[540 nm] = 35 nM, PI > 7143, HI = 4), while **SCV49** exhibited excellent phototoxicity under green, deep-red and far-red light (e.g., IC_50_[670 nm] = 74 nM, PI > 3378, HI = 2), and maintained micromolar activity under NIR light (IC_50_[740 nm] = 9.56 μM, PI > 26, HI = 12). The exceptional phototoxicity of **SCV42** and **SCV49**, even under hypoxic conditions, likely stems from their ability to simultaneously generate Type I and II ROS in sensitive subcellular structures like mitochondria. These findings further highlight the potential of Ru-COUBPY PSs for treating hypoxic tumors.

**Table 4:** Comparison of the (Photo)­cytotoxicity of SCV42 and SCV49 towards CT-26 Cancer Cells under Normoxia (21% O_2_) and Hypoxia (2% O_2_) Expressed as IC_50_ Values (μM)^a^

	**SCV42**				**SCV49**			
	normoxia (21% O_2_)		hypoxia (2% O_2_)		normoxia (21% O_2_)		hypoxia (2% O_2_)	
	IC_50_ (μM)	PI^b^	IC_50_ (μM)	PI^b^	IC_50_ (μM)	PI^b^	IC_50_ (μM)	PI^b^
dark	>250	-	>250	-	>250	-	>250	-
540 nm	0.0082 ± 0.0006	>30,487	0.035 ± 0.005	>7143	0.025 ± 0.002	>10,000	0.086 ± 0.011	>2907
645 nm	0.048 ± 0.003	>5208	0.920 ± 0.09	>272	0.0074 ± 0.0006	>33,783	0.076 ± 0.008	>3290
670 nm	1.460 ± 0.450	>171	13.24 ± 3.64	>19	0.036 ± 0.003	>6944	0.074 ± 0.005	>3378
740 nm	31.3 ± 6.1	>8	>100	-	0.76 ± 0.06	>329	9.56 ± 2.15	>26

a Experimental conditions and irradiation parameters: see legend to Table 3 and SI.

b Phototherapeutic index (PI) = IC_50_(dark)/IC_50_(light).

### Evaluation of (Photo)­cytotoxicity in 3D Multicellular Tumor Spheroids (MCTS)

In order to complete *in vitro* photobiological studies, we next examined the photoactivity of **SCV49** under deep-red light against 3D multicellular tumor spheroids (MCTS). This culture system is known to better mimic the *in vivo* tumor microenvironment compared to 2D monolayer cultures, closely reproducing key factors that influence PDT efficacy, such as nutrient and drug penetration, resistance to treatment, and hypoxic gradients toward the spheroid’s core.^66^ In this way, CT-26 MCTSs were incubated in the dark for 36 h with increasing concentrations of **SCV49**. After refreshing the medium, the spheroids were exposed to deep-red light (645 nm, 9 J cm^–2^) for 1 h. Following treatment, the shape, integrity, and diameter of the MCTSs were monitored over a 7-day period. Notably, as shown in Figure 6C,D, **SCV49**-treated MCTSs exhibited significant growth inhibition at concentrations above 10 μM upon 645 nm irradiation compared to untreated MCTSs or those treated with lower concentrations of the compound.

Encouraged by the promising *in vitro* PDT activity of Ru-COUBPY complexes, specially under deep-red light irradiation, **SCV49** was selected for *in vivo* safety and efficacy evaluation, which are key steps in the drug development process in the pharmaceutical industry. Our preclinical evaluation included pharmacokinetic studies, with a particular focus on plasma and tissue distribution as well as toxicity studies in healthy mice, alongside the assessment of *in vivo* PDT efficacy in tumor-bearing mice.

### *In Vivo* Evaluation of the Pharmacokinetics and Toxicology of SCV49 in Healthy Mice

The pharmacokinetic (PK) profile of **SCV49** was evaluated in male CD1 mice following a single 5 mg/kg intraperitoneal (IP) dose, which is a common dose used in PK studies because it also provides information about drug tolerability. To that end, blood samples were collected for plasma analysis at specific time points (0.17, 0.5, 1, 2, 4, and 24 h) postadministration. Immediately afterward, the mice were sacrificed, perfused with PBS, and their organs harvested for further analysis. Plasma samples were analyzed by UPLC-MS/MS at various time points to quantify **SCV49** concentrations, enabling the construction of the plasma concentration–time curve and the calculation of PK parameters (Tables S10–S11 and Figure 7A). The analysis showed that **SCV49** was rapidly absorbed, reaching a peak plasma concentration of 5.3 μg/mL within 30 min, with levels stabilizing over the next 2 h (e.g., 4.4 μg/mL at 2 h), indicating significant plasma exposure (AUC). The compound distributed well across tissues (Vd = 1.04 L/kg) and was steadily cleared from the body (Cl = 0.20 L/h·kg), with a moderate half-life of 3.63 h.

**Figure 7:**
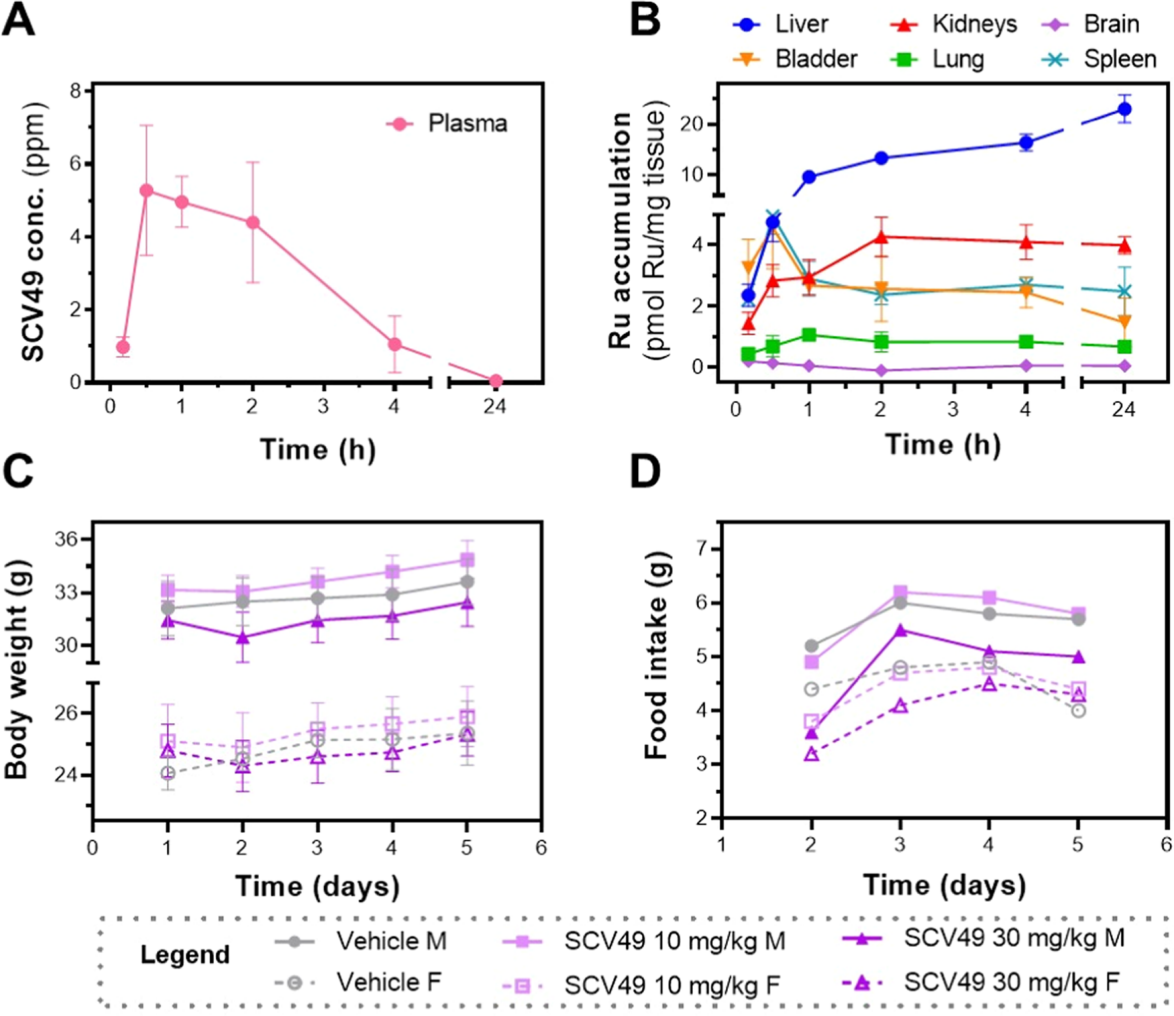
*In vivo* pharmacokinetic (PK) and toxicological evaluation of **SCV49** in healthy CD1 mice. PK includes: (A) Plasma concentration–time curve, and (B) biodistribution profile of ruthenium (Ru) in major organs quantified by ICP-MS at various time points following IP administration of **SCV49** at 5 mg/kg. Data are presented as mean ± SD (*n* = 3 males). Toxicological evaluation includes: (C) Body weight (g) and (D) food intake (g/animal) of mice treated IP with vehicle or **SCV49** (10 or 30 mg/kg) on day 1, with sacrifice on day 5. Data are presented as mean ± SD (*n* = 3 males, *n* = 3 females).

To gain insight into the biodistribution of **SCV49** across various organs and its elimination pathway, the Ru content in key organs (i.e., liver, kidneys, spleen, bladder, lungs, and brain) was quantified using ICP-MS. Figure 7B shows the amount of Ru in each organ at various time points, while Figure S63 provides the percentage of Ru accumulated in each organ relative to the initial dose of **SCV49** administered. Maximum accumulation in the spleen (0.44%), bladder (0.23%), lungs (0.20%), and brain (0.06%) was observed between 10 and 30 min postadministration. However, these values were considerably lower than those observed in the kidneys and liver. Maximum accumulation in the kidneys (1.5%) was observed 4 h postadministration, followed by a decline that paralleled the plasma concentration–time profile, suggesting partial excretion of **SCV49** or its metabolites via renal pathways. The accumulation in the liver steadily increased, reaching a peak (30.9%) at 24 h postadministration, by which time the compound had almost completely cleared from plasma. Accumulation in this organ is not surprising since compounds administered intraperitoneally first pass through the liver before entering the systemic circulation. Although ICP-MS is the most used technique to assess metallodrug biodistribution, it measures only the total metal content and cannot differentiate between the parent compound and its metal-containing metabolites. Since **SCV49** primarily accumulates in the liver, we suspected that its main clearance pathway might involve biliary excretion of the intact drug or its metabolites. To determine whether the detected Ru in the liver was from **SCV49** or a metal-containing metabolite, we quantified the intact **SCV49** in the liver at 1 and 24 h postadministration using UPLC-MS/MS, which required developing a suitable bioanalytical method for liver matrices. As shown in Table S12, the UPLC-MS/MS results mirrored the trend observed in the ICP-MS study, confirming that intact **SCV49** gradually accumulates in the liver. Notably, after 24 h, its levels were more than double those recorded at 1 h, suggesting that **SCV49** is either metabolized very slowly in the liver or primarily excreted unmetabolized via the biliary pathway.

After completing the *in vivo* PK study at an IP dose of 5 mg/kg without any observed toxicity, we designed a 5-day toxicological study in male and female CD1 mice using three increasing doses: low (10 mg/kg), medium (30 mg/kg), and high (100 mg/kg). By systematically increasing the doses, we aimed to identify any dose-dependent toxic effects and establish a safe dosing range for future studies. Unfortunately, the higher dose could not be tested due to solubility problems during administration. Therefore, in this study, mice (3 per group) were intraperitoneally administered either vehicle (Vh) or **SCV49** at 10 or 30 mg/kg (Table S13), and their food consumption and clinical signs were closely monitored. On day 5, blood samples were collected for hematological and plasma biochemical analysis. During necropsy, major organs (thymus, heart, spleen, liver, kidneys, reproductive organs, brain, lung, and bladder) were examined for toxicity markers, harvested, and weighed. Gratifyingly, both doses of **SCV49** were well tolerated by mice of both sexes, with no mortality, clinical signs, or adverse effects observed during the 5-day study. Body weight and food consumption remained within the normal range of variability for the CD1 mice strain (Figures 7C,D and S64). Only one male and one female in the 30 mg/kg group experienced a temporary 4–5% body weight reduction on day 2, which recovered by day 5. Necropsy revealed no organ abnormalities at the 10 mg/kg dose, while all mice in the 30 mg/kg group exhibited slight lilac liver coloration, likely due to drug accumulation in the peritoneum. Again, UPLC-MS/MS analysis indicated that about 15% of the administered **SCV49** dose remained in the liver by day 5. Surprisingly, ICP-MS analysis showed a lower total Ru accumulation of 6%. Regardless, these values, significantly lower than those observed at 24 h postadministration in the PK study, suggest that **SCV49** is gradually cleared from the liver over time. Organ weight and organ weight/body weight ratios in both vehicle and **SCV49** groups were generally within normal ranges (Figure S65). Blood samples were analyzed for changes in red blood cells, hemoglobin, hematocrit, platelets, white blood cells, and related hematological parameters. No significant alterations were observed at either dose of **SCV49** compared to the vehicle group (Figures S66–S67). Furthermore, biochemical analysis of plasma samples focused on hepatic and renal function parameters (Figure S68) revealed no significant differences between **SCV49**-treated and vehicle groups in markers such as albumin (ALB), aspartate aminotransferase, (AST) amylase (AMY), total bilirubin (TBIL) and blood urea nitrogen (BUN), indicating that the temporary accumulation of the PS in the liver and kidneys was not harmful to these organs. Additional metabolic markers, including glucose and cholesterol levels, further confirmed that the overall health of **SCV49**-treated animals was comparable to that of the controls. Thereby, the toxicological study showed that 10 and 30 mg/kg doses of **SCV49** were well tolerated in both male and female CD1 mice over 5 days, with 30 mg/kg identified as the Maximum Tolerated Dose (MTD) in the absence of higher-dose testing.

### *In Vivo* Evaluation of PDT Antitumoral Efficacy of SCV49 in a Mouse Subcutaneous CT-26 Syngeneic Colon Tumor Model

After confirming the *in vivo* safety of **SCV49** in healthy mice, we evaluated its PDT antitumor efficacy using a subcutaneous CT-26 syngeneic colon tumor model. Syngeneic models, which are generated after implanting tumor cells into genetically identical or near-identical mice, are particularly useful in cancer research because they maintain a fully functional immune system. Female BALB/c mice were inoculated with 1.15 × 10^6^ CT-26 cells. Once tumors reached 50–100 mm^3^, the mice were divided into 7 groups (5 animals per group), each receiving a specific treatment as outlined in Figure 8A and Table 5. On days 1 and 3, 40 μL of the vehicle or **SCV49** (3 or 6 mg/kg) were administered intratumorally (IT) over 2 min to ensure even distribution (Figure S69). IT administration offers several advantages over systemic routes in preclinical evaluation but also in the clinic because it allows improved drug concentration in the target tumor tissue and reduces potential side effects due to accumulation in healthy tissues.^67^ Based on the *in vitro* phototoxicity screening results, deep-red light (660 ± 20 nm, 100 mW/cm^2^; Figure S70) was used for irradiation in light-treated groups, with tumors irradiated for 15 (G2, G4) or 20 (G5,G7) min immediately following vehicle or drug administration, which corresponds to light doses of 90 and 120 J cm^–2^, respectively (Table 5). Prior testing demonstrated that this irradiation schedule was well tolerated, causing no skin toxicity or clinical signs for one week post-treatment. Group G5 received additional irradiation on days 2 and 4. Nonirradiated groups (G1, G3, G6) served as controls to assess tumor growth without light exposure, either after vehicle or **SCV49** IT administration at 3 or 6 mg/kg doses.

**Figure 8:**
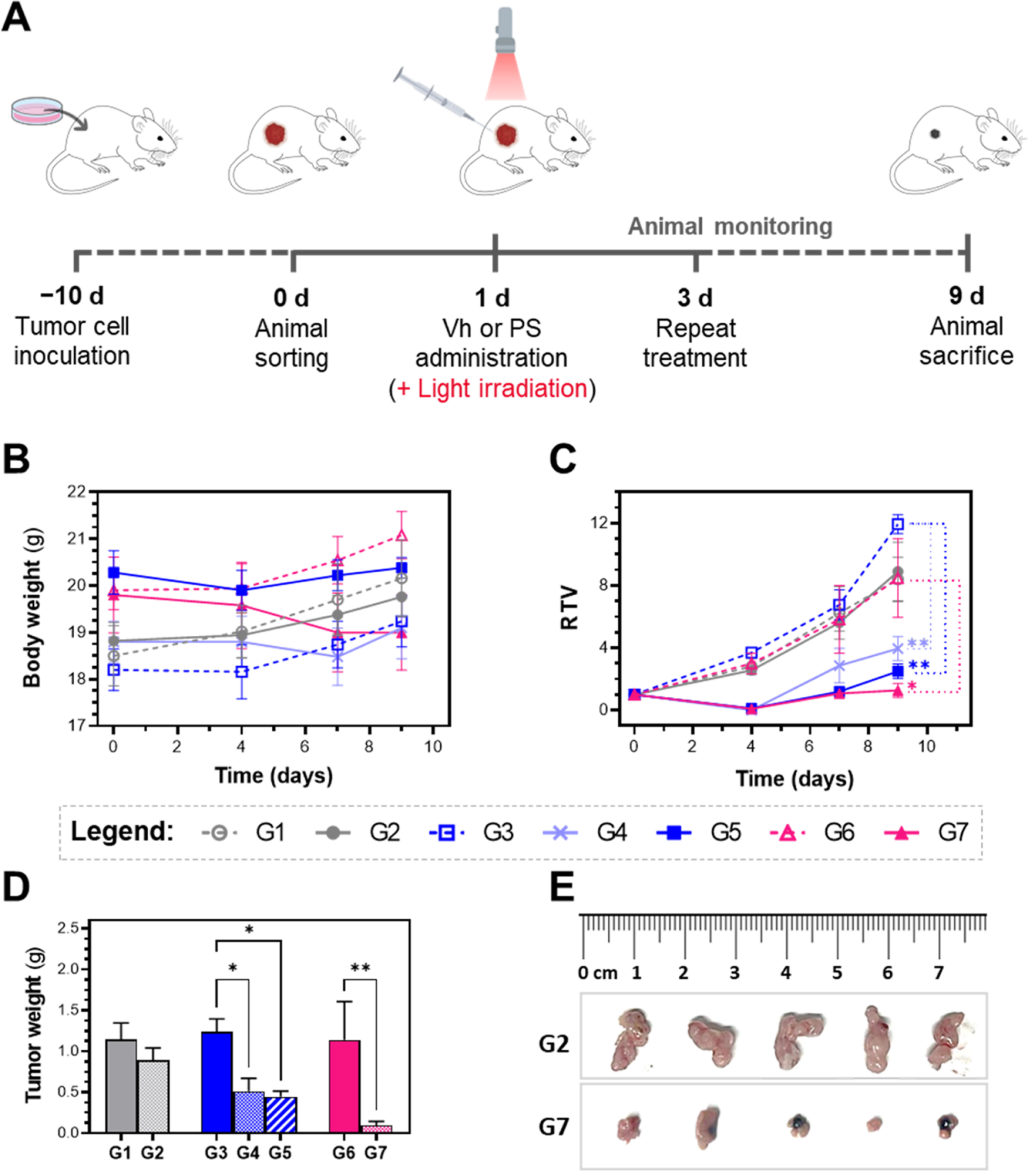
*In vivo* evaluation of **SCV49** PDT antitumor efficacy in a subcutaneous CT-26 tumor model in BALB/c mice. (A) Experimental design: Eight-week-old female BALB/c mice were injected subcutaneously with 1.15 × 10^6^ CT-26 cells on day −10. By day 0, when tumors reached 50–100 mm^3^, mice were randomly divided into 7 groups (*n* = 5/group, Table 4). On days 1 and 3, each group received the assigned treatment and was either exposed to light irradiation or not (660 nm, 15 or 20 min, Table 4, 100 mW/cm^2^). On day 9, animals were sacrificed, and organs and blood samples were collected. (B) Body weight (g) and (C) relative tumor volume (RTV) curves of mice over the 9-day study period. (D) Average tumor weights of mice on the day of sacrifice. Data are presented as mean ± SEM (*n* = 5 females). RTV values on day 9, and average tumor weight data were analyzed using a one-way ANOVA followed by Bonferroni’s multiple comparison test (Asterisks: * *p* < 0.05, ** *p* < 0.001). (E) Representative images of tumors from mice in group G2 (vehicle control, light 2x) and group G7 (SCV49, 6 mg/kg, light 2×) at the study endpoint.

**Table 5:** Experimental Groups of the *In Vivo* PDT Efficacy Study

group	item	conditions	dose/day (mg/kg)	administration schedule	irradiation schedule	light dose/administration (J cm^–2^)
G1	vehicle	dark	-	2 times: day 1 and day 3	-	-
G2	vehicle	light	-	2 times: day 1 and day 3	2 times: 15 min each after 15 min of administration	90
G3	**SCV49**	dark	3	2 times: day 1 and day 3	-	-
G4	**SCV49**	light	3	2 times: day 1 and day 3	2 times: 15 min each after 15 min of administration	90
G5	**SCV49**	light	3	2 times: day 1 and day 3	4 times: 20 min each after 5 min of administration and on days 2 and 4	120
G6	**SCV49**	dark	6	2 times: day 1 and day 3	-	-
G7	**SCV49**	light	6	2 times: day 1 and day 3	2 times: 20 min each after 5 min of administration	120

Following the designated treatment and irradiation regimen, all animals were monitored for clinical signs and body weight changes, and tumor volumes were measured using a caliper. No mortality occurred during the 9-day observation period, and all animals showed normal behavior with no signs of stress or discomfort, consistent with previous PK and toxicological studies of CD1 healthy mice. As shown in Figure 8B, no significant differences in body weight or weight change were observed between **SCV49**-treated groups, whether exposed to light or not, and the vehicle groups. To illustrate the impact of different treatment regimens on tumor volume (mm^3^), the relative tumor volume (RTV) is represented in Figure 8C. Mice treated with **SCV49** (3 or 6 mg/kg) and irradiated with deep-red light showed significantly lower tumor volumes compared to nonirradiated groups, which displayed values similar to those of the vehicle-treated controls. Remarkably, on day 4, tumors in all irradiated **SCV49**-treated groups became unmeasurable (see Figure S71 for details of the images on day 4 for groups G1-G4) regardless of the compound dose and irradiation regime and light dose, indicating highly effective tumor destruction by the Ru-COUBPY complex upon irradiation. This result replicates the potent *in vitro* phototoxicity of **SCV49** against CT-26 cells in an animal model. Comparison between groups G4 and G7 indicated that increasing the dose of the PS from 3 to 6 mg/kg and the light dose (from 90 to 120 J cm^–2^) further enhanced tumor growth inhibition, with group G7 showing complete suppression of tumor regrowth after a slight recurrence on days 5–6. Group G5 (3 mg/kg, 4 consecutive irradiations with 120 J cm^–2^ dose) also achieved similar inhibition but caused skin ulcers in some animals due to the increased number of irradiations. On day 9, the tumor growth inhibition index (TGI, see SI for details), which compares PDT efficacy across treatment groups, confirmed superior efficacy in groups G5 (83%) and G7 (80%) compared with group G4 (69%). The *in vivo* PDT efficacy of **SCV49** was further confirmed by the average tumor weight on day 9 (Figure 8D). Indeed, all **SCV49**-treated, light-irradiated groups showed a significant reduction in tumor weight compared to their nonirradiated counterparts, reaching statistical significance for all relevant comparisons between those groups in which only one variable was modified: G3 vs G4 (3 mg/kg, dark vs light, 2 × 90 J cm^–2^), G3 vs G5 (3 mg/kg, dark vs light, 4 × 120 J cm^–2^) and G6 vs G7 (6 mg/kg, dark vs light, 2 × 120 J cm^–2^). Consistent with tumor volume data, the 6 mg/kg group had significantly lower tumor weight than the 3 mg/kg light-treated groups, regardless of the irradiation schedule and light dose. Images of the tumors from groups 1 to 7 are depicted in Figures 8E and S72.

Finally, to assess the effects of the PDT treatment on animal health, plasma from **SCV49**-treated animals (6 mg/kg, dark and light groups) was subjected to biochemical analysis and compared with vehicle-treated groups. In addition to the parameters analyzed in the toxicological study (ALB, AMY, TBIL, BUN), four new parameters related to liver and kidney function were measured: alkaline phosphatase (ALP), alanine aminotransferase (ALT), creatinine (CRE) and total bile acid (TBA). As shown in Figure S73, no significant differences were found between **SCV49**-treated and vehicle groups, confirming that PDT at 6 mg/kg did not affect hepatic or renal function. Other biochemical markers, including glucose, Na^+^/K^+^ ratio, Ca^2+^, cholesterol, phosphorus, total protein, and globulin, were also within normal limits in both the **SCV49**- and vehicle-treated groups.

## Conclusions

In this work, we described a new family of Ru­(II) polypyridyl complexes incorporating unprecedented coumarin-based COUBPY ligands in the metal coordination sphere exhibiting potent *in vitro* cytotoxicity against cancer cells when irradiated with light within the phototherapeutic window under both normoxic (21% O_2_) and hypoxic (2% O_2_) conditions, while remaining nontoxic in the dark, leading to impressive phototoxic indices (>30,000). Besides singlet oxygen, Ru-COUBPY complexes are able to photogenerate Type I ROS (superoxide and hydroxyl radical), as confirmed by spectroscopic and EPR studies, thereby providing a distinct advantage over current marketed PSs based on the tetrapyrrolic scaffold that primarily rely on Type II PDT mechanism. Thus, the strong phototoxic activity of Ru-COUBPY complexes under hypoxic conditions arises from the coordination of the COUBPY ligands and their ability to photogenerate both Type I and Type II ROS in a key subcellular organelle (mitochondria). Importantly, the results from the *in vivo* safety and efficacy studies in mice underscore the potential of Ru-COUBPY PSs in the PDT treatment of cancer, particularly lead compound **SCV49**. On the one hand, **SCV49** showed a favorable *in vivo* pharmacokinetics profile and excellent toxicological tolerability in healthy mice after IP administration as indicated by several parameters such as animal body weight, food consumption, organ weight, and exhaustive hematological and biochemical analysis. On the other hand, the outstanding *in vitro* phototoxicity of **SCV49** against cancer cells was replicated in an animal model since a potent tumor inhibition in mice bearing subcutaneous CT-26 tumors was observed upon IT administration at doses as low as 3 mg/kg upon deep-red light irradiation (660 nm). Finally, it is worth noting that Ru-COUBPY PSs are highly photostable and aqueous-soluble and can be prepared in high purity from straightforward syntheses, which are highly desirable attributes for further clinical development. Overall, Ru-COUBPY complexes offer new opportunities for the PDT treatment of challenging hypoxic tumors by irradiation with light within the phototherapeutic window.

## Supplementary Material



## Notes

Experimental section; synthesis and chemical, photophysical, and photochemical characterization of the compounds; computational studies; cellular uptake and *in vitro* (photo)­cytotoxicity studies in 2D monolayer cells and in 3D MCTS; *in vivo* PK, toxicological and PDT efficacy studies in mice; and NMR and HRMS spectra (PDF)

## References

[1] Chen Z., Han F., Du Y., Shi H., Zhou W. (2023). Hypoxic microenvironment
in cancer: molecular mechanisms and therapeutic interventions. Signal Transduction Targeted Ther..

[2] Muz B., de la Puente P., Azab F., Azab A. K. (2015). The role of hypoxia
in cancer progression, angiogenesis, metastasis, and resistance to
therapy. Hypoxia.

[3] Chowdhury M., Das P. K. (2024). Hypoxia: Intriguing Feature in Cancer Cell Biology. ChemMedChem.

[4] Yuan X., Ruan W., Bobrow B., Carmeliet P., Eltzschig H. K. (2024). Targeting
hypoxia-inducible factors: therapeutic opportunities
and challenges. Nat. Rev. Drug Discovery.

[5] Wang X., Peng J., Meng C., Feng F. (2024). Fude, Recent advances
for enhanced photodynamic therapy: from new mechanisms to innovative
strategies. Chem, Sci..

[6] Jiang W., Liang M., Lei Q., Li G., Wu S. (2023). The Current
Status of Photodynamic Therapy in Cancer Treatment. Cancers.

[7] Li X., Lovell J. F., Yoon J., Chen X. (2020). Clinical development
and potential of photothermal and photodynamic therapies for cancer. Nat. Rev. Clin. Oncol..

[8] Dolmans D. E. J. G. J., Fukumura D., Jain R. K. (2003). Photodynamic therapy
for cancer. Nat. Rev. Cancer.

[9] Kessel D., Oleinick N. L. (2018). Cell Death Pathways
Associated with Photodynamic Therapy:
An Update. Photochem. Photobiol..

[10] Lu B., Wang L., Tang H., Cao D. (2023). Recent advances in
type I organic photosensitizers for efficient photodynamic therapy
for overcoming tumor hypoxia. J. Mater. Chem.
B.

[11] Zhang C., Hu X., Jin L., Lin L., Lin H., Yang Z., Huang W. (2023). Strategic Design of
Conquering Hypoxia in Tumor for Advanced Photodynamic
Therapy. Adv. Healthcare Mater..

[12] Li G., Wang Q., Liu J., Wu M., Ji H., Qin Y., Zhou X., Wu L. (2021). Innovative strategies for enhanced
tumor photodynamic therapy. J. Mater. Chem.
B.

[13] Pham T. C., Nguyen V.-N., Choi Y., Lee S., Yoon J. (2021). Recent Strategies
to Develop Innovative Photosensitizers for Enhanced Photodynamic Therapy. Chem. Rev..

[14] Zhao X., Liu J., Fan J., Chao H., Peng X. (2021). Recent progress in
photosensitizers for overcoming the challenges of photodynamic therapy:
from molecular design to application. Chem.
Soc. Rev..

[15] Lan M., Zhao S., Liu W., Lee C.-S., Zhang W., Wang P. (2019). Photosensitizers for
Photodynamic Therapy. Adv. Healthcare Mater..

[16] Li X., Kwon N., Guo T., Liu Z., Yoon J. (2018). Innovative
Strategies for Hypoxic-Tumor Photodynamic Therapy. Angew. Chem., Int. Ed..

[17] Singh P. P., Sinha S., Gahtori P., Mishra D. N., Pandey G., Srivastava V. (2024). Recent advancement in photosensitizers for photodynamic
therapy. Dyes Pigm..

[18] Ju M., Yang L., Wang G., Zong F., Shen Y., Wu S., Tang X., Yu D. (2024). A type I and type II chemical biology
toolbox to overcome the hypoxic tumour microenvironment for photodynamic
therapy. Biomater. Sci..

[19] Chen M., Zhu Q., Zhang Z., Chen Q., Yang H. (2024). Recent Advances in
Photosensitizer Materials for Light-Mediated Tumor Therapy. Chem. - Asian J..

[20] An J., Tang S., Hong G., Chen W., Chen M., Song J., Li Z., Peng X., Song F., Zheng W.-H. (2022). An unexpected strategy
to alleviate hypoxia limitation
of photodynamic therapy by biotinylation of photosensitizers. Nat. Commun..

[21] Wang R., Li X., Yoon J. (2021). Organelle-Targeted
Photosensitizers for Precision Photodynamic
Therapy. ACS Appl. Mater. Interfaces.

[22] Desai V. M., Choudhary M., Chowdhury R., Singhvi G. (2024). Photodynamic therapy
induced mitochondrial targeting strategies for cancer treatment: emerging
trends and insights. Mol. Pharmaceutics.

[23] Yaqoob M. D., Xu L., Li C., Leong M. M. L., Xu D. D. (2022). Targeting mitochondria
for cancer photodynamic therapy. Photodiagn.
Photodyn. Ther..

[24] Monro S., Colón K. L., Yin H., Roque J., Konda P., Gujar S., Thummel R. P., Lilge L., Cameron C. G., McFarland S. A. (2019). Transition
Metal Complexes and Photodynamic Therapy
from a Tumor-Centered Approach: Challenges, Opportunities, and Highlights
from the Development of TLD1433. Chem. Rev..

[25] McFarland S. A., Mandel A., Dumoulin-White R., Gasser G. (2020). Metal-based photosensitizers
for photodynamic therapy: the future of multimodal oncology?. Curr. Opin. Chem. Biol..

[26] Gourdon L., Cariou K., Gasser G. (2022). Phototherapeutic anticancer
strategies
with first-row transition metal complexes: a critical review. Chem. Soc. Rev..

[27] Wu Y., Li S., Chen Y., He W., Guo Z. (2022). Recent advances in
noble metal complex based photodynamic therapy. Chem. Sci..

[28] Karges J. (2022). Clinical Development
of Metal Complexes as Photosensitizers for Photodynamic Therapy of
Cancer. Angew. Chem., Int. Ed..

[29] Gandosio A., Purkait K., Gasser G. (2021). Recent approaches towards
the development
of Ru­(II) polypyridyl complexes for anticancer photodynamic therapy. Chimia.

[30] Munegowda M. A., Manalac A., Weersink M., McFarland S. A., Lilge L. (2022). Ru­(II) containing photosensitizers
for photodynamic therapy: A critique
on reporting and an attempt to compare efficacy. Coord. Chem. Rev..

[31] Conti L., Macedi E., Giorgi C., Valtancoli B., Fusi V. (2022). Combination of light and Ru­(II) polypyridyl complexes: Recent advances
in the development of new anticancer drugs. Coord. Chem. Rev..

[32] Cole H. D., Vali A., Roque J. A., Shi G., Kaur G., Hodges R. O., Francés-Monerris A., Alberto M. E., Cameron C. G., McFarland S. A. (2023). Ru­(II)
Phenanthroline-Based Oligothienyl Complexes as Phototherapy Agents. Inorg. Chem..

[33] Zheng M., Lin X., Xiong K., Zhang X., Chen Y., Ji L., Chao H. (2024). A hetero-bimetallic
Ru­(II)-Ir­(III) photosensitizer for effective
cancer photodynamic therapy under hypoxia. Chem.
Commun..

[34] Feng T., Tang Z., Karges J., Shu J., Xiong K., Jin C., Chen Y., Gasser G., Ji L., Chao H. (2024). An iridium­(III)-based
photosensitizer disrupting the mitochondrial respiratory chain induces
ferritinophagy-mediated immunogenic cell death. Chem. Sci..

[35] Liang G., Montesdeoca N., Tang D., Wang B., Xiao H., Karges J., Shang K. (2024). Facile one-pot synthesis of Ir­(III)
Bodipy polymeric gemini nanoparticles for tumor selective NIR photoactivated
anticancer therapy. Biomaterials.

[36] Cole H. D., Roque J. A., Shi G., Lifshits L. M., Ramasamy E., Barrett P. C., Hodges R. O., Cameron C. G., McFarland S. A. (2022). Anticancer
Agent with Inexplicable Potency in Extreme Hypoxia: Characterizing
a Light-Triggered Ruthenium Ubertoxin. J. Am.
Chem. Soc..

[37] Roque J. A., Barrett P. C., Cole H. D., Lifshits L. M., Bradner E., Shi G., von Dohlen D., Kim S., Russo N., Deep G., Cameron C. G., Alberto M. E., McFarland S. A. (2020). Os­(II) Oligothienyl Complexes as a Hypoxia-Active Photosensitizer
Class for Photodynamic Therapy. Inorg. Chem..

[38] Steinke S. J., Dunbar M. N., Suarez M. A. A., Turro C. (2024). Ru­(II) Complexes with
Absorption in the Photodynamic Therapy Window: ^1^O_2_ Sensitization, DNA Binding, and Plasmid DNA Photocleavage. Inorg. Chem..

[39] Kasparkova J., Hernández-García A., Kostrhunova H., Goicuría M., Novohradsky V., Bautista D., Markova L., Santana M. D., Brabec V., Ruiz J. (2024). Novel 2-(5-Arylthiophen-2-yl)-benzoazole
Cyclometalated Iridium­(III) dppz Complexes Exhibit Selective Phototoxicity
in Cancer Cells by Lysosomal Damage and Oncosis. J. Med. Chem..

[40] Zamora A., Vigueras G., Rodríguez V., Santana M. D., Ruiz J. (2018). Cyclometalated
iridium­(III) luminescent complexes in therapy and phototherapy. Coord. Chem. Rev..

[41] Havrylyuk D., Hachey A. C., Fenton A., Heidary D. K., Glazer E. C. (2022). Ru­(II)
photocages enable precise control over enzyme activity with red light. Nat. Commun..

[42] Bonnet S. (2023). Ruthenium-Based
Photoactivated Chemotherapy. J. Am. Chem. Soc..

[43] Zhang L., Wang P., Zhou X.-Q., Bretin L., Zeng X., Husiev Y., Polanco E. A., Zhao G., Wijaya L. S., Biver T., Le Dévédec S. E., Sun W., Bonnet S. (2023). Cyclic Ruthenium-Peptide Conjugates as Integrin-Targeting
Phototherapeutic Prodrugs for the Treatment of Brain Tumors. J. Am. Chem. Soc..

[44] He G., He M., Wang R., Li X., Hu H., Wang D., Wang Z., Lu Y., Xu N., Du J., Fan J., Peng X., Sun W. (2023). A Near-Infrared Light-Activated Photocage
Based on a Ruthenium Complex for Cancer Phototherapy. Angew. Chem., Int. Ed..

[45] Zhang C., Qinc W.-J., Bai X.-F., Zhanga X.-Z. (2020). Nanomaterials to
relieve tumor hypoxia for enhanced photodynamic therapy. Nano Today.

[46] Huang L., Zhao S., Wu J., Yu L., Singh N., Yang K., Lan M., Wang P., Kim J. S. (2021). Photodynamic
therapy for hypoxic tumors: Advances and perspectives. Coord. Chem. Rev..

[47] Wan Y., Fu L.-H., Li C., Lin J., Huang P. (2021). Conquering
the Hypoxia Limitation for Photodynamic Therapy. Adv. Mater..

[48] Gandioso A., Bresolí-Obach R., Nin-Hill A., Bosch M., Palau M., Galindo A., Contreras S., Rovira A., Rovira C., Nonell S., Marchán V. (2018). Redesigning the Coumarin Scaffold
into Small Bright Fluorophores with Far-Red to Near-Infrared Emission
and Large Stokes Shifts Useful for Cell Imaging. J. Org. Chem..

[49] Rovira A., Pujals M., Gandioso A., López-Corrales M., Bosch M., Marchán V. (2020). Modulating photostability and mitochondria
selectivity in far-red/NIR emitting coumarin.fluorophores through
replacement of pyridinium by pyrimidinium. J.
Org. Chem..

[50] Izquierdo-García E., Rovira A., Forcadell J., Bosch M., Marchán V. (2023). Exploring
Structural–Photophysical Property Relationships in Mitochondria-Targeted
Deep-Red/NIR-Emitting Coumarins. Int. J. Mol.
Sci..

[51] Rovira A., Gandioso A., Goñalons M., Galindo A., Massaguer A., Bosch M., Marchán V. (2019). Solid-phase
approaches for labeling
targeting peptides with far-red emitting coumarin fluorophores. J. Org. Chem..

[52] Izquierdo E., López-Corrales M., Abad-Montero D., Rovira A., Fabriàs G., Bosch M., Abad J.-L., Marchán V. (2022). Fluorescently-labelled ceramides and 1-deoxyceramides:
Synthesis, characterization and cellular distribution studies. J. Org. Chem..

[53] Ortega-Forte E., Rovira A., Gandioso A., Bonelli J., Bosch M., Ruiz J., Marchán V. (2021). COUPY Coumarins as Novel Mitochondria-Targeted
Photodynamic Therapy Anticancer Agents. J. Med.
Chem..

[54] Bonelli J., Ortega-Forte E., Rovira A., Bosch M., Torres O., Cuscó C., Rocas J., Ruiz J., Marchán V. (2022). Improving
Photodynamic Therapy Anticancer Activity of a Mitochondria-Targeted
Coumarin Photosensitizer Using a Polyurethane–Polyurea Hybrid
Nanocarrier. Biomacromolecules.

[55] Novohradsky V., Rovira A., Hally C., Galindo A., Vigueras G., Gandioso A., Svitelova M., Bresolí-Obach R., Kostrhunova H., Markova L., Kasparkova J., Nonell S., Ruiz J., Brabec V., Marchán V. (2019). Towards novel
photodynamic anticancer agents generating superoxide anion radicals:
A cyclometalated Ir­(III) complex conjugated to a far-red emitting
coumarin. Angew. Chem., Int. Ed..

[56] Novohradsky V., Markova L., Kostrhunova H., Kasparkova J., Ruiz J., Marchán V., Brabec V. (2021). A cyclometallated IrIII
complex conjugated to a coumarin derivative is a potent photodynamic
agent against prostate differentiated and tumorigenic cancer stem
cells. Chem. - Eur. J..

[57] Rovira A., Ortega-Forte E., Hally C., Jordà-Redondo M., Abad-Montero D., Vigueras G., Martínez J. I., Bosch M., Nonell S., Ruiz J., Marchán V. (2023). Exploring
Structure–Activity Relationships in Photodynamic Therapy Anticancer
Agents Based on Ir­(III)-COUPY Conjugates. J.
Med. Chem..

[58] Ortega-Forte E., Rovira A., López-Corrales M., Hernández-García A., Ballester F. J., Izquierdo-García E., Jordà-Redondo M., Bosch M., Nonell S., Santana M. D., Ruiz J., Marchán V., Gasser G. (2023). A near-infrared light-activatable
Ru­(II)-coumarin photosensitizer active under hypoxic conditions. Chem. Sci..

[59] Skripnikov, L. Chemissian 4.67 2020 www.chemissian.com.

[60] Martin R. L. (2003). Natural
transition orbitals. J. Chem. Phys..

[61] Plasser F. (2020). TheoDORE:
A toolbox for a detailed and automated analysis of electronic excited
state computations. J. Chem. Phys..

[62] Entradas T., Waldron S., Volk M. (2020). The detection sensitivity of commonly
used singlet oxygen probes in aqueous environments. J. Photochem. Photobiol., B.

[63] Roque J. A., Cole H. D., Barrett P. C., Lifshits L. M., Hodges R. O., Kim S., Deep G., Francés-Monerris A., Alberto M. E., Cameron C. G., McFarland S. A. (2022). Intraligand
Excited States Turn a Ruthenium Oligothiophene Complex into a Light-Triggered
Ubertoxin with Anticancer Effects in Extreme Hypoxia. J. Am. Chem. Soc..

[64] Teng K.-X., Chen W.-K., Niu L.-Y., Fang W.-H., Cui G., Yang Q.-Z. (2021). BODIPY-Based Photodynamic Agents for Exclusively Generating
Superoxide Radical over Singlet Oxygen. Angew.
Chem., Int. Ed..

[65] Li M., Xia J., Tian R., Wang J., Fan J., Du J., Long S., Song X., Foley J. W., Peng X. (2018). Near-Infrared
Light-Initiated Molecular Superoxide Radical Generator: Rejuvenating
Photodynamic Therapy against Hypoxic Tumors. J. Am. Chem. Soc..

[66] Friedrich J., Seidel C., Ebner R., Kunz-Schughart L. A. (2009). Spheroid-based
drug screen: considerations and practical approach. Nat. Protoc..

[67] Ma C.-H., Yang J., Mueller J. L., Huang H.-C. (2021). Intratumoral Photosensitizer
Delivery and Photodynamic Therapy. Nano LIFE.

