# Supplementary material for: Ruthenium(II) Polypyridyl Complexes Containing COUBPY Ligands as Potent Photosensitizers for the Efficient Phototherapy of Hypoxic Tumors
Source: J Am Chem Soc. 2025 Feb 15;147(9):7360–76. doi: 10.1021/jacs.4c15036 (PMC12164272; doi:10.1021/jacs.4c15036)
Supplement: Supplementary file 1 [file ja4c15036_si_001.pdf]

SUPPORTING INFORMATION

Ruthenium(II) polypyridyl complexes containing COUBPY ligands as potent photosensitizers for the efficient phototherapy of hypoxic tumors

Diego Abad-Montero,[1,#] Albert Gandioso,[2,#] Eduardo Izquierdo-García,[1,2,#] Sergi Chumillas,[1,#] Anna Rovira,[1] Manel Bosch,[3] Mireia Jordà-Redondo,[4] Davor Castaño,[1] Joaquín Bonelli,[1] Valentin V. Novikov,[5] Alba Deyà,[6] José Luis Hernández,[6] Jorge Galino,[6] Marta E. Alberto,[7] Antonio Francés-Monerris,[8] Santi Nonell,[4] Gilles Gasser,[2,*] Vicente Marchán[1,*]

[1] Departament de Química Inorgànica i Orgànica, Secció de Química Orgànica, Universitat de Barcelona (UB), and Institut de Biomedicina de la Universitat de Barcelona (IBUB), Martí i Franquès 1-11, E-08028 Barcelona, Spain. Email: vmarchan@ub.edu

[2] Chimie ParisTech, PSL University, CNRS, Institute of Chemistry for Life and Health Sciences, Laboratory for Inorganic Chemical Biology, F-75005 Paris, France. Email: gilles.gasser@chimieparistech.psl.eu

[3] Unitat de Microscòpia Òptica Avançada, Centres Científics i Tecnològics, Universitat de Barcelona, Av. Diagonal 643, E- 08028 Barcelona, Spain.

[4] Institut Químic de Sarrià, Universitat Ramon Llull, Vía Augusta 390, E-08017 Barcelona, Spain.

[5] Departament de Química Inorgànica i Orgànica, Secció de Química Inorgànica, Universitat de Barcelona (UB), and Institute of Nanoscience and Nanotechnology of the University of Barcelona (IN2UB), Martí i Franquès 1-11, E-08028 Barcelona, Spain.

[6] Health and Biomedicine Department, Leitat Technological Center, Carrer de la Innovació 2, E-08225 Terrassa, Spain.

[7] Dipartimento di Chimica e Tecnologie Chimiche, Università della Calabria, Arcavacata di Rende I-87036, Italy

[8] Institut de Ciència Molecular, Universitat de València, P.O. Box 22085, València 46071, Spain

[#] These authors contributed equally to this work.

Table of contents

1.- Synthesis and characterization of the compounds

1.1. Materials and methods S4

1.2. Synthesis of COUBPY ligands S5

1.3. Synthesis of Ru-COUBPY complexes S8

1.4. HPLC analysis of the compounds S10

2.- 1D ^{1}H, ^{19}F and ^{13}C NMR spectra and HR ESI-MS of the compounds S11

3.- NOESY spectra of COUBPY ligands and Ru-COUBPY complexes S28

4.- Photophysical characterization: experimental and computational studies

4.1. Spectroscopic studies S34

4.2. Computational studies S36

5.- Dark and light stability studies in cell culture medium

5.1. Dark stability S43

5.2. Photostability S47

6.- Photochemical characterization: experimental and computational studies

6.1. Evaluation of singlet oxygen generation using SOSG S52

6.2. Quantification of singlet oxygen generation

6.2.1. Direct method S54

6.2.2. Indirect method S56

6.3. Evaluation of superoxide anion radical generation using DHR123 S59

6.4. Evaluation of hydroxyl radical generation using HPF S61

6.5. Electron paramagnetic resonance (EPR) studies S63

6.6. Computational studies S64

7. Cellular uptake studies

7.1. Cellular uptake by confocal microscopy S69

7.2. Cellular accumulation by ICP-MS S76

7.3. Lipophilicity determination S77

8. *In vitro* (photo)cytotoxicity determination of Ru-COUBPY PSs S79

8.1. Cell culture

8.2. (Photo)cytotoxicity evaluation in 2D monolayer cells under normoxia (21% O_2)

8.3. (Photo)cytotoxicity evaluation in 2D monolayer cells under hypoxia (2% O_2)

9. (Photo)cytotoxicity evaluation of **SCV49** on 3D multicellular tumor spheroids S84

10. *In vivo* pharmacokinetic (PK) study of **SCV49** in CD1 mice
10.1. Environment and husbandry S85
10.2. Animal Welfare S85
10.3. Administration S85
10.4. Sampling S85
11. *In vivo* toxicological study of **SCV49** in CD1 mice
11.1. Environment and husbandry S89
11.2. Animal Welfare S89
11.3. Formulation and compound administration S89
11.4. Experimental procedure S90
11.5. Data processing and statistical analysis S90
12. *In vivo* PDT efficacy study of **SCV49** in BALB/c mice bearing subcutaneous CT-26 syngeneic colon tumors
12.1. Ethical animal procedures and animal housing conditions S97
12.2. Tumor cell line S97
12.3. Formulation of the compound S97
12.4. Light tolerability test S97
12.5. *In vivo* PDT efficacy study S98
13. Cartesian Coordinates of the Ru-COUBPY complexes S104
14. References S108

1. Synthesis and characterization of the compounds

1.1. Materials and methods

Unless otherwise stated, common chemicals and solvents (HPLC grade or reagent grade quality) were purchased from commercial sources and used without further purification. A hot plate magnetic stirrer, together with an aluminum reaction block of the appropriate size, was used as the heating source in all reactions requiring heat. Aluminum plates coated with a 0.2 mm thick layer of silica gel 60 F_{254} were used for thin-layer chromatography analyses (TLC), whereas flash column chromatography purification was carried out using silica gel 60 (230-400 mesh). NMR spectra were recorded at 25 ^{o}C in 400 or 500 MHz spectrometers using the deuterated solvent as an internal deuterium lock. The residual protic signal of $CHCl_3$, CH_3OH and DMSO was used as a reference in ^{1}H and ^{13}C NMR spectra recorded in $CDCl_3$, CD_3OD and DMSO-d_6, respectively. Chemical shifts are reported in part per million (ppm) in the δ scale, coupling constants in Hz and multiplicity as follows: s (singlet), d (doublet), t (triplet), q (quartet), qt (quintuplet), m (multiplet), dd (doublet of doublets), dt (doublet of triplets), td (triplet of doublets), br (broad signal), etc. The proton signals of the *E* and *Z* rotamers were identified by simple inspection of the ^{1}H spectrum and the rotamer ratio was calculated by peak integration. 2D-NOESY spectra were acquired in $CDCl_3$ or in CD_3OD with mixing times of 500 ms. Electrospray ionization mass spectra (ESI-MS) were recorded on an instrument equipped with single quadrupole detector coupled to an HPLC and high-resolution (HR) ESI-MS on an LC/MS-TOF instrument. Reversed-phase high-performance liquid chromatography (HPLC) analyses were carried out on a Jupiter Proteo C12 column (150 × 4.6 mm, 90 Å, 4 µm, flow rate: 1 mL/min) using linear gradients of 0.1% formic acid in Milli-Q H_2O (A) and 0.1% formic acid in ACN (B). The HPLC column was maintained at 25 °C. All final compounds were >95% pure by this method.

1.2. Synthesis of COUBPY ligands

4-Methyl-4'-((trimethylsilyl)methyl)-2,2'-bipyridine (4)[1]

A solution of 4,4'-dimethyl-2,2'-bipyridine (2 g, 10.86 mmol) in anhydrous THF (80 mL) was added dropwise via cannula to a cold (-78 ºC) solution of LDA in THF (12 mL, 11.95 mmol) under an Ar atmosphere. The resulting maroon mixture was stirred for 1 h at -78 ºC. Then, trimethylsilyl chloride (2 mL, 11.95 mmol) was added to the crude, which turned to blue and, exactly 10 seconds later, 10 mL of absolute ethanol were added carefully, which caused the solution to become yellow. The crude was transferred to a separatory funnel containing a saturated solution of $NaHCO_3$ (200 mL) and extracted with DCM (3x150 mL). The combined organic phases were washed with brine (150 mL), dried over anhydrous Na_2SO_4, filtered and evaporated to dryness to obtain 2.12 g of a yellow solid (yield: 76%) which was used without further purification. ^{1}H-NMR ($CDCl_3$, 400 MHz): δ (ppm) 8.49 (d, *J* = 5.0 Hz, 1H), 8.43 (d, *J* = 5.0 Hz, 1H), 8.18 (s, 1H), 8.01 (s, 1H), 7.10-7.05 (m, 1H), 6.91 (dd, *J* = 5.0, 1.7 Hz, 1H), 2.39 (s, 3H), 2.17 (s, 2H), 0.00 (s, 9H). ^{13}C NMR (101 MHz, $CDCl_3$): δ (ppm) 156.4, 155.9, 151.47, 149.1, 148.8, 148.1, 124.6, 123.6, 122.1, 121.0, 27.7, 21.3, -1.7.

4-(Chloromethyl)-4'-methyl-2,2'-bipyridine (5)[2]

4-Methyl-4'-((trimethylsilyl)methyl)-2,2'-bipyridine (2.12 g, 7.80 mmol), hexachloroethane (7.39 g, 31.2 mmol) and cesium fluoride (4.76 g, 31.2 mmol) were dissolved in anhydrous ACN (120 mL) under an Ar atmosphere and the resulting solution was stirred at 60 ºC for 3.5 h. The reaction mixture was partitioned between 50 mL of H_2O and 50 mL of AcOEt and transferred to a separatory funnel. The aqueous phase was extracted with ethyl acetate (3x50 mL) and the combined organic phases were washed with brine (50 mL), dried over anhydrous Na_2SO_4, filtered and evaporated to dryness. The product was purified by column chromatography (silica gel, 0-10% MeOH in DCM) to obtain 1.01 g of a yellow solid (yield: 57%). TLC: Rf (1:9 MeOH/DCM) 0.6. ^{1}H NMR (400 MHz, $CDCl_3$): δ (ppm) 8.68 (d, *J* = 5.1 Hz, 1H), 8.54 (d, *J* = 5.0 Hz, 1H), 8.43 – 8.39 (m, 1H), 8.26 – 8.21 (m, 1H), 7.38 – 7.34 (m, 1H), 7.18 – 7.12 (m, 1H), 4.63 (s, 2H), 2.44 (s, 3H). ^{13}C NMR (101 MHz, $CDCl_3$): δ 157.0, 155.5, 149.7, 149.2, 148.4, 147.1, 125.1, 123.0, 122.2, 120.6, 44.5, 21.3. LR-ESI MS (ESI): *m/z* 218.8 calc. for $[C_{12}H_{11}N_2Cl+H]^+$: 218.1.

2-(4'-Methyl-[2,2'-bipyridin]-4-yl) acetonitrile (6)

4-(Chloromethyl)-4'-methyl-2,2'-bipyridine (1 g, 4.57 mmol), 18-crown-6 (26.8 mg, 0.09 mmol) and KCN (3.39 g, 36.5 mmol) were dissolved in 100 mL of ACN and stirred overnight

at room temperature. HPLC-MS analysis of the crude revealed that the product was barely formed. Then, 214 mg of 18-crown-6 (0.81 mmol) were added and the solution was stirred overnight at 50 ºC. After confirmation by HPLC-MS that the reaction had finished, the reaction mixture was evaporated to dryness, re-dissolved in deionized water (100 mL) and extracted with DCM (3x100 mL). The combined organic phases were dried over anhydrous Na_2SO_4, filtered and evaporated to dryness. After purification by column chromatography (silica gel, 50-100% AcOEt in hexanes), 615 mg of a white solid were obtained (yield: 64%). White solid. TLC: Rf (AcOEt 100%) 0.7. ^{1}H NMR (400 MHz, $CDCl_3$): δ (ppm) 8.70 (d, J = 5.0 Hz, 1H), 8.53 (d, J = 5.0 Hz, 1H), 8.40 – 8.37 (m, 1H), 8.27 – 8.23 (m, 1H), 7.37 – 7.33 (m, 1H), 7.20 – 7.15 (m, 1H), 3.84 (s, 2H), 2.45 (s, 3H). ^{13}C NMR (101 MHz, $CDCl_3$): δ (ppm) 157.4, 155.1, 150.1, 149.2, 148.5, 140.1, 125.3, 122.7, 122.3, 120.6, 116.5, 23.5, 21.3. HR-ESI MS (ESI): *m/z* 210.1026 calc. for $[C_{13}H_{11}N_3+H]^+$: 210.1025.

COUBPY ligand 1

To a solution of sodium hydride (568 mg of a 60% dispersion in mineral oil, 14.15 mmol) and compound **6** (712 mg, 3.40 mmol) in anhydrous acetonitrile (300 mL), 7-(*N*,*N*-diethylamino)-4-methyl-2-thiocoumarin **7**[3] (703 mg, 5.66 mmol) was added. The orange solution was stirred for 3 h. Then, silver nitrate (962 mg, 4.98 mmol) was added and the reaction mixture was stirred for 2 h at room temperature. The dark-maroon solution was analysed by HPLC-MS to confirm the formation of the desired product in the crude, which was evaporated under reduced pressure. The product was isolated by silica column chromatography starting with hexanes and increasing the eluent polarity with DCM first (0-100%) and then with MeOH (0-10%). 451 mg of an orange/red solid were obtained (yield: 37%). TLC: Rf (1:9 MeOH/DCM) 0.5. HPLC (30-100 % B, 15 min): Rt (min) = 9.03. ^{1}H NMR (400 MHz, $CDCl_3$): δ (ppm) (*E* rotamer) 9.18 (dd, J = 2.0, 0.8 Hz, 1H), 8.62 (dd, J = 5.3, 0.8 Hz, 1H), 8.51 (d, J = 4.9 Hz, 1H), 8.30 – 8.25 (m, 1H), 7.60 (dd, J = 5.4, 2.0 Hz, 1H), 7.32 (d, J = 8.9 Hz, 1H), 7.12 (dd, J = 5.0, 1.7 Hz, 1H), 6.75 (d, J = 1.2 Hz, 1H), 6.70 (d, J = 2.5 Hz, 1H), 6.58 (dd, J = 8.9, 2.5 Hz, 1H), 3.46 (q, J = 7.1 Hz, 4H), 2.45 (s, 3H), 2.34 (s, 3H), 1.25 (t, J = 7.1 Hz, 6H).^{13}C NMR (101 MHz, $CDCl_3$): δ (ppm) (*E* rotamer) 163.8, 156.5, 156.3, 154.6, 150.8, 149.3, 149.0, 148.0, 144.2, 142.1, 125.4, 124.7, 122.1, 120.7, 120.1, 118.4, 112.4, 110.1, 109.2, 97.6, 81.7, 44.9, 21.3, 18.5, 12.7. HR-ESI MS (ESI): *m/z* 423.2181 calc. for $[C_{27}H_{26}N_4O+H]^+$: 423.2179.

COUBPY ligand 2

To a solution of NaH (38 mg of a 60% dispersion in mineral oil, 1.58 mmol) and compound **6** (46 mg, 0.22 mmol) in anhydrous ACN (12 mL), thiocoumarin **8** (50 mg, 0.18 mmol) was added. The maroon solution was stirred at 50ºC for 3 h. Then, silver nitrate (71 mg, 0.41 mmol) was added and the crude was stirred for 2 h at room temperature. The product was isolated by silica column chromatography starting with hexanes and increasing the eluent polarity with DCM first (0-100%) and then with MeOH (0-10%). 20 mg of orange/brown solid were obtained (yield: 20%). TLC: Rf (5% MeOH/DCM) 0.5. HPLC (30-100 % B, 15 min): Rt (min) = 8.78.

^{1}H NMR (400 MHz, $CDCl_3$): δ (ppm) (*E* rotamer) 9.01 (d, *J* = 1.5 Hz, 1H), 8.61 (d, *J* = 5.5 Hz, 1H), 8.50 (d, *J* = 4.9 Hz, 1H), 8.25 (s, 1H), 7.57 (dd, *J* = 5.3, 2.0 Hz, 1H), 7.12 (d, *J* = 5.0 Hz, 1H), 6.93 (s, 1H), 6.76 (d, *J* = 1.2 Hz, 1H), 3.30 – 3.20 (m, 4H), 3.05 (t, *J* = 6.5 Hz, 2H), 2.78 (t, *J* = 6.4 Hz, 2H), 2.45 (s, 3H), 2.32 (s, 3H), 2.06 – 1.86 (m, 4H). ^{13}C NMR (101 MHz, $CDCl_3$): δ (ppm) (*E* rotamer) 163.8, 156.4, 156.2, 150.1, 149.2, 148.9, 148.0, 145.9, 144.4, 142.4, 124.6, 122.1, 121.7, 121.0, 120.7, 118.8, 118.7, 112.2, 110.2, 107.3, 81.1, 50.2, 49.6, 27.8, 21.8, 21.7, 21.3, 21.0, 18.6. HR-ESI MS (ESI): *m/z* 447.2186 calc. for $[C_{29}H_{26}N_4O+H]^+$: 447.2179.

COUBPY ligand 3

To a solution of NaH (18 mg of a 60% dispersion in mineral oil, 0.77 mmol) and compound **6** (41 mg, 0.18 mmol) in anhydrous ACN (35 mL), thiocoumarin **9**[4] (50 mg, 0.15 mmol) was added. The maroon solution was stirred for 3 h. Then, silver nitrate (56 mg, 0.32 mmol) was added and the crude was stirred for 2 h at room temperature. The product was isolated by silica column chromatography starting with hexanes and increasing the eluent polarity with DCM first (0-100%) and then with MeOH (0-20%). 58 mg of bright red solid were obtained (yield: 75%). Bright red solid. TLC: Rf (5% MeOH/DCM) 0.5. HPLC (50-100 % B, 15 min): Rt (min) = 13.93. ^{1}H NMR (400 MHz, $CDCl_3$): δ (ppm) (*E* rotamer) 9.03 (dd, *J* = 1.9, 0.8 Hz, 1H), 8.68 (dd, *J* = 5.3, 0.8 Hz, 1H), 8.50 (d, *J* = 4.9 Hz, 1H), 8.31 – 8.24 (m, 1H), 7.58 (dd, *J* = 5.4, 2.0 Hz, 1H), 7.17 – 7.10 (m, 1H), 7.12 (d, *J* = 0.6 Hz, 1H), 7.00 (s, 1H), 3.36 – 3.18 (m, 4H), 3.00 (t, *J* = 6.5 Hz, 2H), 2.77 (t, *J* = 6.2 Hz, 2H), 2.45 (s, 3H), 2.03 – 1.82 (m, 4H). ^{13}C-NMR (101 MHz, $CDCl_3$): δ (ppm) (*E* rotamer) 161.1, 156.5, 156.0, 150.6, 149.5, 148.9, 148.2, 146.5, 141.0, 132.9 (q, *J* = 32.5 Hz), 124.9, 122.4 (q, *J* = 276.0 Hz), 122.3, 122.2, 121.5, 119.3, 119.2, 119.0, 111.2 (q, *J* = 6 Hz), 107.5, 103.4, 87.3, 77.4, 50.2, 49.6, 27.8, 21.7, 21.4, 21.3, 20.7. ^{19}F-NMR (376 MHz, $CDCl_3$): δ (ppm) -63.70. HR-ESI MS (ESI): *m/z* 501.1889 calc. for $[C_{29}H_{23}F_3N_4O+H]^+$: 501.1887.

1.3. Synthesis of Ru(II)-COUBPY complexes

SCV42

COUBPY **1** (31 mg, 0.073 mmol) and [Ru(bpy)$_2$Cl$_2$] (37 mg, 0.076 mmol) were dissolved in 3 mL of a 3:1 (v/v) solution of EtOH/H_2O. The reaction mixture was stirred overnight at 80 ºC and analysed by HPLC-MS to confirm the formation of the product. The solution mixture was evaporated to dryness and the resulting aqueous solution was lyophilised and the product was isolated by silica column chromatography starting with hexanes and increasing the eluent polarity with DCM first (0-100%) and then with MeOH (0-20%). 41 mg of red-maroon solid were obtained (yield: 62%). Red-maroon solid. TLC: Rf (35% MeOH in DCM) 0.4. HPLC (10-100 % B, 15 min): Rt (min) = 8.05. ^{1}H-NMR (400 MHz, CD_3OD): δ (ppm): (*E* rotamer) 8.78 (d, *J* = 2.1 Hz, 1H), 8.75 – 8.69 (m, 4H), 8.52 – 8.49 (m, 1H), 8.17 – 8.09 (m, 4H), 8.00 – 7.94 (m, 2H), 7.90 – 7.87 (m, 1H), 7.86 – 7.81 (m, 2H), 7.72 (d, *J* = 6.0 Hz, 1H), 7.63 (d, *J* = 5.9 Hz, 1H), 7.59 (d, *J* = 9.0 Hz, 1H), 7.56 – 7.46 (m, 4H), 7.37 – 7.33 (m, 1H), 6.84 (dd, *J* = 9.2, 2.6 Hz, 1H), 6.82 (d, *J* = 1.1 Hz, 1H), 6.77 (d, *J* = 2.4 Hz, 1H), 3.52 (q, *J* = 7.1 Hz, 4H), 2.60 (s, 3H), 2.46 (s, 3H), 1.22 (t, *J* = 7.1 Hz, 6H). ^{13}C-NMR (101 MHz, CD_3OD) δ (ppm) (*E* rotamer): 167.0, 158.7, 158.6, 158.6, 158.6, 158.3, 157.70 156.2, 153.0, 152.7, 152.62, 152.59, 152.0, 151.8, 151.7, 150.0, 144.8, 139.0, 129.7, 128.9, 128.8, 127.4, 126.0, 125.6, 124.1, 120.9, 120.2, 112.1, 111.9, 111.3, 97.8, 80.2, 45.6, 21.4, 18.6, 12.82. HR-ESI MS (ESI): *m/z* 418.1265 calc. for $[C_{47}H_{42}N_8ORu]^{2+}$: 418.1257

SCV45

COUBPY **2** (13 mg, 0.029 mmol) and [Ru(bpy)$_2$Cl$_2$] (14 mg, 0.029 mmol) were dissolved in 3 mL of a 3:1 (v/v) solution of EtOH/H_2O. The reaction mixture was stirred overnight at 80ºC and analysed by HPLC-MS to confirm the formation of the product. The solvent was evaporated to dryness and the product was isolated by silica column chromatography starting with hexanes and increasing the eluent polarity with DCM first (0-100%) and then with MeOH (0-20%). 25 mg of dark-maroon solid were obtained (yield: 93%). Dark-maroon solid. TLC: Rf (1:9 MeOH/DCM) 0.5. HPLC (10-100 % B, 15 min): Rt (min) = 8.17. ^{1}H NMR (500 MHz, CD_3OD): δ (ppm): 8.83 (d, *J* = 2.2 Hz, 1H), 8.77 – 8.69 (m, 4H), 8.55 (s, 1H), 8.19 – 8.08 (m, 4H), 7.96 (d, *J* = 5.5 Hz, 1H), 7.87 (d, *J* = 5.7 Hz, 1H), 7.83 (d, *J* = 4.9 Hz, 2H), 7.70 (dd, *J* = 6.2, 2.2 Hz, 1H), 7.64 (dd, *J* = 6.3, 2.2 Hz, 2H), 7.56 – 7.46 (m, 4H), 7.35 (d, *J* = 5.9 Hz, 1H), 7.19 (s, 1H), 6.75 (s, 1H), 3.37 – 3.32 (m, 4H), 2.88 (t, *J* = 6.1 Hz, 2H), 2.82 (t, *J* = 6.4 Hz, 2H), 2.58 (s, 3H), 2.42 (s, 3H), 2.01 – 1.94 (m, 2H), 1.93 – 1.86 (m, 2H). ^{13}C-NMR (101 MHz, CD_3OD) δ (ppm):

167.7, 158.7, 158.2, 157.7, 152.6, 152.5, 152.0, 151.8, 151.6, 151.5, 150.1, 148.0, 146.2, 139.1, 129.8, 128.9, 125.9, 125.6, 124.5, 123.5, 121.8, 121.5, 120.6, 111.5, 111.4, 106.9, 79.6, 79.3, 79.2, 79.0, 51.1, 50.3, 30.8, 28.7, 22.7, 22.4, 21.6, 21.3, 18.8. HR-ESI MS (ESI): *m/z* 430.1258, calc. for $[C_{49}H_{42}N_8ORu]^{2+}$: 430.1257.

SCV49

COUBPY **3** (31.6 mg, 0.063 mmol) and $[Ru(bpy)_2Cl_2]$ (37.4 mg, 0.077 mmol) were dissolved in 2 mL of a 3:1 (v/v) solution of EtOH/H_2O. The reaction was stirred overnight at 80ºC and analysed by HPLC-MS to confirm the formation of the product. The solvent was evaporated to dryness and the product was isolated by silica column chromatography starting with hexanes and increasing the eluent polarity with DCM first (0-100%) and then with MeOH (0-16%). 51.4 mg of dark-purple solid were obtained (yield: 82%). TLC: Rf (30% MeOH in DCM) 0.4. HPLC (10-100 % B, 15 min): Rt (min) = 8.42. ^{1}H NMR (400 MHz, CD_3OD): δ (ppm) (*E* rotamer) 8.94 (s, 1H), 8.73 (d, *J* = 8.2 Hz, 4H), 8.62 (s, 1H), 8.19 – 8.09 (m, 4H), 7.98 – 7.94 (m, 1H), 7.91 – 7.81 (m, 3H), 7.80 – 7.76 (m, 1H), 7.76 – 7.72 (m, 1H), 7.66 (d, *J* = 5.6 Hz, 1H), 7.60 – 7.46 (m, 4H), 7.38 (d, *J* = 4.6 Hz, 1H), 7.08 (s, 1H), 7.02 (s, 1H), 3.43 – 3.34 (m, 4H), 2.87 – 2.82 (m, 2H), 2.79 (t, *J* = 6.2 Hz, 2H), 2.60 (s, 3H), 2.02 – 1.93 (m, 2H), 1.93 – 1.84 (m, 2H). ^{13}C NMR (101 MHz, CD_3OD) δ (ppm) (*E* rotamer): 163.9, 158.64, 158.61, 158.58, 158.53, 158.3, 157.9, 152.7, 152.6, 152.5, 152.2, 152.1, 152.0, 151.9, 148.5, 143.4, 139.23, 139.19, 135.5 (q, *J* = 32.3 Hz), 130.0, 128.9, 126.2, 125.7, 125.5, 123.6 (q, *J* = 274.7 Hz), 123.3, 122.5, 122.0, 119.1, 109.8 (q, *J* = 6.1 Hz), 107.7, 104.4, 85.5, 51.1, 50.3, 28.7, 22.5, 22.1, 21.3. ^{19}F NMR (376 MHz, $CDCl_3$) δ (ppm) -65.0 (*E* rotamer), -64.8 (*Z* rotamer). HR-ESI MS (ESI): *m/z* 457.1102 calc. for $[C_{49}H_{39}N_8OF_3Ru]^{2+}$: 457.1115.

1.4. HPLC analysis of the compounds

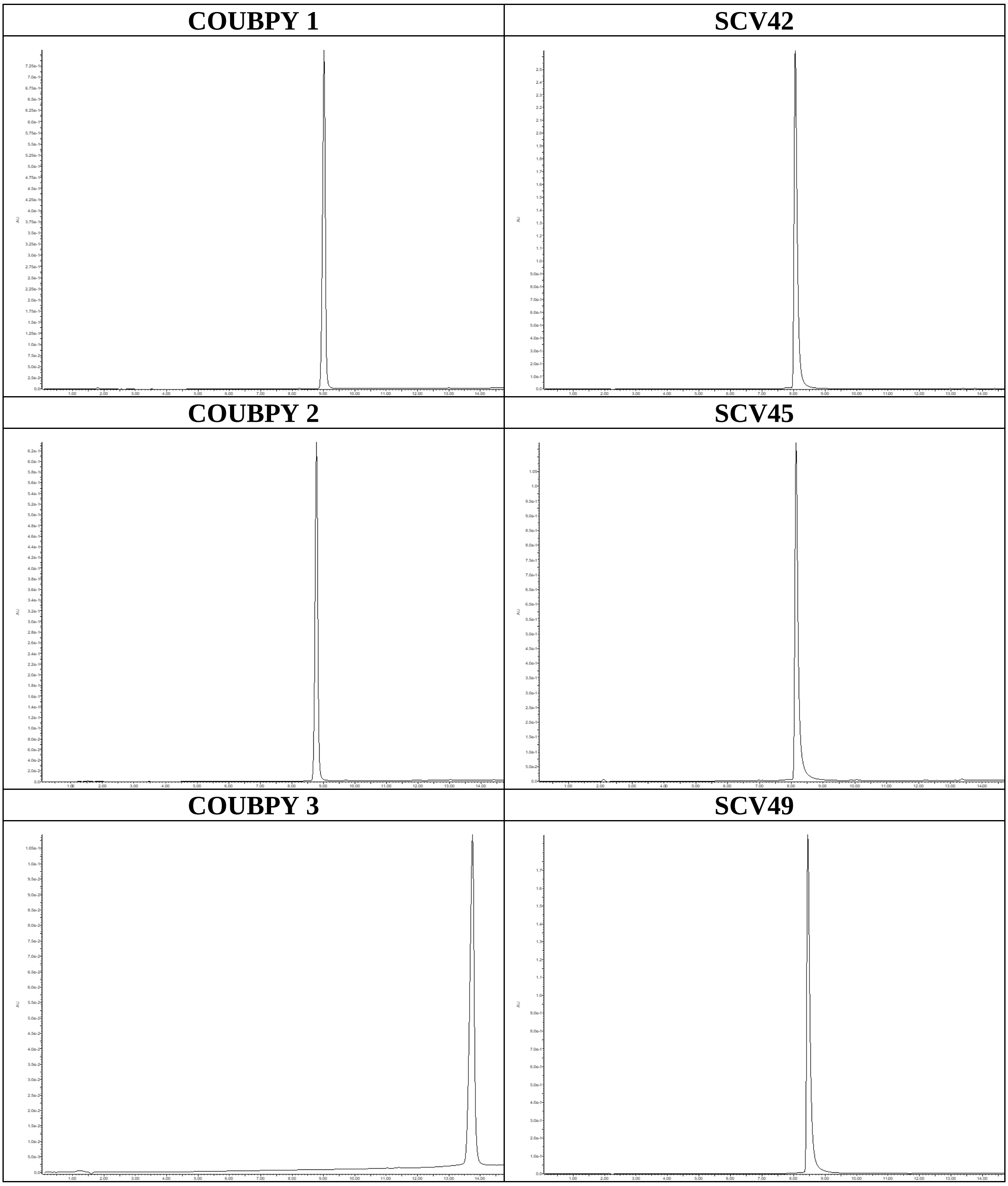

Figure S1. Reversed-phase HPLC analysis of COUBPY ligands and Ru-COUBPY complexes.

2. 1D ^{1}H, ^{19}F and ^{13}C NMR spectra and HR ESI-MS of the compounds

4-(Chloromethyl)-4'-methyl-2,2'-bipyridine (5)

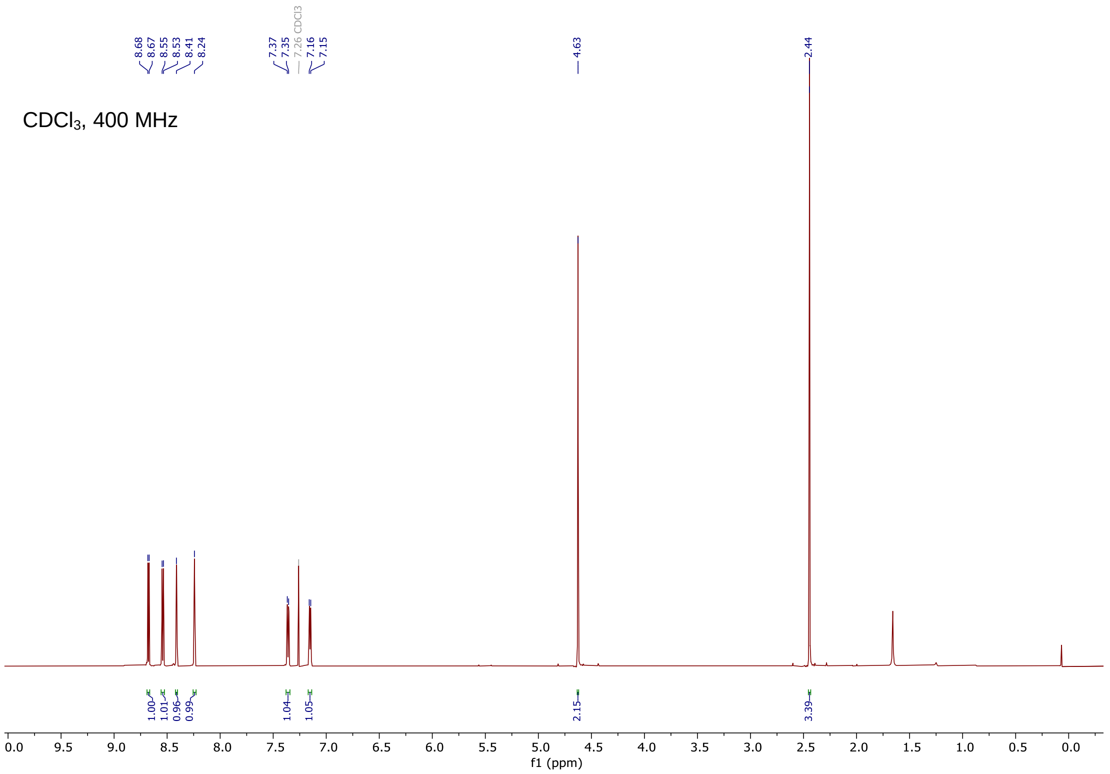

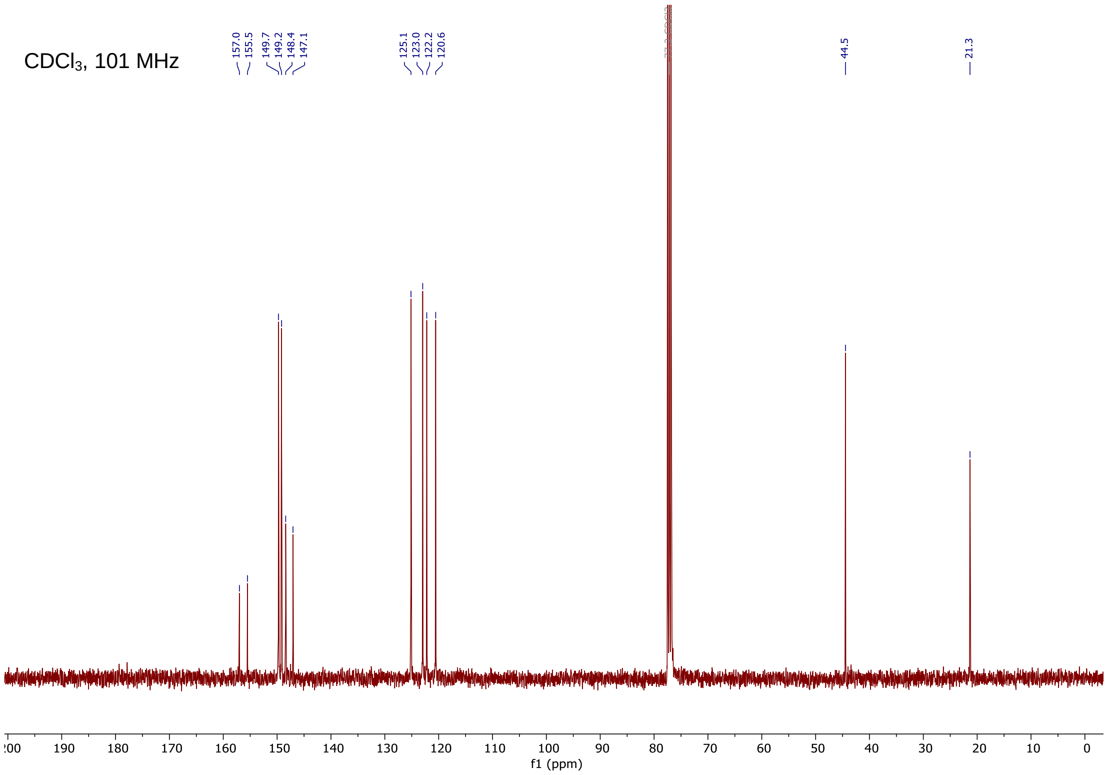

Figure S2. ^{1}H and ^{13}C NMR spectra of compound **5** in $CDCl_3$.

2-(4'-Methyl-[2,2'-bipyridin]-4-yl) acetonitrile (6)

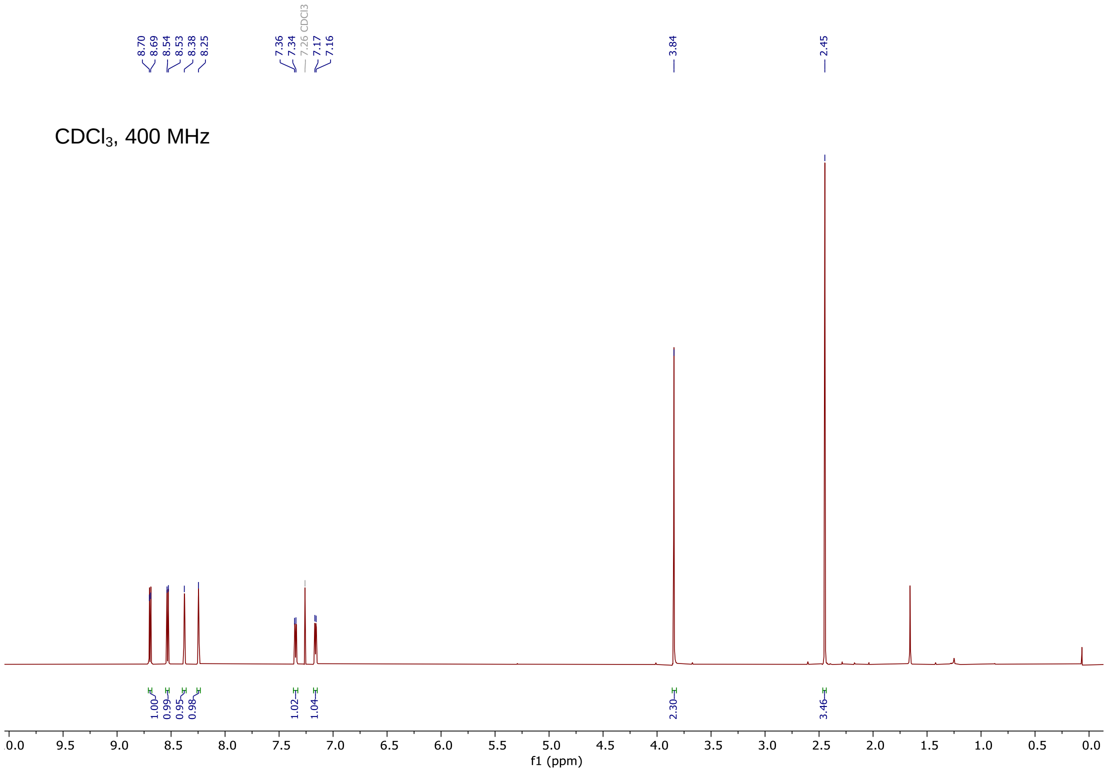

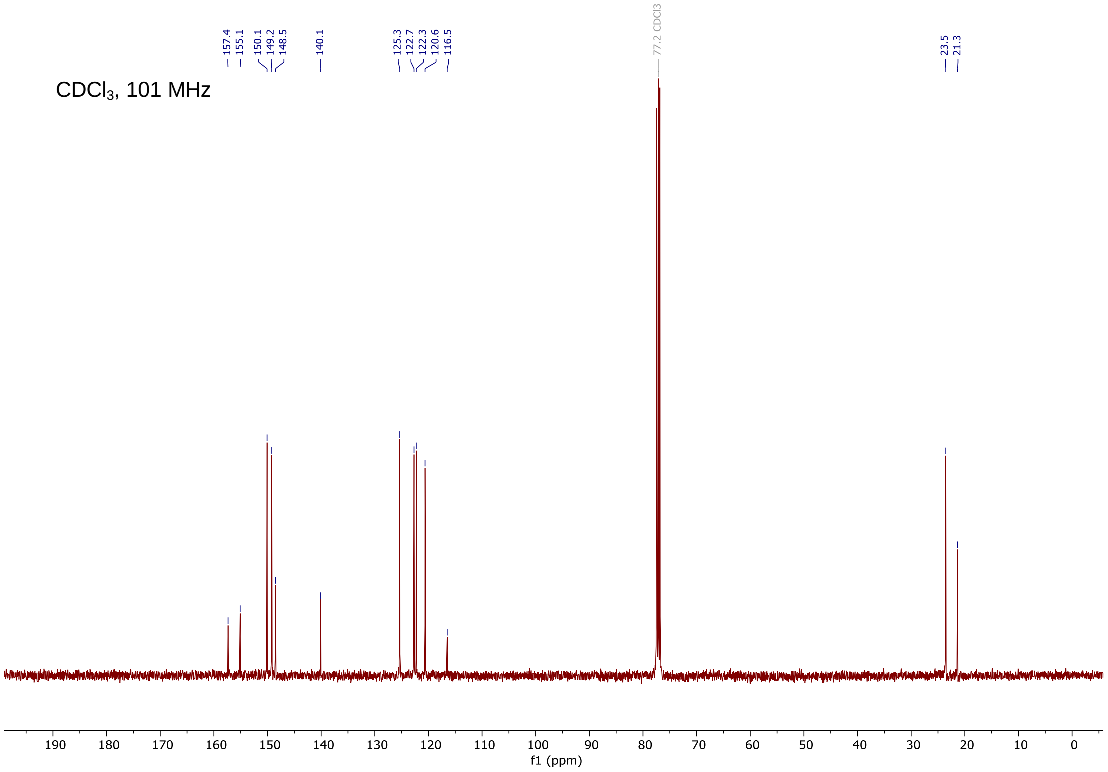

Figure S3. ^{1}H and ^{13}C NMR spectra of compound **6** in $CDCl_3$.

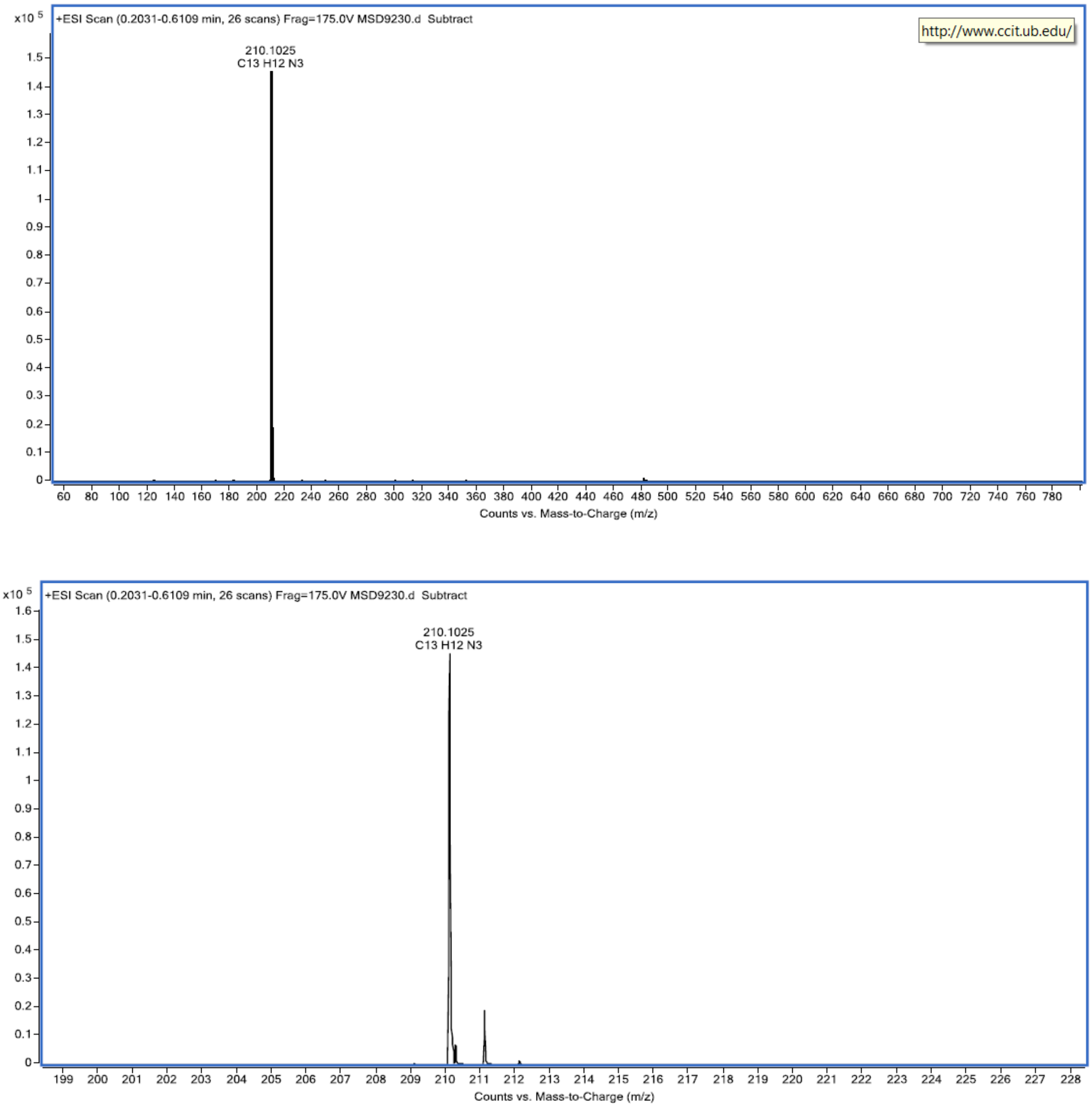

Figure S4. HR ESI-MS spectrum of compound **6**.

COUBPY 1

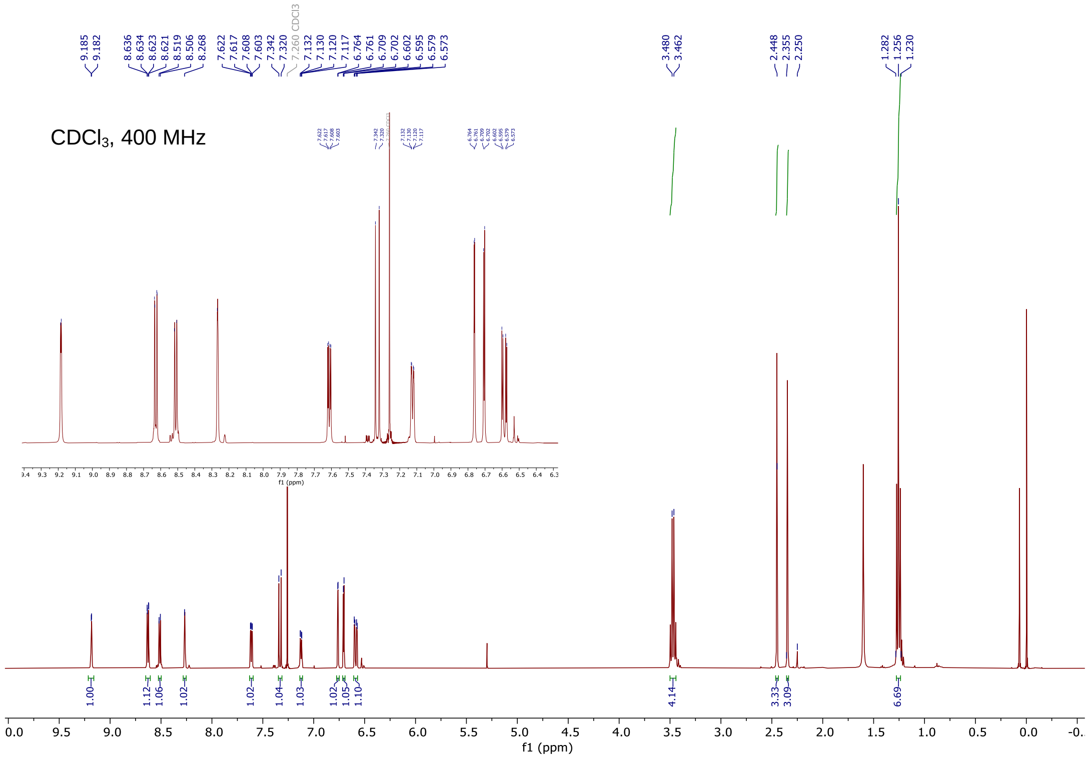

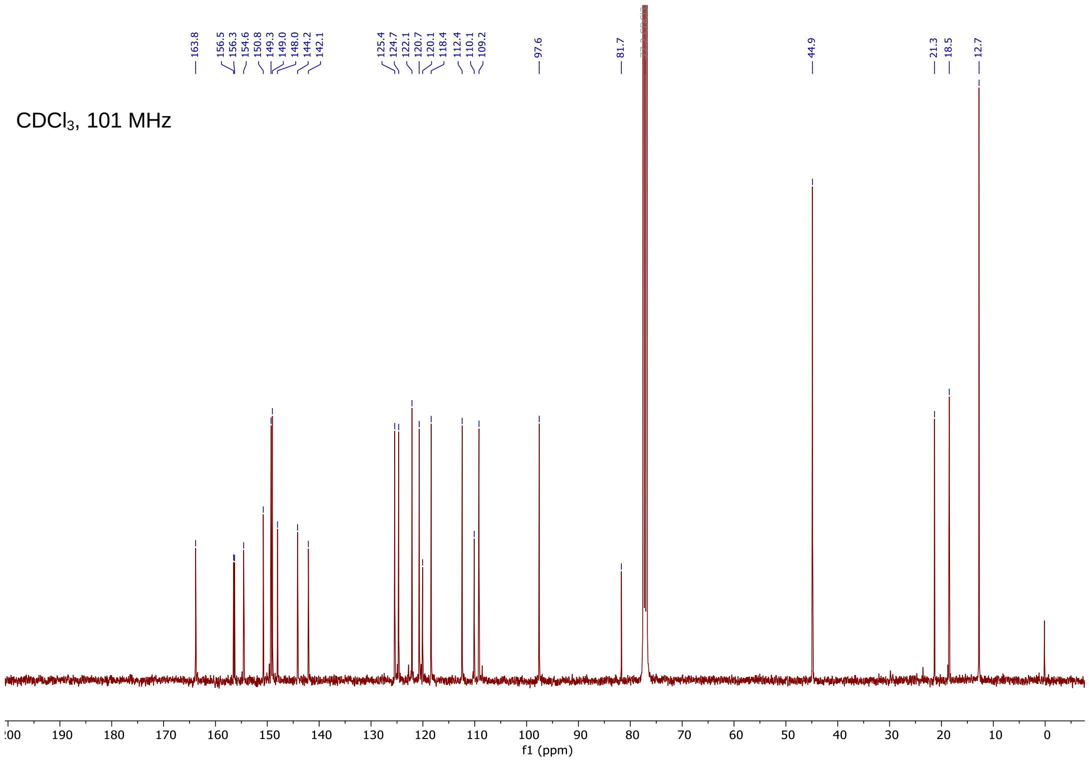

Figure S5. ^{1}H and ^{13}C NMR spectra of COUBPY ligand **1** in $CDCl_3$.

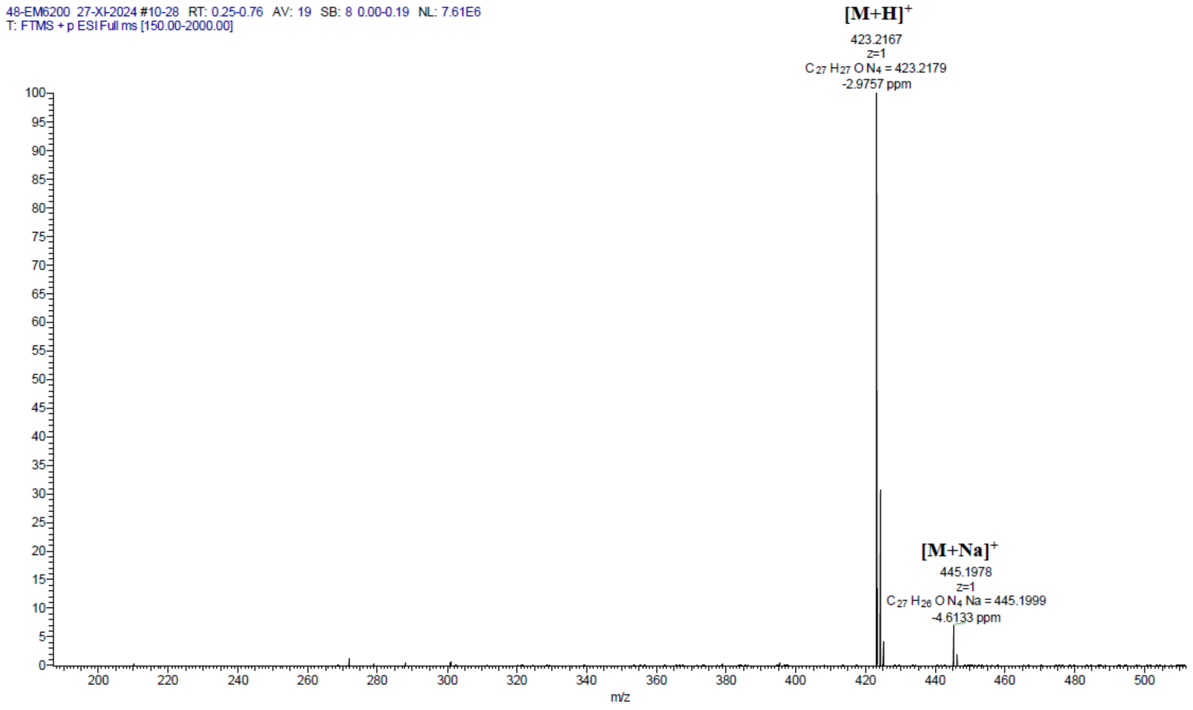

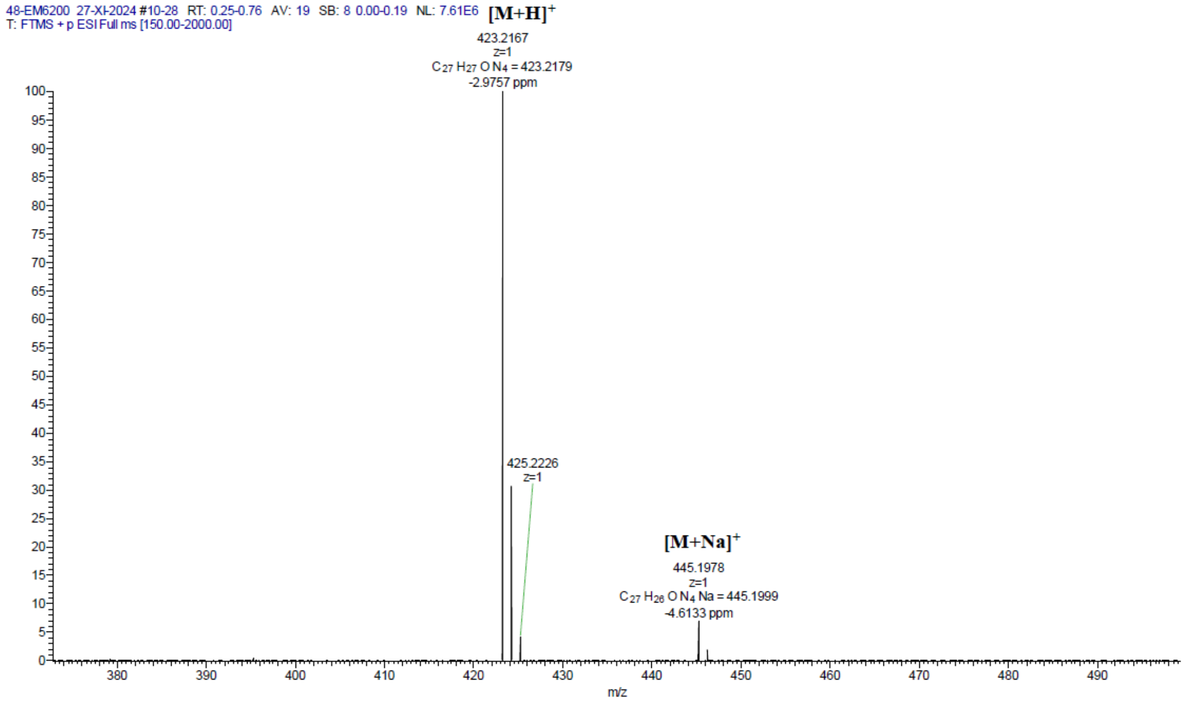

Figure S6. HR ESI-MS spectrum of COUBPY ligand **1.**

COUBPY 2

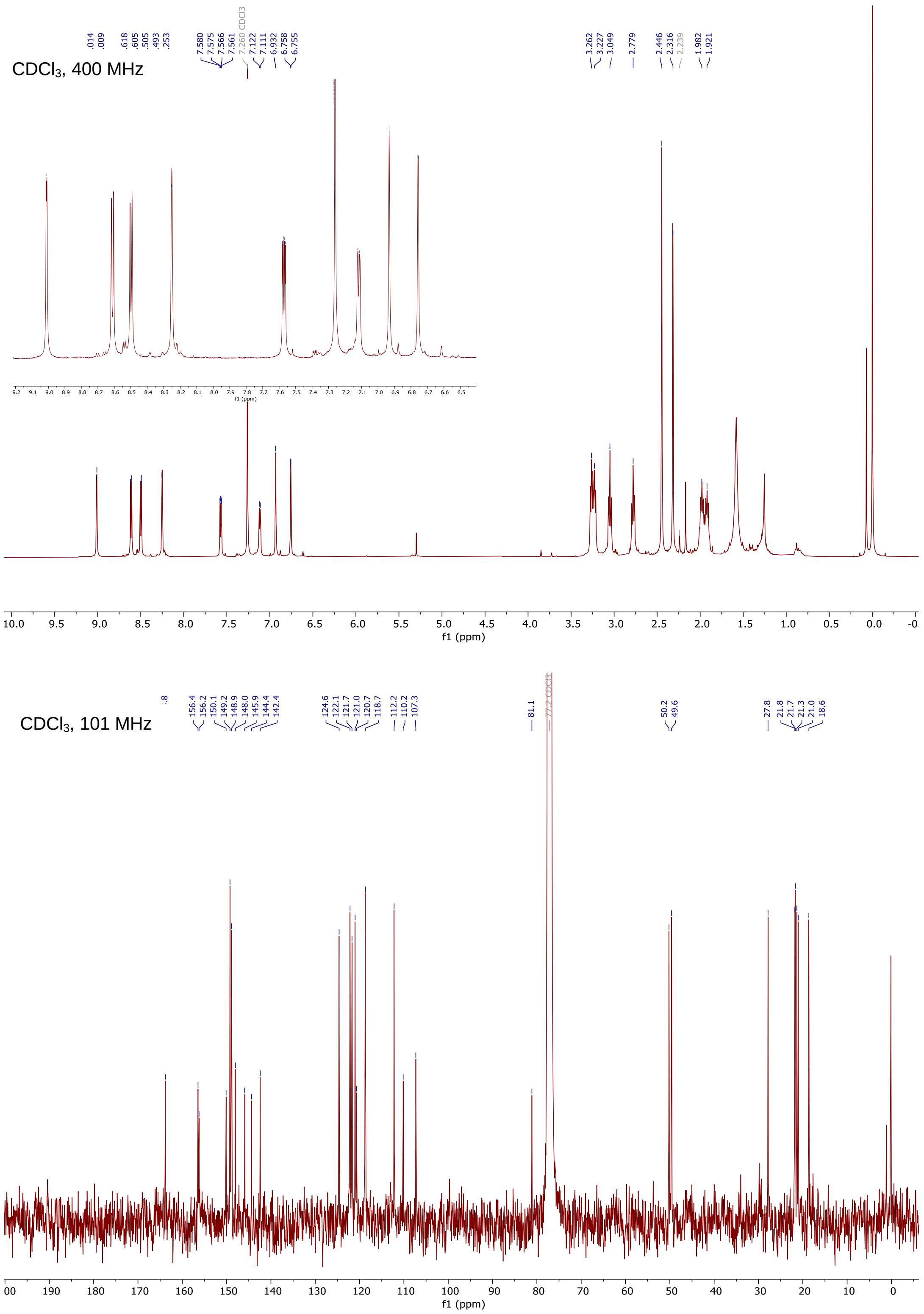

Figure S7. ^{1}H and ^{13}C NMR spectra of COUBPY ligand **2** in $CDCl_3$.

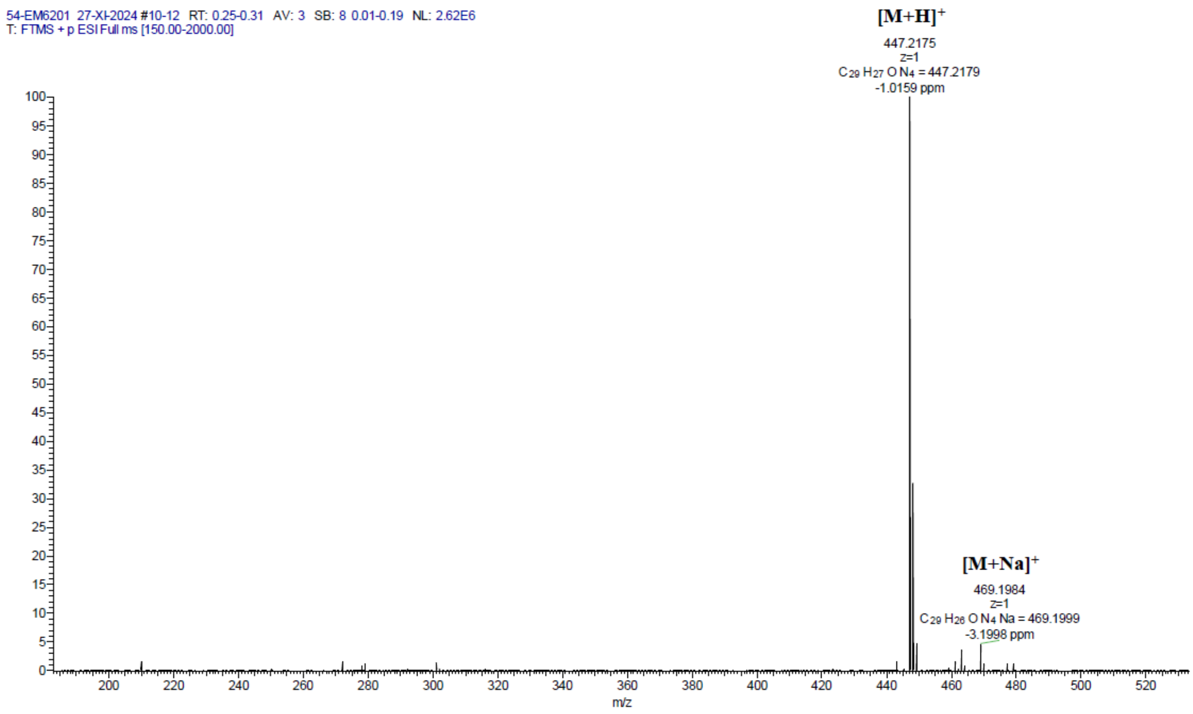

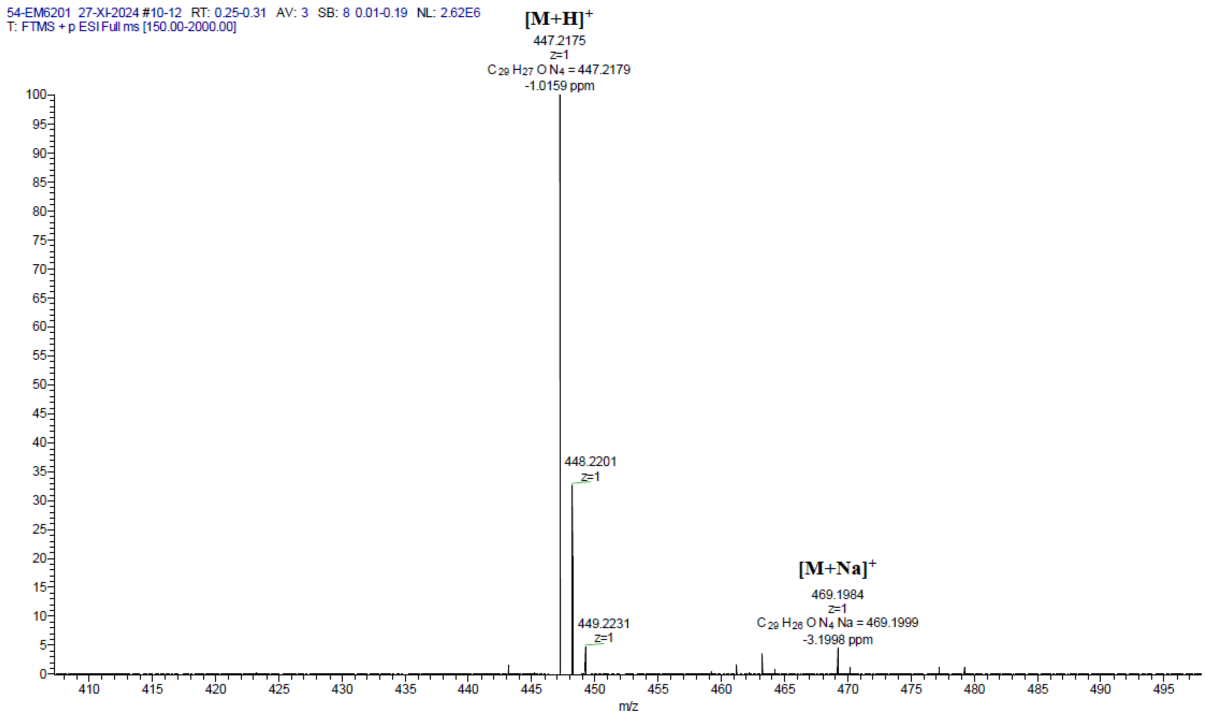

Figure S8. HR ESI-MS spectrum of COUBPY ligand **2**.

COUBPY 3

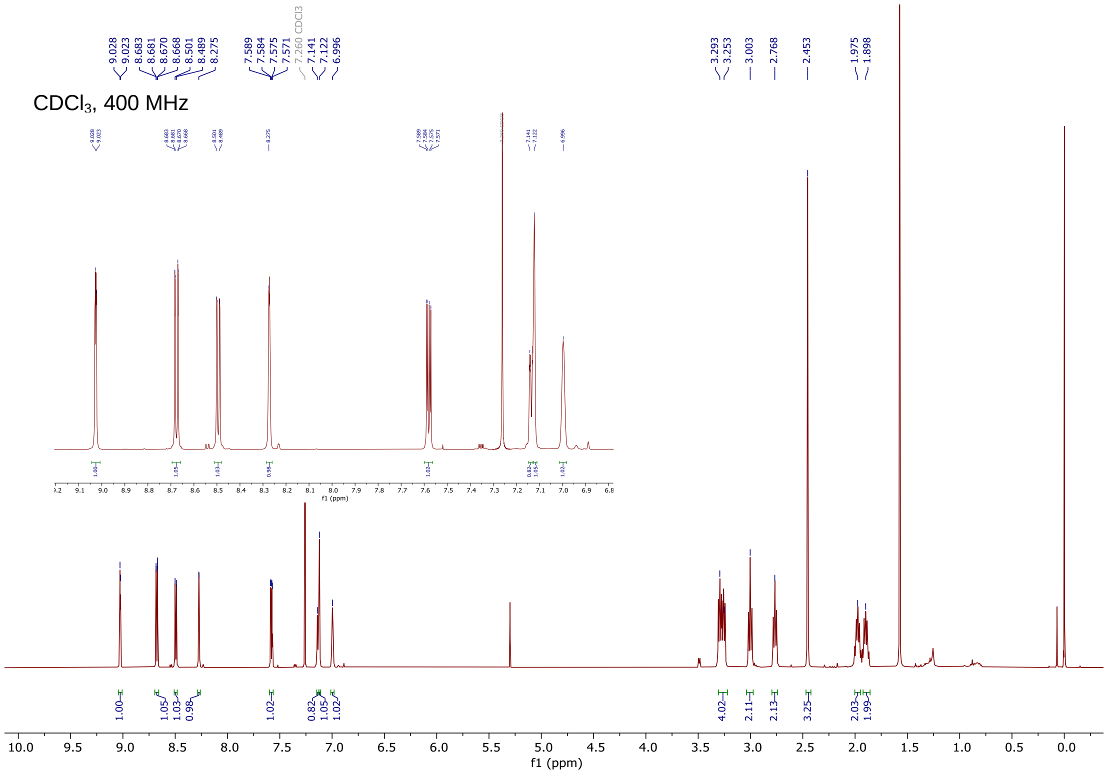

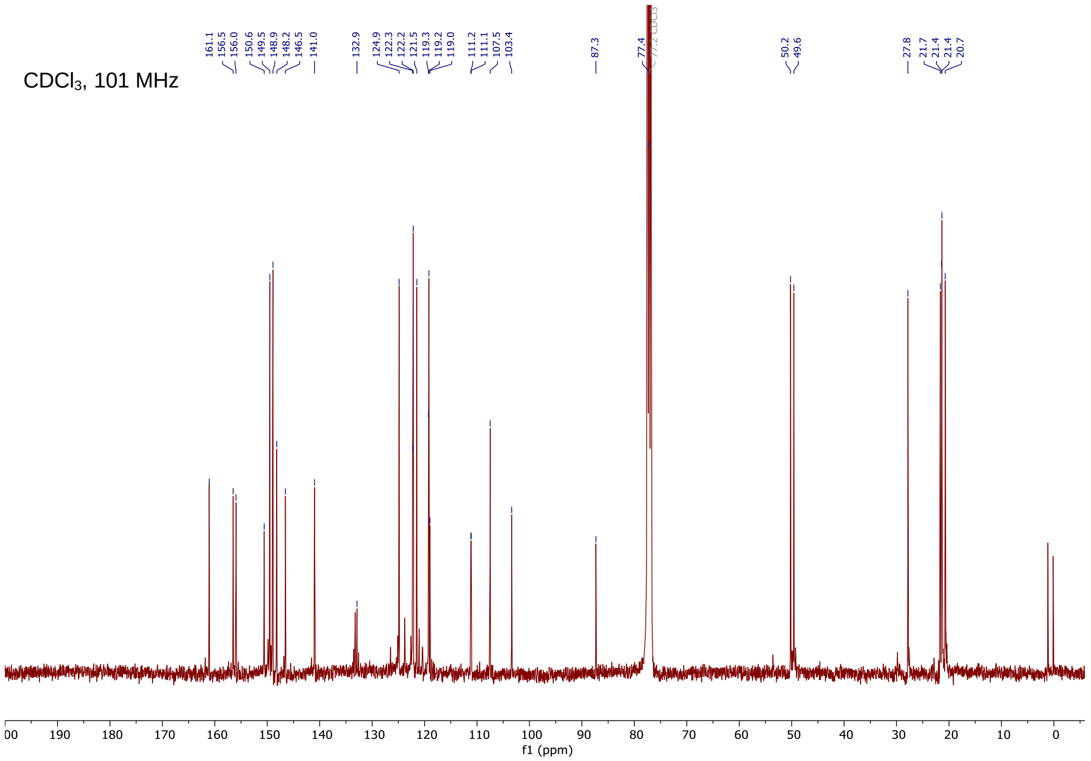

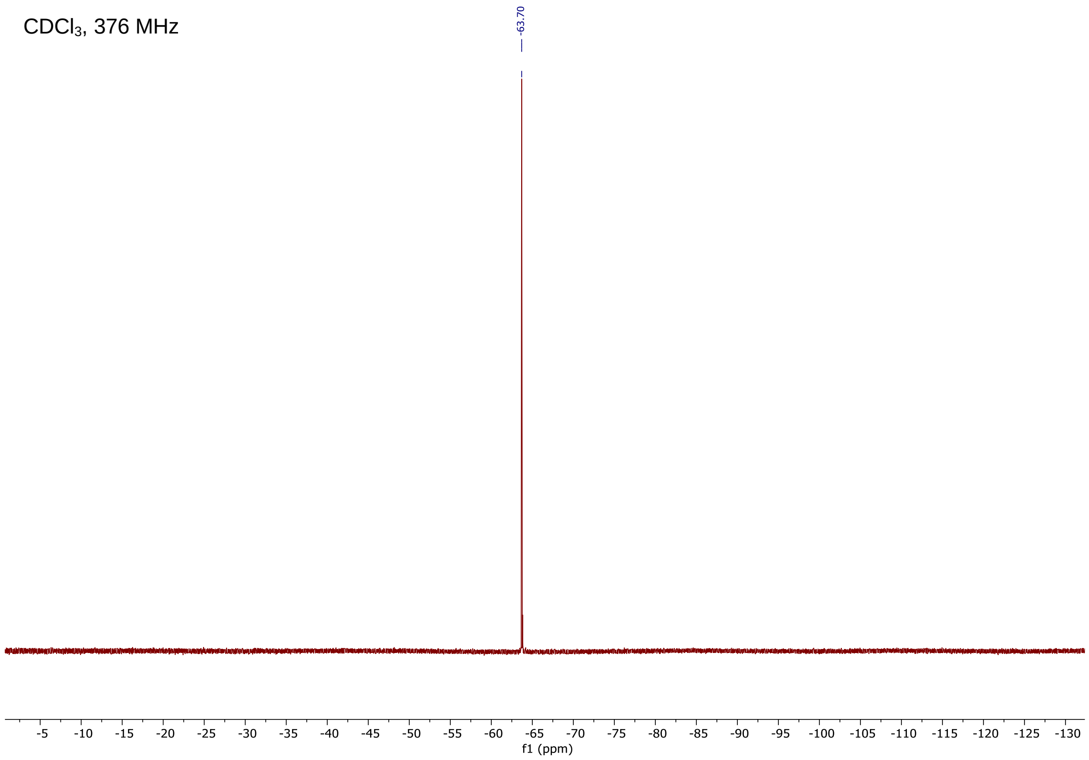

Figure S9. ^{1}H, ^{13}C and ^{19}F NMR spectra of COUBPY ligand **3** in $CDCl_3$.

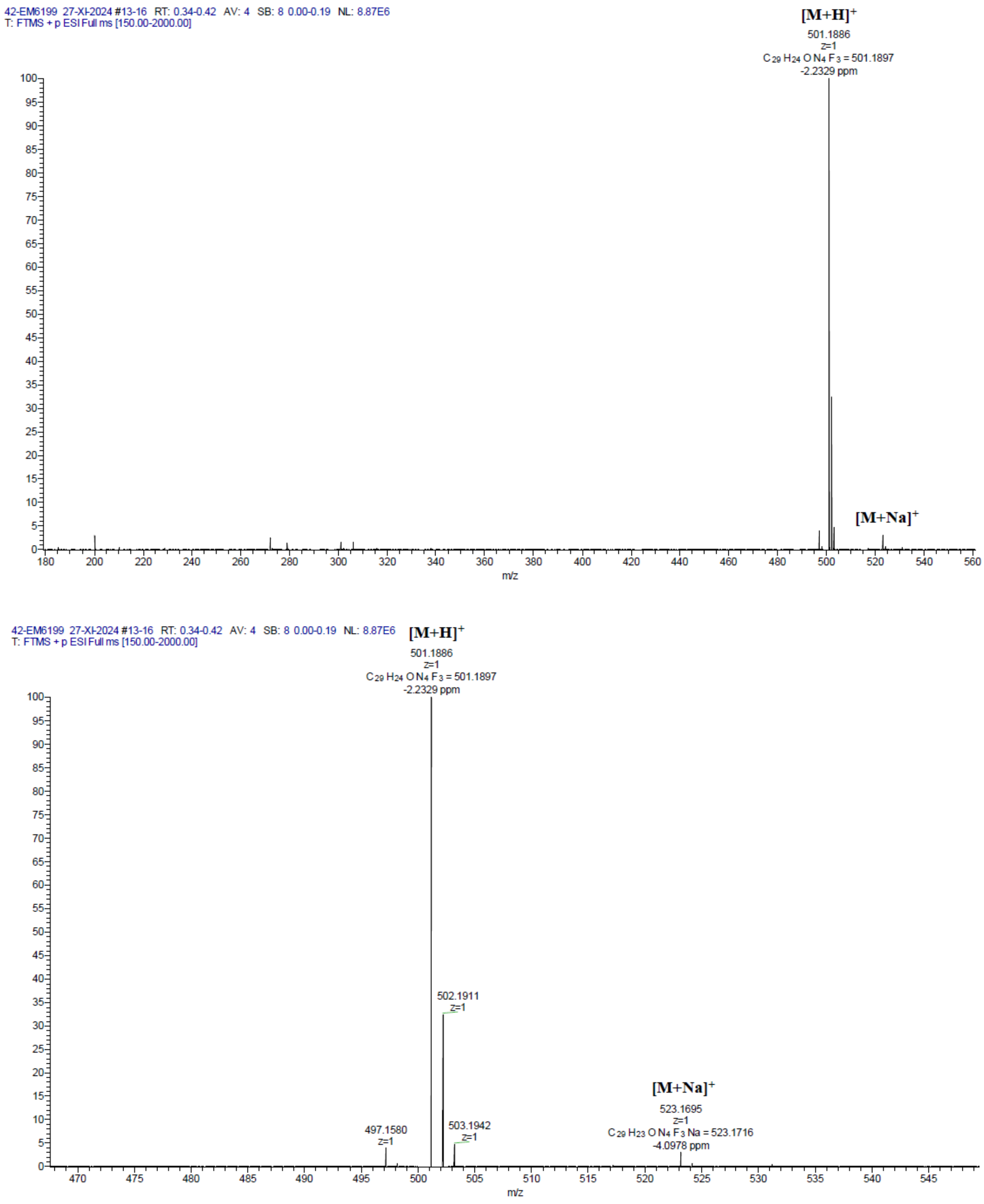

Figure S10. HR ESI-MS spectrum of COUBPY ligand **3**.

SCV42

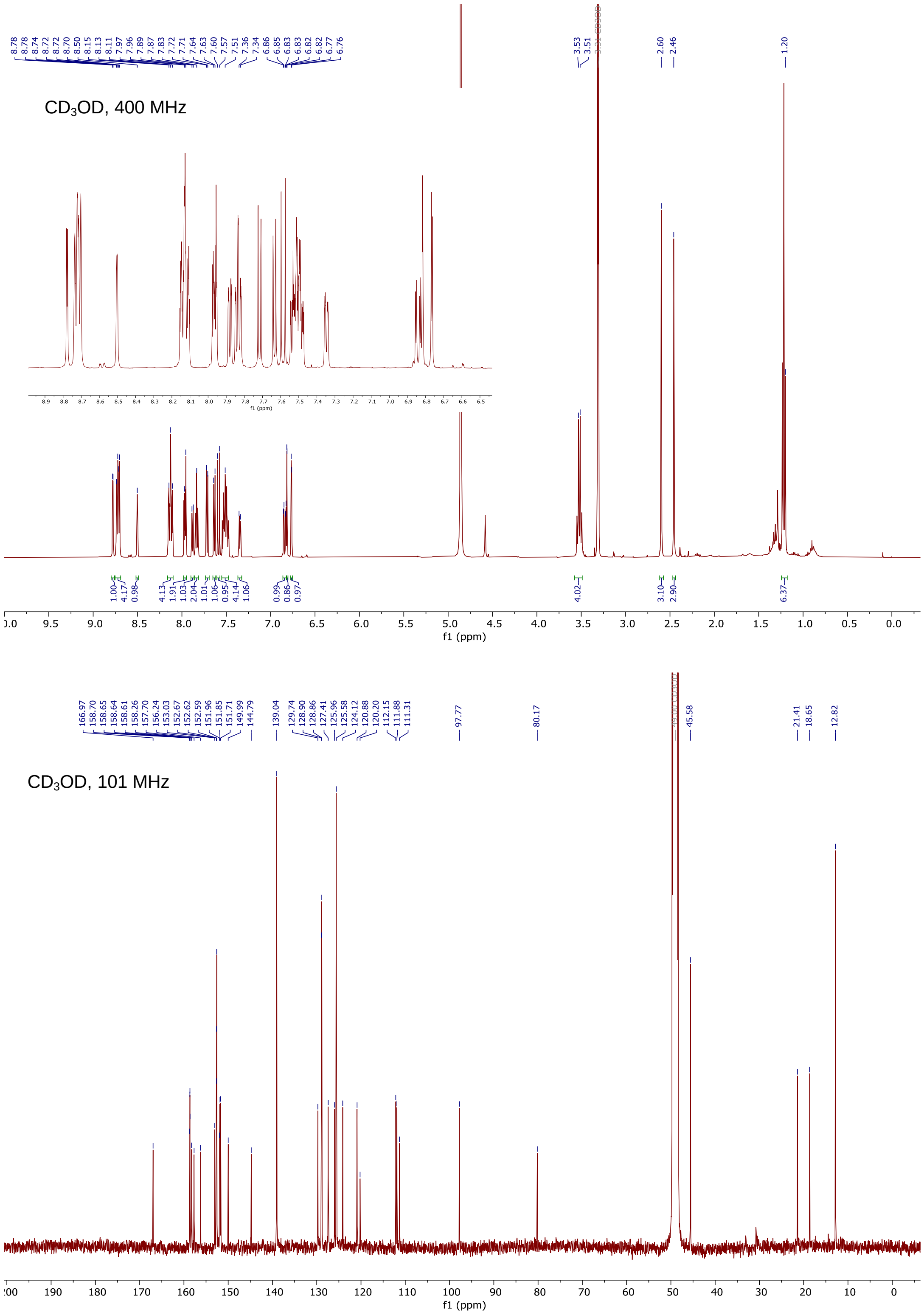

Figure 11. ^{1}H and ^{13}C NMR spectra of Ru-COUBPY complex **SCV42** in CD$_3$OD.

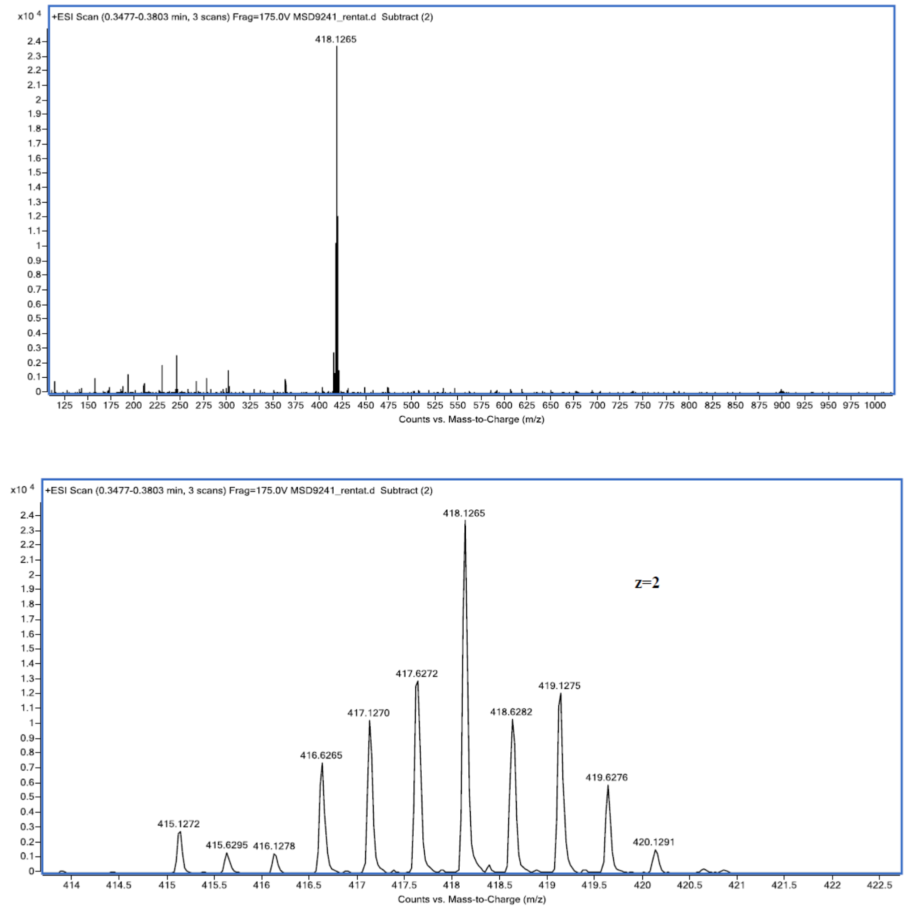

Figure S12. HR ESI-MS spectrum of Ru-COUBPY complex **SCV42**.

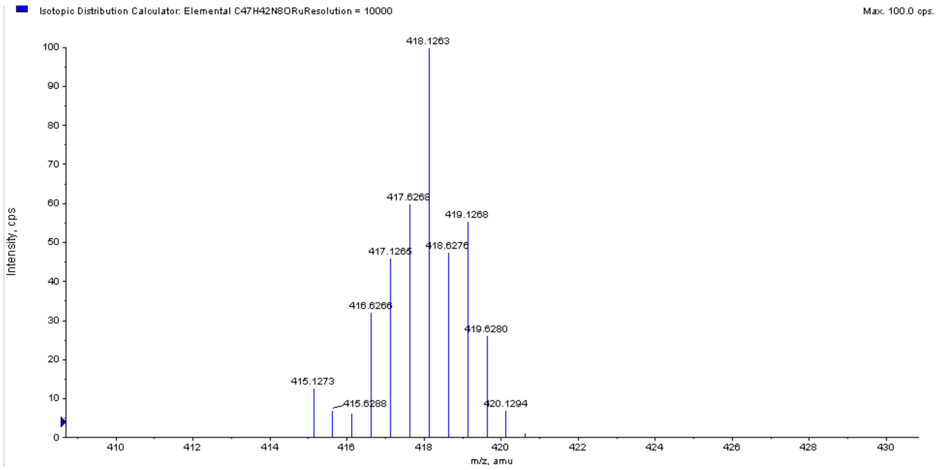

Figure S13. Predicted HR ESI-MS spectrum of Ru-COUBPY complex **SCV42**.

SCV45

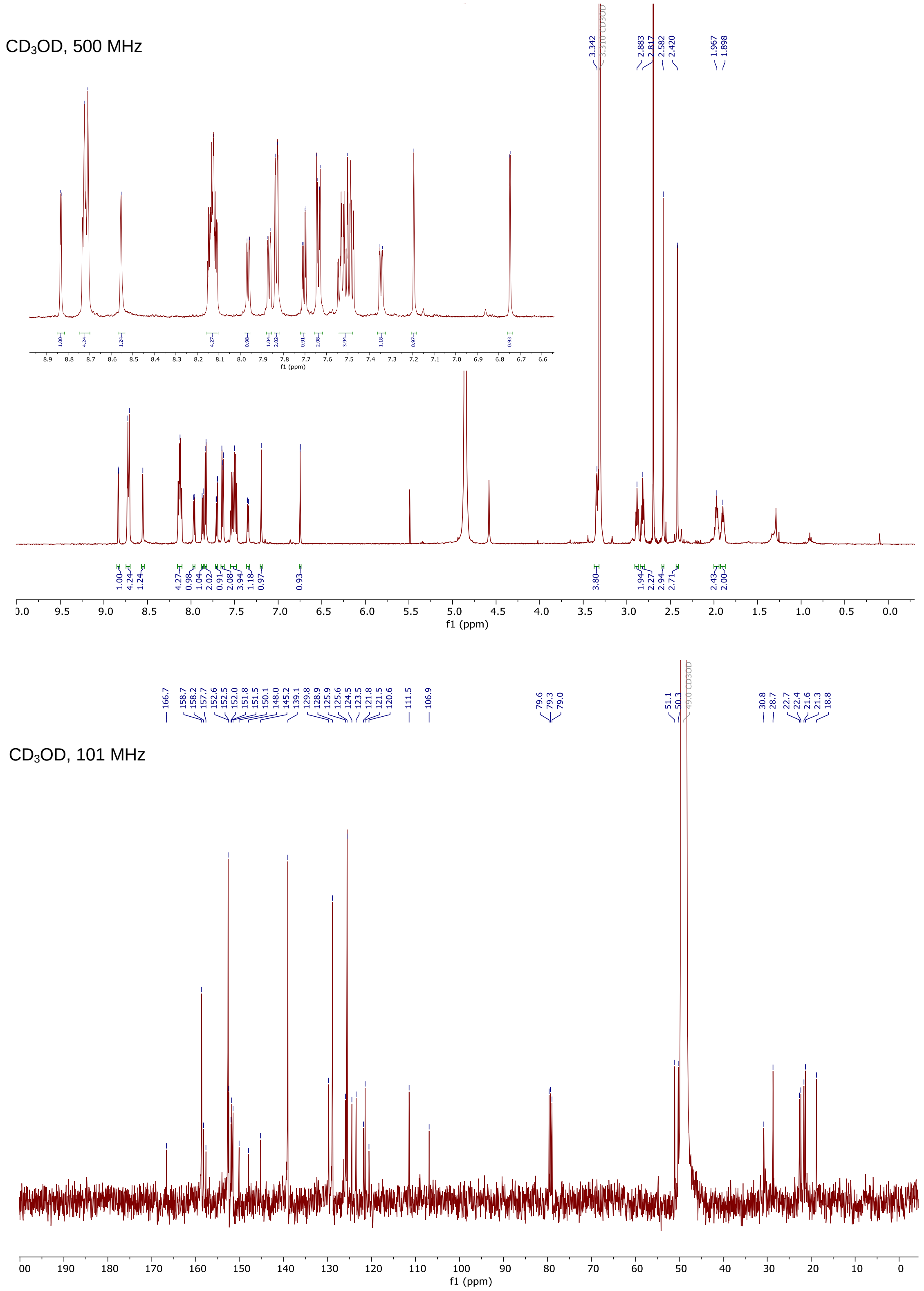

Figure S14. ^{1}H and ^{13}C NMR spectra of Ru-COUBPY complex **SCV45** in CD$_3$OD.

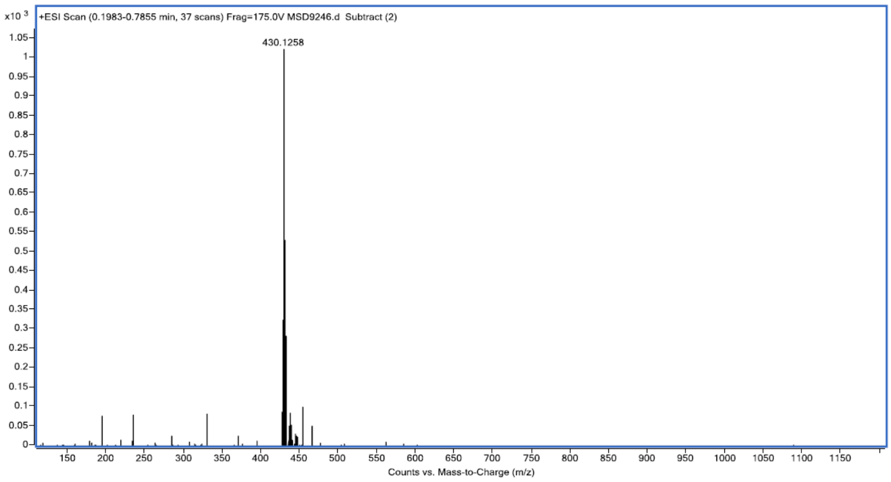

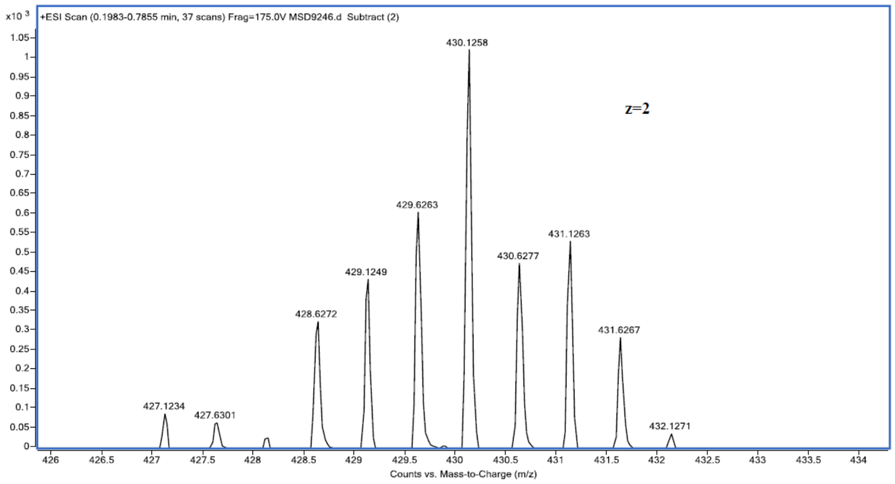

Figure S15. HR ESI-MS spectrum of Ru-COUBPY complex **SCV45**.

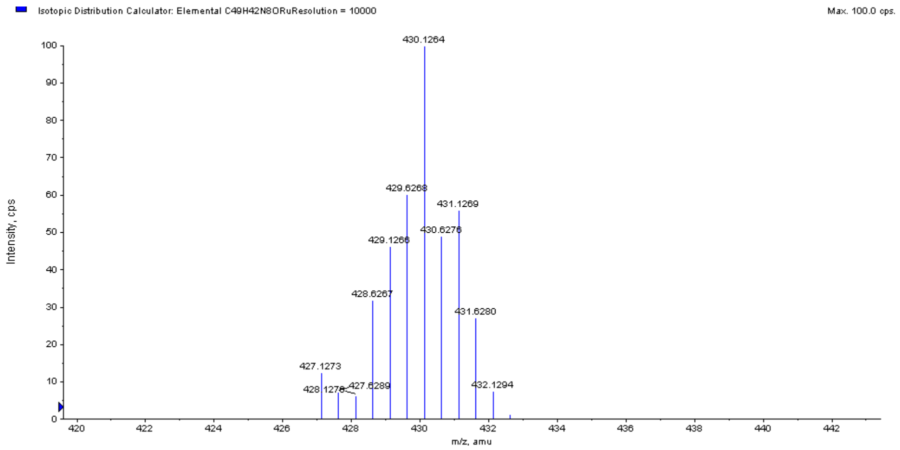

Figure S16. Predicted HR ESI-MS spectrum of Ru-COUBPY complex **SCV45**.

SCV49

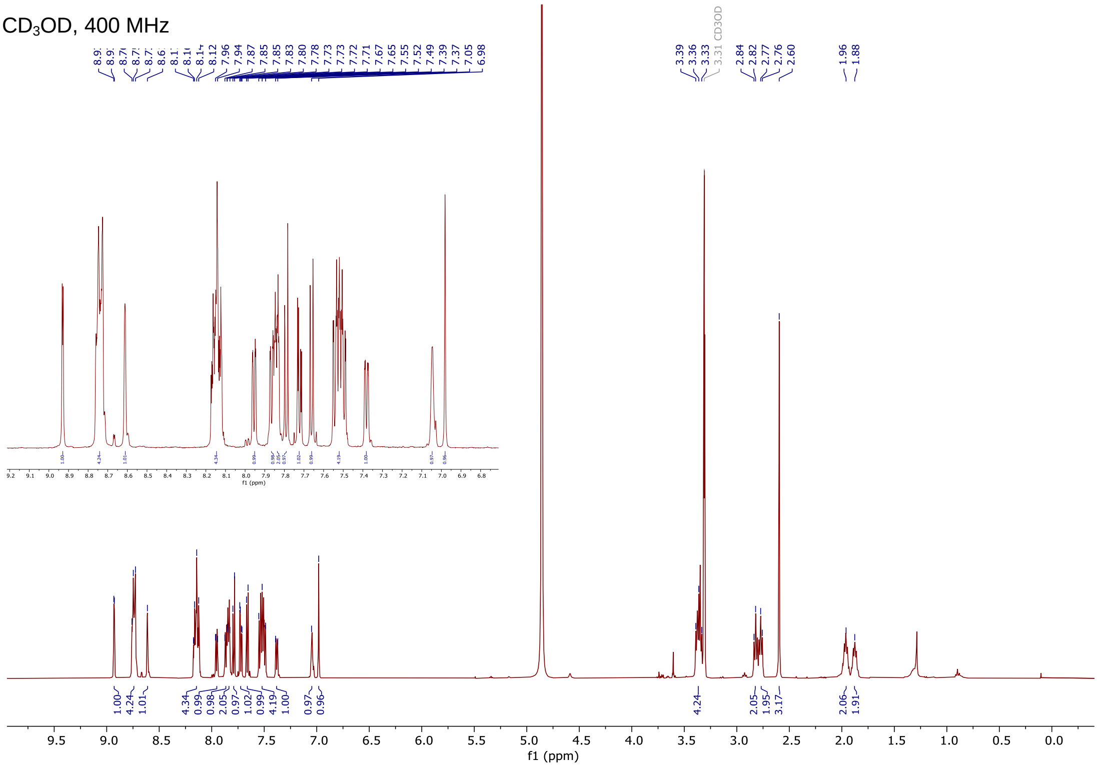

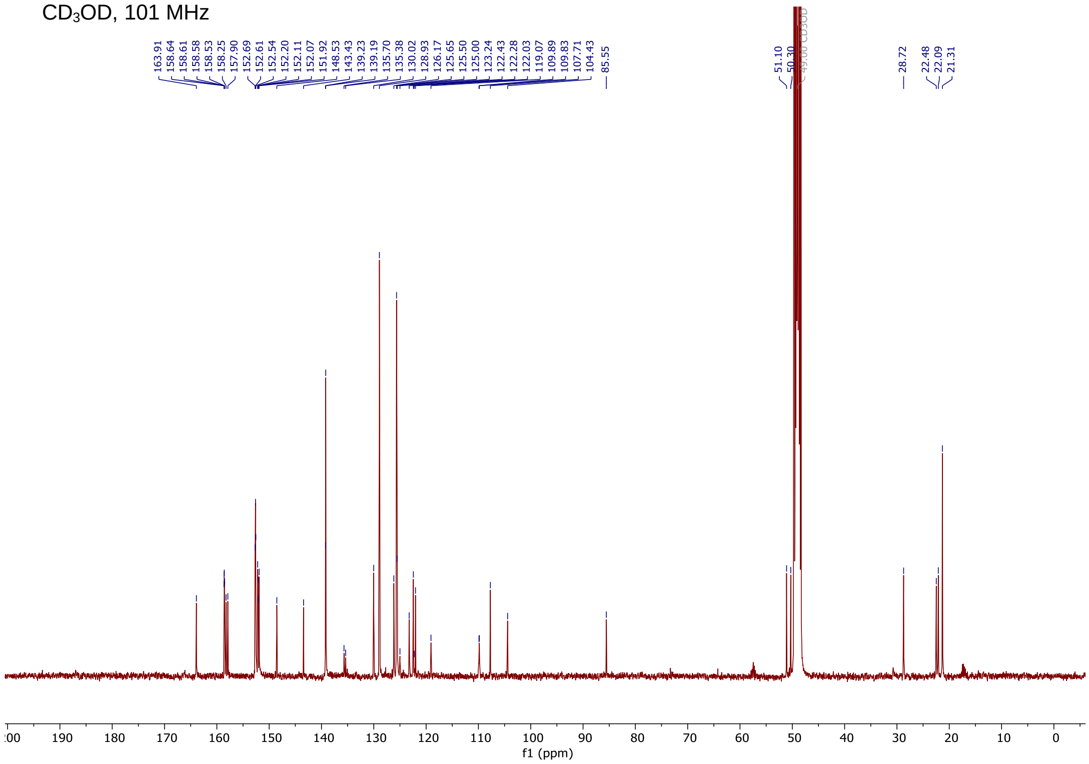

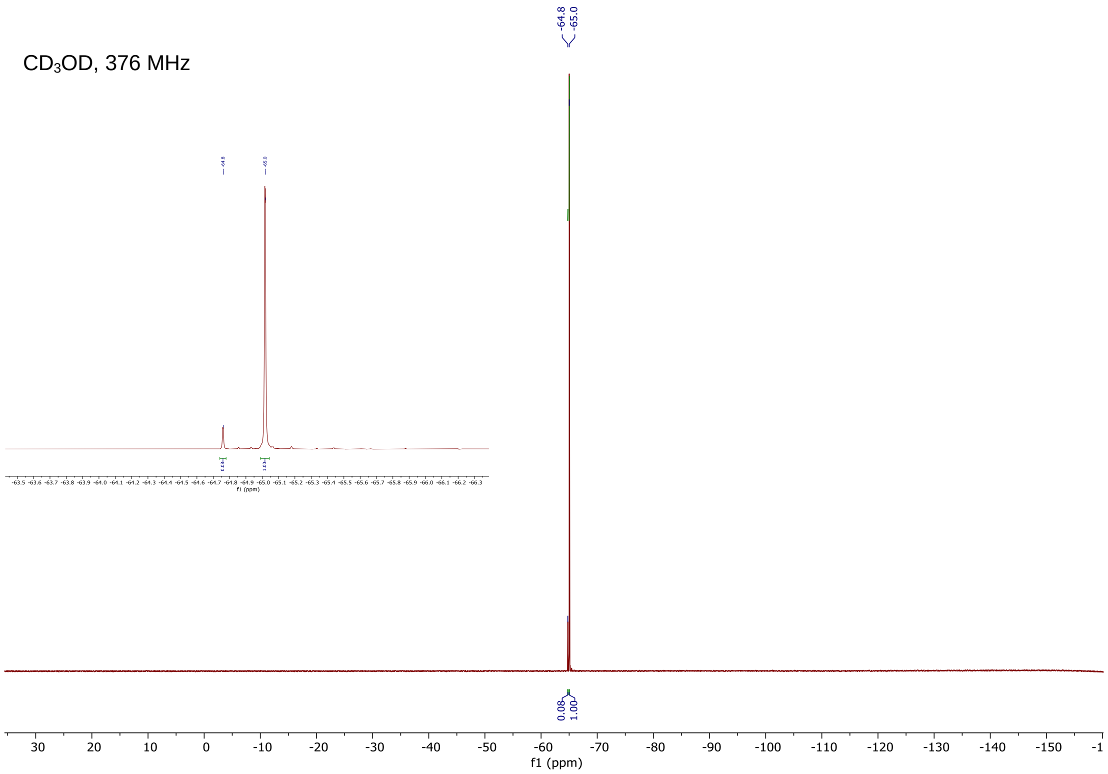

Figure S17. ^{1}H, ^{13}C and ^{19}F NMR spectra of Ru-COUBPY complex **SCV49** in CD_3OD.

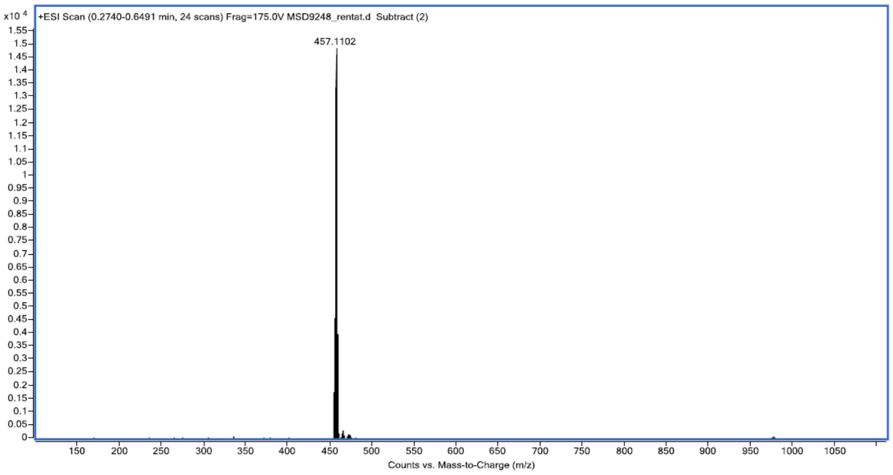

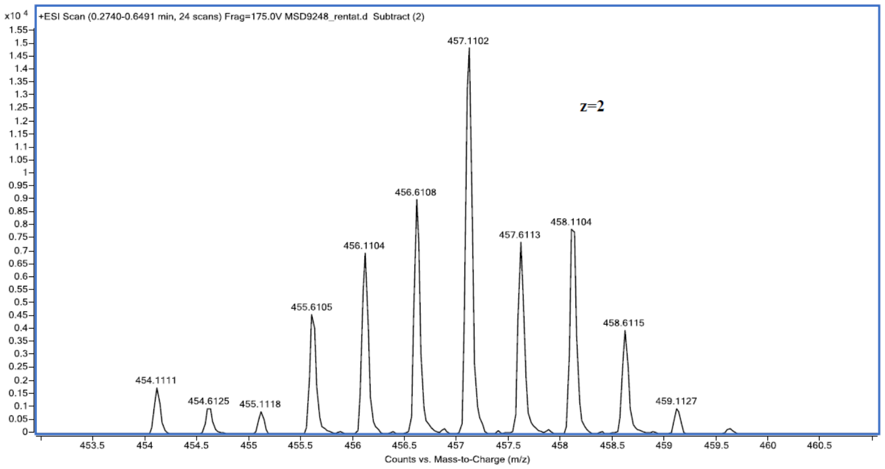

Figure S18. HR ESI-MS spectrum of Ru-COUBPY complex **SCV49**.

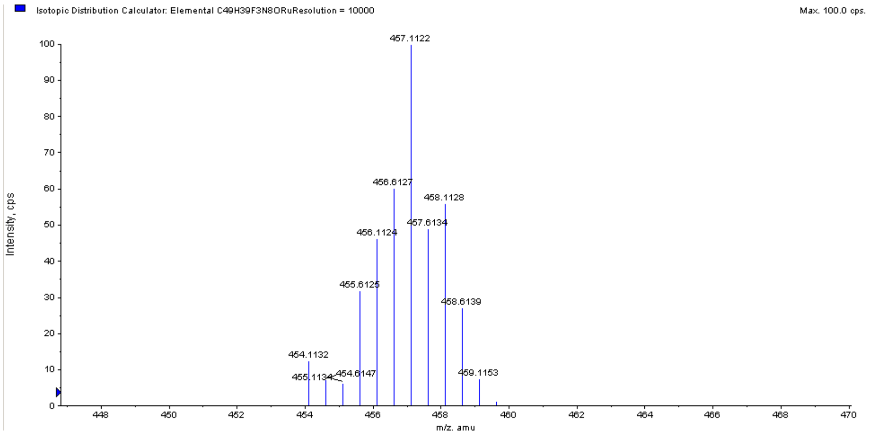

Figure S19. Predicted HR ESI-MS spectrum of Ru-COUBPY complex **SCV49**.

3. NOESY spectra of COUBPY ligands and Ru-COUBPY complexes

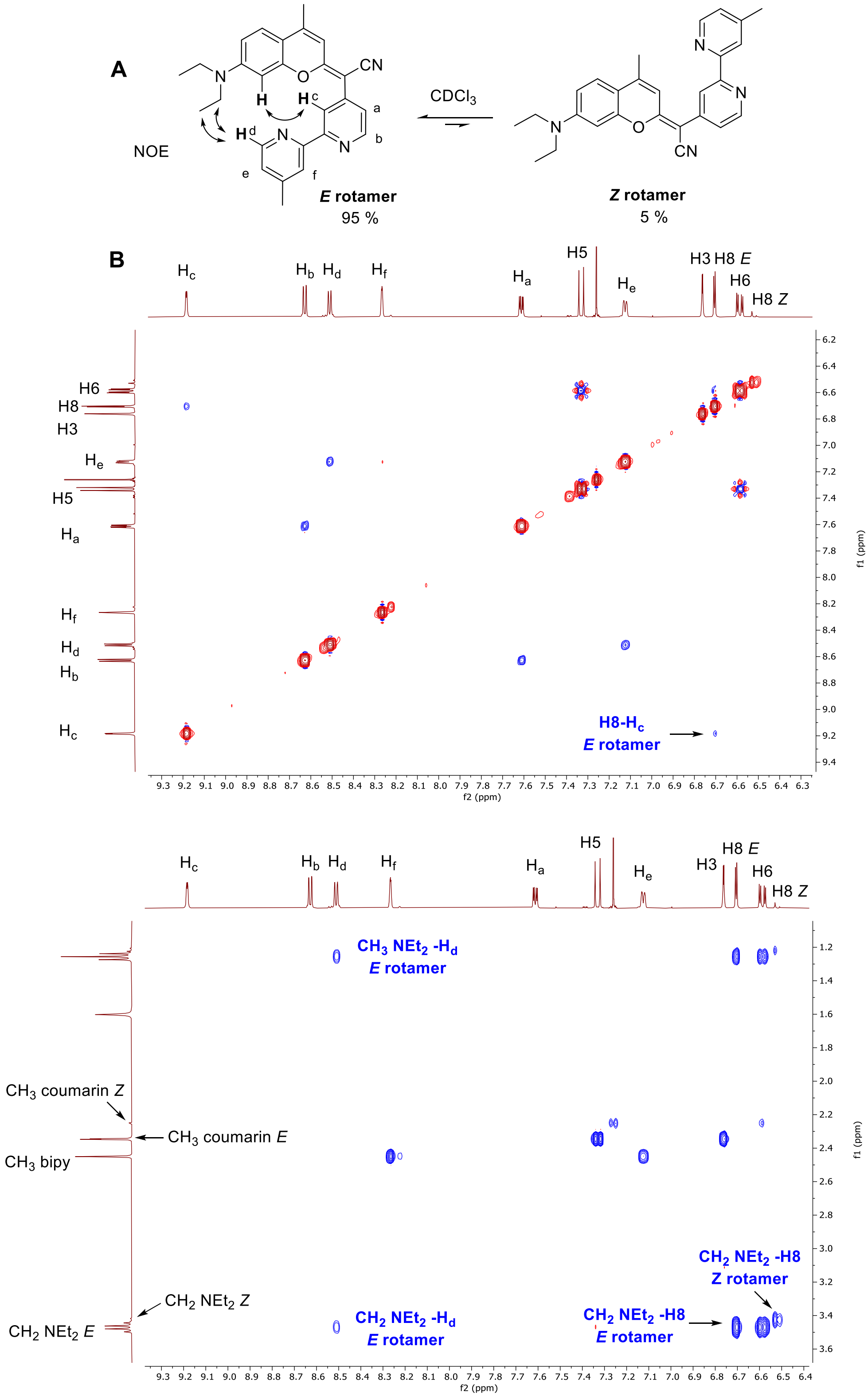

Figure S20. (A) Structures of the *E* and *Z* rotamers of COUBPY ligand **1**. (B) Expansion of the 2D NOESY spectrum (t_m = 500 ms, 25 °C) of **1** in $CDCl_3$ showing NOE cross-peaks.

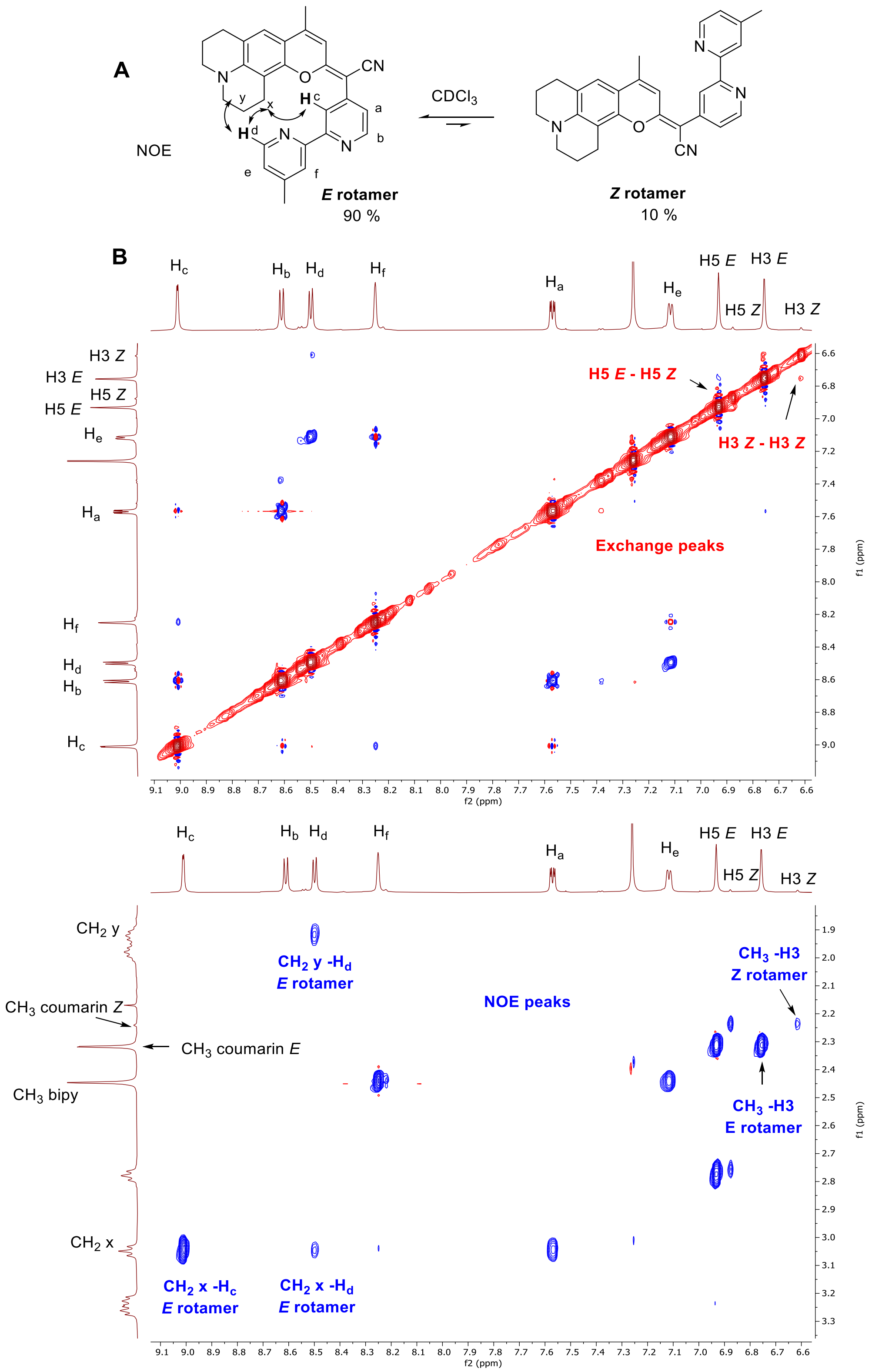

Figure S21. (A) Structures of the *E* and *Z* rotamers of COUBPY ligand **2**. (B) Expansion of the 2D NOESY spectrum (t_m = 500 ms, 25 °C) of **2** in $CDCl_3$ showing NOE cross-peaks and exchange cross-peaks between rotamer resonances of the same sign as the diagonal.

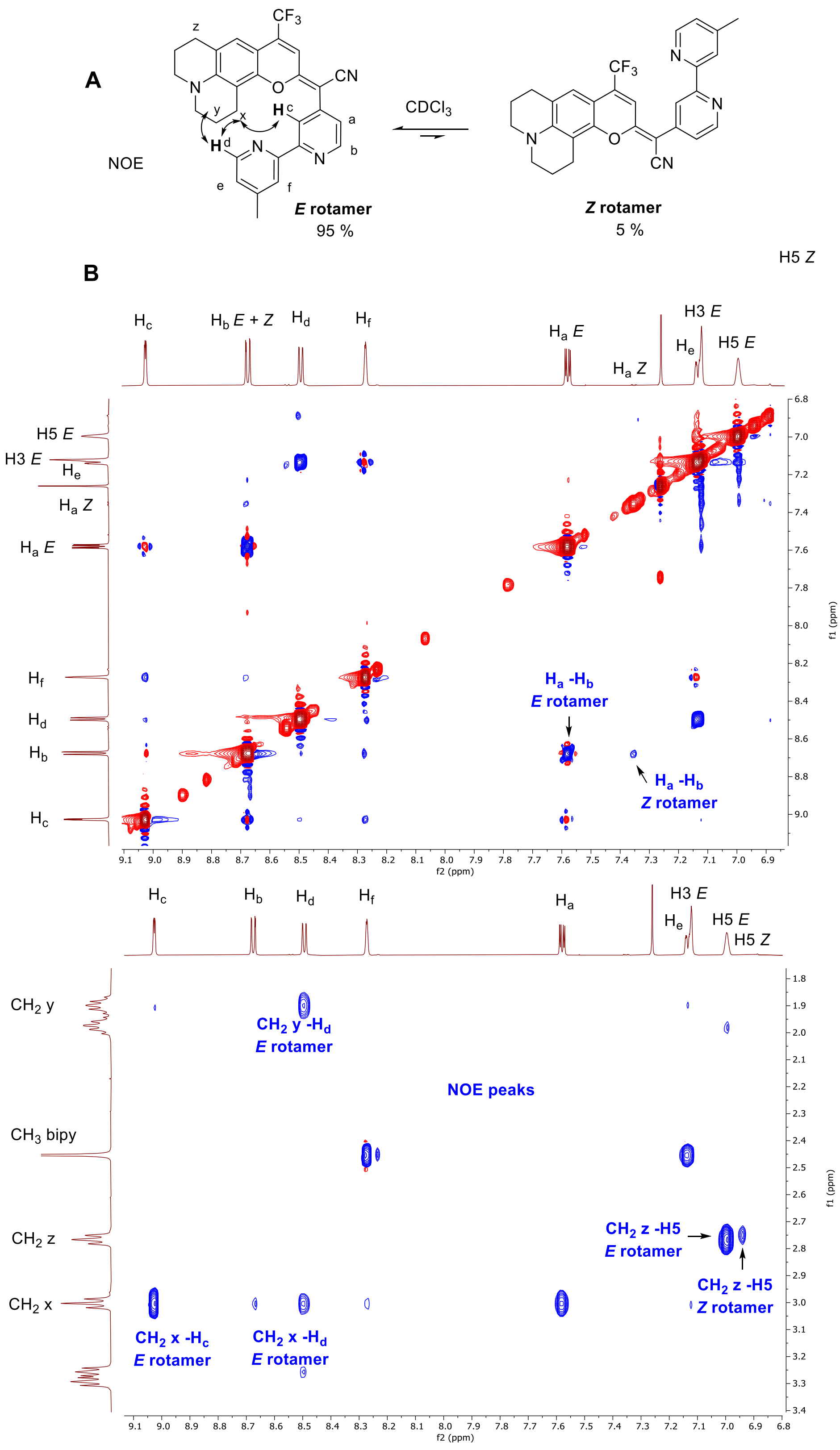

Figure S22. (A) Structures of the *E* and *Z* rotamers of COUBPY ligand **3**. (B) Expansion of the 2D NOESY spectrum (t_m = 500 ms, 25 °C) of **3** in $CDCl_3$ showing NOE cross-peaks.

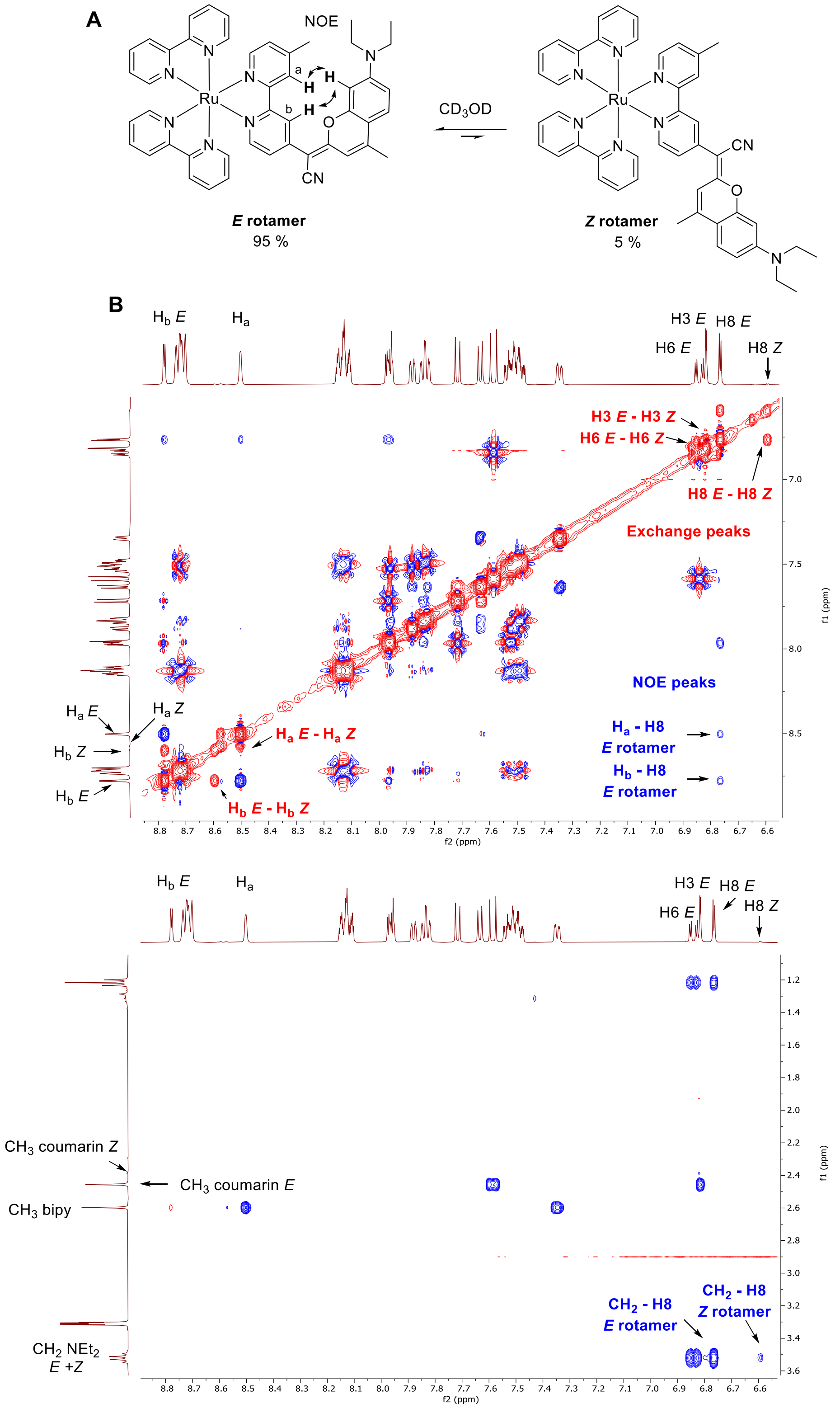

Figure S23. (A) Structures of the *E* and *Z* rotamers of Ru-COUBPY complex **SCV42**. (B) Expansion of the 2D NOESY spectrum (t_m = 500 ms, 25 °C) of **SCV42** in CD_3OD showing NOE cross-peaks and exchange cross-peaks between rotamer resonances of the same sign as the diagonal.

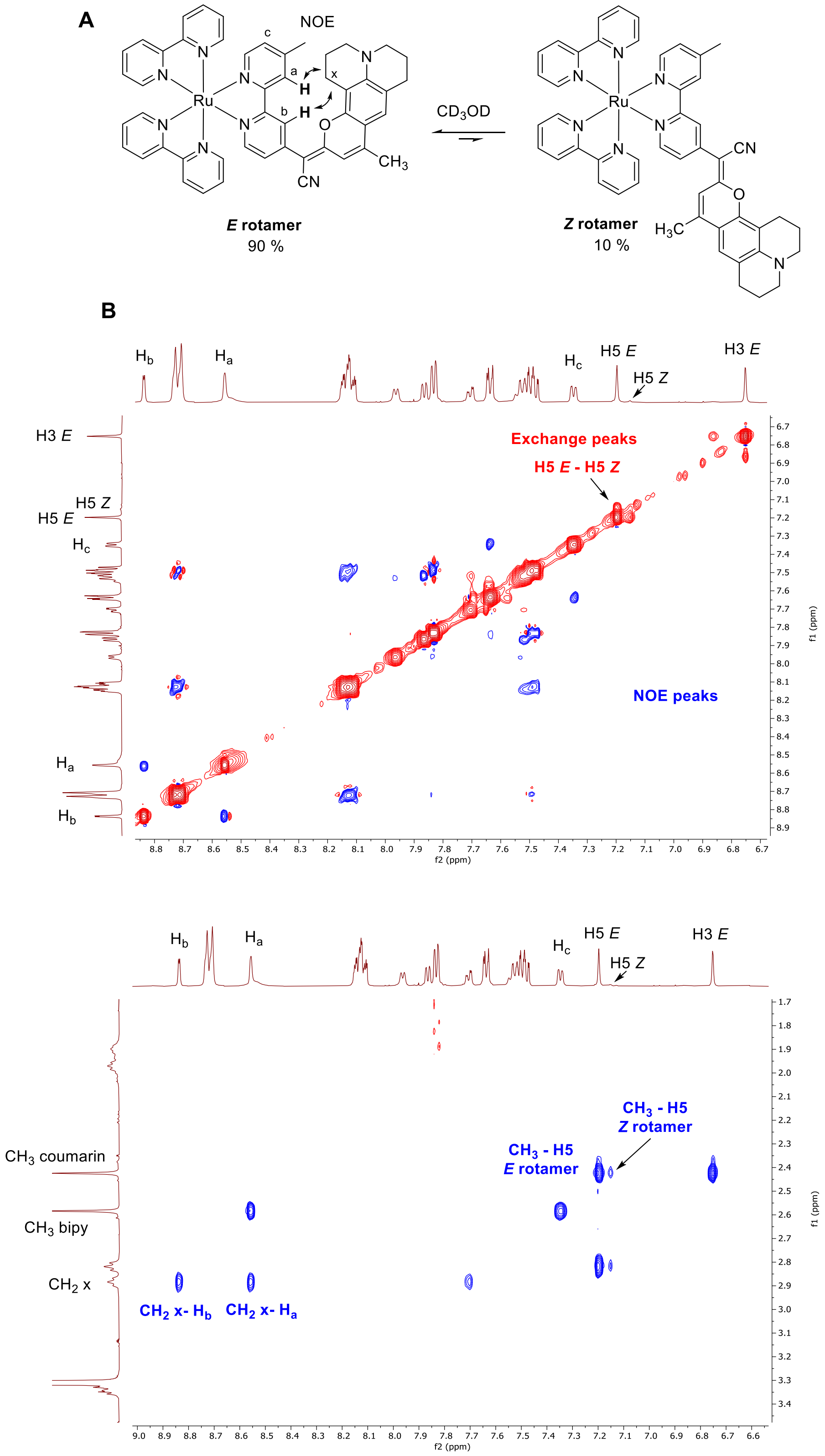

Figure S24. (A) Structures of the *E* and *Z* rotamers of Ru-COUBPY complex **SCV45**. (B) Expansion of the 2D NOESY spectrum (t_m = 500 ms, 25 °C) of **SCV45** in CD_3OD showing NOE cross-peaks and exchange cross-peaks between rotamer resonances of the same sign as the diagonal.

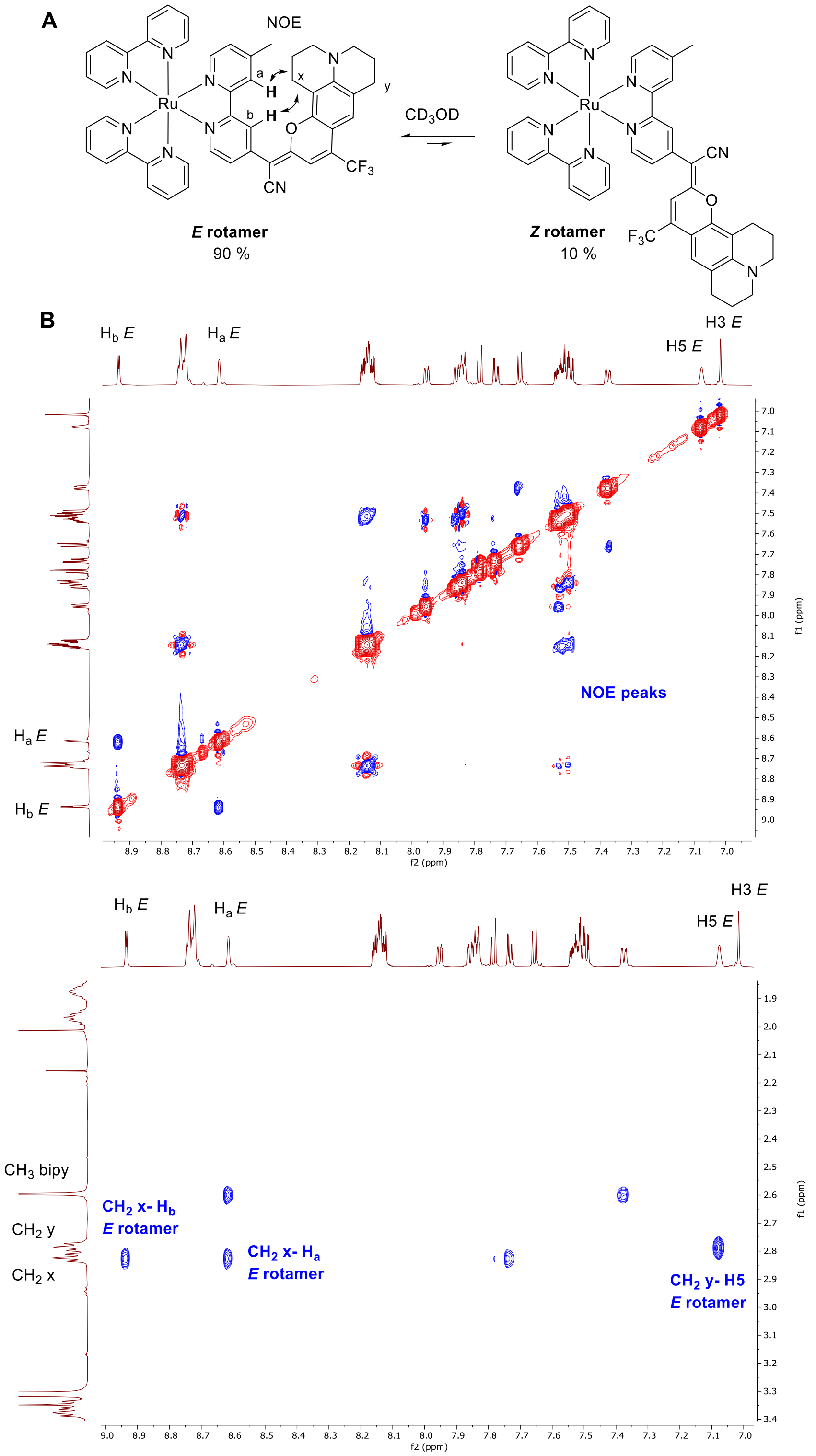

Figure S25. (A) Structures of the *E* and *Z* rotamers of Ru-COUBPY complex **SCV49**. (B) Expansion of the 2D NOESY spectrum (t_m = 500 ms, 25 °C) of **SCV49** in CD_3OD showing NOE cross-peaks.

4.- Photophysical characterization: experimental and computational studies

4.1.- Spectroscopic studies

The photophysical properties of the compounds were investigated in CH_3CN at room temperature and compared with those of the complex $[Ru(bpy)_3]Cl_2$ which was chosen as reference. Absorption spectra were recorded in a Varian Cary 500 UV/Vis/NIR at room temperature. Molar absorption coefficients (ε) were determined by direct application of the Beer-Lambert law, using solutions of the compounds in each solvent with concentrations ranging from 10^{-6} to 10^{-5} M. Emission spectra were registered in a Photon Technology International (PTI) fluorimeter. Luminescence quantum yields (Φ_F) were measured by comparative method using cresyl violet in ethanol (CV; $\Phi_{F;Ref}$ = 0.54 ± 0.03) as reference.[5] Then, optically-matched solutions of the compounds and CV were excited and the fluorescence spectra were recorded. The absorbance of sample and reference solutions was set below 0.1 at the excitation wavelength and Φ_F were calculated using the following equation (1):

$$\Phi_{F;Sample} = \frac{Area_{Sample}}{Area_{Ref}} x \left(\frac{\eta_{Sample}}{\eta_{Ref}}\right)^2 x\, \Phi_{F;ref} \quad (1)$$

where $Area_{Sample}$ and $Area_{Ref}$ are the integrated fluorescence for the sample and the reference and η_{Sample} and η_{Ref} are the refractive index of sample and reference solutions respectively. The uncertainty in the experimental value of Φ_F has been estimated to be approximately 10%.

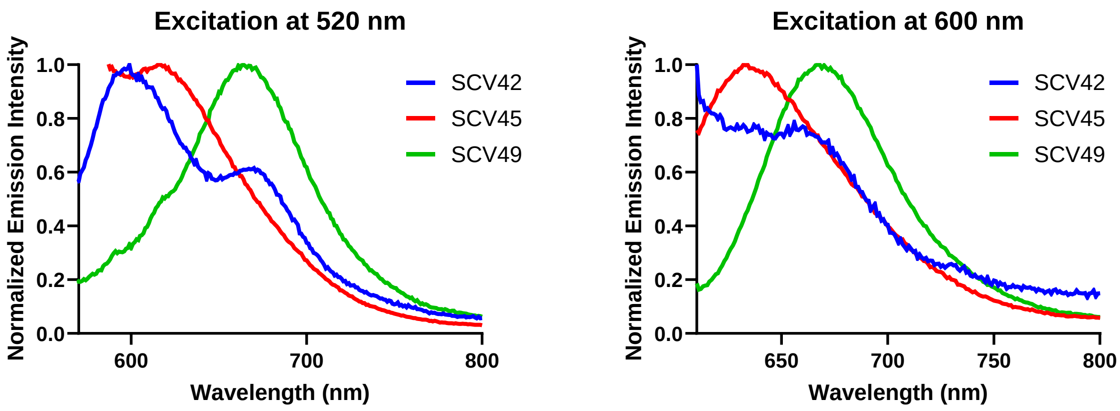

Figure S26. Normalized emission spectra of Ru-COUBPY complexes upon excitation at 520 or 600 nm.

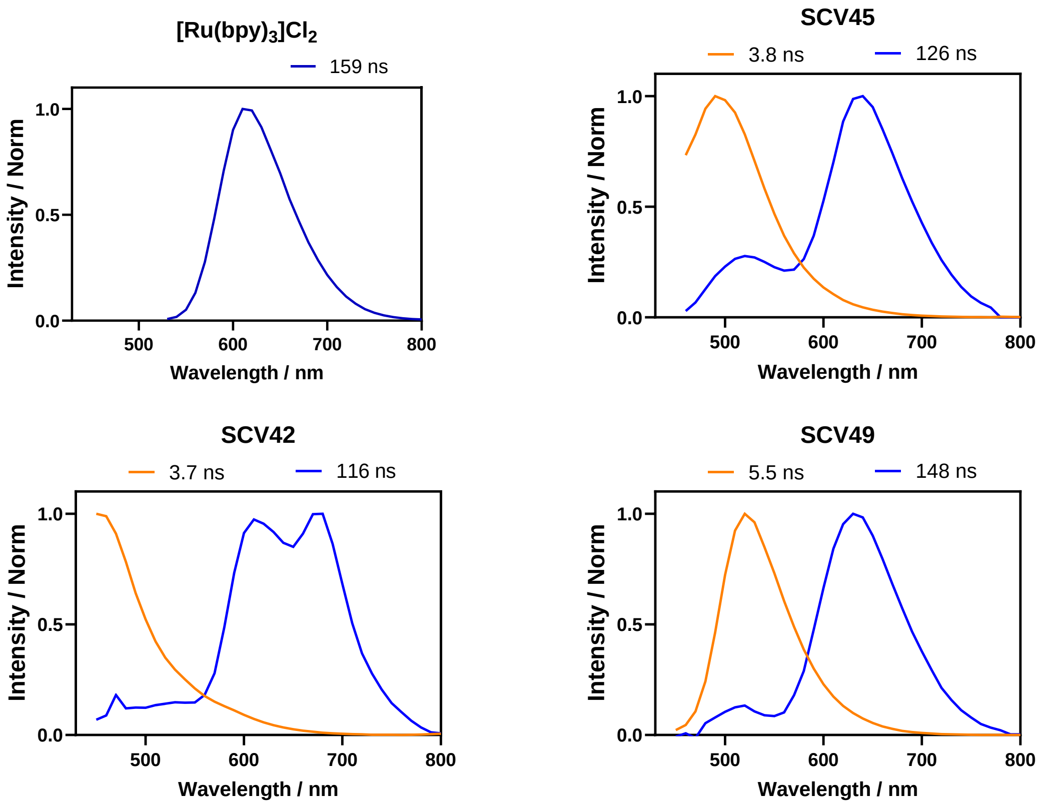

Figure S27. Time-Resolved Emission Spectra of air-saturated acetonitrile solutions of Ru-COUBPY complexes upon pulsed laser excitation at 405 nm. $[Ru(bpy)_3]Cl_2$ has been included as reference.

4.2. Computational studies

Ground-state optimizations were performed at the DFT/PBE0[6] level of theory, describing all atoms with the 6-31+G(d,p) basis set except Ru, which was treated with the SDD pseudopotential (hereafter, PBE0/6-31+G(d,p)/SDD method).[7] Singlet and triplet excited-state vertical determinations and geometry optimizations were calculated with the time-dependent (TD)-DFT ansatz,[8] in particular, with the M06/6-31+G(d,p)/SDD level of theory,[9] using the Tamm-Dancoff approximation throughout.[10] Solvent effects (ACN) were described with the conductor-like polarizable continuum model (CPCM).[11] This computational protocol has been successfully applied to characterize the structure and excited-state properties of related Ru(II) complexes.[12,13,14] All DFT and TD-DFT calculations were conducted with the Gaussian 16 quantum chemistry software.[15] The nature of the transitions was unambiguously characterized by computing the natural transition orbitals (NTOs)[16,17,18] with the Chemissian program[19] through the post-processing the corresponding Gaussian 16 outputs. Quantitative wave function analyses, including local and charge transfer descriptors, were calculated with the TheoDORE 3.1.1 software.[20] Atoms of the **SCV** molecules were divided into two fragments (1: Ru(II) complex and 2:**COUBPY**), and the descriptors were defined as local (1→1, 2→2) or charge transfer (1→2, 2→1), as indicated in the corresponding figures.

Table S1. TD-M06/6-31+G(d,p)/SDD absorption energies (ΔE_{abs}) and wavelengths (λ_{abs}), oscillator strengths (*f*), and nature of the states for **SCV42**. Experimental band maxima energies (ΔE_{exp}) and wavelengths (λ_{exp}) in ACN are also shown and ascribed to theoretical determinations. All energies in eV and λ in nm.

State	Nature	ΔE_{abs}	λ_{abs}	f	ΔE_{exp}	λ_{exp}
S_1	MLCT	2.38	522	0.0039	~2.23	~555
S_2	MLCT	2.39	519	0.0321		
S_3	MLCT/IL$_{cou}$	2.44	507	0.2846	~2.38	~520
S_4	MLCT	2.56	485	0.0252		
S_5	MLCT	2.57	482	0.1199		
S_6	MLCT/IL$_{cou}$	2.64	469	0.3660		
S_7	MLCT	2.73	454	0.1423	~2.70	~459
S_8	MLCT	2.78	447	0.0864		
S_9	IL$_{cou}$	2.90	428	0.1536		

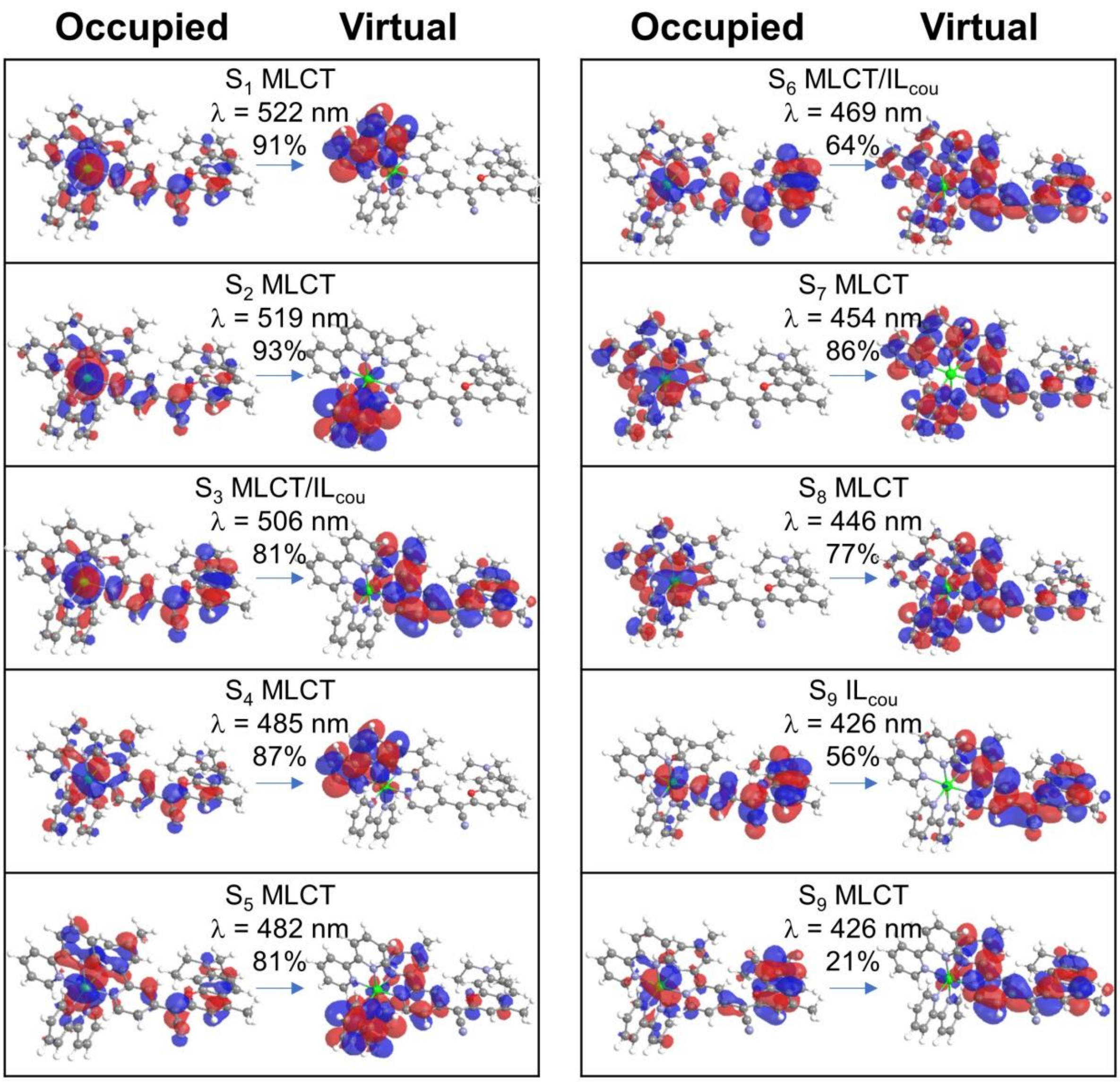

Figure S28. Transition natural orbitals (NTOs) for absorptions λ_{abs}>420 nm for **SCV42**.

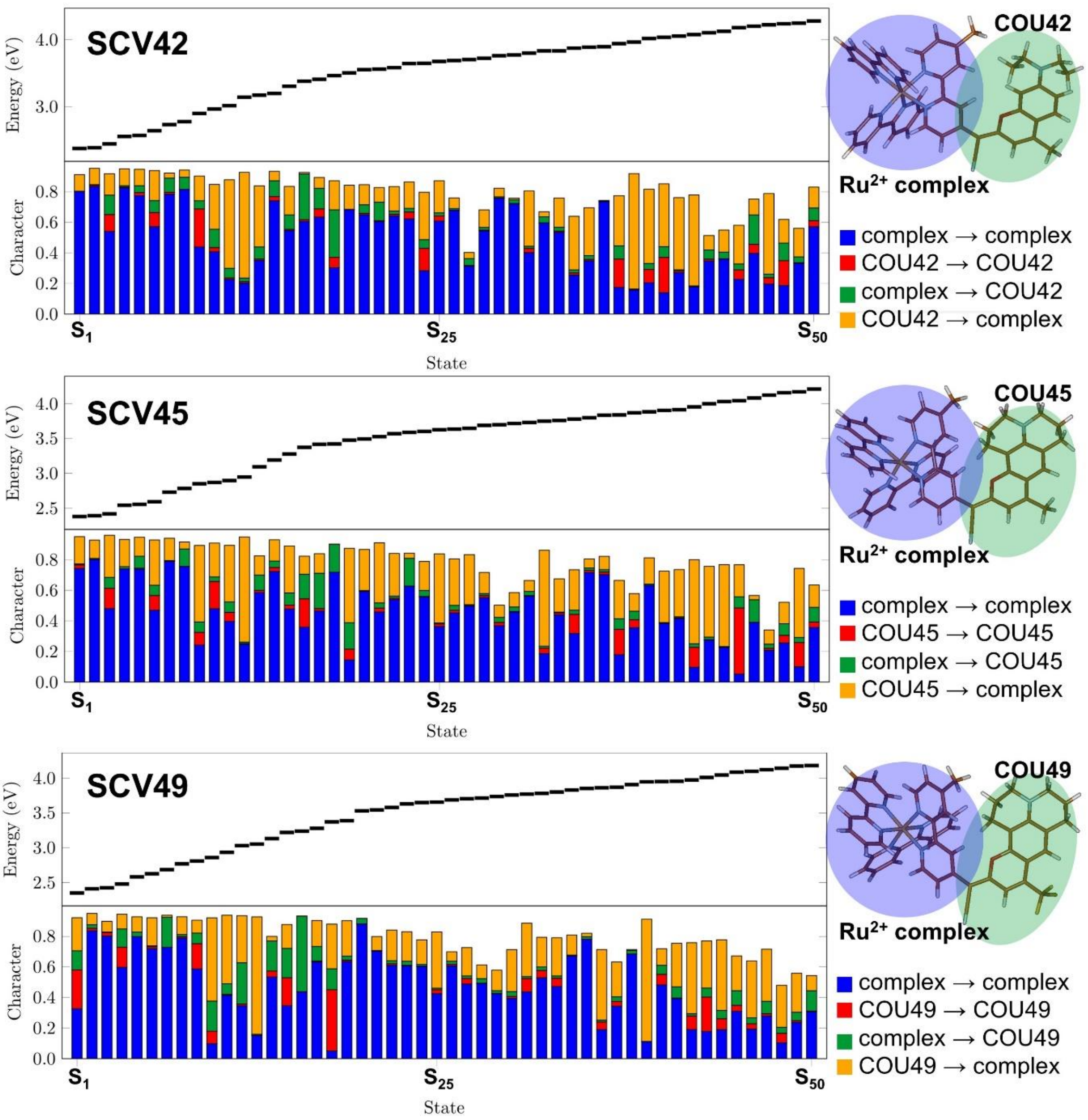

Figure S29. Molecular topology of the singlet excited states at the ground-state geometry of each Ru-COUBPY complex. TD-M06/6-31+G(d,p)/SDD spectrum in ACN, Gaussian output post-processing conducted with the TheoDORE 3.1.1 program.[20]

Table S2. TD-M06/6-31+G(d,p)/SDD absorption energies (ΔE_{abs}) and wavelengths (λ_{abs}), oscillator strengths (*f*), and nature of the states for **SCV45**. Experimental band maxima energies (ΔE_{exp}) and wavelengths (λ_{exp}) in ACN are also shown and ascribed to theoretical determinations. All energies in eV and λ in nm.

State	Nature	ΔE_{abs}	λ_{abs}	f	ΔE_{exp}	λ_{exp}
S_1	MLCT/LLCT	2.38	521	0.0492	2.20	564
S_2	MLCT	2.39	519	0.0033		
S_3	MLCT/IL_{cou}	2.42	513	0.3026	2.41	515
S_4	MLCT/LLCT	2.54	488	0.0014		
S_5	MLCT	2.55	485	0.0589		
S_6	MLCT/IL_{cou}	2.59	479	0.1944	2.67	460
S_7	MLCT	2.73	454	0.1149		
S_8	MLCT	2.78	446	0.0131		
S_9	LLCT	2.85	435	0.1135		
S_{10}	MLCT/LLCT	2.87	432	0.1012		
S_{11}	LLCT	2.90	428	0.1791		
S_{12}	LLCT	2.95	421	0.0142		

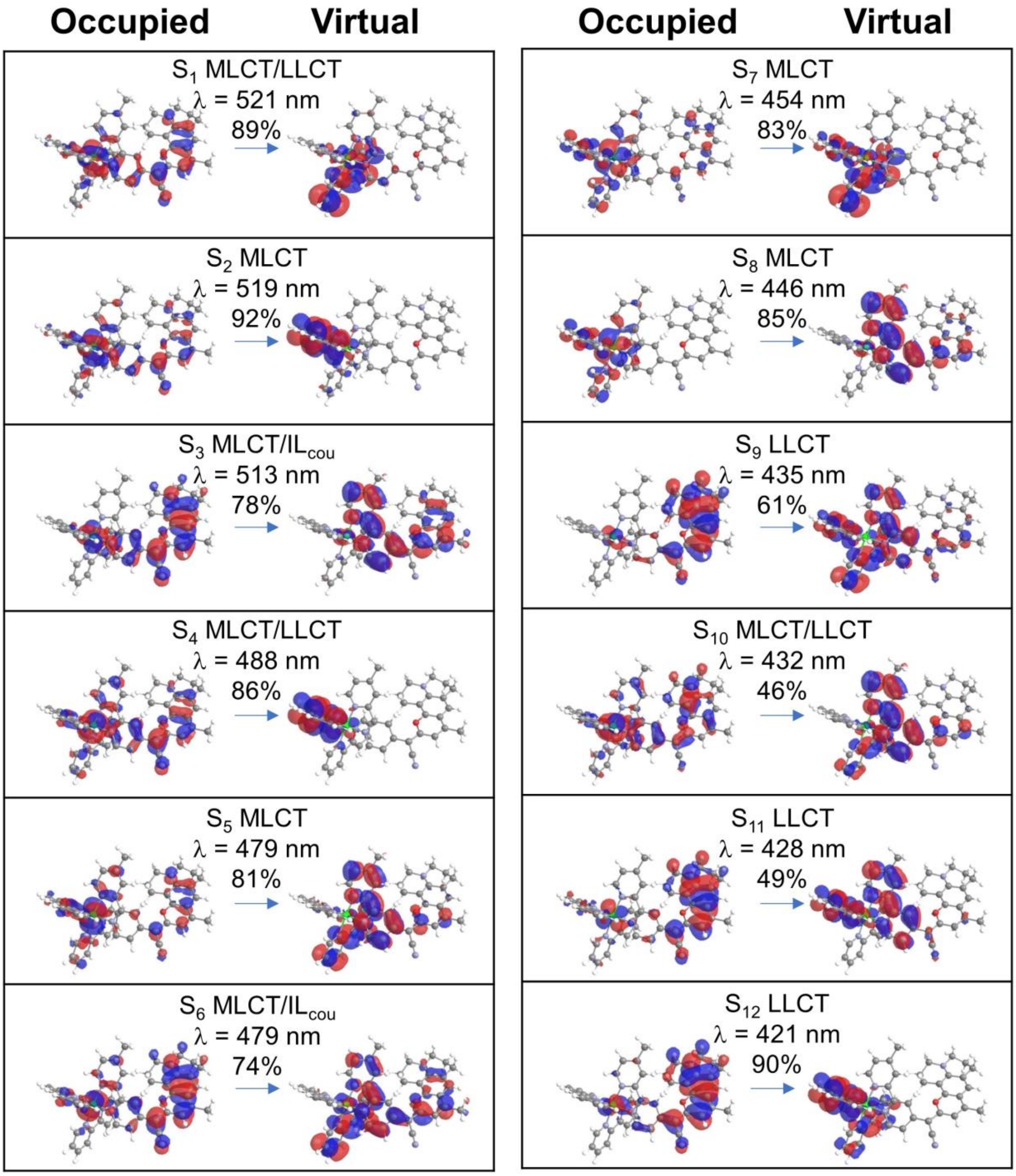

Figure S30. Transition natural orbitals (NTOs) for absorptions λ_{abs}>420 nm for **SCV45**.

Table S3. TD-M06/6-31+G(d,p)/SDD absorption energies (ΔE_{abs}) and wavelengths (λ_{abs}), oscillator strengths (*f*), and nature of the states for **SCV49**. Experimental band maxima energies (ΔE_{exp}) and wavelengths (λ_{exp}) in ACN are also shown and ascribed to theoretical determinations. All energies in eV and λ in nm.

State	Nature	ΔE_{abs}	λ_{abs}	*f*	ΔE_{exp}	λ_{exp}
S_1	MLCT/IL_{cou}	2.35	527	0.3915	2.17	571
S_2	MLCT	2.41	514	0.0266		
S_3	MLCT	2.43	511	0.0408		
S_4	MLCT/IL_{cou}	2.48	500	0.2174	2.17	571
S_5	MLCT	2.58	480	0.0045		
S_6	MLCT/LLCT	2.63	472	0.0412		
S_7	MLCT	2.69	461	0.0138		
S_8	MLCT	2.77	448	0.1338	2.69	461
S_9	MLCT/IL_{cou}	2.81	441	0.1443	2.69	461
S_{10}	LLCT	2.86	434	0.0872		
S_{11}	MLCT/IL_{cou}	2.94	422	0.0457		

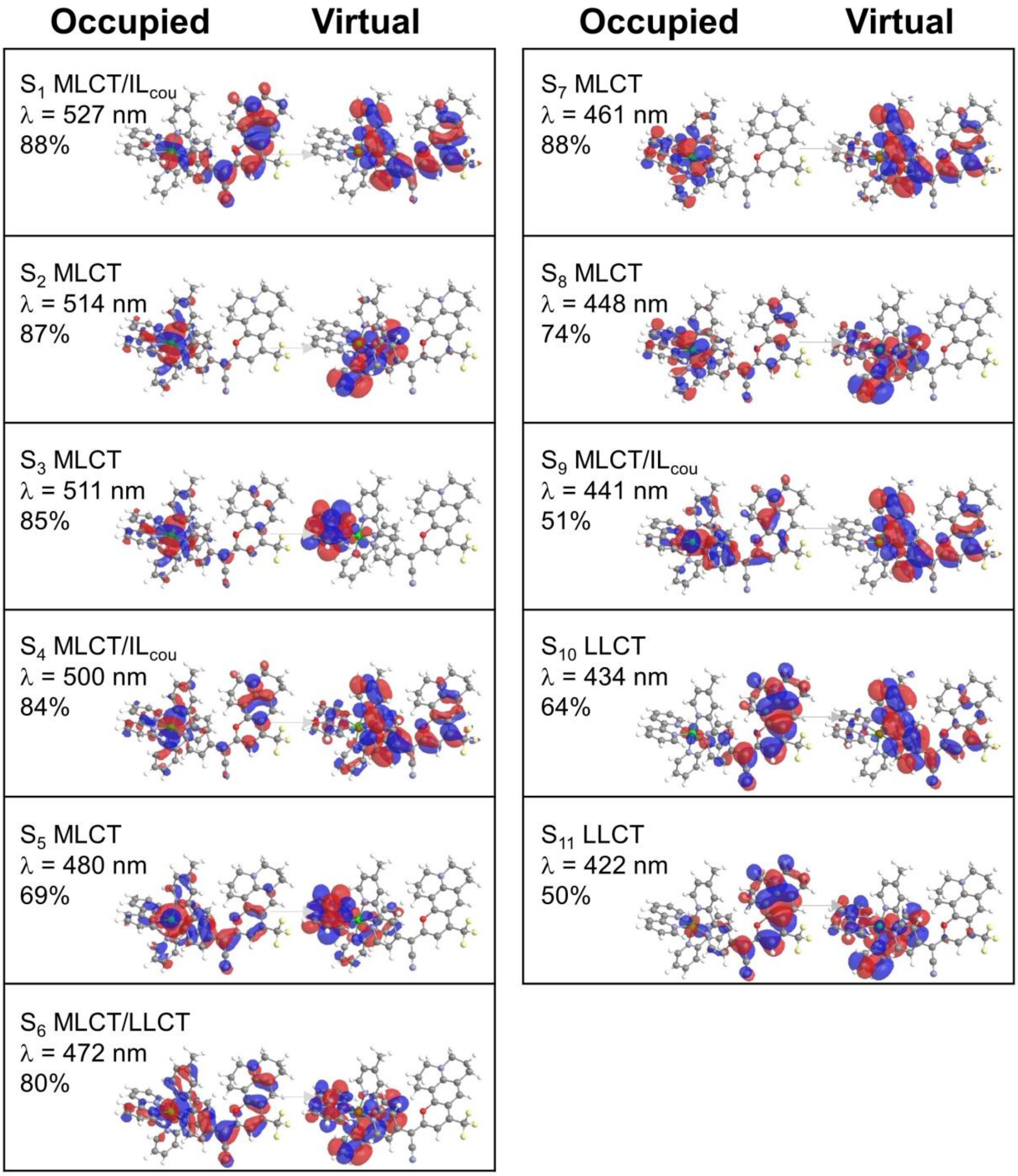

Figure S31. Transition natural orbitals (NTOs) for absorptions λ_{abs}>420 nm for **SCV49**.

5.- Dark and light stability studies in cell culture medium

5.1. Dark stability

For the dark stability studies, a solution of the compounds (30 µM) in DMEM culture medium supplemented with 10% FBS, 2 mM L-glutamine and 100 U·mL^{-1} of penicillin-streptomycin mixture, and containing 2,3,6,7-tetrahydro-1,1,7,7,9-pentamethyl-1*H*,5*H*,11*H*[1]benzopyrano-[6,7,8-*ij*]quinolizin-11-one (30 µM) as an internal standard, was incubated at 37 °C for 24 h. A 60 µL aliquot of the previous solutions was taken after the indicated time intervals (0, 1, 5 and 24 h) and analyzed by reversed-phase HPLC. The extent of degradation of the different compounds was evaluated by comparing the ratio between the peak area of investigated compound and that of the internal standard, before and after incubation for the indicated time interval.

The HPLC analysis was performed with a Waters alliance 2695 Separations Module, comprised of a quaternary pump solvent delivery module, online degasser, auto sampler and a Waters 2996 photodiode array detector. HPLC separation was carried out using a Jupiter Proteo C12 column (150 x 4.6 mm, 90 Å, 4 µm) from Phenomenex. The mobile phase was a linear gradient beginning with 70:30 (v/v) A/B and ending with 0:100 (v/v) A/B over 15 min at a flow rate of 1 mL/min (A: 0.05% TFA in H_2O; B: 0.05% TFA in ACN). The injection volume was 50 µL. Control of the HPLC instrument, as well as processing of the chromatogram output (annotation of retention times, integration of peaks, calculation of peak areas) was carried out with MassLynx V4.1 software.

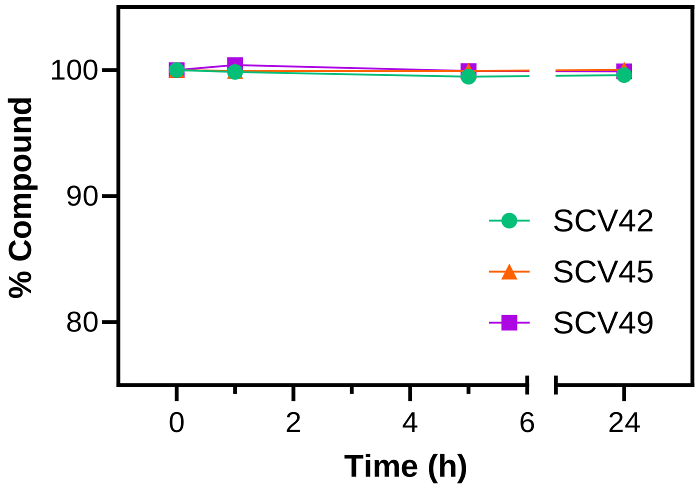

Figure S32. Stability of **SCV42**, **SCV45** and **SCV49** in complete cell culture medium at t = 0 and after incubation in the dark at 37 ºC for 1 h, 5 h and 24 h.

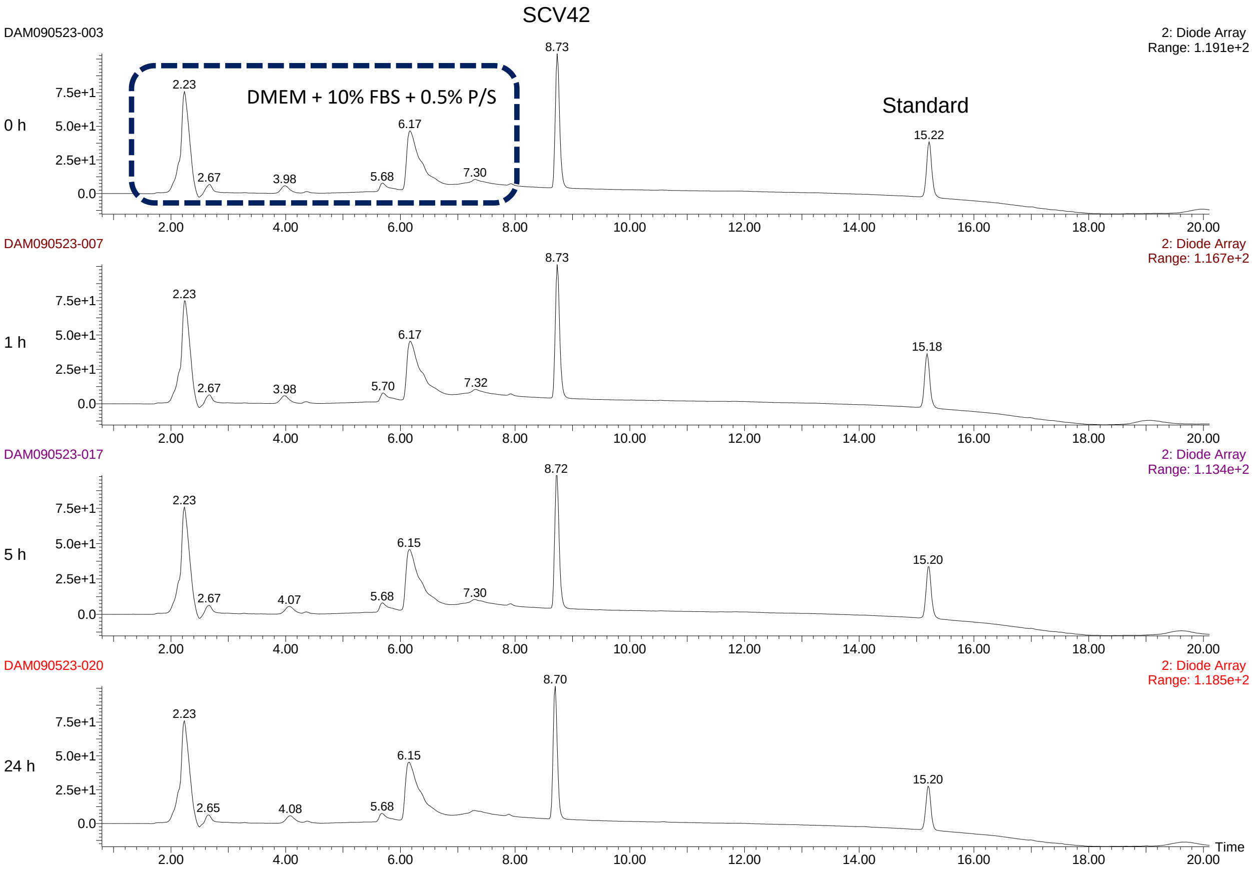

Figure S33. HPLC chromatograms showing the stability of **SCV42** in cell culture medium in the dark at 37 ºC after 0 h, 1 h, 5 h and 24 h.

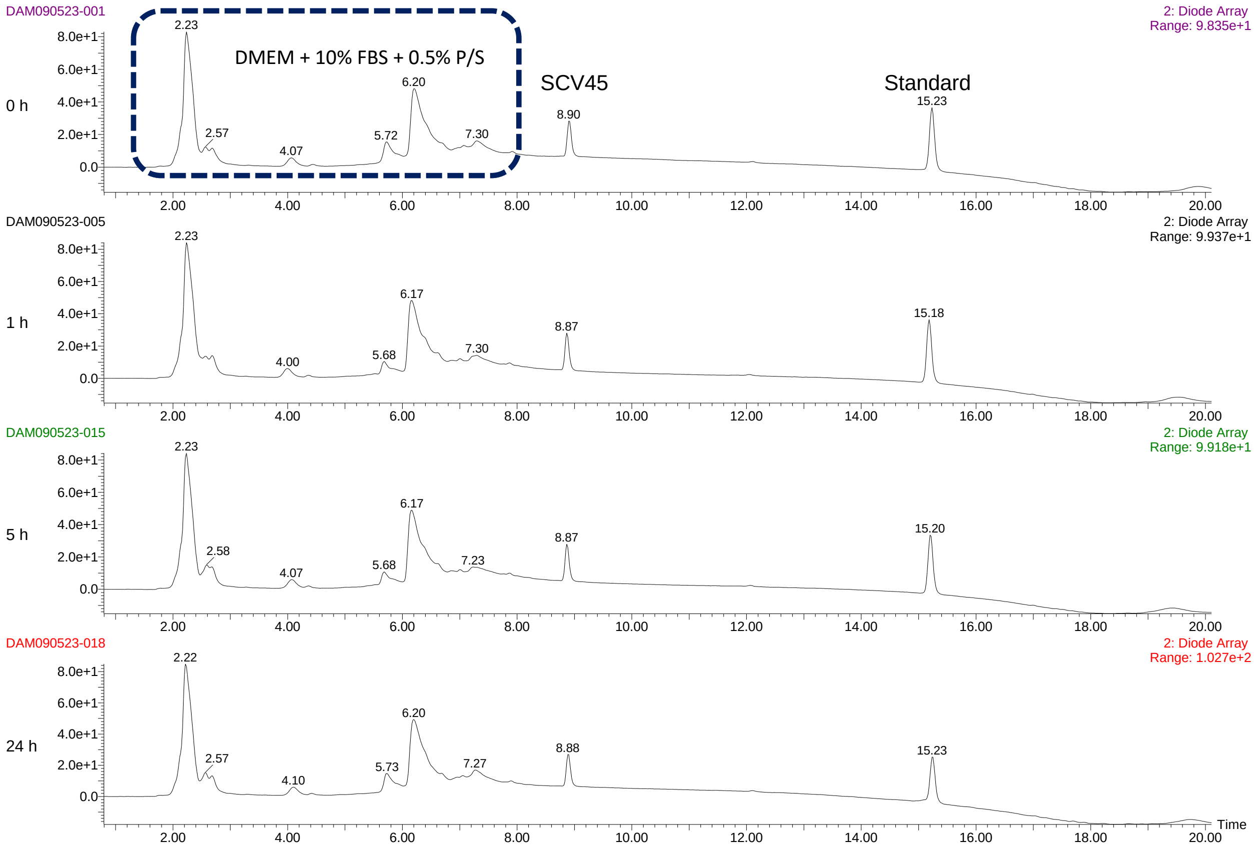

Figure S34. HPLC chromatograms showing the stability of **SCV45** in cell culture medium in the dark at 37 ºC after 0 h, 1 h, 5 h and 24 h.

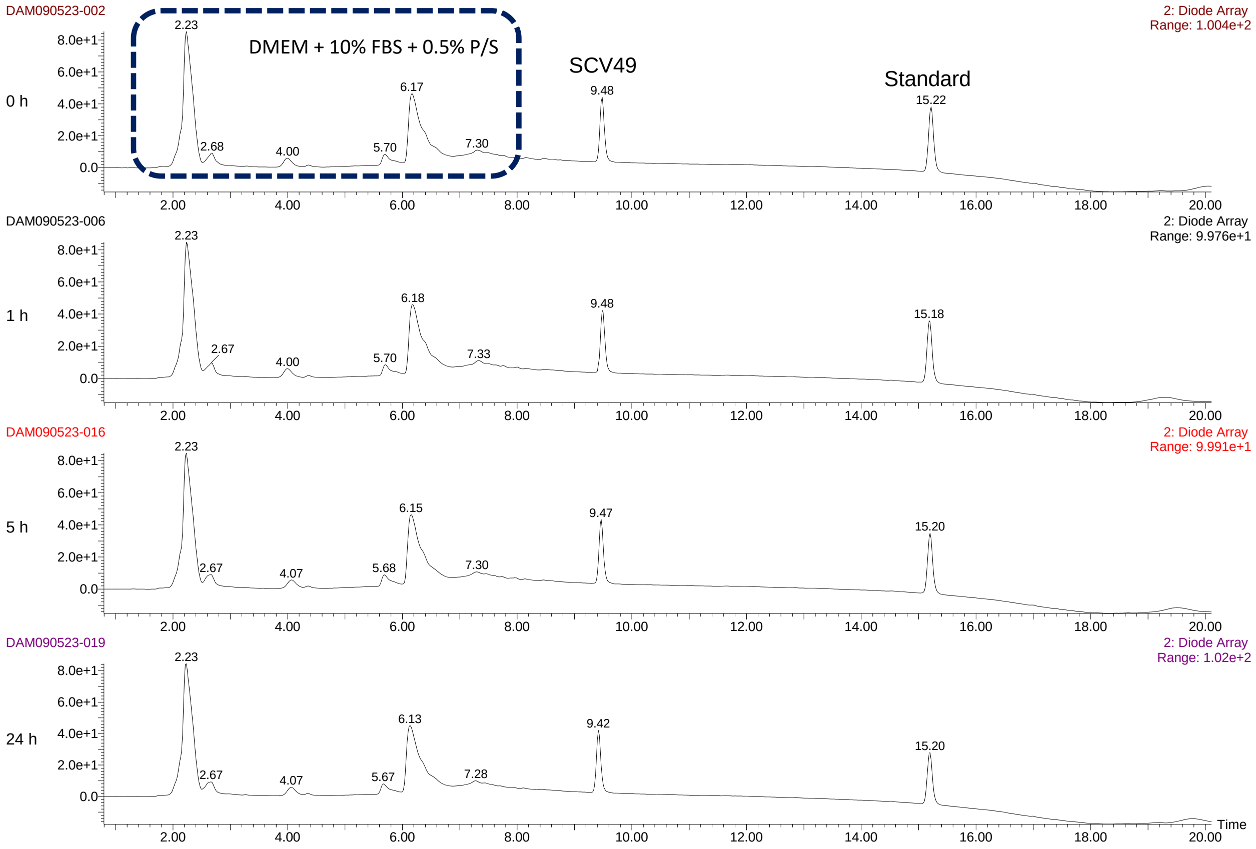

Figure S35. HPLC chromatograms showing the stability of **SCV49** in cell culture medium in the dark at 37 ºC after 0 h, 1 h, 5 h and 24 h.

5.2. Photostability

For the photostability studies, a solution of the compounds (20 μM) in DMEM culture medium supplemented with 10% FBS, 2 mM L-glutamine and 100 U·mL^{-1} of penicillin-streptomycin mixture, and containing 2,3,6,7-tetrahydro-1,1,7,7,9-pentamethyl-1*H*,5*H*,11*H*[1]benzopyrano-[6,7,8-*ij*]quinolizin-11-one (20 μM) as an internal standard, was irradiated with green ((505±35 nm, 100 mW cm^{-2}) or red light (620±15 nm; 130 mW cm^{-2}) LED light for 2 h at 37 ºC. A 60 μL aliquot of the previous solution was taken at t = 0 and after irradiation for the indicated time intervals for each compound (15, 30, 45, 60, 90 and 120 min or 0, 5, 10, 15, 20, 30, 45 min) and analyzed by reversed-phase HPLC. The extent of photodegradation of the PS was evaluated by comparing the ratio between the peak area of the compounds and that of the internal standard, before and after irradiation for the indicated time intervals.

The HPLC analysis was performed with a Waters alliance 2695 Separations Module, comprised of a quaternary pump solvent delivery module, online degasser, auto sampler and a Waters 2996 photodiode array detector. HPLC separation was carried out using a Jupiter Proteo C12 column (150 x 4.6 mm, 90 Å, 4 μm) from Phenomenex. The mobile phase was a linear gradient beginning with 70:30 (v/v) A/B and ending with 0:100 (v/v) A/B over 15 min at a flow rate of 1 mL/min (A: 0.05% TFA in H_2O; B: 0.05% TFA in ACN). The injection volume was 50 μL. Control of the HPLC instrument, as well as processing of the chromatogram output (annotation of retention times, integration of peaks, calculation of peak areas) was carried out with MassLynx V4.1 software. Elution traces were obtained at 260 nm or 288 nm.

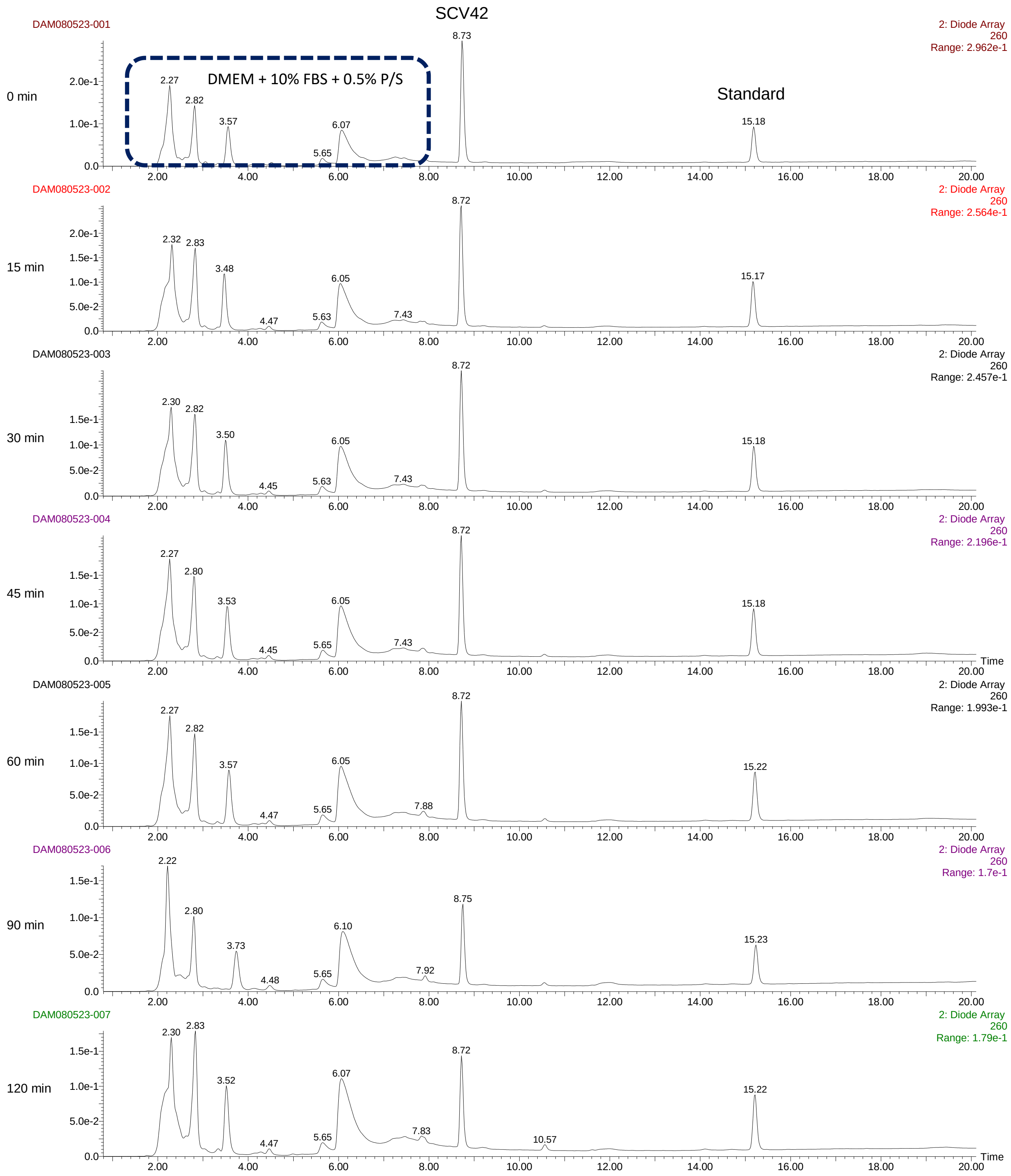

Figure S36. Photostability of **SCV42** in supplemented DMEM culture medium upon irradiation with green light. From top to bottom: HPLC chromatograms of **SCV42** + standard in culture medium before and after irradiation with green light for 15 min, 30 min, 45 min, 60 min, 90 min and 120 min at 37 ºC. Elution traces were obtained at 260 nm.

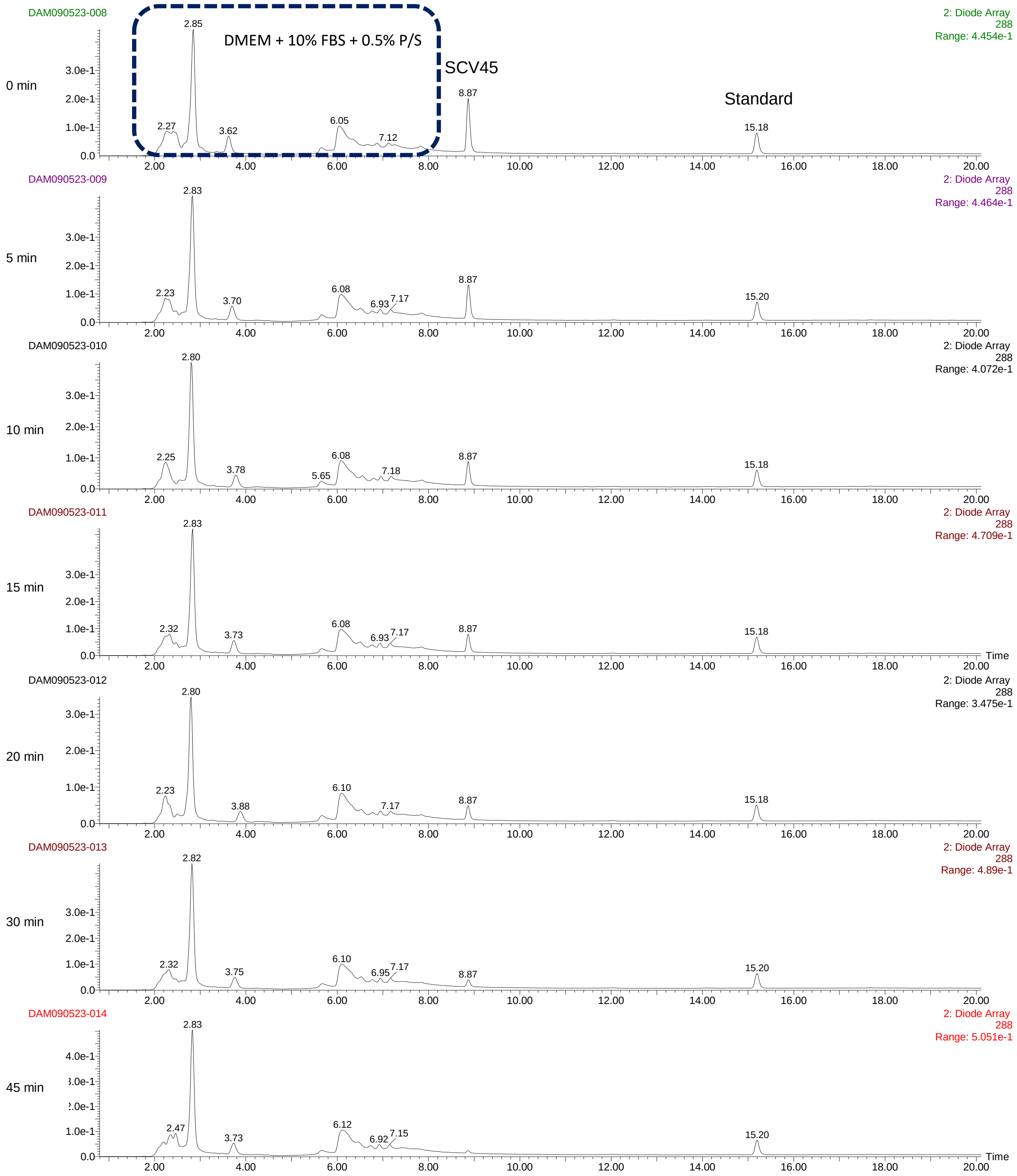

Figure S37. Photostability of **SCV45** in supplemented DMEM culture medium upon irradiation with green light. From top to bottom: HPLC chromatograms of **SCV45** + standard in culture medium before and after irradiation with green light for 5 min, 10 min, 15 min, 20 min, 30 min and 45 min at 37 ºC. Elution traces were obtained at 288 nm.

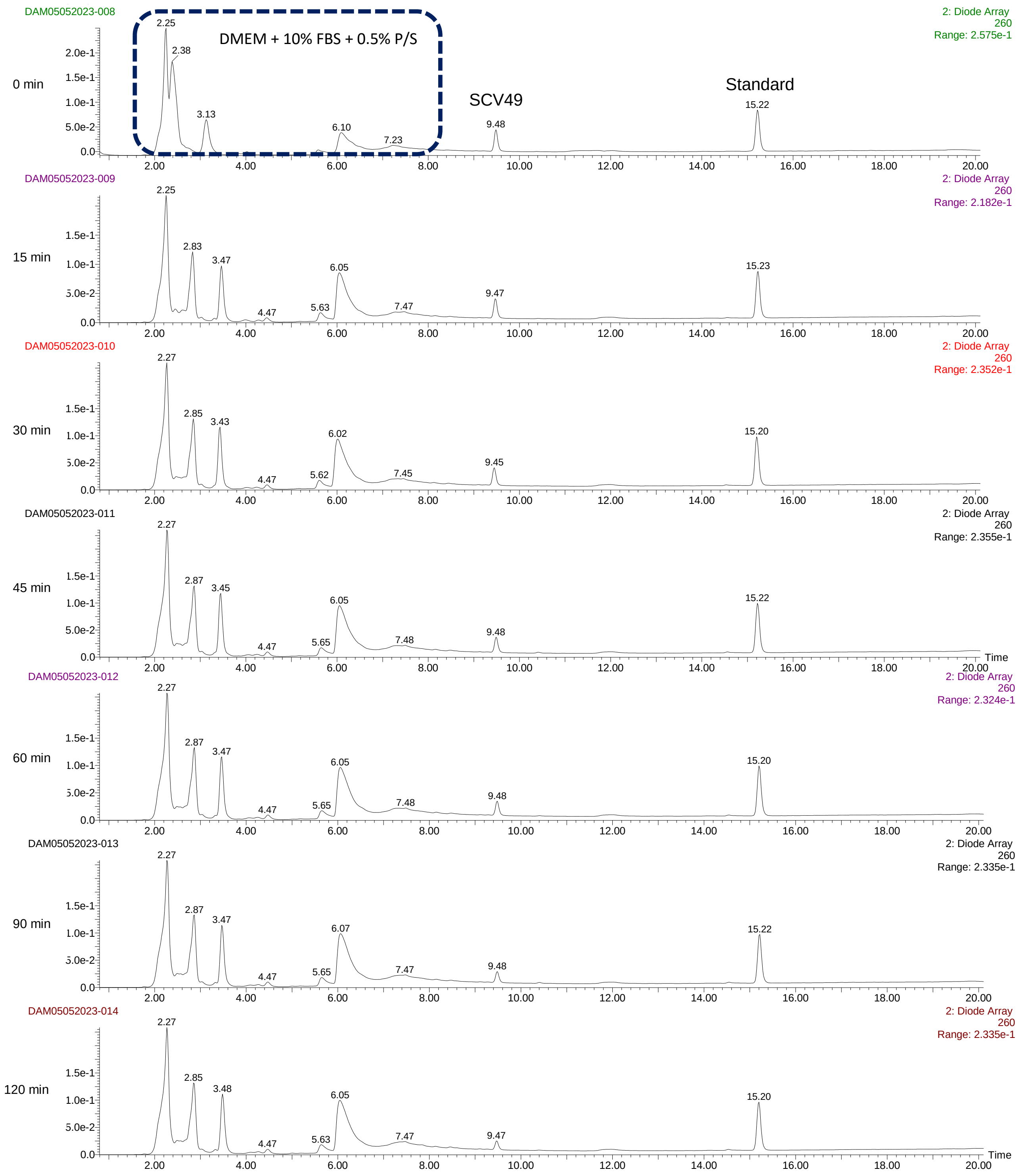

Figure S38. Photostability of **SCV49** in supplemented DMEM culture medium upon irradiation with green light. From top to bottom: HPLC chromatograms of **SCV49** + standard in culture medium before and after irradiation with green light for 15 min, 30 min, 45 min, 60 min, 90 min and 120 min at 37 ºC. Elution traces were obtained at 260 nm.

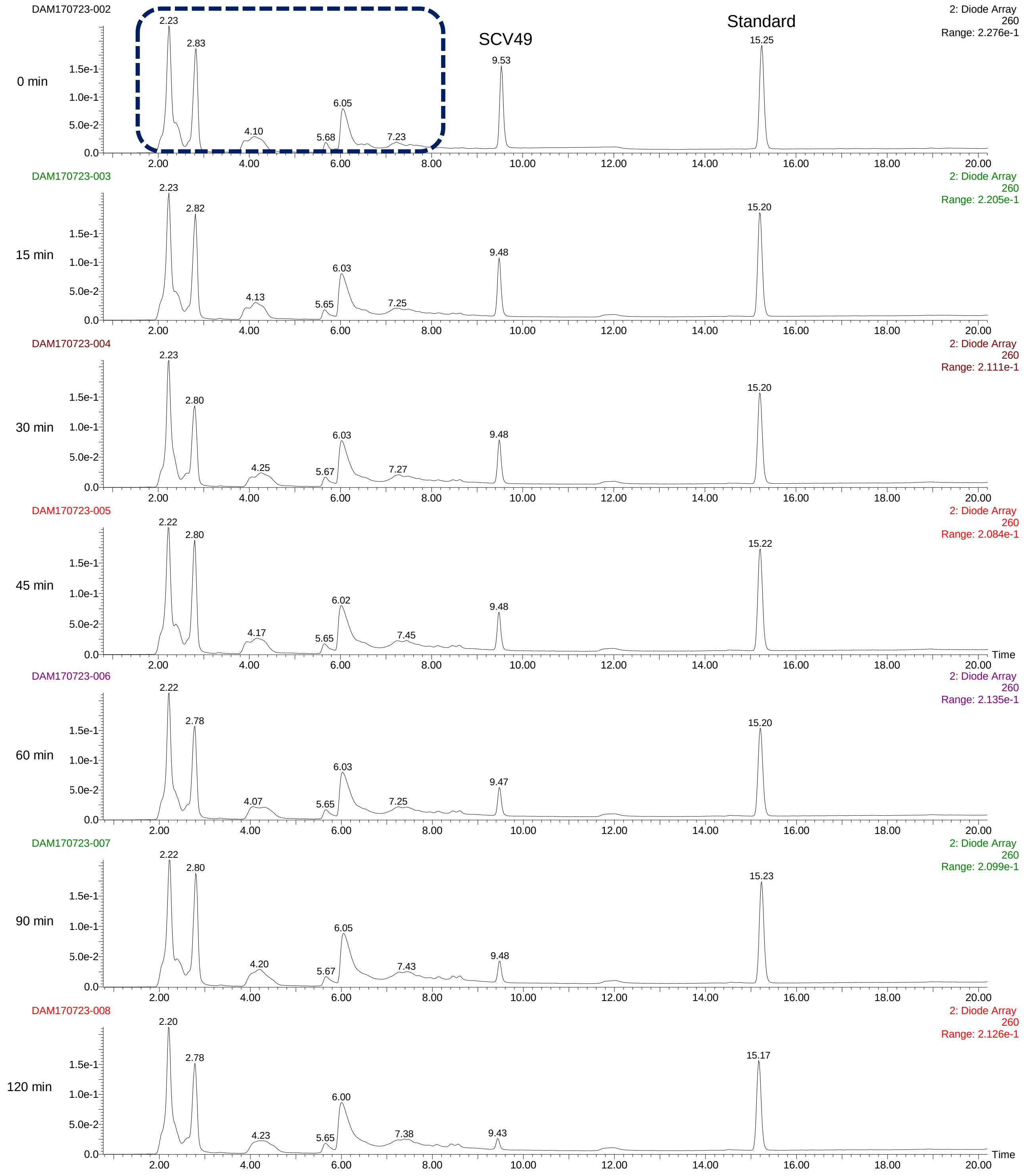

Figure S39. Photostability of **SCV49** in supplemented DMEM culture medium upon irradiation with red light. From top to bottom: HPLC chromatograms of **SCV49** + standard in culture medium before and after irradiation with red light for 15 min, 30 min, 45 min, 60 min, 90 min and 120 min at 37 ºC. Elution traces were obtained at 260 nm.

6.- Photochemical characterization: experimental and computational studies

6.1. Evaluation of singlet oxygen generation using SOSG

All measurements were carried using a Hellma 1.5 mL PTFE-stoppered fluorescence quartz cuvette (4 clear windows) with a 1 cm path length. SOSG (5 µM) was added to a solution of the corresponding studied compound (10 µM) in PBS containing 2% DMSO. The resulting solutions were irradiated with green (505±35 nm, 100 mW·cm^{-2}) or red (620±15 nm, 130 mW·cm^{-2}) LED light for the indicated time intervals (0, 1, 2, 3, 4 and 5 min). Immediately, the fluorescence spectra of the irradiated samples were collected by using a Photon Technology International (PTI) fluorimeter. The excitation wavelength was set to 500 nm, and emission spectra were recorded from 510-600 nm (SOSG: λ_{Ex}=504 nm, λ_{Em}=525 nm). The entrance and exit slits of the excitation and emission monochromators were set at 0.5 mm, giving a spectral bandwidth of 2 nm. The data interval was 1 nm and the integration time was 0.7 sec. Positive control experiments were carried out using Rose Bengal as a reference. Negative control experiments were carried out using sodium azide-saturated PBS as a singlet oxygen scavenger.

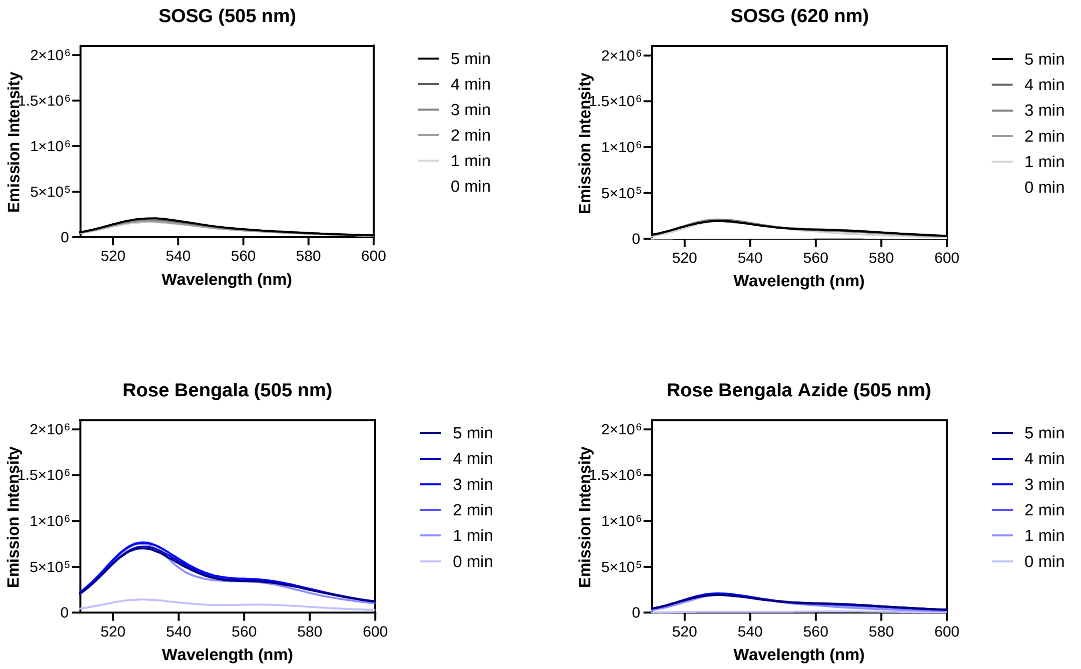

Figure S40. Top: Emission spectra of SOSG upon irradiation with green or red light in PBS (2 % DMSO). Bottom: Photogeneration of singlet oxygen by Rose Bengal. Increase of the fluorescence spectra emission of SOSG upon irradiation of Rose Bengal with green light in PBS (2 % DMSO) alone or in the presence of sodium azide-saturated PBS (2 % DMSO).

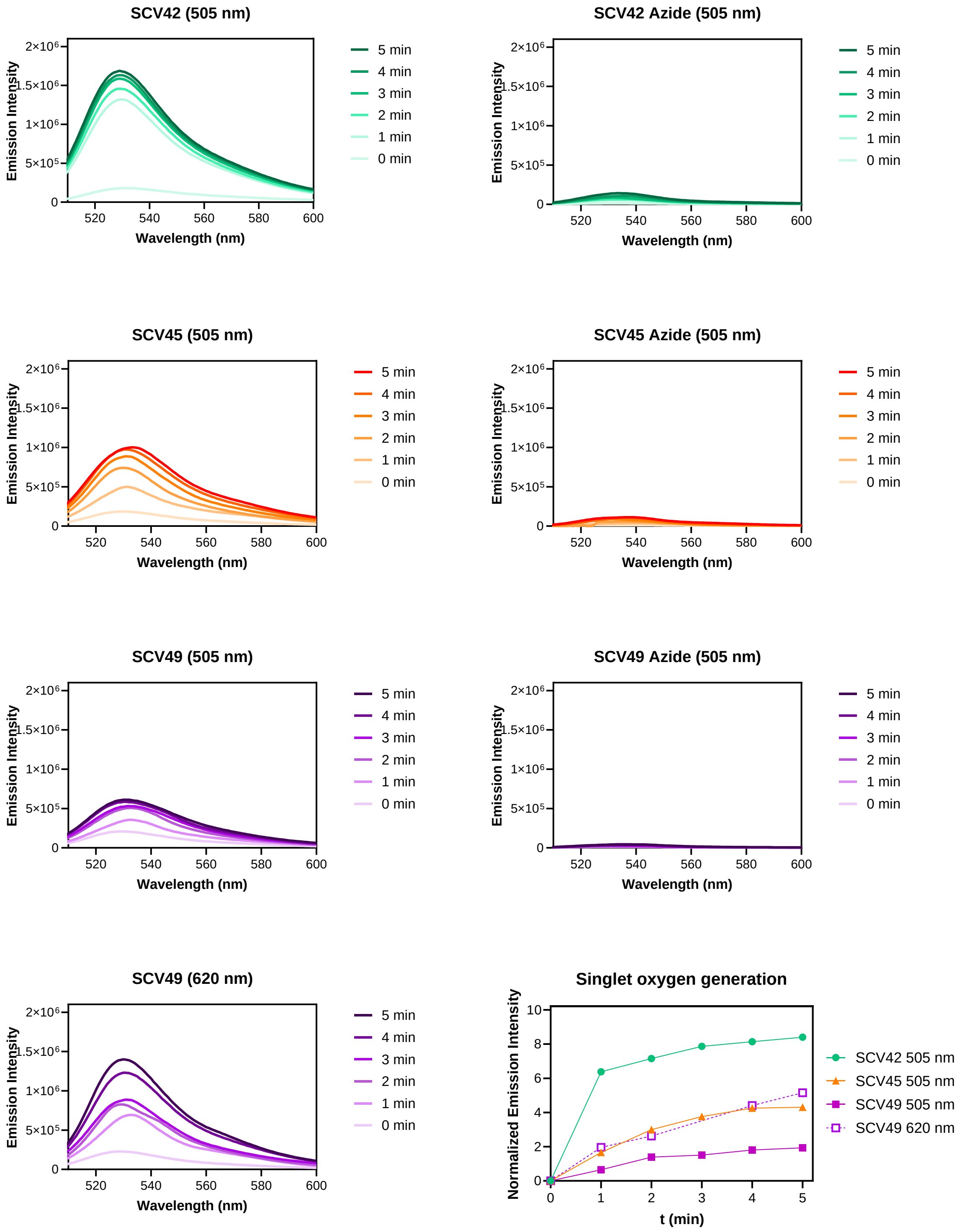

Figure S41. Photogeneration of singlet oxygen by **SCV42**, **SCV45** and **SCV49**. Increase of the fluorescence spectra emission of SOSG upon irradiation of the compounds alone with green or red light in PBS (2 % DMSO) or in the presence of sodium azide-saturated PBS (2 % DMSO).

6.2. Quantification of singlet oxygen generation

6.2.1. Direct method

Singlet oxygen generation was studied by time-resolved near-infrared phosphorescence by means of a customised setup. Briefly, a pulsed Nd:YAG laser (FTSS355-Q, Crystal Laser, Berlin, Germany) working at 1 or 10 kHz repetition rate at 355 nm (0.5 µJ per pulse) or 532 nm (1.2 µJ per pulse) was used to excite the sample. A 1064-nm rugate notch filter (Edmund Optics) and an uncoated SKG-5 filter (CVI Laser Corporation) were placed in the laser path to remove any NIR emission. The light emitted by the sample was filtered with a 1000-nm long-pass filter (Edmund Optics) and later by a narrow bandpass filter at 1275 nm (BK-1270-70-B, bk Interferenzoptik). A thermoelectric-cooled NIR-sensitive photomultiplier tube assembly (H9170-45, Hamamatsu Photonics, Hamamatsu, Japan) was used as detector. Photon counting was achieved with a multichannel scaler (NanoHarp 250, PicoQuant). The time dependence of the $^1O_2$ phosphorescence with the signal intensity $S(t)$ is described by Equation 2, in which τ_T and τ_Δ are the lifetimes of the photosensitizer triplet state and of $^1O_2$ respectively, and S_0 a preexponential parameter proportional to Φ_Δ.

$$S_{1275}(t) = S_{1275}(0) \times \frac{\tau_\Delta}{\tau_\Delta - \tau_T} \times \left(e^{-t/\tau_\Delta} - e^{-t/\tau_T}\right) \qquad (2)$$

The Φ_Δ values of the different samples were obtained by comparing S_0 values of optically matched samples and using an appropriate reference, by means of equation 3.

$$\Phi_{\Delta,\mathrm{sample}} = \Phi_{\Delta,\mathrm{ref}} \times \frac{S_{0\,Sample}}{S_{0\,Ref}} \qquad (3)$$

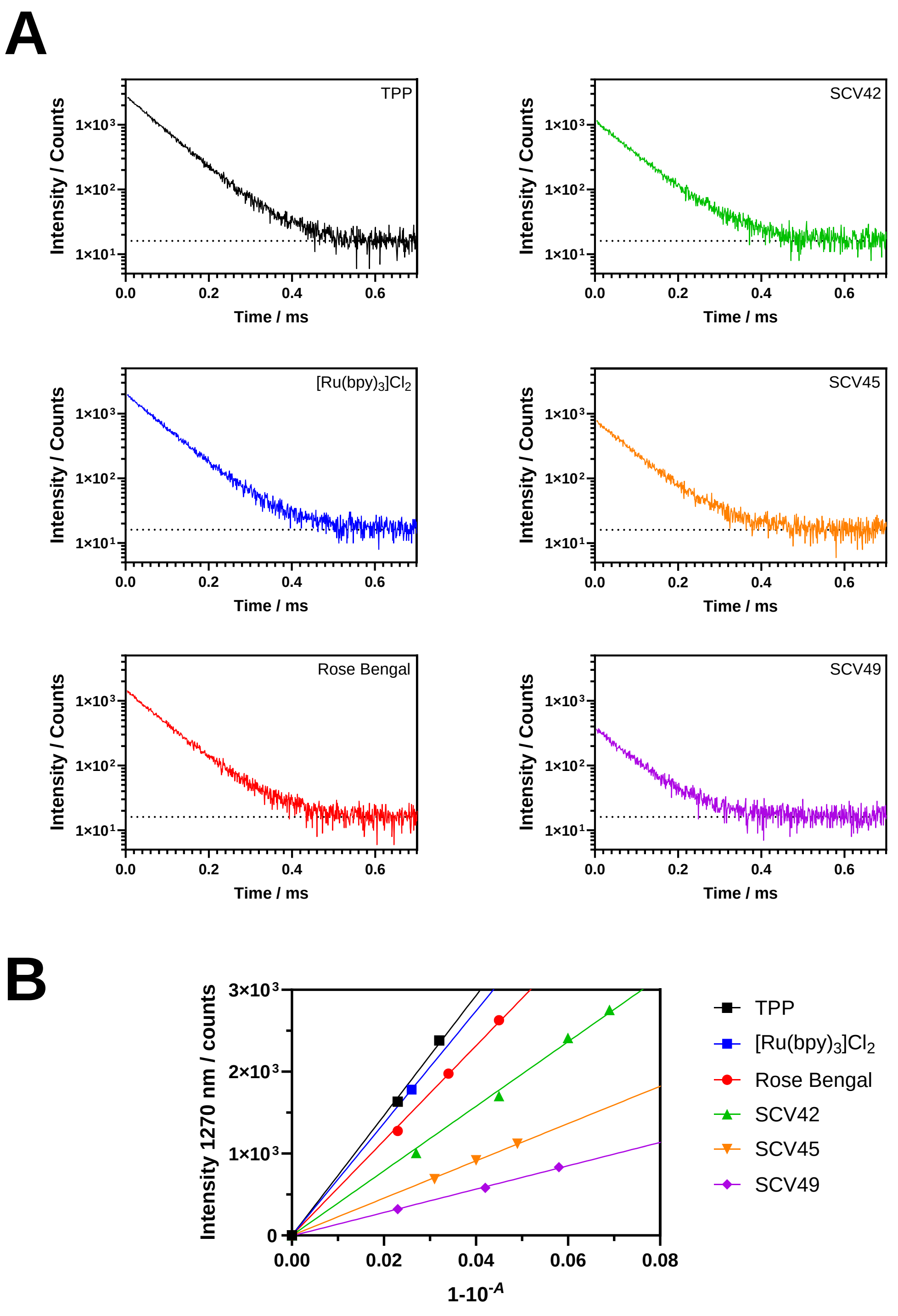

Figure S42. Determination of singlet oxygen quantum yields. **A**: Representative time-resolved singlet oxygen phosphorescence traces at 1270 nm upon pulsed laser excitation (532 nm) of air-saturated solutions of the complexes and reference compounds in acetonitrile. The references were *meso*-tetraphenylporphine (TPP, $F_D = 0.60$)[21], $[Ru(bpy)_3]Cl_2$ ($F_D = 0.55$)[22], and Rose Bengal ($F_D = 0.53$)[22]. **B**: Dependence of the singlet oxygen phosphorescence intensity on the compounds' absorbance.

6.2.2. Indirect method.

Following previously reported procedures,[23] singlet oxygen quantum yields of Ru(II)-COUBPY complexes were determined in an air-saturated DCM or CH_3CN solution (bubbled for 15 min) using 1,3-diphenylisobenzofuran (DPBF) as a chemical trap upon green or red light irradiation using a high-power LED source (505±35 nm, 100 mW cm^{-2}; 620±15 nm; 130 mW cm^{-2}).[24] Upon reaction with singlet oxygen, the fluorescent scavenger DPBF decomposes into a colorless product.[25] The starting absorbance of DPBF in DCM or CH_3CN was adjusted around 1.0 (50 μM), then Ru(II)-CBOUPY complexes were added to the cuvette and their absorbance was adjusted to 0.06 at the light irradiation wavelength (505 or 620 nm). Then, the decrease in the absorbance of DPBF at 411 nm was monitored after irradiation at 505 or 620 nm. The linear relation of the variation in the absorbance (A_0-A_t) of DPBF at 411 nm against irradiation time was plotted as shown in Figures S41. Singlet oxygen quantum yields were calculated by the following equation (4):

$$\Phi_{\Delta,\mathrm{sample}} = \Phi_{\Delta,\mathrm{ref}} \frac{m_s}{m_r} \frac{(1-10^{\mathrm{A\lambda r}})}{(1-10^{\mathrm{A\lambda s}})} \qquad (4)$$

where $\Phi_{\Delta,\mathrm{ref}}$ is the reference singlet oxygen quantum yield of $[Ru(bpy)_3]Cl_2$ ($\Phi_{\Delta,\mathrm{ref}}$ = 0.57 in aerated CH_3CN)[26] or methylene blue (MB) ($\Phi_{\Delta,\mathrm{ref}}$ = 0.57 in aerated DCM),[27] *m* are the slopes and Aλs and Aλr are the absorbance of the compounds and of the reference ($[Ru(bpy)_3]Cl_2$ or MB at the irradiation wavelength, respectively.

Table S4. Singlet oxygen quantum yields (Φ_Δ) in CH_3CN determined either by direct method or by a DPBF-based indirect method using $[Ru(bpy)_3]Cl_2$ and MB as a reference.

	Direct method		Indirect method		
	**Φ_Δ 355 nm**	**Φ_Δ 532 nm**	**Φ_Δ 505 nm**		**Φ_Δ 620 nm**
**cmpd**			**$[Ru(bpy)_3]Cl_2$**	**MB**	**MB**
**SCV42**	0.36	0.33	0.48	0.69	-
**SCV45**	0.16	0.19	0.32	0.33	-
**SCV49**	0.11	0.12	0.21	0.22	0.20

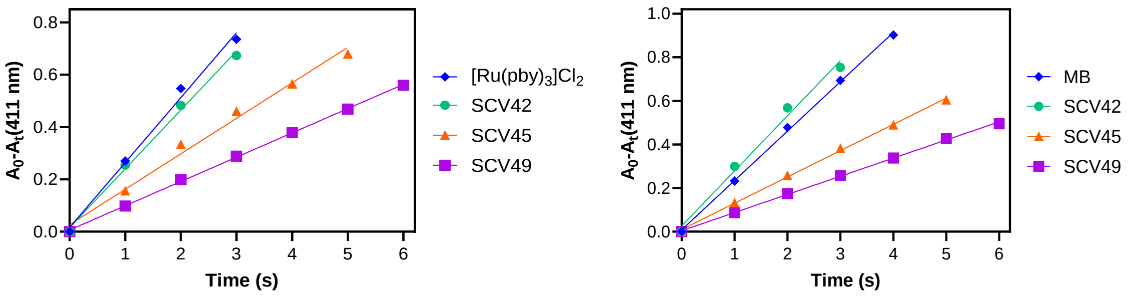

Figure S43. Plot of the changes in the absorbance (A_0-A_t) of DPBF at 411 nm against irradiation time with green light in the presence of the standard sensitizer $[Ru(bpy)_3]Cl_2$ (left) or methylene blue (right) and the Ru(II)-COUBPY complexes in aerated DCM.

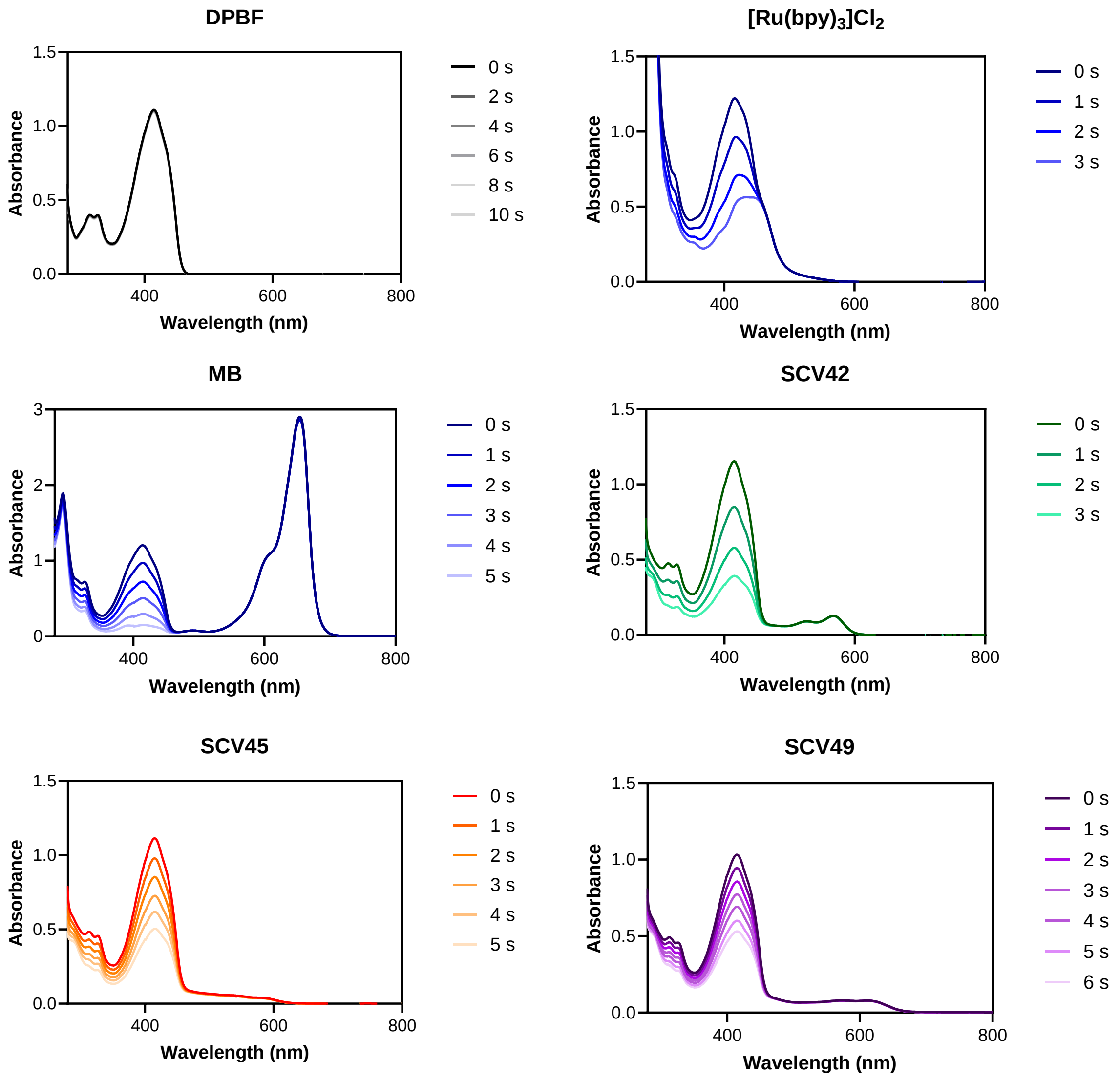

Figure S44. Changes in the absorption spectra of DPBF resulting from the irradiation with green LED light in the absence and in the presence of [Ru(bpy)$_3$]Cl$_2$, methylene blue, **SCV42**, **SCV45** and **SCV49**.

6.3. Evaluation of superoxide anion radical generation using DHR123

All measurements were carried using a Hellma 1.5 mL PTFE-stoppered fluorescence quartz cuvette (4 clear windows) with a 1 cm path length. DHR123 (10 µM) was added to a solution of the corresponding studied compound (10 µM) in PBS containing 2% DMSO. The resulting solutions were irradiated with green (505±35 nm, 100 mW·cm^{-2}) or red (620±15 nm, 130 mW·cm^{-2}) LED light for the indicated time intervals (0, 1, 2, 3, 4 and 5 min). Immediately, the fluorescence spectra of the irradiated samples were collected by using a Photon Technology International (PTI) fluorimeter. The excitation wavelength was set to 500 nm, and emission spectra were recorded from 500-600 nm (DHR123: λ_{Ex}=507 nm, λ_{Em}=529 nm). The entrance and exit slits of the excitation and emission monochromators were set at 0.5 mm, giving a spectral bandwidth of 2 nm. The data interval was 1 nm and the integration time was 0.7 sec. Positive control experiments were carried out using methylene blue (MB) as a reference. Negative control experiments were carried out using sodium 4,5-dihydroxybenzene-1,3-disulfonate (tiron)-saturated PBS as a superoxide anion radical scavenger.

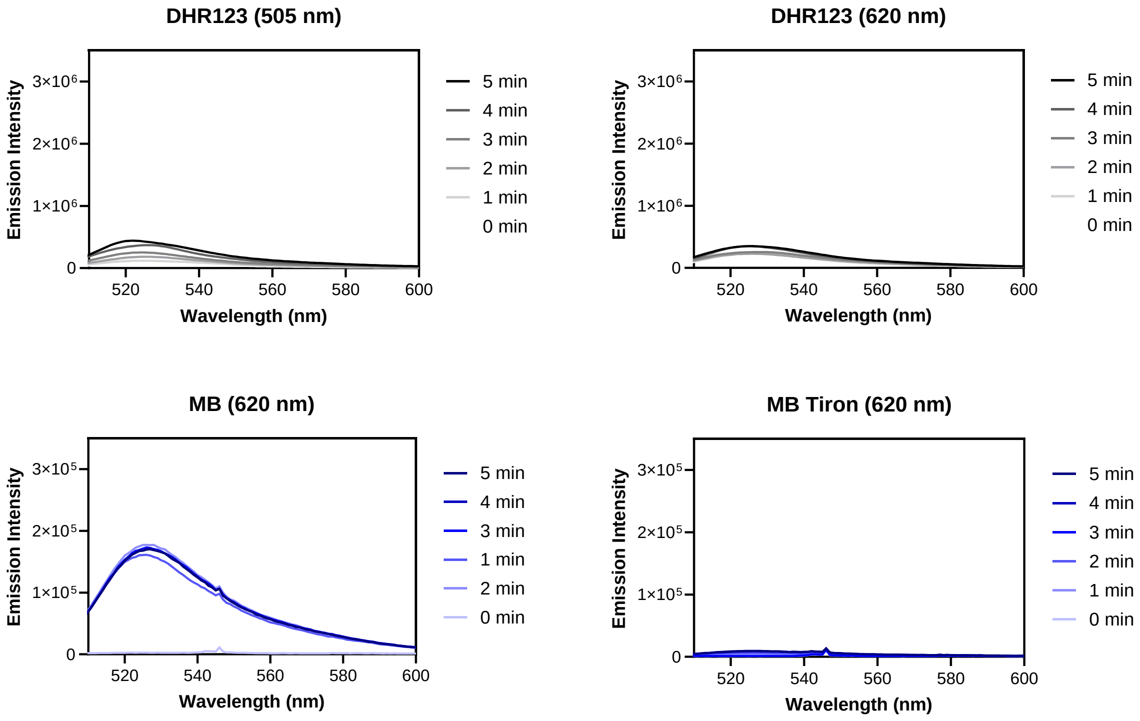

Figure S45. Top: Emission spectra of DHR123 upon irradiation with green or red light in PBS (2 % DMSO). Bottom: Photogeneration of superoxide anion by **MB**. Increase of the fluorescence spectra emission of DHR123 upon irradiation of the compound alone with red light in PBS (2 % DMSO) or in the presence of tiron-saturated PBS (2 % DMSO).

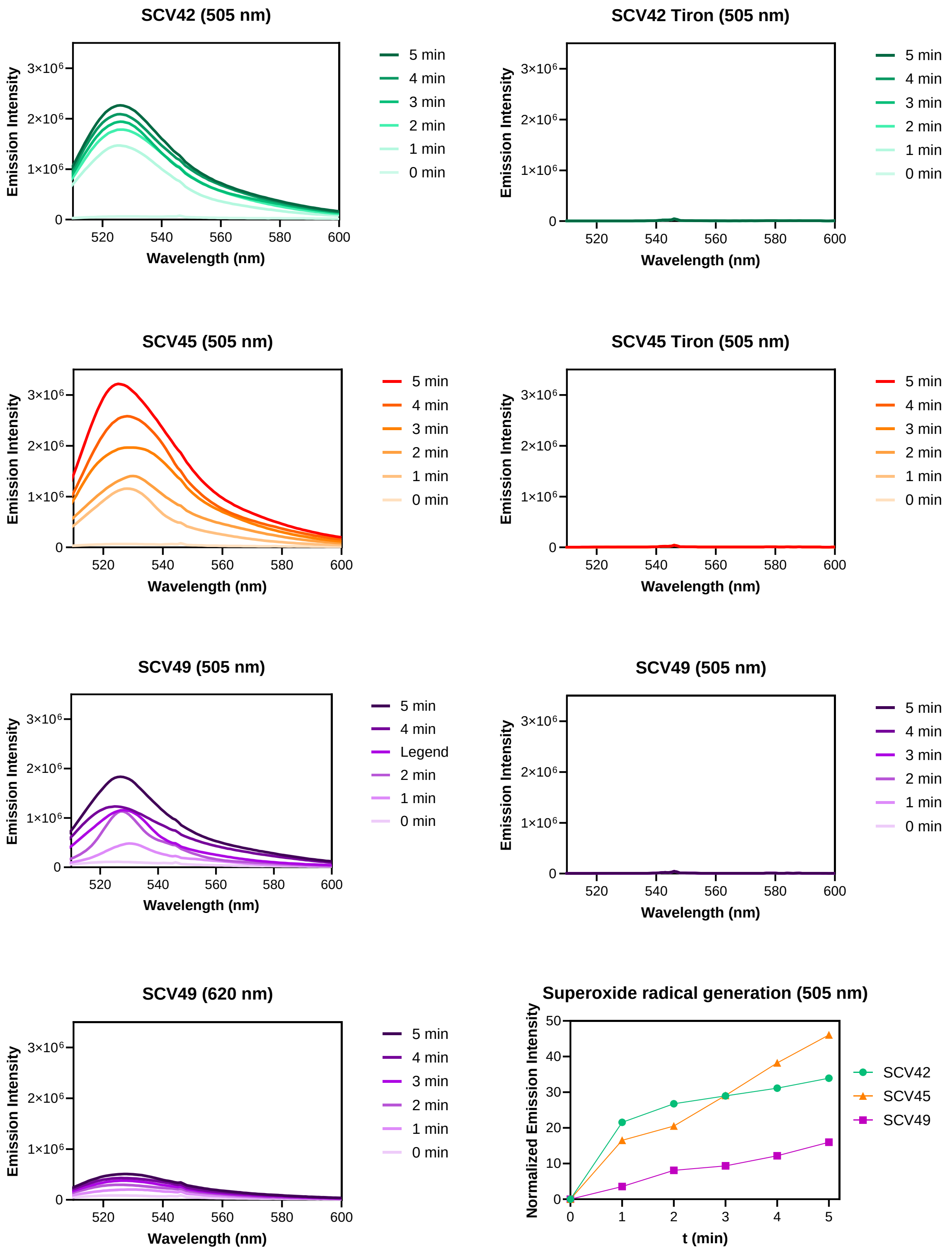

Figure S46. Photogeneration of superoxide anion by **SCV42**, **SCV45** and **SCV49**. Increase of the fluorescence spectra emission of DHR123 upon irradiation of the compounds alone with green or red light in PBS (2 % DMSO) or in the presence of tiron-saturated PBS (2 % DMSO).

6.4. Evaluation of hydroxyl radical generation using HPF

All measurements were carried using a Hellma 1.5 mL PTFE-stoppered fluorescence quartz cuvette (4 clear windows) with a 1 cm path length. HPF (5 µM) was added to a solution of the corresponding studied compound (10 µM) in PBS containing 2% DMSO. The resulting solutions were irradiated with green (505±35 nm, 100 mW·cm^{-2}) or red (620±15 nm, 130 mW·cm^{-2}) LED light for the indicated time intervals (0, 1, 2, 3, 4 and 5 min). Immediately, the fluorescence spectra of the irradiated samples were collected by using a Photon Technology International (PTI) fluorimeter. The excitation wavelength was set to 490 nm, and emission spectra were recorded from 500-600 nm (HPF: λ_{Ex}=490 nm, λ_{Em}=515 nm). The entrance and exit slits of the excitation and emission monochromators were set at 0.5 mm, giving a spectral bandwidth of 2 nm. The data interval was 1 nm and the integration time was 0.7 sec. Positive control experiments were carried out using MB as a reference. Negative control experiments were carried out using terephthalic acid (TA)-saturated PBS as a hydroxyl radical scavenger.

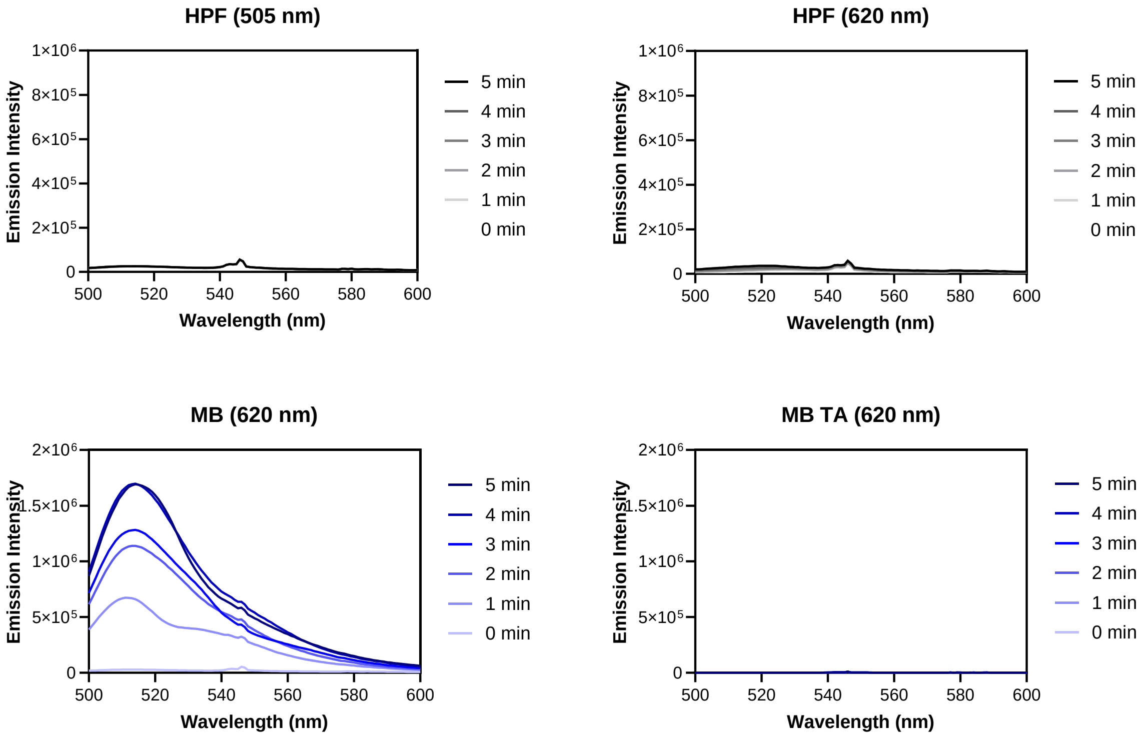

Figure S47. Top: Emission spectra of HPF alone upon irradiation with green or red light in PBS (2 % DMSO). Bottom: Photogeneration of hydroxyl radical by **MB**. Increase of the fluorescence spectra emission of HPF upon irradiation of the compound and HPF with red light in PBS (2 % DMSO) or in the presence of TA-saturated PBS (2 % DMSO).

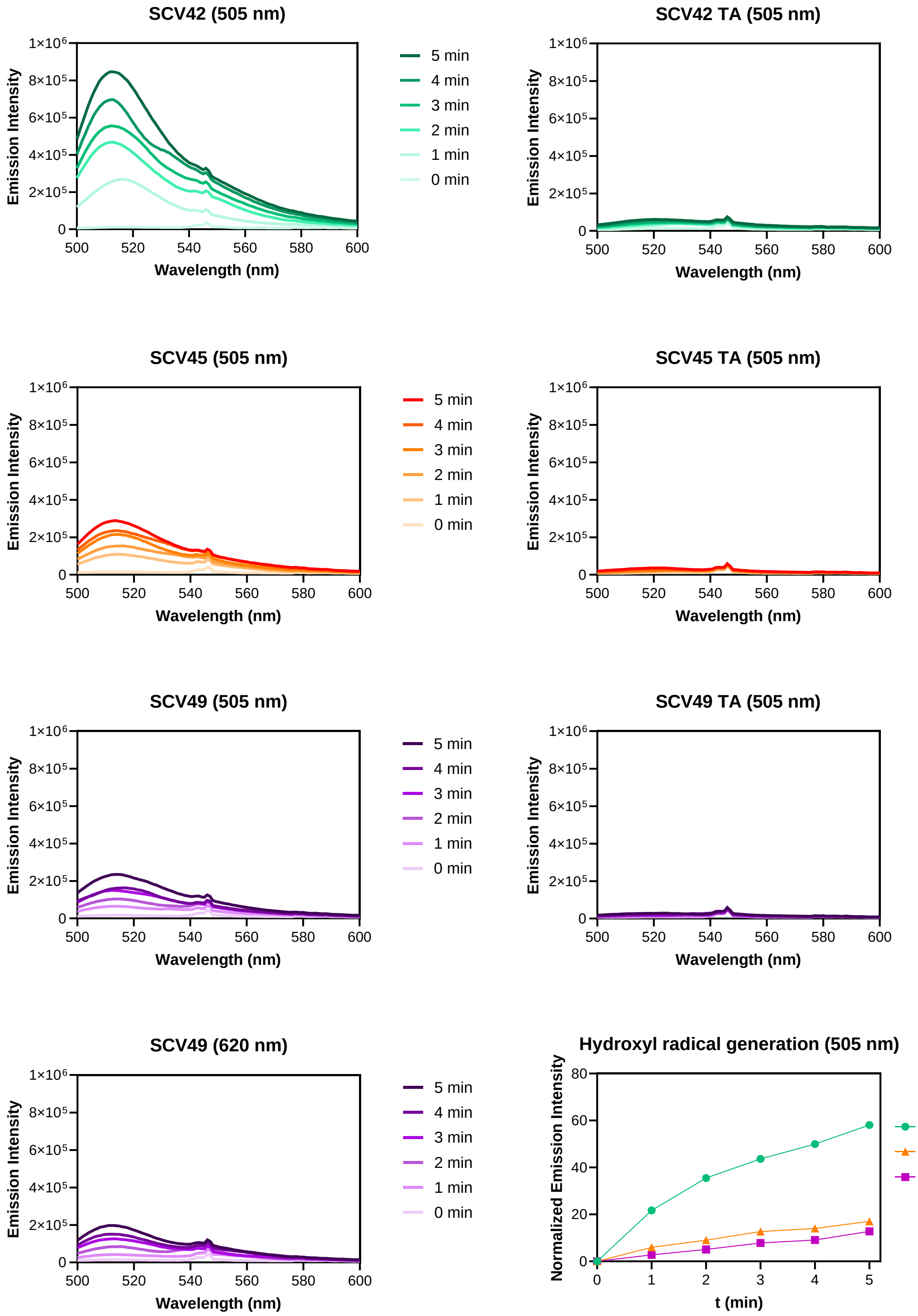

Figure S48. Photogeneration of hydroxyl radical by **SCV42**, **SCV45** and **SCV49**. Increase of the fluorescence spectra emission of HPF upon photoirradiation of the compounds alone with green or red light in PBS (2 % DMSO) or in the presence of TA-saturated PBS (2 % DMSO).

6.5. Electron paramagnetic resonance (EPR) studies

Ru-COUBPY complexes (500 µM) were dissolved in MeOH containing either 60 mM 4-amino-TEMP (4-amino-2,2,6,6-tetramethylpiperidine) as a spin trap for $^1O_2$, or 720 mM DMPO (5,5-dimethyl-1-pyrroline-*N*-oxide) as a spin trap for $O_2^{\bullet -}$, and the resulting samples were loaded into Hirschmann 50 µL capillary tubes, with both ends open, and sealed with Critoseal®. EPR spectra were recorded in the dark and after 2 min for $^1O_2$ or 1 min for $O_2^{\bullet -}$ of irradiation with a high-power green (505 nm, 100 mW cm^{-2}) or red (620 ± 15 nm, 130 mW cm^{-2}) light LED. EPR spectra were recorded on a Bruker Elexys 580 Spectrometer working in X-band at room temperature. All spectra were collected in continuous-wave (CW) regime using a Bruker ER4122 SHQE superhigh-Q cylindrical resonator. The microwave frequency was 9.858 GHz, and both the modulation amplitude (0.05 mT) and the microwave power (4.7 mW) were selected in such a way that no distortion or saturation of the signal was produced.

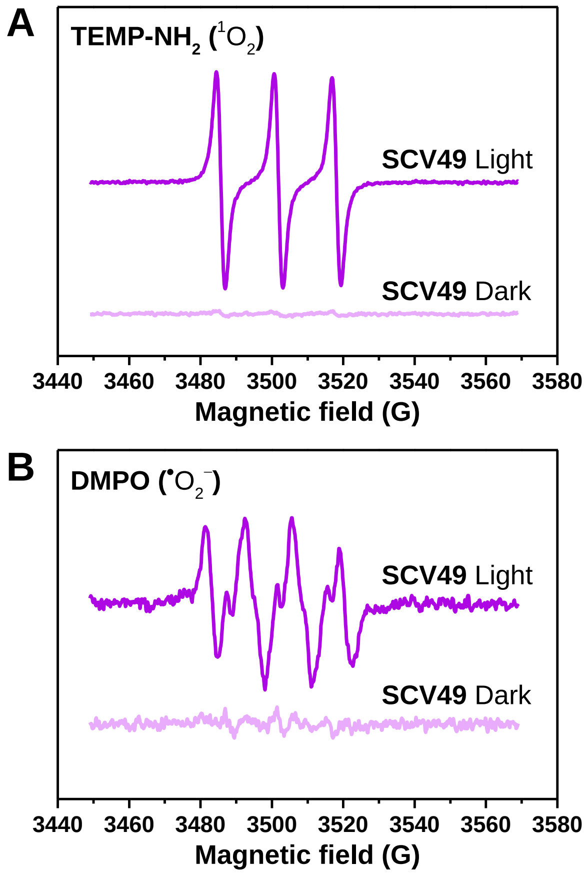

Figure 49 EPR spectra of **SCV49** trapped by 4-amino-TEMP (A) or DMPO (B) in MeOH, measured in the dark and after red light irradiation (620 ± 15 nm, 130 mW/cm^2).

6.6. Computational studies

All thermodynamic values have been computed with the PBE0/6-31+G(d,p)/SDD method in water (CPCM) as implemented in the Gaussian 16 quantum chemistry software. Vertical electron affinities (VEAs) and ionization potentials (VIPs) are computed as follows:

VEA = $E(A_{n+1}) - E(A_n)$

VIP = $E(A_{n-1}) - E(A_n)$

Where E stands for absolute energy, A is the molecule under study, and n refers to the total number of electrons. VEAs and VIPs have been computed by means of restricted and unrestricted DFT determinations for closed and open shell systems, respectively, as summarized in Table S5 and S6. VEAs and VIPs involving excited triplet states (see Table 2 of the main text) were computed by adding the vertical T_1-S_0 energy gap at the TD-M06 level of theory (Table S7), for consistency with the photophysical description provided in section 4.2 above. Thermodynamic properties of the PDT type I electron transfer reactions shown in Table 2 were computed using the VIPs, VEAs, and vertical T_1-S_0 energy gaps of the involved species as summarized in Tables S6 and S7. The use of VEAs and VIPs to evaluate the feasibility of Type I mechanisms by metal- or metal-free photosensitizers for PDT is widely used in literature.[28,29,30,31]

Table S5. Energies and system details used to compute VIPs and VEAs shown below and the thermodynamic values reported in Table 2.

Geometry	Electronic state	Molecular Charge	Multiplicity (2S+1)	Energy (a.u.)
^{1}SCV42^{2+}	^{1}SCV42^{2+}	2	1	-2421.80615521
^{1}SCV42^{2+}	^{2}SCV42^{3+}	3	2	-2421.60685714
^{1}SCV42^{2+}	^{2}SCV42^{+}	1	2	-2421.91236677
^{1}SCV45^{2+}	^{1}SCV45^{2+}	2	1	-2497.96844408
^{1}SCV45^{2+}	^{2}SCV45^{3+}	3	2	-2497.77565973
^{1}SCV45^{2+}	^{2}SCV45^{+}	1	2	-2498.07248439
^{1}SCV49^{2+}	^{1}SCV49^{2+}	2	1	-2795.43259020
^{1}SCV49^{2+}	^{2}SCV49^{3+}	3	2	-2795.23454879
^{1}SCV49^{2+}	^{2}SCV49^{+}	1	2	-2795.54689491
^{1}COU42	^{1}COU42	0	1	-843.657725307
^{1}COU42	^{2}COU42^{+}	1	2	-843.654132096
^{2}COU42^{+}	^{2}COU42^{+}	1	2	-843.47188440
^{2}COU42^{+}	^{1}COU42	0	1	-843.65413210
^{1}COU45	^{1}COU45	0	1	-919.819645664
^{1}COU45	^{2}COU45^{+}	1	2	-919.635881534
^{2}COU45^{+}	^{2}COU45^{+}	1	2	-919.64024926
^{2}COU45^{+}	^{1}COU45	0	1	-919.81631653
^{1}COU49	^{1}COU49	0	1	-1217.28860634
^{1}COU49	^{2}COU49^{+}	1	2	-1217.09849204
^{2}COU49^{+}	^{2}COU49^{+}	1	2	-1217.10214461
^{2}COU49^{+}	^{1}COU49	0	1	-1217.28580978
^{1}[Ru(bpy)$_2$(dmbpy)]$^{2+}$	^{1}[Ru(bpy)$_2$(dmbpy)]$^{2+}$	2	1	-1657.88248662
^{1}[Ru(bpy)$_2$(dmbpy)]$^{2+}$	^{2}[Ru(bpy)$_2$(dmbpy)]$^{+}$	1	2	-1657.98706028
^{2}[Ru(bpy)$_2$(dmbpy)]$^{+}$	^{2}[Ru(bpy)$_2$(dmbpy)]$^{+}$	1	2	-1657.99231815
^{2}[Ru(bpy)$_2$(dmbpy)]$^{+}$	^{1}[Ru(bpy)$_2$(dmbpy)]$^{2+}$	2	1	-1657.87661714

Table S6. VIPs, VEAs, and vertical ΔE(T$_1$-S$_0$) energy gaps.

Molecule	VEA	VIP	ΔE(T$_1$-S$_0$)^a
^{1}SCV42	-2.89	5.42	2.0328
^{1}SCV45	-2.83	5.25	1.9965
^{1}SCV49	-3.11	5.39	1.7275
^{1}COU42		5.15	2.2417
^{1}COU45		5.00	2.1924
^{1}COU49		5.17	1.8903
^{2}COU42^{+}	-4.96		
^{2}COU45^{+}	-4.79		
^{2}COU49^{+}	-5.00		
^{1}[Ru(bpy)$_2$(dmbpy)]$^{2+}$	-2.85		
^{2}[Ru(bpy)$_2$(dmbpy)]$^{+}$		3.15	
^{3}O$_2$	-3.42^b		

^{a} Computed with the TD-M06/6-31+G(d,p)/SDD method in ACN at the ground-state geometry.

^{b} Adiabatic value taken from ref. 29.

Table S7. Determination of the PDT type I thermodynamics based on VIPs, VEAs, and vertical ΔE(T_1-S_0) energy gaps.

#	Reaction	**ΔE = *E*(products) – *E*(reactants)**
(1)	$^1SCV^{2+} + {}^3O_2 \rightarrow {}^2SCV^{3+} + {}^2(^\bullet O_2^-)$	VIP($^1SCV^{2+}$) + VEA($^3O_2$)
(2)	$^3SCV^{2+} + {}^3O_2 \rightarrow {}^2SCV^{3+} + {}^2(^\bullet O_2^-)$	VIP($^1SCV^{2+}$) + VEA($^3O_2$) □ $\Delta E(T_1\text{-}S_0)_{SCV}$
(3)	$^1SCV^{2+} + {}^1SCV^{2+} \rightarrow {}^2SCV^{3+} + {}^2SCV^{+}$	VIP($^1SCV^{2+}$) + VEA($^1SCV^{2+}$)
(4)	$^3SCV^{2+} + {}^3SCV^{2+} \rightarrow {}^2SCV^{3+} + {}^2SCV^{+}$	VIP($^1SCV^{2+}$) + VEA($^1SCV^{2+}$) □ $2\cdot\Delta E(T_1\text{-}S_0)_{SCV}$
(5)	$^2SCV^{+} + {}^3O_2 \rightarrow {}^1SCV^{2+} + {}^2(^\bullet O_2^-)$	-VEA($^1SCV^{2+}$) + VEA($^3O_2$)
(6)	$^3COU + {}^1[Ru(bpy)_2(dmbpy)]^{2+} \rightarrow {}^2COU^{+} + {}^2[Ru(bpy)_2(dmbpy)]^{+}$	VIP(^1COU) + VEA($^1[Ru(bpy)_2(dmbpy)]^{2+}$) □ $\Delta E(T_1\text{-}S_0)_{COU}$
(7)	$^2[Ru(bpy)_2(dmbpy)]^{+} + {}^3O_2 \rightarrow {}^1[Ru(bpy)_2(dmbpy)]^{2+} + {}^2(^\bullet O_2^-)$	VIP($^2[Ru(bpy)_2(dmbpy)]^{+}$) + VEA($^3O_2$)
(8)	$^2[Ru(bpy)_2(dmbpy)]^{+} + {}^2COU^{+} \rightarrow {}^1[Ru(bpy)_2(dmbpy)]^{2+} + {}^1COU$	VIP($^2[Ru(bpy)_2(dmbpy)]^{+}$) + VEA($^2COU^{+}$)
(9)	$^3SCV^{2+} + {}^2(^\bullet O_2^-) \rightarrow {}^2SCV^{+} + {}^3O_2$	VEA($^2SCV^{+}$) - VEA($^3O_2$)

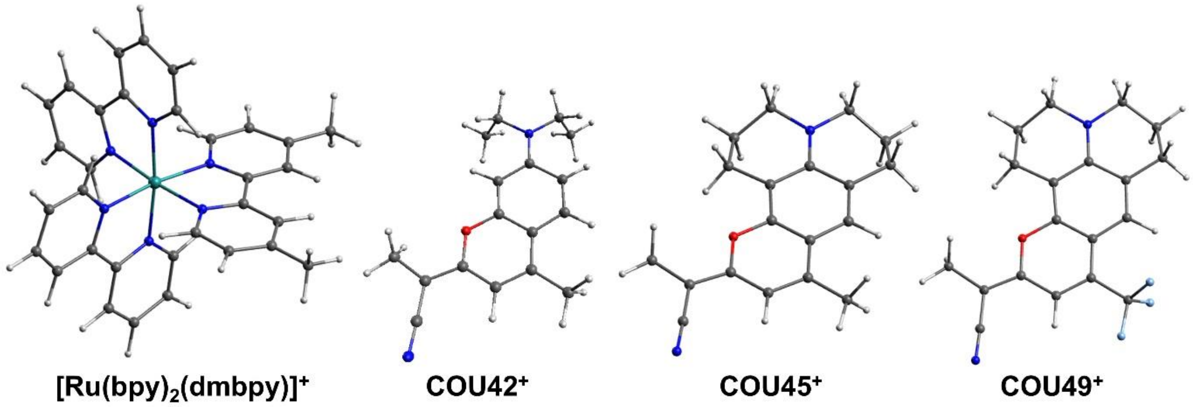

Figure S50. 3D structure of the separated ionic molecular fragments studied in this work.

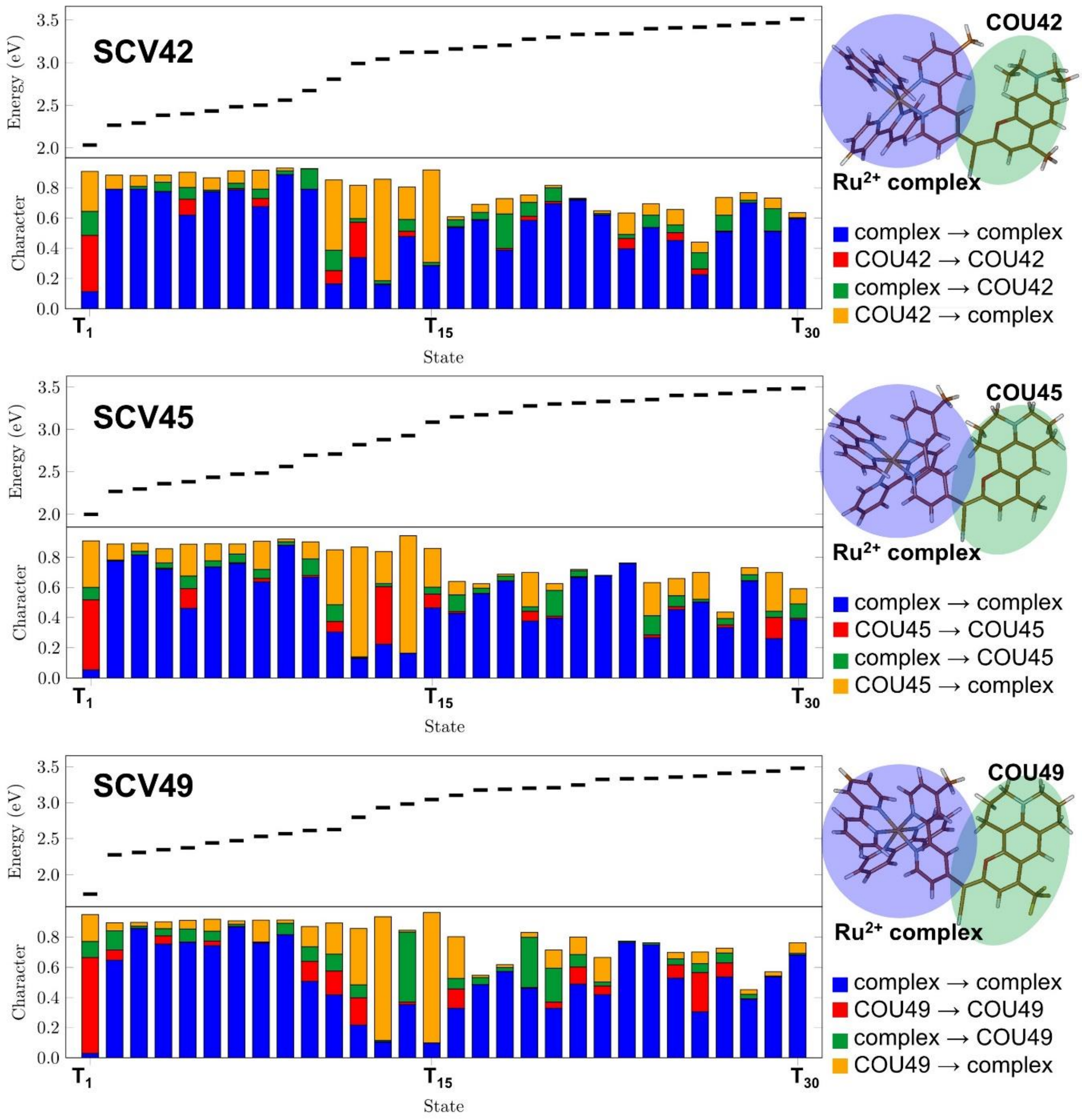

Figure S51. Molecular topology of the triplet excited states at the ground-state geometry of each Ru-COUBPY complex. TD-M06/6-31+G(d,p)/SDD spectrum in ACN, Gaussian output post-processing conducted with the TheoDORE 3.1.1 program.[20]

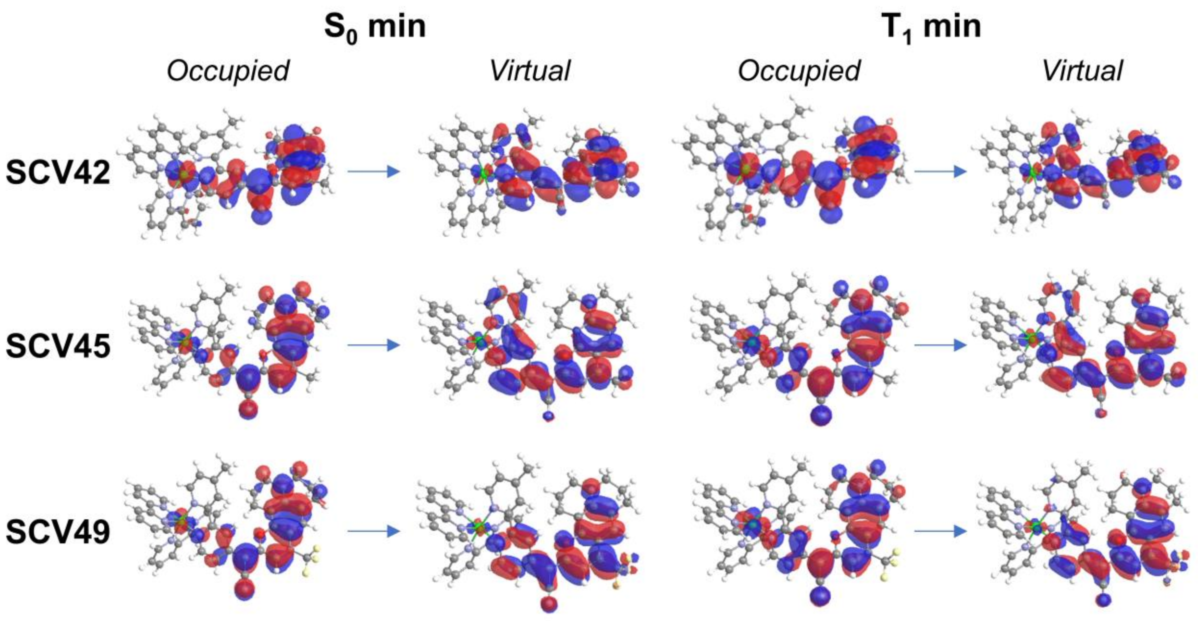

Figure S52. Transition natural orbitals (NTOs) for the T_1 state of the Ru-COUBPY complexes.

7. Cellular uptake studies

7.1. Cellular uptake and confocal microscopy

Cell Culture and Treatments. HeLa cells were maintained in Dulbecco's Modified Eagle Medium (DMEM, 31966021 Gibco) containing GlutaMAX and high glucose (4.5 g/L) supplemented with 10% fetal bovine serum (FBS) and 50 U/mL penicillin-streptomycin. For cellular uptake experiments and posterior observation under the microscope, cells were seeded on glass-bottom dishes (P35G-1.5-14-C, Mattek). Twenty-four hours after cell seeding, cells were incubated for 30 min at 37 °C with the compounds (10 μM) in supplemented DMEM. Then cells were washed two times with Dulbecco's Phosphate-Buffered Saline (DPBS, pH 7.0-7.3) to remove the excess of the compounds and kept in low glucose DMEM with HEPES (10mM, Corning) and without phenol red for fluorescence imaging.

For colocalization experiments with Mitoview 650 or Lysoview 633, HeLa cells were treated with the compounds (10 μM) for 30 min at 37 °C. Then cells were washed with DPBS and incubated with Mitoview 650 (0.1 μM) or Lysoview 633 (1X) for 30 min at 37 °C in non-supplemented DMEM. After removal of the medium and washing two times with DPBS, cells were kept in low glucose DMEM with HEPES (10mM, Corning) and without phenol red for fluorescence imaging. For colocalization experiments with LipidSpot 610, HeLa cells were treated with **SCV49** (10 μM) and LipidSpot (1X) in DMEM complete medium for 30 min at 37ºC. Then cells were washed with DPBS and cells were kept in low glucose DMEM with HEPES (10mM, Corning) and without phenol red for fluorescence imaging.

Fluorescence Imaging. All microscopy observations were performed using a Zeiss LSM 880 confocal microscope equipped with 405 nm, argon-ion, 561 nm and 633 nm lasers. The microscope was also equipped with a Heating Insert P S (Pecon). Cells were observed at 37ºC using a 63× 1.4 oil immersion objective. Compounds **SCV42**, **SCV45** and **SCV49** were excited using the 405 nm, 458 nm and 514 nm laser lines and detected from 460 to 560 nm (when exciting at 405 nm or 458 nm) and from 520 to 620 nm (when exciting at 514 nm). Mitoview 650, Lysoview 633 and LipidSpot 610 were excited using the 633 nm laser line and detected from 640 to 750 nm. In all observations a stack of images was acquired with a step size of 0.36 mm. Image processing and analysis were performed using Fiji.[32]

Image analysis. The *M*itoview or *L*ysoview and the compound channels were processed by median filtering (radius = 1), Gaussian filtering (sigma = 1), and background subtraction (rolling ball radius = 30 for the *M*itoview and radius = 10 for the *L*ysoview staining). In all

experiments colocalization coefficients were measured in 3D using the JaCoP plugin on the stacks of images acquired. For each compound more than 25 cells on average were analysed.

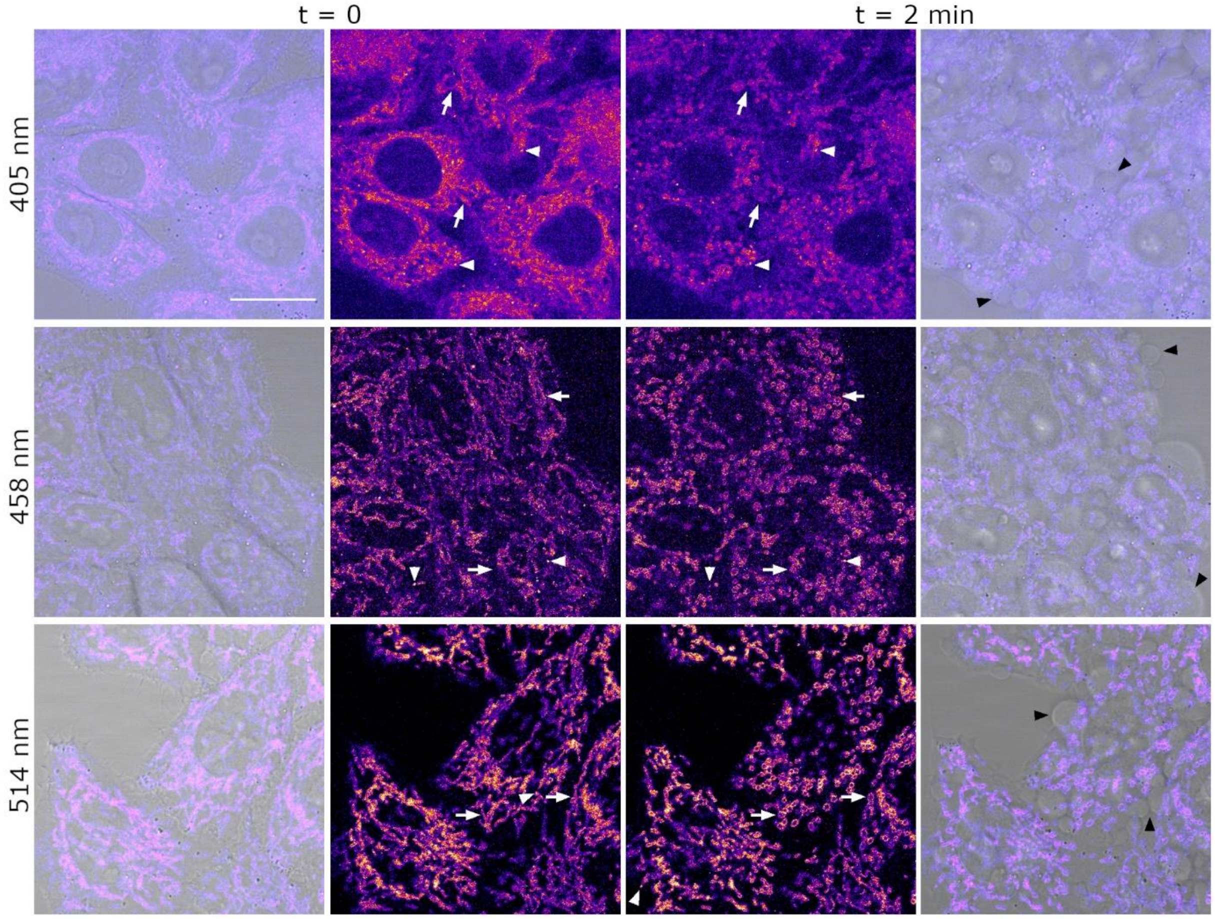

Figure S53. Cellular uptake of compound **SCV42**. Single confocal planes of HeLa cells incubated with the compound (10 μM) for 30 min at 37 °C, imaged at t = 0 and after 2 min of first observation. Excitation was performed with three different laser lines (405, 458 and 514 nm). White arrows point out mitochondria and white arrowheads vesicle staining. Black arrowheads on the right column point out cell blebbings. Scale bar: 20 μm. LUT for compound images: Fire. Left and right columns: merge of compound and brightfield images.

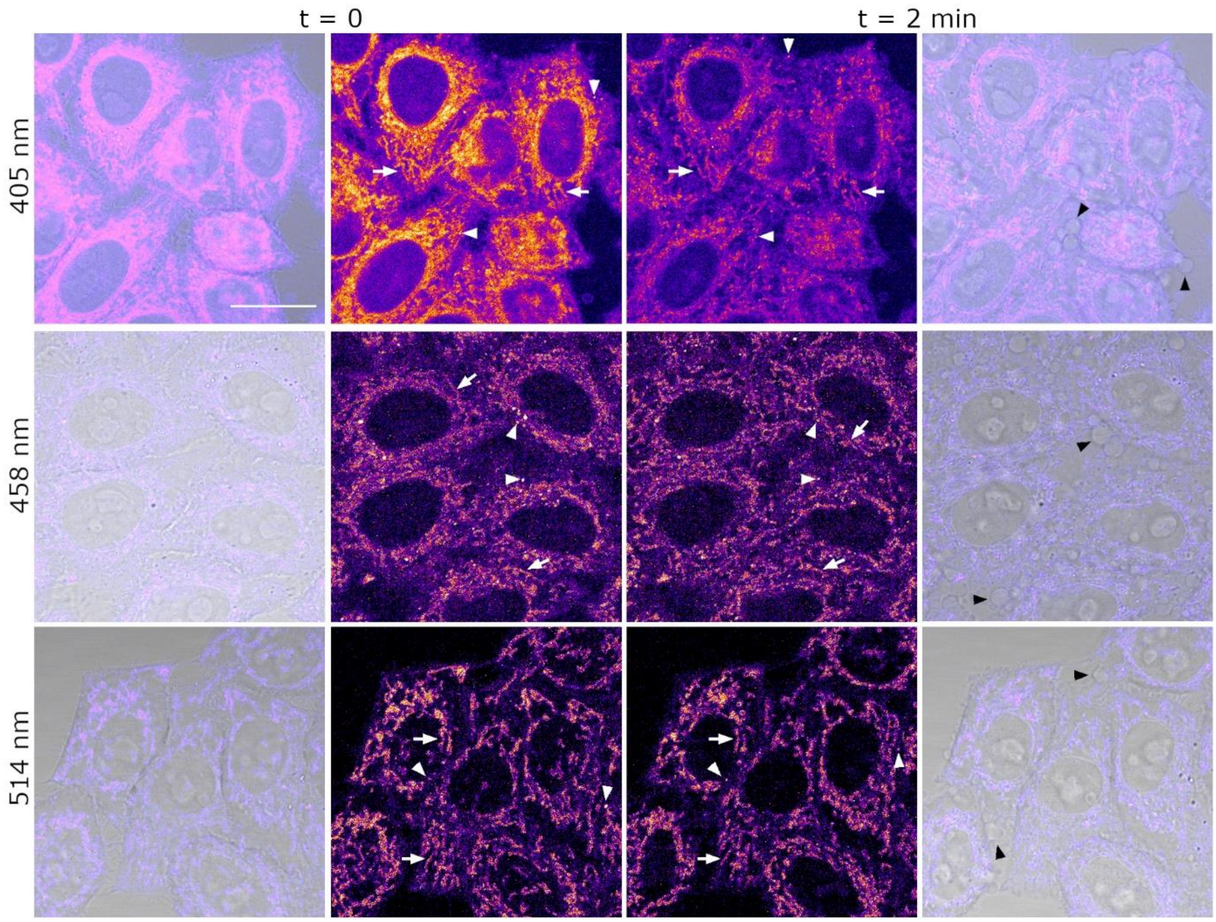

Figure S54. Cellular uptake of compound **SCV45**. Single confocal planes of HeLa cells incubated with the compound (10 μM) for 30 min at 37 °C, imaged at t = 0 and after 2 min of first observation. Excitation was performed with three different laser lines (405, 458 and 514 nm). White arrows point out mitochondria and white arrowheads vesicle staining. Black arrowheads on the right column point out cell blebbings. Scale bar: 20 μm. LUT for compound images: Fire. Left and right columns: merge of compound and brightfield images.

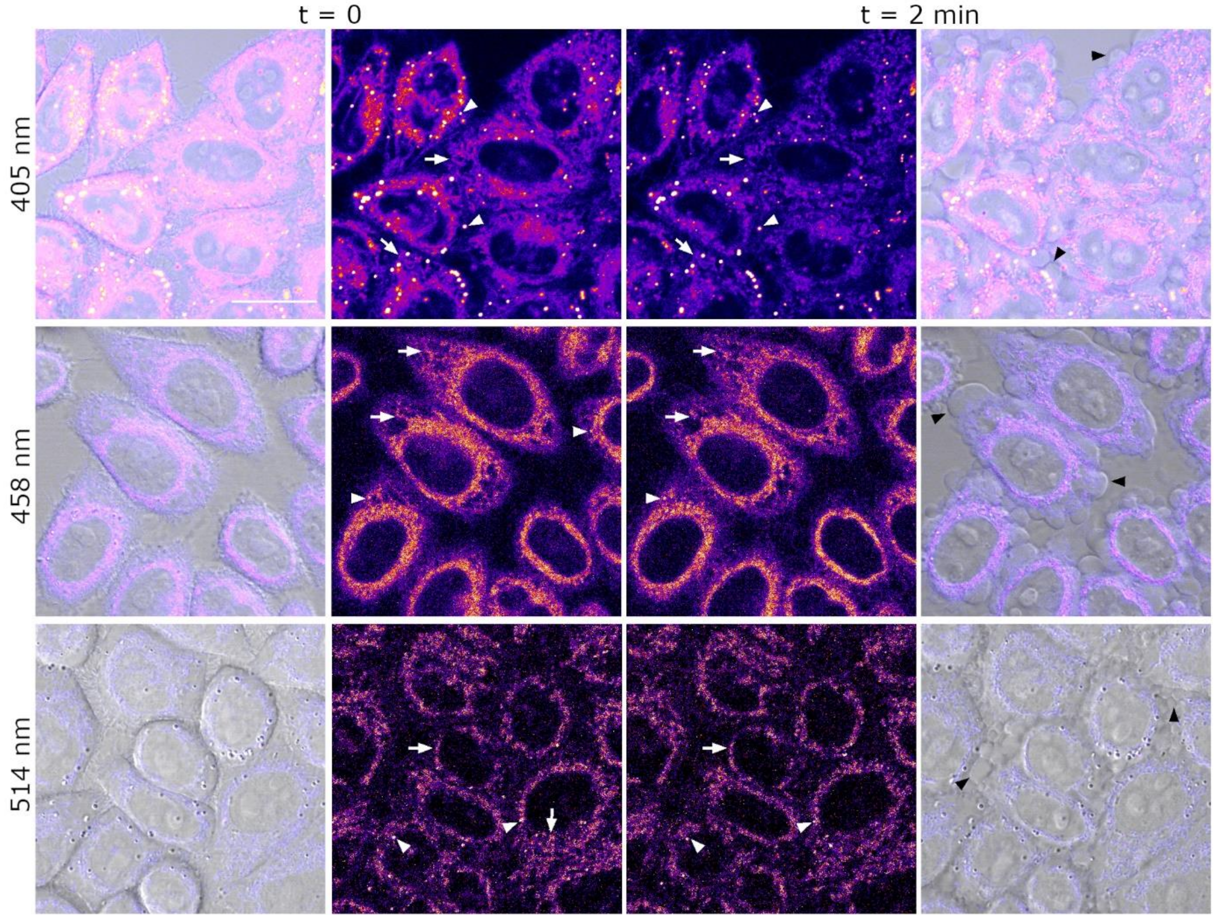

Figure S55. Cellular uptake of compound **SCV49**. Single confocal planes of HeLa cells incubated with the compound (10 μM) for 30 min at 37 °C, imaged at t = 0 and after 2 min of first observation. Excitation was performed with three different laser lines (405, 458 and 514 nm). White arrows point out mitochondria and white arrowheads vesicle staining. Black arrowheads on the right column point out cell blebbings. Scale bar: 20 μm. LUT for compound images: Fire. Left and right columns: merge of compound and brightfield images.

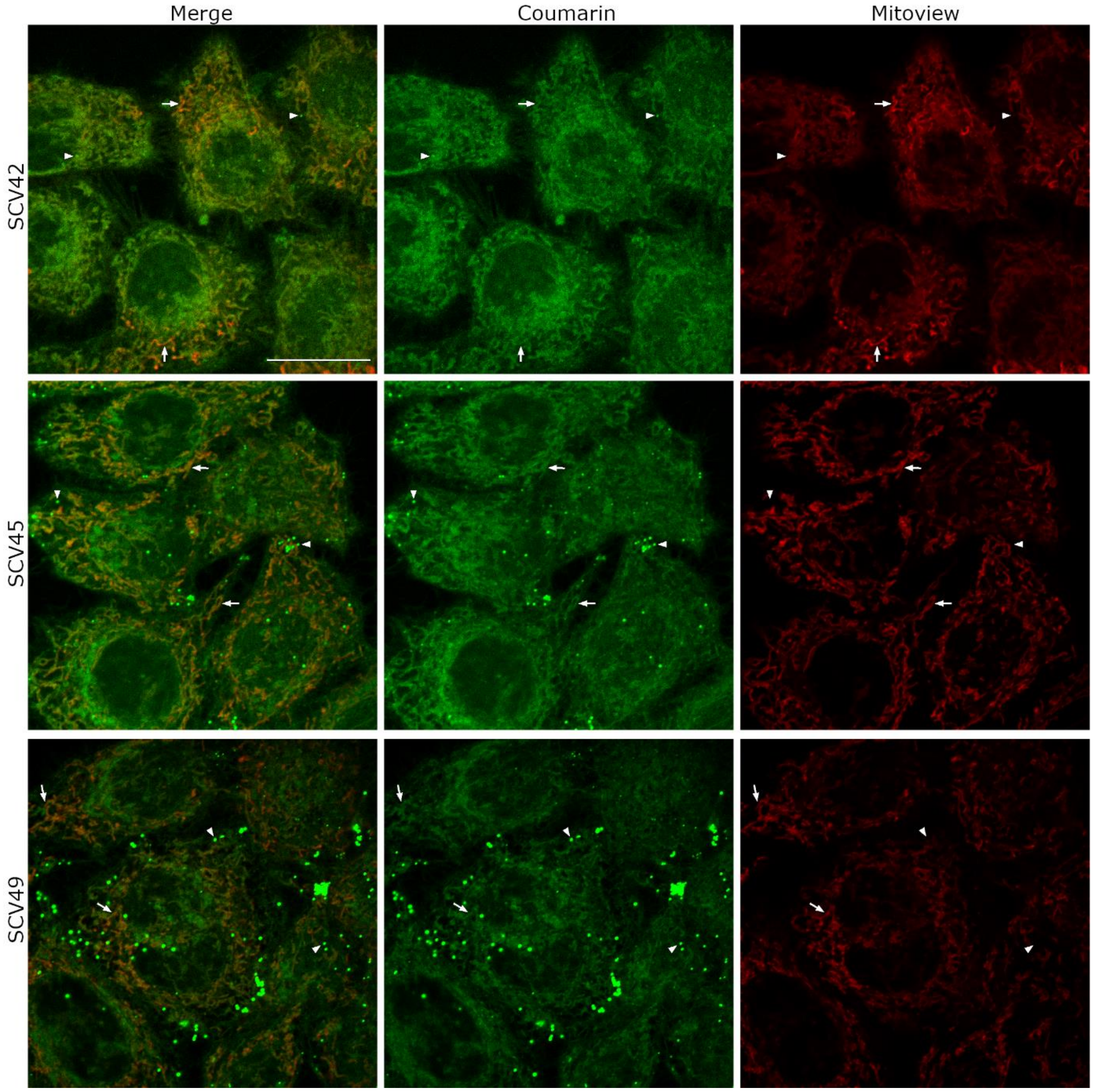

Figure S56. Co-localization studies of Ru(II) complexes (**SCV42**, **SCV45** and **SCV49**) with Mitoview 650. Single confocal planes of HeLa cells incubated with the compounds (10 μM, green) and Mitoview (0.1 μM, red). Left: Overlay of the two staining. Center: Ru(II) complexes' signal. Right: Mitoview signal. White arrows and arrowheads point out positive and negative (vesicles staining in compound images) colocalization, respectively. Scale bar: 20 μm.

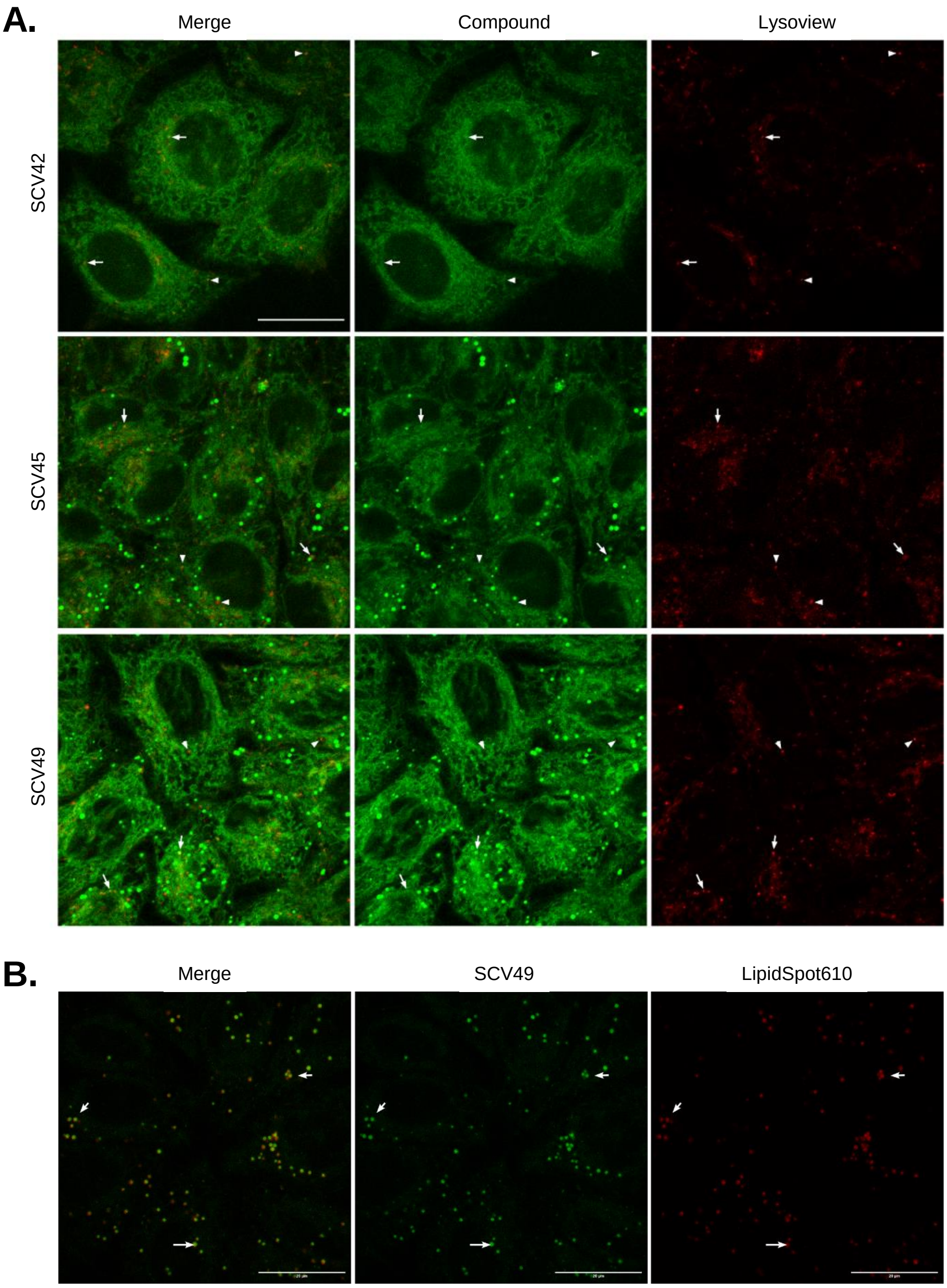

Figure S57. **A.** Co-localization studies of Ru(II) complexes (**SCV42**, **SCV45** and **SCV49**) with Lysoview 633. Single confocal planes of HeLa cells incubated with the compounds (10 µM, green) and Lysoview (1X, red). Left: Overlay of the two staining. Center: Ru(II) complexes' signal (Excitation at 514 nm). Right: Lysoview signal (Excitation at 633 nm). White arrows and arrowheads point out positive and negative colocalization, respectively. Scale bar: 20 µm. **B.** Co-localization studies of **SCV49** with LipidSpot 610. Single confocal planes of HeLa cells incubated with **SCV49** (10 µM, green) and LipidSpot 610 (1X, red). Left: Overlay of the two staining. Center: **SCV49** signal (Excitation at 405 nm). Right: LipidSpot 610 signal (Excitation at 633 nm). White arrows point out positive colocalizations. Scale bar: 20 µm.

Table S8. Pearson's correlation coefficient and Mander's overlap colocalization coefficients of the compounds with Mitoview 650, Lysoview 633 and Lipid Spot 610. M1 corresponds to the percentage of colocalization of the compound channel towards the marker channel and M2 the percentage of colocalization of the marker channel towards the compound channel.

	**Mitoview 650**			**Lysoview 633**			**LipidSpot 610**		
**Comp.**	**Pearson**	**M1**	**M2**	**Pearson**	**M1**	**M2**	**Pearson**	**M1**	**M2**
**SCV42**	0.80±0.04	0.59±0.13	0.78±0.04	0.25±0.02	0.08±0.02	0.43±0.04	-	-	-
**SCV45**	0.67±0.01	0.56±0.11	0.74±0.08	0.18±0.03	0.02±0.01	0.56±0.04	-	-	-
**SCV49**	0.55±0.03	0.42±0.07	0.61±0.008	0.23±0.02	0.07±0.03	0.60±0.06	0.69±0.01	0.62±0.02	0.60±0.02

7.2. Cellular accumulation by ICP-MS.

CT-26 cells were seeded in a 6-cm cell culture dish at a density of $1x10^6$ cells/dish and were incubated at 37 °C, 5% CO_2 for 24 h. The medium was replaced with 2 mL of a 5-µM dilution of the Ru(II) complexes in fresh DMEM (DMSO <1 %) and cells were incubated at 37 °C, 5% CO_2 for 4 h. Then, cells were trypsinized, collected, counted and the resulting cell pellets were dried overnight in an oven at 50ºC and stored in a freezer at –80 °C. ICP-MS samples were prepared as follows: cell pellets were digested using 70% nitric acid (500 µL, 65 °C, overnight) and then further diluted 1:100 using a 1% (w/v) solution of HCl in Milli-Q H_2O. All samples were analyzed by ICP-MS using a high-resolution Agilent 7900 quadrupole ICP-MS instrument from the Institut de Physique du Globe de Paris, France. The monitored isotopes were ^{99}Ru and ^{101}Ru. Throughout the course of the analytical sequence, an indium internal standard was injected after in-line mixing with the samples to correct for signal drift and matrix effects. A set of calibration standards were analyzed to confirm and model (through simple linear regression) the linear relationship between signal and concentration. The model was then used to convert measured sample counts to concentrations. Reported uncertainties were calculated using error propagation equations and considering the combination of standard deviation on replicated consecutive signal acquisitions (n=3), internal-standard ratio, and blank subtraction. The non-linear term (internal-standard ratio) was linearized using a first-order Taylor series expansion to simplify error propagation. The amount of metal detected in the cell samples was transformed from ppb to µg of Ru/mL. Data were normalized to the number of cells and expressed as ng of Ru/10^6 cells. Data are expressed as the mean ± SD of one independent experiment with n = 3 replicates.

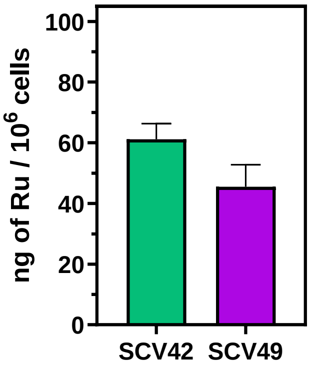

Figure S58. Cellular uptake of the studied compounds by ICP-MS. Intracellular accumulation of **SCV42** and **SCV49** in CT-26 cells after 4 h treatment at 10 µM.

7.3. Lipophilicity determination

Distribution coefficients between octanol and water ($K_{O/W}$) and log *P* values of compounds **SCV42**, **SCV45**, **SCV49** and $[Ru(bpy)_3]Cl_2$ were calculated using the "shake-flask" method (adapted from Ref[33]). To this end, solutions of the studied compounds in *n*-octanol-saturated Milli-Q H_2O (4 mL, final concentration 30 μM) were prepared in centrifuge tubes from a 10 mM stock solution in DMSO. The solutions were sonicated for 5 min in an ultrasonic bath and a 2 mL-aliquot of each solution was reserved in another centrifuge tube. To the remaining 2-mL of the solutions, an equal volume of Milli-Q H_2O-saturated *n*-octanol was added, and the resulting mixtures were vigorously shaken in a vortex for 15 min. Then, the octanol/water mixtures were centrifuged at 7800 rpm for 5 min to separate the phases. The UV-Vis absorption spectra of the Milli-Q H_2O phases, as well as those of the reserved aliquots were registered using a Jasco V-550 UV-Vis spectrophotometer. Log *P* values were calculated according to the following equation (5):

$$\log P = \log(K_{O/W}) = log\left(\frac{A_0 - A}{A}\right) \quad (5)$$

where A_0 refers to the absorbance of the reserved aliquots of the initial aqueous solutions of the studied compounds at their maximum absorption wavelengths (λ_{Abs}(**SCV42**) = 463 nm, λ_{Abs}(**SCV45**) = 460 nm, λ_{Abs}(**SCV49**) = 461 nm) and *A* is the absorbance of the aqueous phase of the corresponding octanol/water mixtures at the same wavelengths.

Table S9. Log P values of **SCV42**, **SCV45**, **SCV49** and $[Ru(bpy)_3]Cl_2$ in octanol/water.

Compound	log P
**SCV42**	– 1.05
**SCV45**	– 0.81
**SCV49**	– 0.88
$\mathbf{[Ru(bpy)_3]Cl_2}$	– 1.42

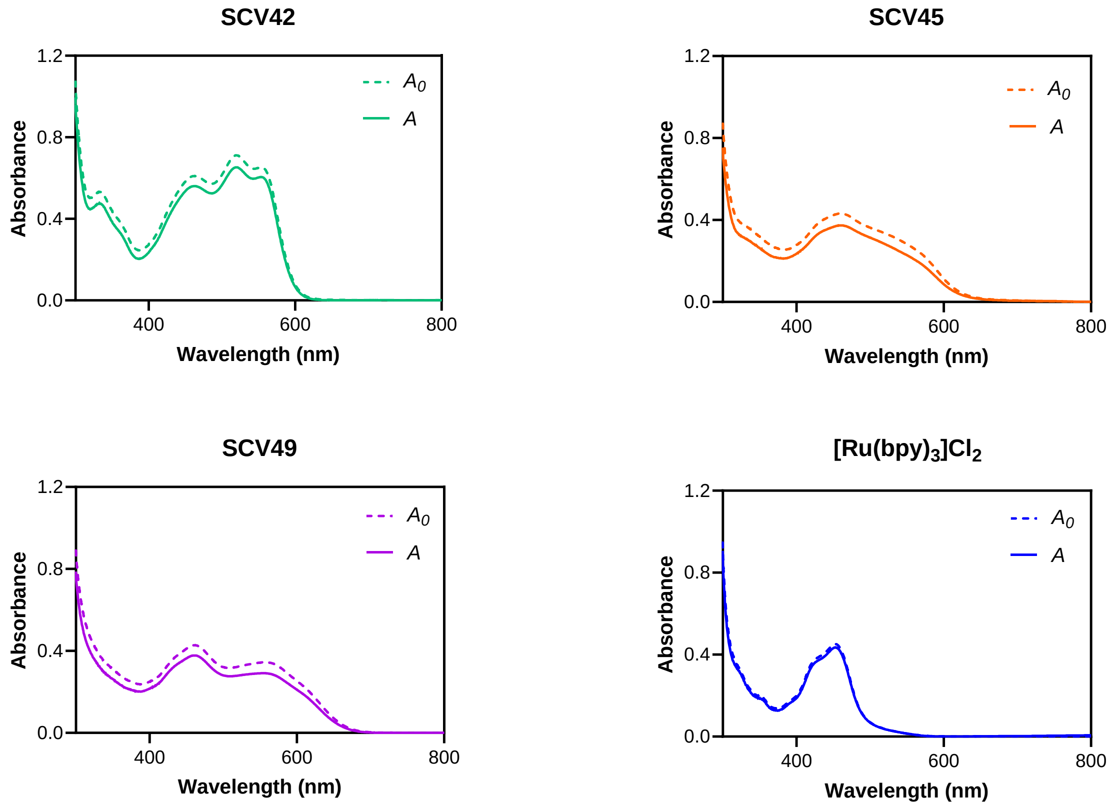

Figure S59. UV-Vis spectra of **SCV42**, **SCV45**, **SCV49** and $[Ru(bpy)_3]Cl_2$ (30 µM) used for log P determination. The spectra of the reserved aliquots (A_0) are shown in dark blue dashed lines, whereas the spectra of the aqueous phases of the O/W mixtures (A) are shown in light blue solid lines.

8. *In vitro* (photo)cytotoxicity determination of Ru-COUBPY PSs

8.1. Cell culture

CT-26 cells were cultured in Dulbecco's modified Eagle medium (DMEM) (Gibco) supplemented with 10% fetal calf serum (Gibco) and 2 mM L-glutamine. For both cell lines, the corresponding culture medium was supplemented with 100 U·mL−1 penicillin streptomycin mixture (Gibco) and cells were maintained in a humidified atmosphere at 37 °C and 5 % of CO_2.

8.2. (Photo)cytotoxicity evaluation in 2D monolayer cells under normoxia (21% O_2)

The (photo)cytotoxicity of the test compounds under normoxia (21% O_2) was assessed by a fluorometric cell viability assay using Resazurin (Acros Organics). CT-26 cells were seeded in triplicate in 96-well plates at a density of 4000 cells/well in complete medium in a final volume of 100 µL/well. After 24 h, cells were treated with increasing concentrations of the test compounds using serial dilutions of the test compounds in complete culture medium (DMSO ≤1 %) at final concentrations in the range of 0 to 250 µM (100 µL/well), followed by a 4-h incubation at 37 ºC, 5% CO_2 in the dark. The medium containing the PS was then replaced with fresh medium (100 µL/well) and plates were irradiated for 1 h at 37 ºC, 0 % CO_2 with 540 nm (spectral half-width: 32 nm, 40 min, 3.75 mW cm^{-2}, 9.0 J cm^{-2}), 595 nm (spectral half-width: 32 nm, 60 min, 0.94 mW cm^{-2}, 3.4 J cm^{-2}), 620 nm (spectral half-width: 32 nm, 60 min, 1.88 mW cm^{-2}, 6.7 J cm^{-2}), 645 nm (spectral half-width: 32 nm, 60 min, 2.50 mW cm^{-2}, 9.0 J cm^{-2}), 670 nm (spectral half-width: 32 nm, 60 min, 3.75 mW cm^{-2}, 13.5 J cm^{-2}) or 740 nm (spectral half-width: 32 nm, 60 min, 3.50 mW cm^{-2}, 12.6 J cm^{-2}) light using a LED multi-well plate photo-irradiation system (Atlas Photonics Lumos Bio). To evaluate the cytotoxicity of the test compounds in the dark, three additional plates of treated cells were kept in the dark for 1 h at 37 °C, 0 % CO_2. Cells were then kept in the incubator (37 °C, 5 % CO_2) over a recovery period of 44 h. Next, the medium was replaced with complete medium containing Resazurin (0.2 mg/mL, 100 µL/well), followed by a 4-h incubation at 37 °C, 5 % CO_2. Finally, the fluorescence signal of the Resorufin product was measured (λ_{ex} = 540 nm, λ_{em} = 590 nm) using a Infinite 200 PRO Microplate Reader from TECAN. IC_{50} values were then calculated based on the inhibitory rate curves using the following equation (6)

$$I = \frac{I_{max}}{1 + \left(\frac{IC_{50}}{C}\right)^n} \quad (6)$$

where I represent the percentage inhibition of viability observed, I_{max} is the maximal inhibitory effect, IC_{50} is the concentration that inhibits 50% of maximal growth, C is the concentration of the treatment and n is the slope of the semi-logarithmic dose-response sigmoidal curves. The non-linear fitting was performed using GraphPad Prism software. All experiments were performed in three independent studies with triplicate points per concentration level.

8.3. (Photo)cytotoxicity evaluation in 2D monolayer cells under hypoxia (2% O_2)

For hypoxia experiments, a Hypoxia condition was set up using nitrogen (N_2) to displace O_2 down to a minimum of 2% in a Forma™ Steri-Cycle™ i160 incubator (ThermoFisher Scientific) and cells were cultured under hypoxia for two weeks prior to experiments. The photocytotoxicity of the test compounds under hypoxia (2% O_2) was assessed by a fluorometric cell viability assay using Resazurin (Acros Organics). Cells were seeded in triplicate in 96-well plates at a density of 2500 cells/well in a final volume of 100 µL/well in the incubator at 2% O_2. A flask containing the corresponding cell culture medium was also kept in the incubator at 2% O_2. After 48 h in the hypoxia incubator, cells were treated with increasing concentrations of the test compounds using serial dilutions of the test compounds in deoxygenated complete culture medium (DMSO ≤1 %) at final concentrations in the range of 0 to 250 µM (100 µL/well), followed by a 4-h incubation (37 ºC, 2% O_2, 5% CO_2). The medium containing the PS was then replaced with fresh medium (100 µL/well). To evaluate the phototoxicity of the compounds under hypoxic conditions, the LED multi-well plate photo-irradiation system (Atlas Photonics Lumos Bio) used to irradiate cells (see above) was placed inside a Glove Box from Plas-Labs (856-Series) equipped with an O_2 concentration sensor. O_2 concentration was maintained at 2% using a nitrogen flow. To evaluate the cytotoxicity of the test compounds in the dark, three additional plates of treated cells were kept in the dark for 1 h in the incubator under hypoxia. Cells were then kept in the incubator (37 ºC, 2% O_2, 5% CO_2) over a recovery period of 44 h. Next, the medium was replaced with complete medium containing Resazurin (0.2 mg/mL, 100 µL/well). After 4 h of incubation (37 °C, 21% O_2, 5% CO_2), the fluorescence signal of the Resorufin product was measured (λ_{ex} = 540 nm, λ_{em} = 590 nm) using an Infinite 200 PRO Microplate Reader from TECAN. IC_{50} values were calculated as indicated in section 8.2.

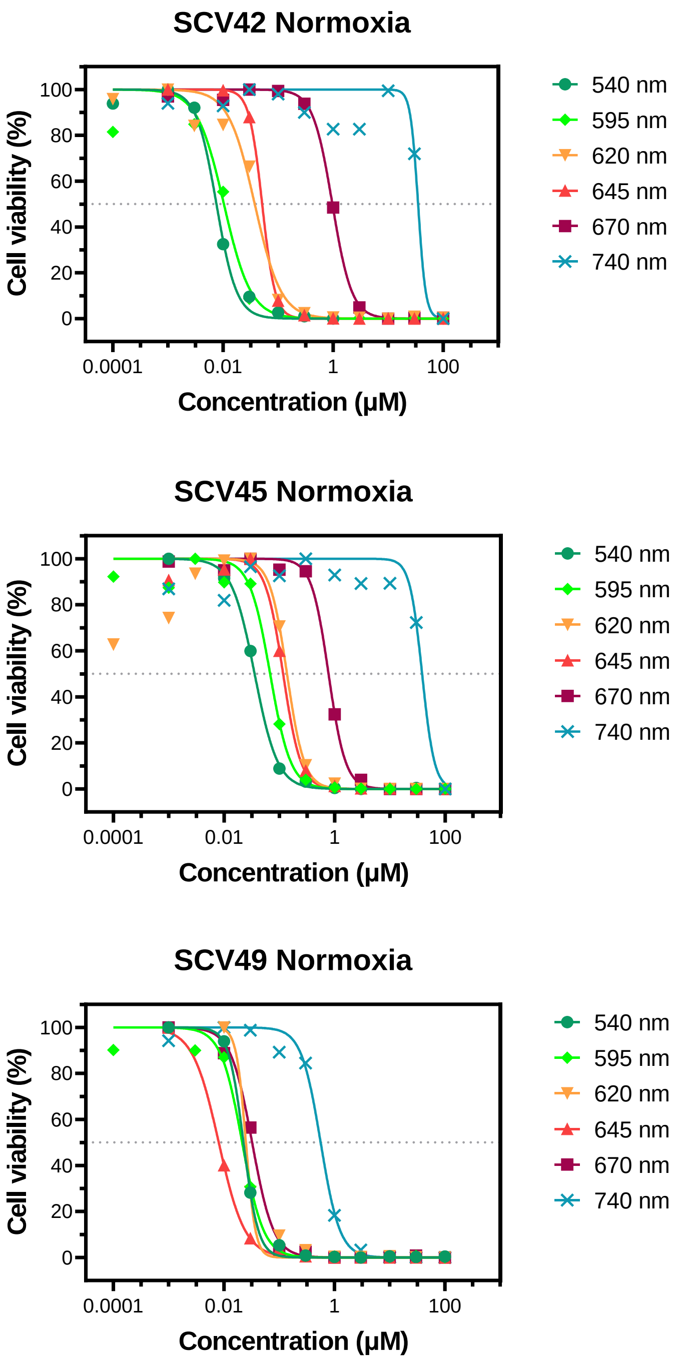

Figure S60. Dose-response curves for chromatic (photo)cytotoxicity screening of **SCV42** (top), **SCV45** (middle), and **SCV49** (bottom) in CT-26 cells under normoxic conditions (21% O_2). Irradiation conditions: 540 nm (40 min, 3.75 mW/cm², 9.0 J/cm²), 595 nm (60 min, 0.94 mW/cm², 3.4 J/cm²), 620 nm (60 min, 1.88 mW/cm², 6.7 J/cm²), 645 nm (60 min, 2.50 mW/cm², 9.0 J/cm²), 670 nm (60 min, 3.75 mW/cm², 13.5 J/cm²) and 740 nm (60 min, 3.50 mW/cm², 12.6 J/cm²).

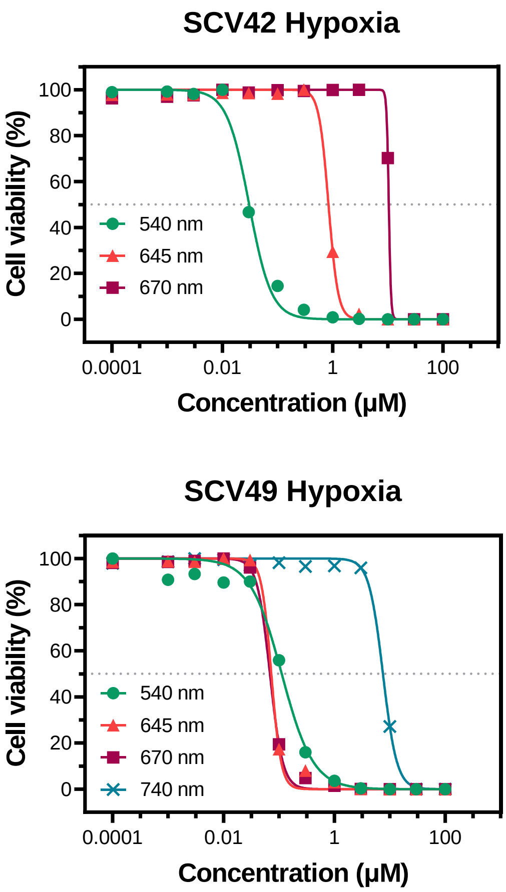

Figure S61. Dose-response curves for chromatic (photo)cytotoxicity screening of **SCV42** (top) and **SCV49** (bottom) in CT-26 cells under hypoxic conditions (2% O_2). Irradiation conditions: 540 nm (40 min, 3.75 mW/cm², 9.0 J/cm²), 645 nm (60 min, 2.50 mW/cm², 9.0 J/cm²), 670 nm (60 min, 3.75 mW/cm², 13.5 J/cm²) and 740 nm (60 min, 3.50 mW/cm², 12.6 J/cm²).

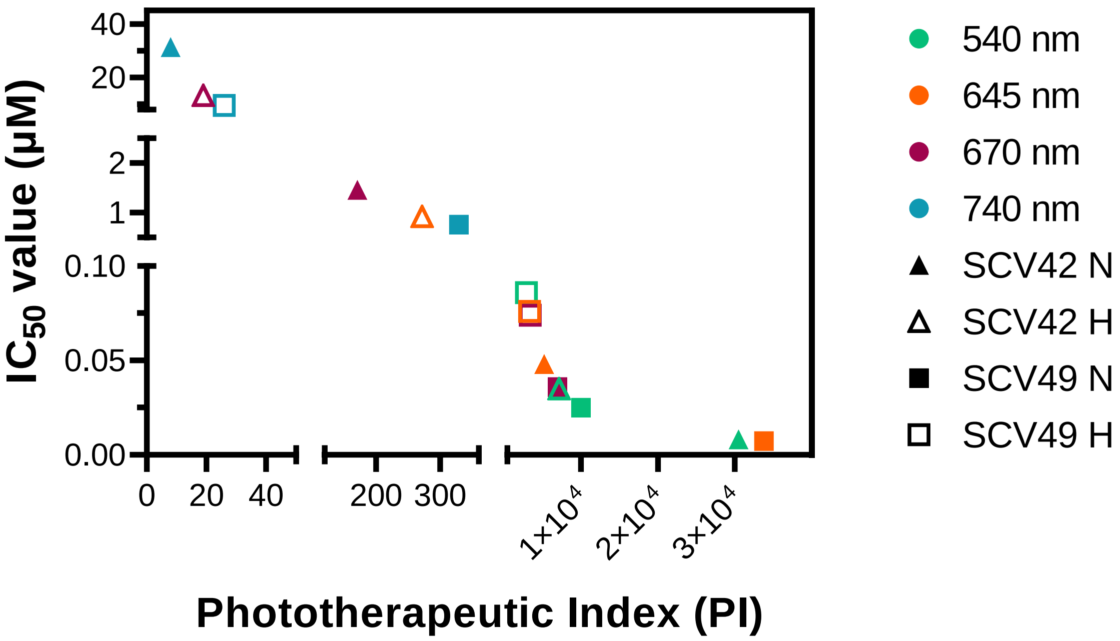

Figure S62. (Photo)cytotoxicity screening of compounds **SCV42** and **SCV49** in CT-26 cells following green (540±32 nm, 40 min, 3.75 mW cm^{-2}, 9.0 J cm^{-2}), deep red (645±32 nm, 1 h, 2.50 mW cm^{-2}, 9.0 J cm^{-2}), far-red (670±32 nm, 1 h, 3.75 mW cm^{-2}, 13.5 J cm^{-2}) or NIR (740±32 nm, 1 h, 3.50 mW cm^{-2}, 12.6 J cm^{-2}) light irradiation, under normoxic (20 % O_2) and hypoxic (2 % O_2) conditions, represented as IC_{50} and phototherapeutic index (PI) values.

9 (Photo)cytotoxicity evaluation of SCV49 on 3D multicellular tumor spheroids

For the generation of CT-26 multicellular tumor spheroids (MTCSs), U-bottom 96-well Corning microplates with ultra-low attachment surface coating were used. A suspension of CT-26 cells at a density of 10^5 cells/mL was prepared in complete DMEM medium and dispensed into wells (100 µL/well, 10.000 cells/well). The plates were covered and transferred to an incubator at 37 ºC with 5% CO_2 atmosphere. Within 3 days, uniform MTCSs with a diameter of *ca.* 500 µm were formed from the cell suspension maintained under the same conditions. On day 0 (*i.e.* after the MCTS were formed), MCTSs were treated with serial dilutions of **SCV49** in DMEM (DMSO ≤1 %) at final concentrations in the range of 0 to 100 µM (100 µL/well), followed by a 36-h incubation at 37 ºC, 5% CO_2. The medium containing the compound was then replaced with fresh medium (100 µL/well) and plates were irradiated with deep-red (645 nm, 2.50 mW·cm^{-2}, 60 min, 9.00 J·cm^{-2}) at 37 ºC, 0 % CO_2 using a LED multi-well plate photo-irradiation system (Atlas Photonics Lumos Bio). MCTSs were further incubated for 5 days (37 °C, 5% CO_2), replacing half of the volume of the medium in the wells with fresh medium once every 2 days. The formation, integrity and diameter of the MCTSs were monitored using a BioTek Cytation 5 cell imaging multimode plate reader (Agilent Technologies, Inc.) over a span of 7 days.

10. *In vivo* pharmacokinetic (PK) study of SCV49 in CD1 mice

10.1. Environment and husbandry

The *in vivo* PK study was carried out with 21 male CD1 mice (30 grams aprox.), which were supplied by Envigo. Upon arrival, animals were housed in groups of 3 animals/cage. Cages type GM-500, 501 cm^2 (Tecniplast). Absorbent bedding: Safe® select fine (Renttenmaier Ibérica S.L). Animals were kept in an environmentally controlled room (ventilation, temperature 22 ± 3ºC and humidity 35-70%) on a 12 h light/dark cycle. A period of 6 days of acclimatization underwent between the date of arrival and the start of the procedure. During this period the animals were observed to check their general health state. The maintenance diet was supplied by Renttenmaier Ibérica S.L (A40). Diet was provided to the animals *ad libitum*. Diet was analysed by the manufacturer to detect possible contaminants. Water was supplied by Aigües de Barcelona and given to the animals by bottles.

10.2. Animal Welfare

The animals were maintained in accordance with: European Directive for the Protection of Vertebrate Animals Used for Experimental and other Scientific Purposes (86/609/EU). Decree 214/1997 of 30th July. Ministry of agriculture, livestock and fishing of the Autonomous Government of Catalonia, Spain. Royal Decree 53/2013 of 1st February (Spain).

All the experimental procedures were approved by the Animal Experimentation Ethical Committee of Parc Científic de Barcelona (PCB) and by the Animal Experimentation Commission of the Generalitat de Catalunya. DAAM: 10527.

10.3. Administration

All animals were weighted and identified with permanent marker (tail code numbers). **SCV49** was intraperitoneally administered (5 mg/kg) at 0.5 mg/mL in 2.5% DMSO + 0.1% Tween-80 + 0.5% CMC. Administration volume was 10 mL/kg.

10.4. Sampling

At different timepoints after administration of the compound (0.16, 0.5, 1, 2, 4, and 24 h), animals (3 mice/group) were anesthetized with isoflurane. Blood was collected through intracardiac punction in an Eppendorf tube containing K_2-EDTA. Blood was centrifuged at 10,000 rpm for 5 minutes for plasma collection. Plasma was stored at -80 ºC until analysis. Animals were sacrificed by bleeding. After cutting both jugular veins, the aorta was clamped, and the animal was perfused with 10 mL of PBS at 4 ºC through the left ventricle. Several

organs were removed (brain, lung, kidneys, liver, bladder, and spleen), weighted, and frozen with liquid N_2. Tissues were stored at -80 ºC until analysis.

To evaluate the amount of Ru accumulated in the collected tissues (brain, lung, kidneys, liver, bladder and spleen) of mice after different times of administration with respect to the administered dose, samples were digested with 70% nitric acid (110 °C, overnight for all organs except for liver which required 48 h) and then analyzed by ICP-MS after being diluted 1:100 using a 1% (w/v) solution of HCl in Milli-Q H_2O. In the case of liver samples corresponding to 1 h and 24 h timepoints, they were divided into two parts to allow analysis by both ICP-MS and UPLC-MS/MS.

Table S10. Mean of concentrations of **SCV49** in albino swiss adult male CD1 mice plasma at different times after IP administration at 5 mg/Kg.

**Time (h)**	**[SCV49] in plasma (ng/mL)**
0.167	978.7 ± 158
0.5	5276.7 ± 1029
1	4960 ± 402
2	4396.7 ± 951
4	1050.3 ± 447
24	49.9 ± 8.2

Table S11. Pharmacokinetic parameters of **SCV49** in mouse plasma (bottom). after IP administration at 5 mg/Kg

**Pharmacokinetic parameters**	
T_{max} (h)	0.5
C_{max} (ng/mL in plasma)	5276.7
AUC_{last} (h·ng/mL in plasma)	24802
AUC_{inf} (h·ng/mL in plasma)	25064
$t_{1/2}$ (h) - elimination	3.63
k_e (h^{-1})	0.191
Vd (L/kg)	1.04
Cl (L/h·kg)	0.20

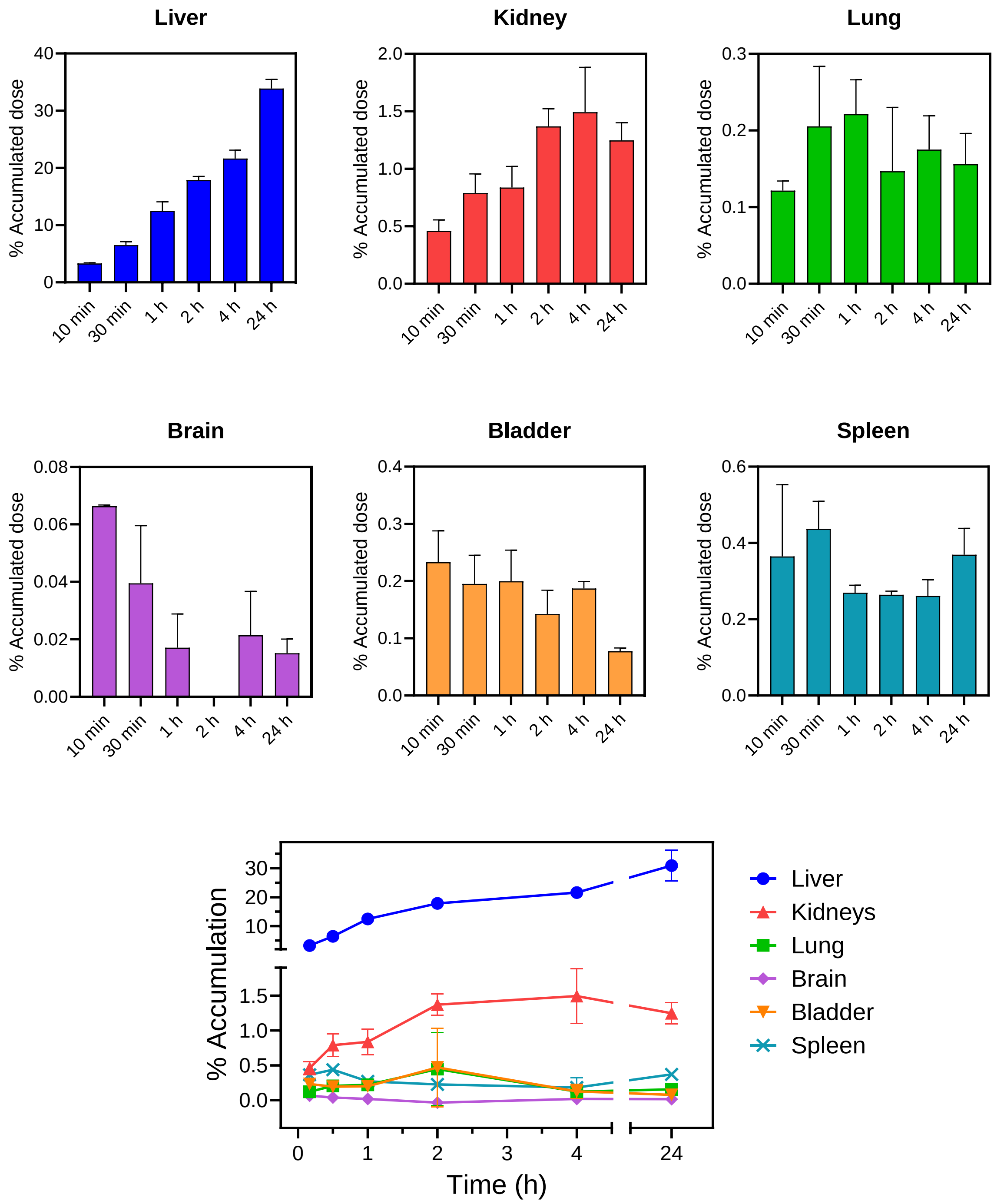

Figure S63. Biodistribution of Ru content (% accumulated Ru with respect the administered dose, n= 3) in major organs of albino swiss adult male CD1 mice at the different timepoints of the PK study following IP administration of **SCV49** (5 mg/kg). Ru content was quantified by ICP-MS.

Table S12. Biodistribution of Ru content and of the intact **SCV49** (% accumulated Ru or **SCV49** with respect the administered dose, respectively, n= 3) in liver of albino swiss adult male CD1 mice at 1 h or 24 h following IP administration of **SCV49** (5 mg/kg). Ru and **SCV49** content were quantified by ICP-MS and UPLC-MS/MS, respectively.

	**% administered dose accumulated in liver**	
**Time**	**ICP-MS**	**UPLC-MS/MS**
1 h	12.5 ± 1.6	19.4 ± 3.2
24 h	30.9 ± 5.3	38.2 ± 19

11. *In vivo* toxicological study of SCV49 in CD1 mice

11.1. Environment and husbandry

The *in vivo* toxicological study was carried out with 18 male CD1 mice 5 weeks old (9 female and 9 male of 20 and 25 g aprox., respectively), which were supplied by Envigo. Upon arrival, animals were housed in groups of 3 animals/cage. Cages type GM-500, 501 cm^2 (Tecniplast). Absorbent bedding: Safe® select fine (Renttenmaier Ibérica S.L). Animals were kept in an environmentally controlled room (ventilation, temperature 22 ± 3ºC and humidity 35-70%) on a 12 h light/dark cycle. A period of 6 days of acclimatization underwent between the date of arrival and the start of the procedure. During this period the animals were observed to check their general health state. The maintenance diet was supplied by Renttenmaier Ibérica S.L (A40). Diet was provided to the animals *ad libitum*. Diet was analysed by the manufacturer to detect possible contaminants. Water was supplied by Aigües de Barcelona and given to the animals by bottles.

11.2. Animal Welfare

The animals were maintained in accordance with: European Directive for the Protection of Vertebrate Animals Used for Experimental and other Scientific Purposes (86/609/EU). Decree 214/1997 of 30th July. Ministry of agriculture, livestock and fishing of the Autonomous Government of Catalonia, Spain. Royal Decree 53/2013 of 1st February (Spain).

All the experimental procedures were approved by the Animal Experimentation Ethical Committee of Parc Científic de Barcelona (PCB) and by the Animal Experimentation Commission of the Generalitat de Catalunya. DAAM: 10527.

11.3. Formulation and compound administration

All animals were weighted and identified with permanent marker (tail code numbers). **SCV49** was intraperitoneally administered to CD1 mice at two doses (10 or 30 mg/kg). Administration volume was 10 mL/kg. First, **SCV49** was dissolved in 2.5% DMSO, vortexed and sonicated during at least 15 minutes to obtain a homogenous suspension. Thereafter, the vehicle (0.1 % Tween 80 + 0.5% CMC (99%)) was added to obtain the high concentration (3 mg/mL) to be administered to the animals. Low concentration was obtained from 3 mg/mL (3-fold dilution in the vehicle). The two solutions were protected from light and maintained in constant agitation during the administrations to all the animals.

11.4. Experimental procedure

-On day 1, animals were randomized into the different experimental groups and identified by tail code numbers (1-18) and weighed before the first administration. Animals were intraperitoneally administered (volume: 10 mL/kg) with the corresponding vehicle or test item dose (10 or 30 mg/kg). Food consumption and clinical signs were monitored and recorded as well. Special monitoring of clinical signs was recorded for 1 hour following the first administration and at 2, 3 and 4 hours after administration.

-On day 2, day 3 and day 4, body weight was registered. Food consumption and clinical signs were monitored and recorded too.

-On day 5, body weight and food consumption were registered. Then, animals were deeply anesthetized with isoflurane and blood was collected by vena cava puncture (collected on recipients containing EDTA.2K 5%). Macroscopic exploration of the main organs (thymus, heart, lungs, spleen, liver, kidneys, stomach, intestines, adrenal, testes/uterus:ovaries, prostate-vesicles, urinary tract and brain) was carried out to identify target organs for toxicity. 100 µL of blood was used for haematological analysis and the remaining blood was centrifuged at 10.000 rpm for 5 minutes to obtain plasma that was stored at -80ºC for biochemical analysis.

Table S13. Experimental groups of the *in vivo* toxicological study.

Group	Treatment	Route	Dose (mg/kg)	n	Males	Females
1	Vehicle	IP	-	6	3	3
2	**SCV49**	IP	10	6	3	3
3	**SCV49**	IP	30	6	3	3

11.5. Data processing and statistical analysis

Raw data was tabulated, expressed as mean ± SD, and analysed by using the appropriate statistical test. Significance for all tests was set at $p \leq 0.05$. The statistical analysis was made using Graph Pad Prism version 9.5.1. Body weight increase/decrease was analyzed using Two-way analysis of variance (2-way ANOVA), followed by Bonferroni's post-test. Organ weight/Body weight ratio and haematological results of white blood cells and red blood cells were determined and analyzed using One-way analysis of variance (1-way ANOVA), followed by Bonferroni's post-test.

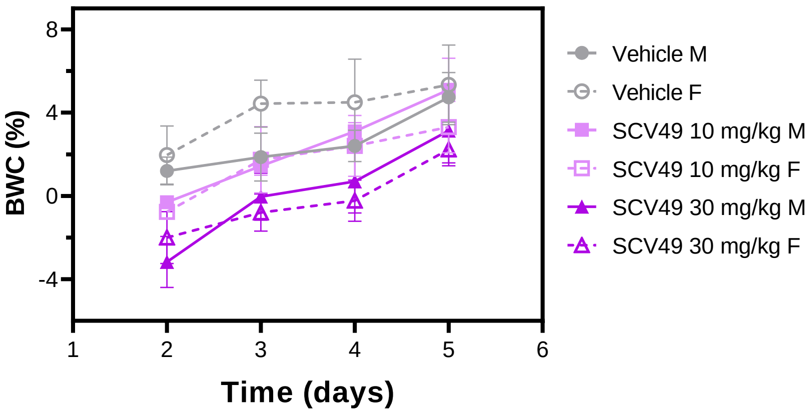

Figure S64. Body weight change relative to day 1 for animals treated intraperitoneally with vehicle or **SCV49** (10 or 30 mg/kg) on day 1, with sacrifice on day 5. Results are presented as mean ± SD (n = 3 males; n = 3 females).

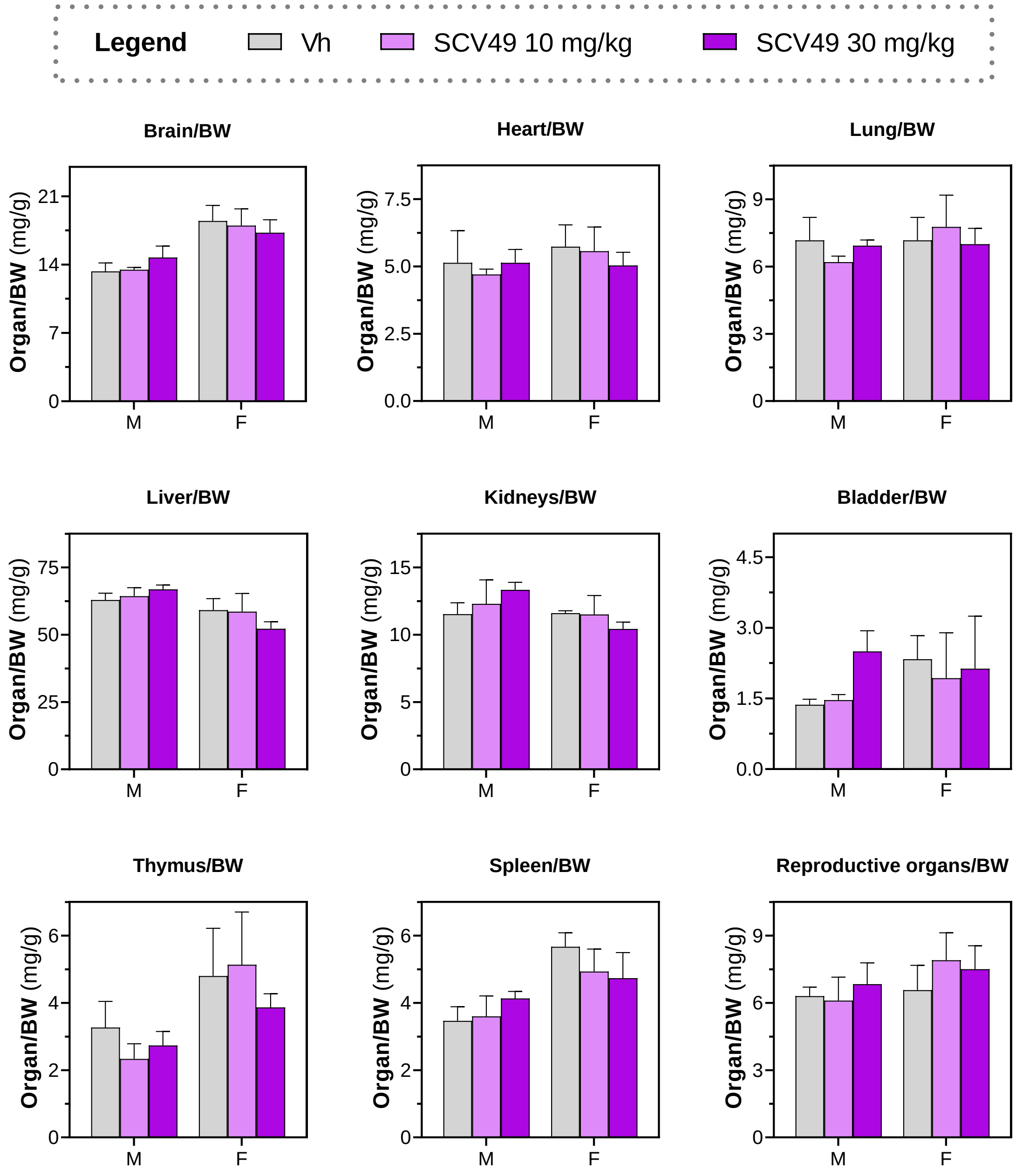

Figure S65. Organ weight (mg)/Body weight (g) ratio of animals intraperitoneally treated with Vehicle and **SCV49** (10 or 30 mg/kg) on day 1 and sacrifice on day 5. Results are expressed as mean ± SD (n=3 Males; n=3 Females).

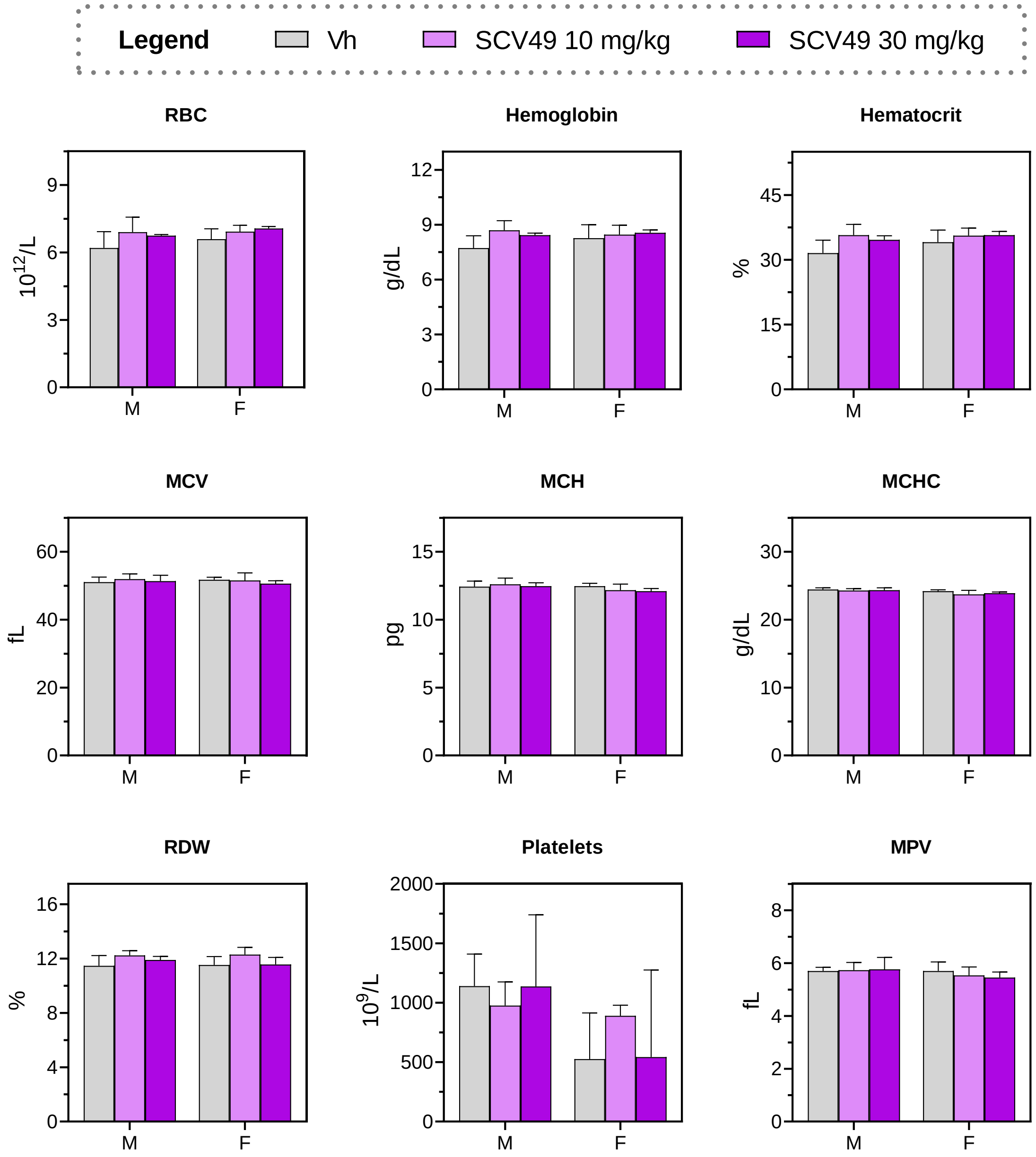

Figure S66. Hematology analysis of red blood cells and platelets from animals treated intraperitoneally with vehicle or **SCV49** (10 or 30 mg/kg) on day 1, with sacrifice on day 5. Results are expressed as mean ± SD (n = 3 males; n = 3 females). RBC parameters: red blood cells (RBC), mean corpuscular volume (MCV), mean corpuscular hemoglobin (MCH), mean corpuscular hemoglobin concentration (MCHC), and red cell distribution width (RDW). Platelet parameter: mean platelet volume (MPV).

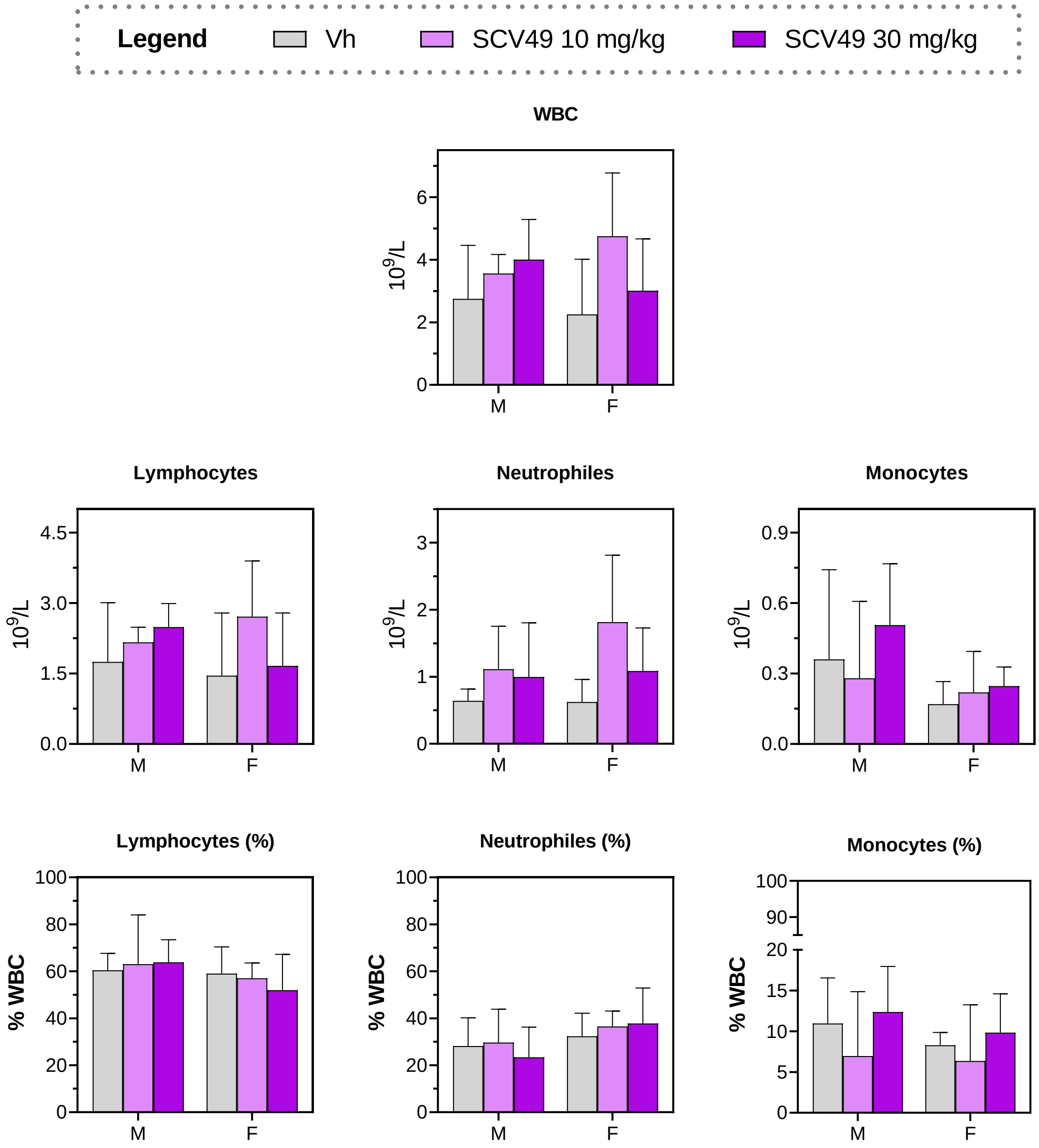

Figure S67. Hematology analysis of white blood cells from animals treated intraperitoneally with vehicle or **SCV49** (10 or 30 mg/kg) on day 1, with sacrifice on day 5. Results are expressed as mean ± SD (n = 3 males; n = 3 females). WBC parameters: white blood cells (WBC), lymphocytes (LYM), neutrophils (NEU), monocytes (MON), basophils (BAS), and eosinophils (EOS).

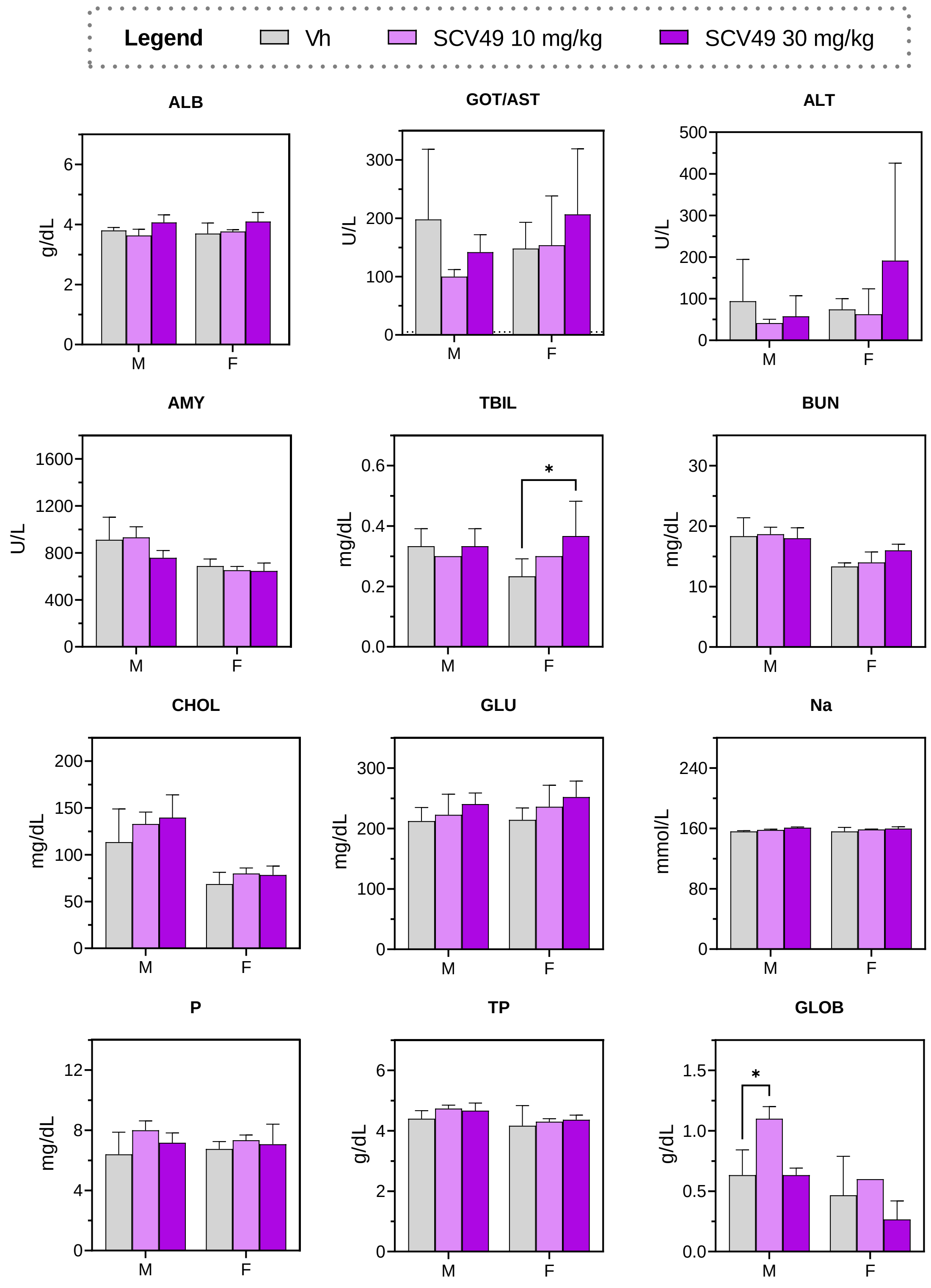

Figure S68. Results from biochemical analysis of animals intraperitoneally treated with Vehicle and **SCV49** (10 or 30 mg/kg) on day 1 and sacrifice on day 5. Results are expressed as mean ± SD (n=3 Males; n=3 Females). ALB: albumin; GOT/AST: glutamic-oxaloacetic transaminase; ALT; alanine transaminase; AMY: amylase; TBIL; total bilirubin; BUN: blood

urea nitrogen; CHOL: cholesterol; GLU: glucose; P: phosphorus; TP: total protein; GLOB: globulin.

12. *In vivo* PDT efficacy study of SCV49 in BALB/c mice bearing subcutaneous CT-26 syngeneic colon tumors

12.1. Ethical animal procedures and animal housing conditions

Animal experimentation was performed in accordance with local, national and European legislation. All the procedures were reviewed by the Parc Científic de Barcelona (PCB) Ethical Committee for Animal Experimentation, with the authorization number 23-044-P1-(SC). Animals used for this study were maintained in the animal facility of the Parc Científic de Barcelona. This animal facility is registered by the regional competent authority to house and maintain animals used by scientific procedures (B-9900044) and by National Competent authority (to use genetically modified organisms (A/ES/16/1-03; Notification date: 65-7951/2016). Animals were housed under sterile conditions at a constant temperature of 20-22°C and relative humidity (45-65%) under daily cycles of light/darkness (12 hours). Manipulation was performed in laminar flow hood and sterilized water and food will be available *ad libitum*. For animal identification, a chip was inserted in the back-front of each mouse.

12.2. Tumor cell line

CT-26 (CT26.WT ATCC ® CRL-2638™), a colon carcinoma cell line, was grown in RPMI-1640 (Thermo Fisher, 42401042), supplemented with 10% FCS (Gibco, 10106-169) at 37°C and 5% CO_2. Cells were expanded when reached 90% confluence. Viability was monitored before and after injection, that should be >90%.

12.3. Formulation of the compound

The corresponding volume of DMSO was added to a vial containing the required amount of **SCV49**, sonicated in an ultrasonic bath at 37 °C for 2 min and vortexed homogenized. Then, the corresponding volume of DMEM was added, sonicated in an ultrasonic bath at 37 °C for 3 min and vortexed homogenized. The vial was maintained at 37 °C until it was administered.

12.4. Light tolerability test

Four BALB/c mice bearing subcutaneous CT-26 syngeneic colon tumors after sorting (see section 10.5 for further details) were used for irradiation tolerability test in the right dorso-lateral side by using deep-red light (660 ± 20 nm, 100 mW cm^{-2}). The irradiation device was assembled by CD6 (Spain) and integrates an M660L4 LED (SN M00929533) as a light source which was regulated by an LEDD1B power supply (SN M00936983), both from Thorlabs, Inc. 2 mice received 10 min of irradiation on day 1 and 15 min on day 3, and the other 2 mice

received 15 min of irradiation on day 1 and 20 min on day 3. Animals were kept one week more to monitor any clinical sign associated to the irradiation.

12.5. *In vivo* PDT efficacy study

Animals (8-week-old BALB/c female mice from Envigo) were anesthetized with isoflurane and CT-26 cells were injected subcutaneously in the right dorso-lateral side at $1.15x10^6$ cells/inoculation in 100 µL of RPMI. To have a reduced deviation, 65 mice were initially injected with cells. When tumor size reached 50-100 mm^3 (at day 10, the mean tumor volume was 83.49 mm^3), mice were randomly divided into 7 groups (n=5/group). Then, each group of mice were injected with the corresponding treatment and dose, as it is described in the Table 3.

Vehicle: DMEM+1% DMSO
Injection: Bolus administration for all treatments, using a 100 µL Hamilton syringe equipped with a 29G needle for IT administration.
Conditions: Mice were anesthetized with isoflurane during the treatment.
Volume dose:

Group 1, 40 µL vehicle/mouse (administered during 2 min).
Group 2, 40 µL vehicle/mouse (administered during 2 min).
Groups 3 to 5, 40 µL 3 mg **SCV49**/kg mouse /day (administered during 2 min).
Groups 6 and 7, 40 µL 6 mg **SCV49**/kg mouse /day (administered during 2 min).

In groups 2 and 4, the tumor was irradiated 15 min after compound administration (20 min, 660 nm LED source, 100 mW cm^{-2}) on day 1 and day 3. In groups 5 and 7 the tumor was irradiated 5 min after compound administration (20 min, 660 nm LED source, 100 mW cm^{-2}), daily for 4 days (group 5) and on day 1 and day 3 (group 7).

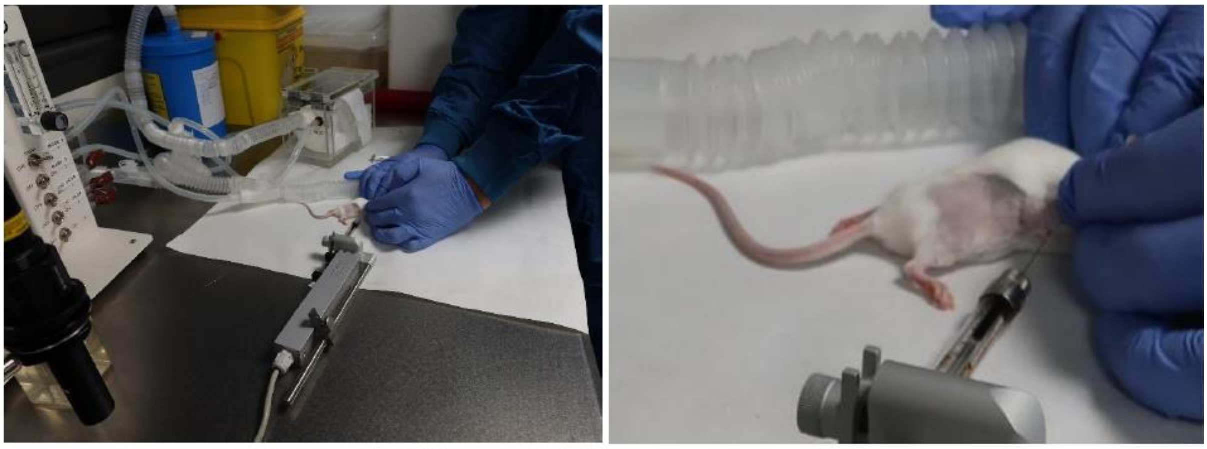

Figure S69. Images of IT administration of **SCV49**.

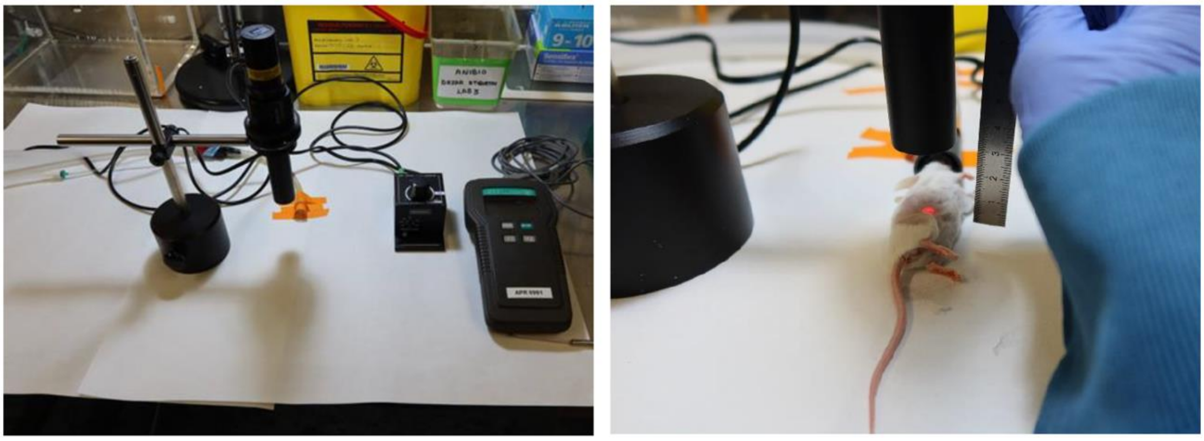

Figure S70. Workstation for irradiation system in mice.

After dose administration, all the animals were observed carefully for treatment-related clinical signs, including morbidity and mortality. Attention was paid to determine the toxic reactions, their severity and time of onset and length of recovery period. Observations included but not limited to evaluation of changes in skin, fur, eyes, mucous membranes and also respiratory, circulatory, autonomic, central nervous systems, somatomotor activity and behaviour pattern. At the time of routine monitoring, the animals were checked for any effects on normal behaviour such as mobility, visual estimation of food and water consumption, eye/hair matting and any other abnormal effect.

Body weight was monitored before drug administration and every 2-3 times per week along the experiment. The body weight loss was calculated according to the formula (7):

$$\frac{\text{weight} - \text{weightstart}}{\text{weightstart}} * 100 \ (7)$$

Tumor volume was measured twice a week using a caliper. Usually, tumors are not completely spherical, so volume was interpolated from the measure of maximum and minimum diameter according to the formula: $Dxd^2\!/2$

Relative tumor volume (RTV) was calculated according to the formula (8):

$$RTV = \frac{\text{volume day X}}{\text{volume day 1}} * 100 \ (8)$$

Percentage of tumor growth inhibition (%TGI), which was used for the evaluation of antitumor efficacy, was calculated with the following formula (9):

$$TGI = \frac{1 - \ (\text{change of tumor volume in treatment group})}{\text{change of tumor volume in control group}} * 100 \ (9)$$

Tumor growth over time was recorded. Experimental data was recorded and analyzed using Prism 9.0 for windows from GraphPad Software Inc.

The study was finished when tumors of any group reached an average volume of 1500 mm^3. At sacrifice, mice were euthanized with CO_2 and blood samples were collected by cardiac puncture using labelled micro centrifuge tubes containing EDTA as anticoagulant and centrifuged at 4°C for 10 min at 5000 rpm to obtain plasma and stored at -70°C until biochemical analysis.

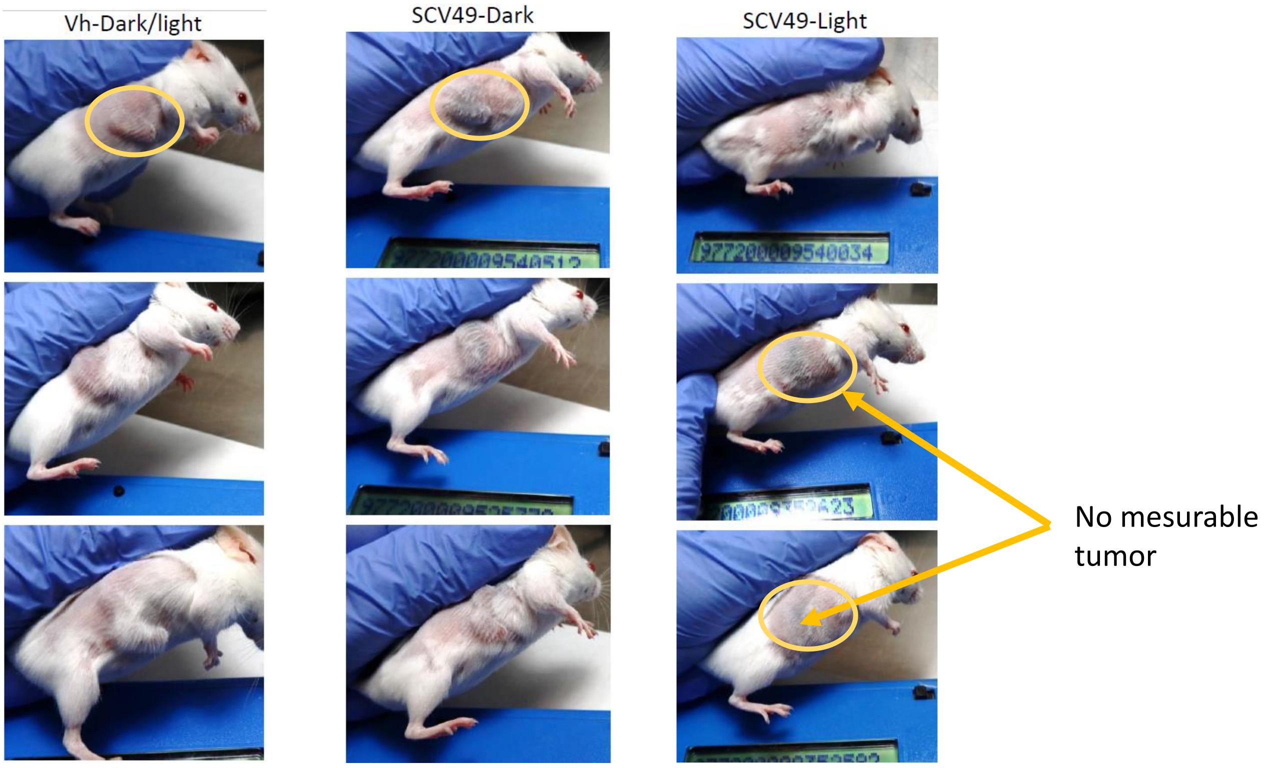

Figure S71. Images of animals 4 days after treatment (groups 1 to 4).

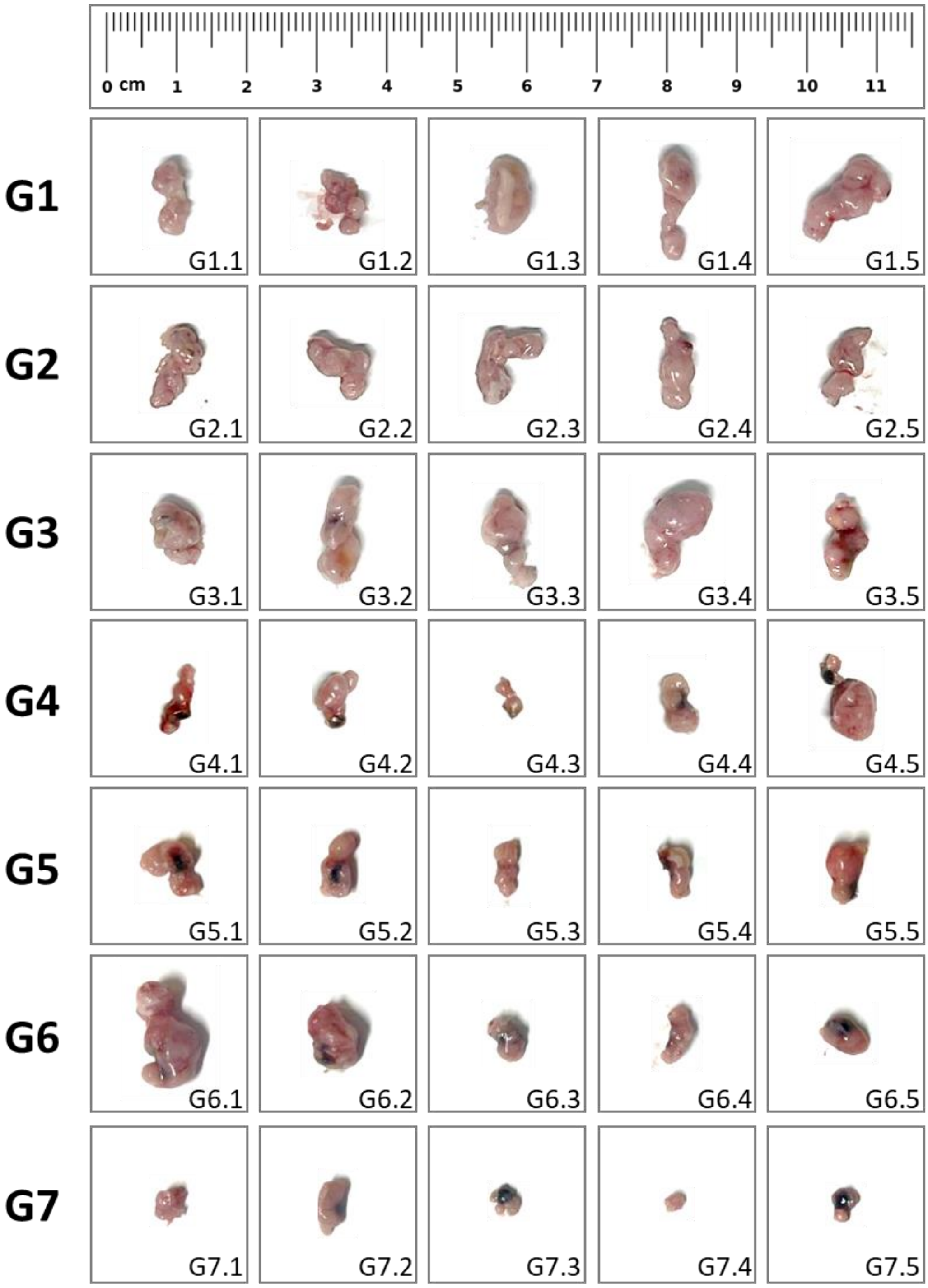

Figure S72. Images of tumors at the endpoint. Group 1: vehicle dark; Group 2: vehicle light (2x); Group 3: **SCV49** 3 mg/kg dark; Group 4: **SCV49** 3 mg/kg light (2x); Group 5: **SCV49** 3 mg/kg light (4x); Group 6: **SCV49** 6 mg/kg dark; Group 7: **SCV49** 6 mg/kg light (2x).

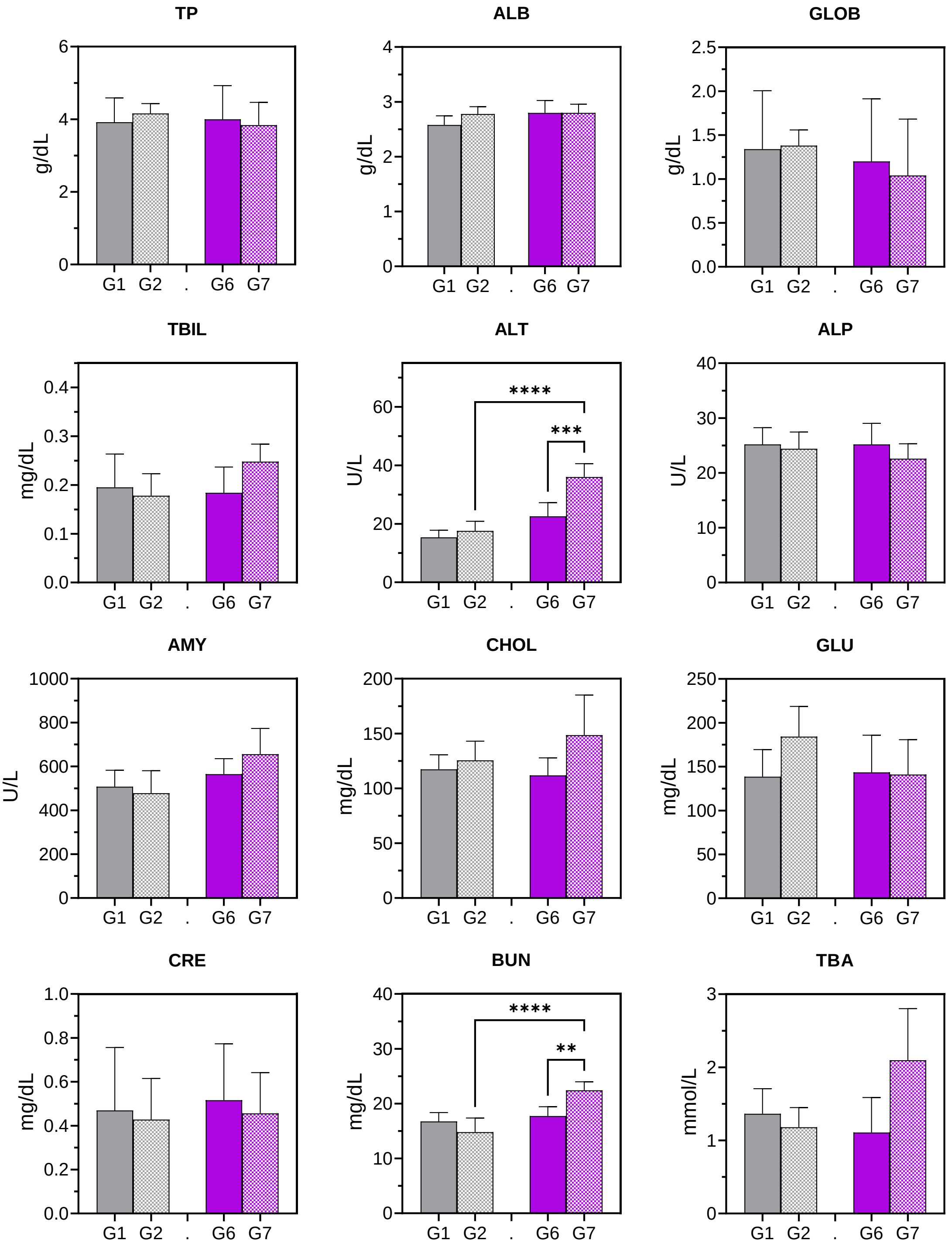

Figure S73. Results from biochemical analysis of plasma samples from animals intratumorally treated with Vehicle or **SCV49** (6 mg/kg) on day 1, and either irradiated or not, and sacrifice on day 9. Results are expressed as mean ± SD (n=5). TP: total protein; ALB: albumin; GLOB: globulin; TBIL: total bilirubin; ALT; alanine transaminase; ALP: alkaline phosphatase; AMY: amylase; CHOL: cholesterol; GLU: glucose; CRE: creatinine; BUN: blood urea nitrogen; TBA: total bile acids.

13. Cartesian Coordinates of the Ru-COUBPY complexes

Table S14. Cartesian coordinates of Ru-COUBPY complexes optimized with the PBE0/6-31+G(d,p)/SDD method in ACN.

SCV42

C	-3.455128	-2.991497	-0.787603
N	-2.928166	-1.795934	-1.155581
C	-3.242598	-4.138671	-1.550943
C	-2.187933	-1.724504	-2.272851
C	-2.477529	-4.058681	-2.707605
C	-1.940445	-2.828295	-3.075709
C	-4.247418	-2.964645	0.450536
C	-4.898243	-4.077155	0.982191
N	-4.306316	-1.756399	1.066503
C	-5.620837	-3.948364	2.161503
C	-5.008099	-1.636821	2.204288
C	-5.677080	-2.704684	2.784158
C	-0.822105	1.205118	-0.466355
N	-2.122759	1.166206	-0.848343
C	0.056555	2.146459	-0.996151
C	-2.551258	2.049748	-1.763315
C	-0.378673	3.069553	-1.946776
C	-1.721489	3.001359	-2.331031
C	-0.440296	0.193151	0.534323
C	0.860617	0.027853	0.988407
N	-1.464802	-0.574551	0.982998
C	1.169141	-0.952579	1.950174
C	-1.188893	-1.505939	1.911360
C	0.077800	-1.717639	2.413763
C	0.554717	4.076519	-2.542515
C	2.502119	-1.189768	2.484192
C	2.587296	-2.015491	3.632502
C	3.724815	-0.730043	1.976568
O	3.671713	-0.009996	0.841939
C	4.988667	-0.990570	2.563109
C	4.797468	0.465862	0.230089
C	6.150956	-0.534335	1.993233
C	6.067022	0.219216	0.779635
C	7.476984	-0.815571	2.621699
C	4.592255	1.175222	-0.936853
C	5.695991	1.707990	-1.640884
C	7.162173	0.741874	0.061219
C	6.994692	1.452164	-1.104946
N	5.526639	2.444436	-2.773387
C	6.669754	2.901828	-3.555584
C	4.194267	2.689876	-3.309463
C	-5.049968	1.897547	1.109101
N	-3.877535	1.282247	1.407617
C	-5.527329	2.953544	1.884444
C	-3.169060	1.703736	2.466647
C	-4.791234	3.384296	2.980966
C	-3.589917	2.747050	3.278086
C	-5.748503	1.362181	-0.068861
C	-6.982946	1.828083	-0.519457
N	-5.099294	0.358932	-0.713160
C	-7.559984	1.256862	-1.646480
C	-5.662388	-0.190760	-1.800429
C	-6.886993	0.228208	-2.299527
Ru	-3.304585	-0.223183	0.121529
H	-3.666758	-5.087596	-1.246010
H	-1.791961	-0.746455	-2.520994
H	-2.304161	-4.944206	-3.310060
H	-1.336285	-2.715537	-3.968942
H	-4.845044	-5.036159	0.481225
H	-6.131682	-4.806493	2.585558
H	-5.021645	-0.651726	2.656220
H	-6.228475	-2.554007	3.705481
H	1.086155	2.176029	-0.658994
H	-3.597091	1.981656	-2.040695
H	-2.128552	3.691037	-3.063414
H	1.637996	0.665688	0.600544
H	-2.026504	-2.100677	2.257954
H	0.214862	-2.491462	3.161259
H	0.956184	3.700431	-3.490732
H	0.037690	5.015500	-2.756200
H	1.398007	4.280807	-1.878449
N	2.637053	-2.695970	4.579296
H	5.015139	-1.564498	3.481851

H	8.118717	-1.378211	1.934745
H	7.999055	0.118011	2.858286
H	7.364119	-1.392683	3.541001
H	3.576184	1.288000	-1.289745
H	8.168837	0.577892	0.432497
H	7.877367	1.818303	-1.613181
H	-6.463102	3.439234	1.635736
H	-2.241946	1.176789	2.660460
H	-5.151451	4.204930	3.592468
H	-2.980218	3.047134	4.122943
H	-7.493092	2.627705	0.003796
H	-8.520155	1.610680	-2.007175
H	-5.101838	-0.985778	-2.278517
H	-7.296798	-0.248840	-3.182721
C	3.617428	1.532492	-4.120226
H	3.585524	0.608430	-3.535233
H	4.214840	1.345768	-5.016779
H	2.596349	1.769956	-4.436096
H	3.525185	2.951105	-2.482401
H	4.256839	3.584158	-3.935386
C	7.299800	1.820906	-4.428960
H	7.632274	0.966926	-3.830966
H	8.169495	2.226752	-4.955433
H	6.589031	1.455724	-5.175434
H	7.416993	3.336917	-2.882851
H	6.317999	3.725079	-4.182664

SCV45

C	-2.448659	-2.565333	-0.987843
N	-2.170309	-1.244696	-1.130705
C	-1.799305	-3.526057	-1.762000
C	-1.253287	-0.863840	-2.033761
C	-0.849131	-3.126468	-2.693421
C	-0.571499	-1.769730	-2.832863
C	-3.466720	-2.878444	0.026283
C	-3.932113	-4.166219	0.288896
N	-3.941250	-1.807545	0.712777
C	-4.899123	-4.358129	1.267570
C	-4.877808	-1.998180	1.654878
C	-5.381628	-3.253298	1.963242
C	-0.914418	1.895275	0.200860
N	-2.128709	1.725518	-0.379264
C	-0.141810	3.023041	-0.056477
C	-2.571284	2.665911	-1.227277
C	-0.583704	3.997473	-0.952656
C	-1.834392	3.796058	-1.543034
C	-0.499674	0.805539	1.099040
C	0.777134	0.702249	1.626809
N	-1.457020	-0.122287	1.353767
C	1.121745	-0.373284	2.462607
C	-1.163598	-1.110231	2.214825
C	0.082463	-1.261073	2.793738
C	0.264815	5.186020	-1.279949
C	2.475450	-0.565267	2.968861
C	2.629234	-1.236845	4.201884
C	3.640583	-0.226625	2.269322
O	3.451561	0.247892	1.025314
C	4.963027	-0.381697	2.748195
C	4.489302	0.552029	0.190007
C	6.045193	-0.068558	1.961368
C	5.816155	0.411582	0.632795
C	7.438375	-0.227889	2.479552
C	4.131643	0.985509	-1.077784
C	5.170887	1.329198	-1.978809
C	6.822109	0.759412	-0.289198
C	6.535137	1.219037	-1.556304
N	4.869842	1.743620	-3.246167
C	5.903365	2.236289	-4.143371
C	3.498598	2.068938	-3.603136
C	-5.482933	1.577345	0.964422
N	-4.282330	1.169565	1.448796
C	-6.329533	2.384130	1.723713
C	-3.906247	1.555946	2.677862
C	-5.937207	2.778980	2.996517
C	-4.702752	2.357132	3.482692
C	-5.796792	1.101112	-0.390768
C	-6.988914	1.379827	-1.057782
N	-4.824397	0.354348	-0.974047
C	-7.187042	0.884517	-2.340389
C	-5.021582	-0.123346	-2.212599
C	-6.184658	0.118530	-2.928881
Ru	-3.148629	0.017153	0.175962
H	-2.026248	-4.577873	-1.637302

H	-1.070879	0.201779	-2.110659
H	-0.334382	-3.864724	-3.299392
H	0.161432	-1.408673	-3.545733
H	-3.547928	-5.013311	-0.266306
H	-5.269599	-5.355304	1.481056
H	-5.221128	-1.110638	2.173871
H	-6.136866	-3.352156	2.734838
H	0.807357	3.157404	0.450684
H	-3.546688	2.493160	-1.667884
H	-2.244359	4.516936	-2.242970
H	1.516281	1.451922	1.384744
H	-1.959221	-1.817678	2.420593
H	0.253235	-2.098264	3.462452
H	0.996815	4.917290	-2.051021
H	-0.336699	6.013147	-1.663560
H	0.822848	5.527723	-0.404162
N	2.736049	-1.790336	5.224964
H	5.099970	-0.751572	3.757894
H	8.006249	-0.926148	1.854931
H	7.971671	0.728843	2.458869
H	7.438434	-0.602349	3.504718
H	7.864146	0.672930	0.005785
H	-7.285419	2.704789	1.327138
H	-2.938033	1.200996	3.012208
H	-6.586466	3.406835	3.597710
H	-4.353280	2.640431	4.469219
H	-7.757961	1.975811	-0.581668
H	-8.110438	1.093911	-2.870196
H	-4.213285	-0.712057	-2.631021
H	-6.293079	-0.290091	-3.927286
C	2.529564	1.077166	-2.987410
H	3.426687	2.049992	-4.694465
H	3.252265	3.092815	-3.276503
C	2.682428	1.082535	-1.474817
H	2.737801	0.076863	-3.385482
H	1.505102	1.340164	-3.268902
H	2.112719	0.262898	-1.025257
H	2.256525	2.011191	-1.073181
C	7.207440	1.488016	-3.942302
H	5.539982	2.104855	-5.167414
H	6.058467	3.316540	-3.988566
C	7.645312	1.621012	-2.491981
H	7.966748	1.892776	-4.618003
H	7.062686	0.431529	-4.199110
H	7.924975	2.665624	-2.295593
H	8.535586	1.016346	-2.290816

SCV49

C	-2.448130	-2.559305	-1.003537
N	-2.167465	-1.238644	-1.141377
C	-1.802850	-3.517754	-1.783820
C	-1.252091	-0.855352	-2.044983
C	-0.854688	-3.115714	-2.716234
C	-0.574559	-1.758980	-2.850241
C	-3.464015	-2.875095	0.012025
C	-3.930905	-4.163157	0.270248
N	-3.934522	-1.806511	0.704811
C	-4.895171	-4.357646	1.251159
C	-4.868269	-1.999581	1.649135
C	-5.373337	-3.255205	1.953476
C	-0.919334	1.909832	0.201590
N	-2.130176	1.732698	-0.383417
C	-0.150393	3.040178	-0.054466
C	-2.573502	2.669493	-1.234815
C	-0.593164	4.011104	-0.954210
C	-1.840441	3.802691	-1.549104
C	-0.502754	0.822750	1.101523
C	0.770856	0.729235	1.641310
N	-1.451590	-0.116519	1.344640
C	1.114157	-0.347532	2.471853
C	-1.156448	-1.108976	2.199383
C	0.087262	-1.252011	2.786794
C	0.251394	5.202689	-1.280255
C	2.469870	-0.529198	2.988796
C	2.619540	-1.167451	4.242161
C	3.627580	-0.207394	2.285046
O	3.451717	0.264356	1.038110
C	4.952360	-0.373630	2.770027
C	4.489668	0.556522	0.196437
C	6.011846	-0.067634	1.963865
C	5.820961	0.409158	0.633868
C	7.409732	-0.244070	2.499721
C	4.130755	0.986542	-1.068574

C	5.170021	1.322658	-1.975856
C	6.828276	0.745693	-0.293207
C	6.536222	1.202567	-1.558019
N	4.867836	1.742626	-3.235462
C	5.900719	2.193634	-4.157286
C	3.495169	2.062152	-3.597495
C	-5.481773	1.573572	0.962462
N	-4.279932	1.168425	1.445849
C	-6.330332	2.376836	1.723218
C	-3.904752	1.553755	2.675479
C	-5.938782	2.770786	2.996546
C	-4.703182	2.351492	3.481821
C	-5.795171	1.098403	-0.393215
C	-6.988292	1.375301	-1.059094
N	-4.821553	0.354665	-0.978144
C	-7.186092	0.881232	-2.342240
C	-5.018332	-0.121862	-2.217169
C	-6.182410	0.118356	-2.932401
Ru	-3.143740	0.020303	0.170246
H	-2.031530	-4.569668	-1.663315
H	-1.067940	0.210287	-2.117361
H	-0.343320	-3.852202	-3.327186
H	0.156848	-1.396311	-3.563809
H	-3.550115	-5.008451	-0.289985
H	-5.266840	-5.355090	1.461262
H	-5.208337	-1.113805	2.173231
H	-6.126208	-3.356187	2.727092
H	0.796559	3.179802	0.455414
H	-3.546042	2.491465	-1.679555
H	-2.250866	4.520376	-2.252022
H	1.506019	1.487012	1.411753
H	-1.946987	-1.825052	2.393530
H	0.263262	-2.092642	3.449692
H	0.991486	4.934015	-2.043574
H	-0.351424	6.024438	-1.673143
H	0.799999	5.551806	-0.401439
N	2.721948	-1.692879	5.279100
H	5.095280	-0.739369	3.778454
F	8.115502	-1.119878	1.756171
F	8.092082	0.918710	2.487397
F	7.424895	-0.696703	3.761716
H	7.871232	0.654589	-0.007899
H	-7.287236	2.695416	1.327467
H	-2.935815	1.200959	3.009886
H	-6.589630	3.395908	3.598854
H	-4.354231	2.633975	4.468749
H	-7.758384	1.968925	-0.581767
H	-8.110291	1.089269	-2.871167
H	-4.209116	-0.708106	-2.637184
H	-6.290466	-0.289173	-3.931277
C	2.525790	1.076479	-2.973383
H	3.425116	2.032760	-4.688366
H	3.251351	3.088924	-3.280744
C	2.681386	1.091255	-1.461329
H	2.730192	0.073084	-3.365288
H	1.501916	1.341263	-3.254601
H	2.108046	0.277911	-1.005485
H	2.264314	2.025896	-1.064349
C	7.198008	1.437100	-3.946646
H	5.525581	2.035716	-5.173095
H	6.066825	3.275647	-4.034975
C	7.642856	1.593450	-2.501031
H	7.957370	1.823129	-4.632879
H	7.043961	0.377219	-4.182850
H	7.919354	2.641699	-2.320507
H	8.534788	0.993739	-2.293754

14. References

[1] J. M. Haider, Z. Pikramenou. Metal Assembly of Cyclodextrin Recognition Sites. *Eur. J. Inorg. Chem.* **2001**, 1892194.
[2] H. Nishide, N. Shimidzu, E. Tsuchida, Eishun. Chelating resin: pyridine derivatives attached to poly(styrene) beads with spacer group. *J. Appl. Polym. Sci*. **1982**, *27*, 4161-4169.
[3] A. Gandioso, R. Bresoli-Obach, A. Nin-Hill, M. Bosch, M. Palau, A. Galindo, S. Contreras, A. Rovira, C. Rovira, S. Nonell, V. Marchán. Redesigning the coumarin scaffold into small bright fluorophores with far-red to NIR emission and large Stokes' shifts useful for cell imaging. *J. Org. Chem.* **2018**, *83*, 1185-1195.
[4] E. Ortega-Forte, A. Rovira, M. López-Corrales, A. Hernández-García, F. J. Ballester, E. Izquierdo-García, M. Jordà-Redondo, M. Bosch, S. Nonell, M. D. Santana, J. Ruiz, V. Marchán, G. Gasser. A near-infrared light-activatable Ru(II)-coumarin photosensitizer active under hypoxic conditions. *Chem. Sci*. **2023**, *14*, 7170–7184.
[5] D. Magde, J. H. Brannon, T. L. Cremers, J. Olmsted. Absolute luminescence yield of cresyl violet. A standard for the red. *J. Phys. Chem*. **1979**, *83*, 696-699.
[6] C. Adamo, V. Barone. Toward reliable density functional methods without adjustable parameters: The PBE0 model. *J. Chem. Phys.* **1999**, *110*, 6158-6170.
[7] D. Andrae, U. Häußermann, M. Dolg, H. Stoll, H. Preuß. Energy-Adjusted ab initio Pseudopotentials for the Second and Third Row Transition Elements. *Theor Chim Acta* **1990**, *77*, 123–141.
[8] M. E. Casida, in *Recent Advances in Density Functional Methods (Part I)* (Ed.: D.P. Chong), World Scientific, Sin, 1995, pp. 155–192.
[9] Y. Zhao, D. G. Truhlar. The M06 suite of density functionals for main group thermochemistry, thermochemical kinetics, noncovalent interactions, excited states, and transition elements: two new functionals and systematic testing of four M06-class functionals and 12 other functionals. *Theor. Chem. Acc.* **2008**, *120*, 215–241.
[10] S. Hirata, M. Head-Gordon, Time-dependent density functional theory within the Tamm–Dancoff approximation. *Chem. Phys. Lett.* **1999**, *314*, 291–299.
[11] M. Cossi, N. Rega, G. Scalmani, V. Barone. Energies, structures, and electronic properties of molecules in solution with the C-PCM solvation model. *J. Comput. Chem.* **2003**, *24*, 669–681.
[12] J. Roque III, H. Cole, P. Barrett, L. Lifshits, R. Hodges, S. Kim, A. Francés-Monerris, M. E. Alberto, C. Cameron, S. McFarland. Intraligand Excited States Turn a Ruthenium Oligothiophene Complex into a Light-Triggered Ubertoxin with Anticancer Effects in Extreme Hypoxia. *J. Am. Chem. Soc.* **2022**, *144*, 8317–8336.
[13] H. D. Cole, A. Vali, J. A. I. I. I. Roque, G. Shi, G. Kaur, R. O. Hodges, A. Francés-Monerris, M. E. Alberto, C. G. Cameron, S. A. McFarland. Ru(II) Phenanthroline-Based Oligothienyl Complexes as Phototherapy Agents. *Inorg. Chem.* **2023**, *62*, 21181–21200.
[14] H. D. Cole, A. Vali, J. A. I. I. I. Roque, G. Shi, A. Talgatov, G. Kaur, A. Francés-Monerris, M. E. Alberto, C. G. Cameron, S. A. McFarland. Ru(II) Oligothienyl Complexes with Fluorinated Ligands: Photophysical, Electrochemical, and Photobiological Properties. *Inorg. Chem.* **2024**, *63*, 9735–9752.
[15] M. J. Frisch, G. W. Trucks, H. B. Schlegel, G. E. Scuseria, M. A. Robb, J. R. Cheeseman, G. Scalmani, V. Barone, G. A. Petersson, H. Nakatsuji, X. Li, M. Caricato, A. V. Marenich, J. Bloino, B. G. Janesko, R. Gomperts, B. Mennucci, H. P. Hratchian, J. V. Ortiz, A. F. Izmaylov, J. L. Sonnenberg, D. Williams-Young, F. Ding, F. Lipparini, F. Egidi, J. Goings, B. Peng, A. Petrone, T. Henderson, D. Ranasinghe, V. G. Zakrzewski, J. Gao, N. Rega, G.

Zheng, W. Liang, M. Hada, M. Ehara, K. Toyota, R. Fukuda, J. Hasegawa, M. Ishida, T. Nakajima, Y. Honda, O. Kitao, H. Nakai, T. Vreven, K. Throssell, J. A. , Jr. Montgomery, J. E. Peralta, F. Ogliaro, M. J. Bearpark, J. J. Heyd, E. N. Brothers, K. N. Kudin, V. N. Staroverov, T. A. Keith, R. Kobayashi, J. Normand, K. Raghavachari, A. P. Rendell, J. C. Burant, S. S. Iyengar, J. Tomasi, M. Cossi, J. M. Millam, M. Klene, C. Adamo, R. Cammi, J. W. Ochterski, R. L. Martin, K. Morokuma, O. Farkas, J. B. Foresman, D. J. Fox, "Gaussian 16, Gaussian, Inc., Wallingford CT, 2016," 2016.

[16] R. L. Martin. Natural transition orbitals. *J. Chem. Phys.* **2003**, *118*, 4775–4777.

[17] T. Etienne, X. Assfeld, A. Monari. Toward a Quantitative Assessment of Electronic Transitions' Charge-Transfer Character. *J. Chem. Theory. Comput.* **2014**, *10*, 3896–3905.

[18] T. Etienne, X. Assfeld, A. Monari, *J. Chem. Theory Comput.* **2014**, *10*, 3906–3914.

[19] L. Skripnikov, 2020, Chemissian 4.67. www.chemissian.com.

[20] F. Plasser. TheoDORE: A toolbox for a detailed and automated analysis of electronic excited state computations. *J. Chem. Phys.* **2020**, *152*, 084108.

[21] R. Schmidt, E. Afshari. Comment on "Effect of solvent on the phosphorescence rate constant of singlet molecular oxygen (1.DELTA.g)". *J. Phys. Chem.* **1990**, *94*, 10, 4377–4378.

[22] K. El-Naggar, H. S. Abdel-Samad, R M. Ramadan, M. E. El-Khouly, A A. Abdel-Shafi. Participation of fractional charge transfer on the efficiency of singlet oxygen production: Heteroleptic ruthenium (II) bipyridine derivatives. *J. Photochem. Photobiol., A* **2023**, *436*, 114405.

[23] a) N. Adarsh, R. R. Avirah, D. Ramaiah. Tuning photosensitized singlet oxygen generation efficiency of novel aza-BODIPY dyes. *Org. Lett*. **2010**, *12*, 5720-5723. b) W. Li, L. Li, H. Xiao, R. Qi, Y. Huang, Z. Xie, X. Jing, H. Zhang. Iodo-BODIPY: a visible-light-driven, highly efficient and photostable metal-free organic photocatalyst. *RSC Adv*. **2013**, *3*, 13417-13421. c) Z. Lv, H. Wei, Q. Li, X. Su, S. Liu, K. Y. Zhang, W. Lv, Q. Zhao, X. Li, W. Huang. Achieving efficient photodynamic therapy underboth normoxia and hypoxia using cyclometalated Ru(II) photosensitizer through type I photochemicalprocess. *Chem. Sci*. **2018**, *9*, 502-512.

[24] M. López-Corrales, A. Rovira, A. Gandioso, M. Bosch, S. Nonell, V. Marchán. Transformation of COUPY Fluorophores into a Novel Class of Visible-Light-Cleavable Photolabile Protecting Groups. *Chem. Eur. J.* **2020**, *26*, 16222-16227.

[25] X. Zhang, G. Q. Zhang, J. Zhu, J. Methylated unsymmetric BODIPY compounds: synthesis, high fluorescence quantum yield and long fluorescence time. *J. Fluoresc*. **2019**, *29*, 407-416.

[26] E. K. Pefkianakis, D. Christodouleas, D. L. Giokas, K. Papadopoulos, G.C. Vougioukalakis. A family of Ru II photosensitizers with high singlet oxygen quantum yield: synthesis, characterization, and evaluation. *Eur. J. Inorg. Chem*. **2013**, 4628–4635.

[27] U. Yoshiharu. Determination of quantum yield of singlet oxygen formation by photosensitization. *Chem. Lett*. **1973**, *2*, 743-744.

[28] J. Llano, J. Raber, L. A Eriksson. Theoretical study of phototoxic reactions of psoralens. *J. Photochem. Photobiol., A*, **2003**, *154*, 235–243.

[29] J. A. Roque, P. C. Barrett, H. D. Cole, L. M. Lifshits, G. Shi, S. Monro, D. von Dohlen, S. Kim, N. Russo, G. Deep, C. G. Cameron, M. E. Alberto, S. A. McFarland. Breaking the barrier: an osmium photosensitizer with unprecedented hypoxic phototoxicity for real world photodynamic therapy. *Chem. Sci.* **2020**, *11*, 9784–9806.

[30] M. Ayoubi-Chianeh, F. Jafarpour. Theoretical study of new promising conjugated psoralens in psoralen ultraviolet A therapy. *J. Phys. Org. Chem*. **2022**, *35*, e4308.

[31] M. Spiegel, C. Adamo. Tuning the Photophysical Properties of Ru(II) Photosensitizers for PDT by Protonation and Metallation: A DFT Study. *J. Phys. Chem. A* **2023**, *127*, 3625–3635.

[32] J. Schindelin, I, Arganda-Carreras, E. Frise, V. Kaynig, M. Longair, T. Pietzsch, S. Preibisch, C. Rueden, S. Saalfeld, B. Schmid, J. Y. Tinevez, D. J. White, V. Hartenstein, K. Eliceiri, P. Tomancak, A. Cardona. Fiji: an open-source platform for biological-image analysis. *Nat. Methods* **2012**, *9*, 676−682.
[33] A. Gandioso, E. Izquierdo-García, P. Mesdom, P. Arnoux, N. Demeubayeva, P. Burckel, B. Saubaméa, M. Bosch, C. Frochot, V. Marchán, G. Gasser. Ru(II)-Cyanine Complexes as Promising Photodynamic Photosensitizers for the Treatment of Hypoxic Tumours with Highly Penetrating 770 nm Near-Infrared Light. *Chem. Eur. J*. **2023**, *29*, e202301742.
